# Persistent four-coordinate iron-centered radical stabilized by π-donation†
†Electronic supplementary information (ESI) available: Experimental, crystallographic, computational details, and crystal data for **2**, **4**, **5** and **8**. CCDC 1057111–1057113 and 1425703. For ESI and crystallographic data in CIF or other electronic format see DOI: 10.1039/c5sc02601f



**DOI:** 10.1039/c5sc02601f

**Published:** 2015-09-25

**Authors:** Yusuke Sunada, Shintaro Ishida, Fumiya Hirakawa, Yoshihito Shiota, Kazunari Yoshizawa, Shinji Kanegawa, Osamu Sato, Hideo Nagashima, Takeaki Iwamoto

**Affiliations:** a Institute for Materials Chemistry and Engineering , Kyushu University , 6-1 Kasugakoen , Kasuga , Fukuoka 816-8580 , Japan; b Department of Chemistry , Graduate School of Science , Tohoku University , Aoba-ku , Sendai 980-8578 , Japan; c Institute for Materials Chemistry and Engineering , Kyushu University , Nishi-ku , Fukuoka 819-0395 , Japan; d CREST , Japan Science and Technology Agency (JST) , 6-1 Kasugakoen , Kasuga , Fukuoka 816-8580 , Japan

## Abstract

Dinuclear iron carbonyl complex **2**, which contains an elongated unsupported Fe–Fe bond, was synthesized by the reaction between Fe_2_(CO)_9_ and phosphinyl radical **1**. Thermal Fe–Fe bond homolysis led to the generation of a four-coordinate carbonyl-based iron-centered radical, **3**, which is stabilized by π-donation. Complex **3** exhibited high reactivity toward organic radicals to form diamagnetic five-coordinate Fe(ii) complexes.

## Abstract 2

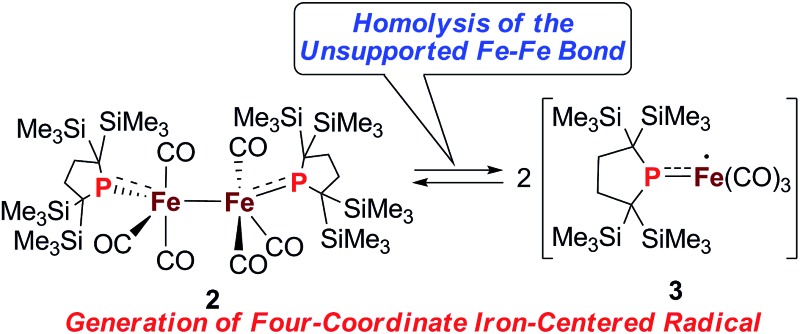
Generation of four-coordinate iron-centered radical **3** was realized by the thermal homolysis of the unsupported Fe–Fe bond of **2**.

## Introduction

**Chart 1:**
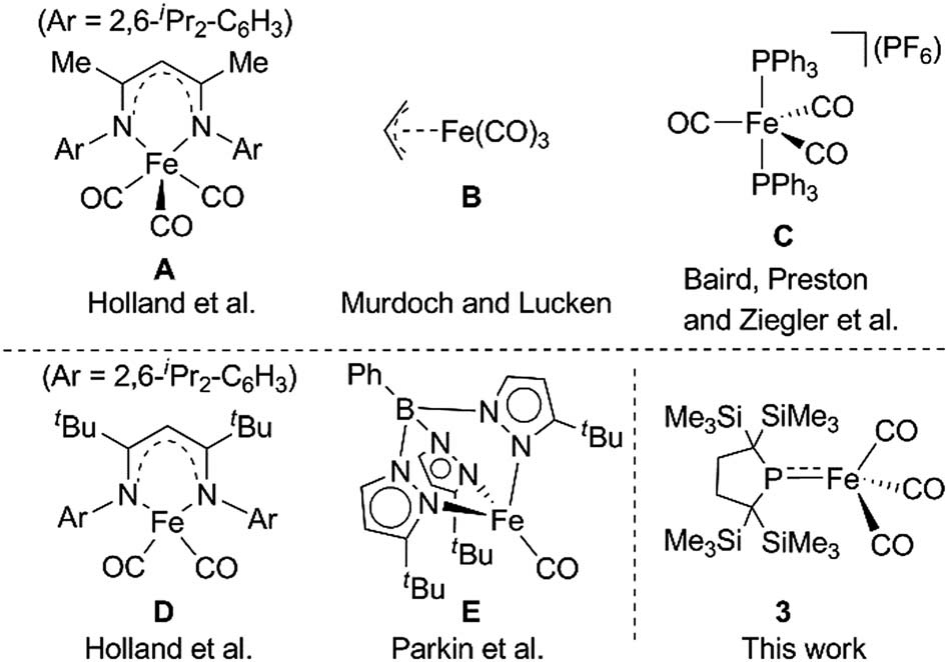
Selected examples of five coordinate Fe(i) species (**A–C**) (upper) and previously reported four coordinate Fe(i) complexes **D** and **E** (lower).

**Scheme 1:**
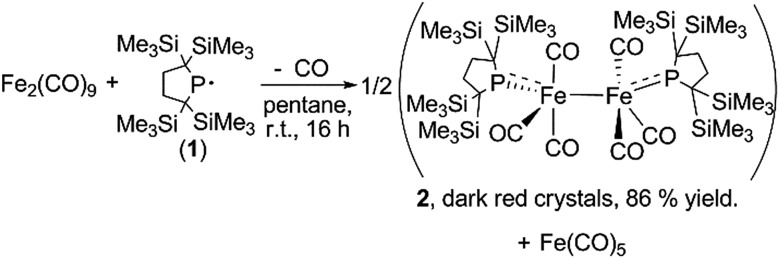
Synthesis of dinuclear iron carbonyl complex **2**.

The diverse reactivity of transition metal-centered radicals towards various organic substrates has garnered significant interest in both organometallic and catalytic synthetic chemistry over the past several decades.^1,2^ Among the transition metal complexes, iron carbonyl based five coordinate iron(i) species such as **A–C** shown in Chart 1 have received particular attention owing to their catalytic activity and unique properties.^3–6^ Thus, construction of coordinatively unsaturated iron(i) carbonyl complexes is expected to facilitate the development of novel organometallic and catalytic reactions. However, few examples of four or less coordinate iron(i) carbonyl complexes have been explored.^7–9^ Holland *et al.* described the synthesis of a four-coordinate Fe(i) dicarbonyl complex **D** in which the iron center adopts an *S* = 1/2 electronic configuration.^8^ Parkin *et al.* synthesized a four-coordinate Fe(i) monocarbonyl complex **E** using a tris(pyrazolyl)borate ligand.^9^ Chelating multidentate ligands bearing sterically hindered substituents are needed to stabilize these coordinatively unsaturated species.‡
‡Although complex **D** in Chart 1 can capture atmospheric CO, no further reactions of four coordinated iron carbonyl based Fe(i) complexes have been reported. We have attempted to construct a reactive, coordinatively unsaturated, iron carbonyl based iron-centered radical based on an alternative synthetic strategy involving stabilization by ligand-to-metal π-donation. Phosphinyl radical **1**,^10^ shown in Scheme 1, was thought to be appropriate for this purpose, as it exhibits reactivity towards transition metal complexes, and the lone pair of electrons on the phosphorus center can effectively stabilize the low-coordinate metal complex *via* π-donation.^10*b*^ In this paper, we report that **1** functions as an effective ligand to form the dinuclear iron carbonyl complex **2** with an unsupported Fe–Fe bond. Complex **2** was a suitable precursor for the generation of four-coordinate iron-centered radical **3** *via* homolysis of the Fe–Fe bond. Reactions of **3** with organic radicals are also reported.

A yellow suspension of Fe_2_(CO)_9_ in pentane gradually dissolved to give a dark red solution during the course of the reaction with **1** at room temperature for 16 h. From the dark red solution, complex **2** was obtained in 86% yield (based on **1**) (Scheme 1). The reaction was accompanied by the formation of Fe(CO)_5_, which was confirmed by IR spectroscopy. In the course of the reaction, radical **1** formally underwent a one-electron reduction to form a monoanionic phosphido ligand. As a closely related example to this reaction, Cowley *et al.* prepared a five-coordinate iron-centered radical *via* the reaction between Fe_2_(CO)_9_ and [{(Me_3_Si)_2_CH}_2_P]˙. However, the molecular structure of this complex was not determined crystallographically.^11^

**Fig. 1:**
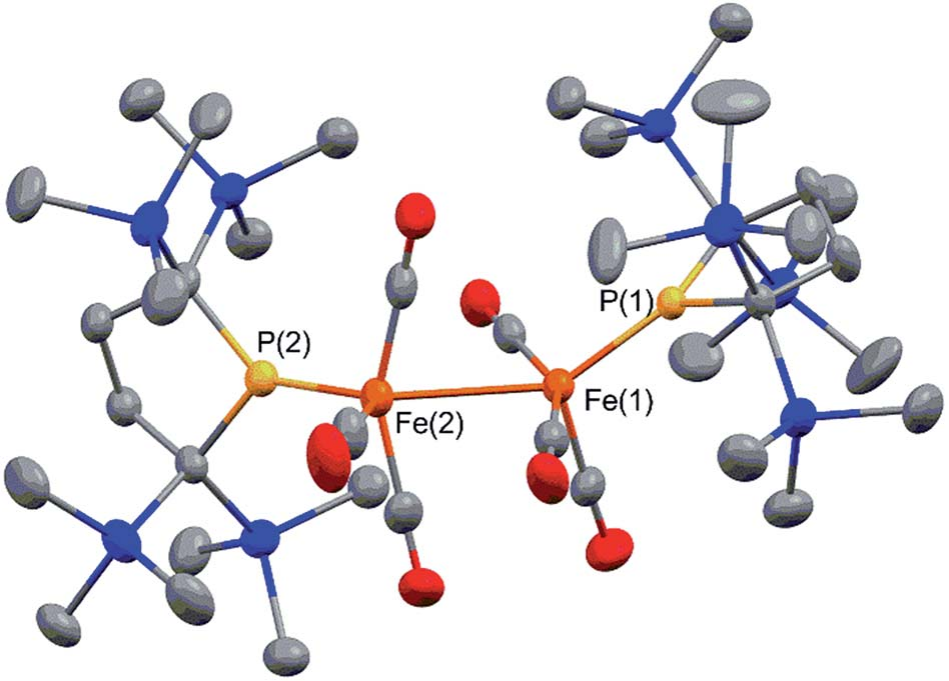
Molecular structure of **2** with 50% probability ellipsoids.

Complex **2** consists of two trigonal-bipyramidal iron centers with six CO ligands (Fig. 1). The distances between the iron and the carbon atoms of the CO ligands coordinated to the other iron center were within 2.932(4)–3.825(6) Å, which was too long for bonding interactions. Thus, complex **2** has an unsupported Fe–Fe bond. There are only two examples in the literature of structurally characterized coordinatively unsaturated neutral dinuclear iron complexes,^12^ *i.e.*, [Fe(tim)]_2_ (tim = 2,3,9,10-tetramethyl-1,4,8,11-tetraazacyclotetradeca-1,3,8,10-tetraene)^12*a*^ and [(3,5-^i^Pr_2_–Ar*)Fe–FeCp(CO)_2_] ((3,5-^i^Pr_2_–Ar*) = C_6_H–2,6–(C_6_H_2_–2,4,6-^i^Pr_3_)_2_–3,5–^i^Pr_2_–Ar*).^12*b*^ The Fe–Fe bond distance in these complexes (2.6869(6)^12*a*^ and 2.3931(8)^12*b*^ Å) are significantly shorter than that of **2** (2.7374(10) Å), which reflects the weak bonding interaction between the two iron centers in **2** (*vide infra*). The sum of the three angles around P(1) and P(2) are 358.6° and 358.1°, respectively, indicating planarity at P, which is consistent with π-bonding. The short Fe–P bond lengths, 2.0934(12) Å for Fe–P(1) and 2.1047(13) Å for Fe(2)–P(2), also support the multiple bond character of the Fe–P bond (*vide infra*).^14^

Complex **2** is diamagnetic, and the ^1^H NMR of **2** confirms the molecular structure. The formal oxidation state of the two iron centers in **2** can be considered as Fe(i), and an intramolecular antiferromagnetic coupling between two adjacent iron centers makes complex **2** diamagnetic. A sharp singlet appears at 0.35 ppm in toluene-d_8_ at 293 K, whereas four slightly broad singlets are observed at 0.31, 0.35, 0.37, and 0.48 ppm with an integral ratio of 1 : 1 : 1 : 1 at 193 K. These signals are attributed to the magnetically inequivalent SiMe_3_ moieties in **2**. Notably, the ^31^P NMR spectrum shows one significantly downfield shifted peak at 425.9 ppm, which is typically seen for planar sp^2^-phosphorus species.^15^ This ^31^P peak, as well as the planar geometry around P and the short Fe–P bond distances observed in X-ray crystallography, strongly suggest that the phosphido ligands formally function as LX-type ligands with the aid of π-donation.

**Scheme 2:**
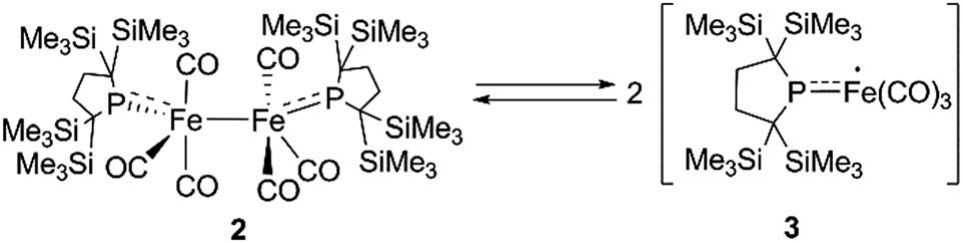
Equilibrium between **2** and **3**.

At higher temperatures (293–353 K), one sharp singlet for the SiMe_3_ group and one doublet due to the CH_2_ moiety of the backbone of the phosphido ligand are observed in the ^1^H NMR of **2**. Additionally, it should be noted that the appearance of a broad signal is confirmed at ∼3–5 ppm. The intensity of this broad peak increases with temperature (Fig. S5-2 in ESI†). Considering the elongated Fe–Fe bond in **2**, we hypothesized that the broad peak observed at higher temperatures could be ascribed to the generation of paramagnetic species **3** *via* the homolytic cleavage of the Fe–Fe bond, as shown in Scheme 2. The generation of a radical species is supported by ESR spectroscopy; the ESR spectrum of **2** in toluene exhibits one broad signal at *g* = 2.0519 (*A*(^31^P) = 3.43 mT) at 293 K. The intensity of this peak gradually increases upon heating, indicating that the concentration of the ESR-active species significantly increases with temperature (Fig. S6-1†). The observed *g*-value is comparable to those reported for iron carbonyl-based five-coordinate seventeen electron radicals.^1a,3b,3c^ In addition, the ESR spectrum of a flash-frozen toluene solution measured at 77 K showed a rhombic signal (*g* = 2.0838, 2.0573, 2.0409), suggesting that the iron species generated *in situ* has an *S* = 1/2 ground state (Fig. S6-2†).

**Fig. 2:**
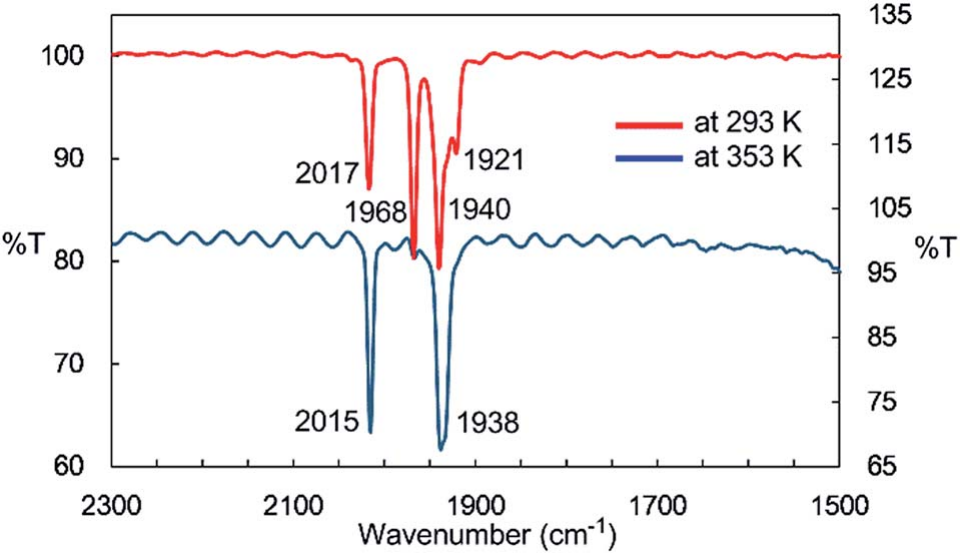
IR spectra of the *n*-octane solution of **2** at 293 K and 353 K.

IR spectroscopy provided more detailed information about the species generated at higher temperatures. The solid-state IR (ATR) spectrum of **2** features bands at 2014, 1963, 1932, and 1916 cm^–1^, which are attributed to terminal CO ligands (Fig. S7-1†). In the IR spectrum of **2** in *n*-octane at 293 K, four *ν*_CO_ absorptions appear at 2017, 1968, 1940, and 1921 cm^–1^, indicating the presence of dinuclear complex **2** in solution at this temperature (Fig. 2). An absorption band assignable to the bridging CO ligand is not observed in the solid or solution states. The solution spectrum at 293 K includes a shoulder at around 1938 cm^–1^ together with the four aforementioned *ν*_CO_ absorption bands. At 353 K in *n*-octane in the dark, this band represents the major absorption and is accompanied by a strong band at 2015 cm^–1^, and the four CO bands observed at 293 K are almost completely absent (Fig. 2). If we assume that three CO ligands are maintained on the iron center during heating, this spectral change could be explained by the formation of an iron tricarbonyl species with *C*_3v_ symmetry (*vide infra*).

**Fig. 3:**
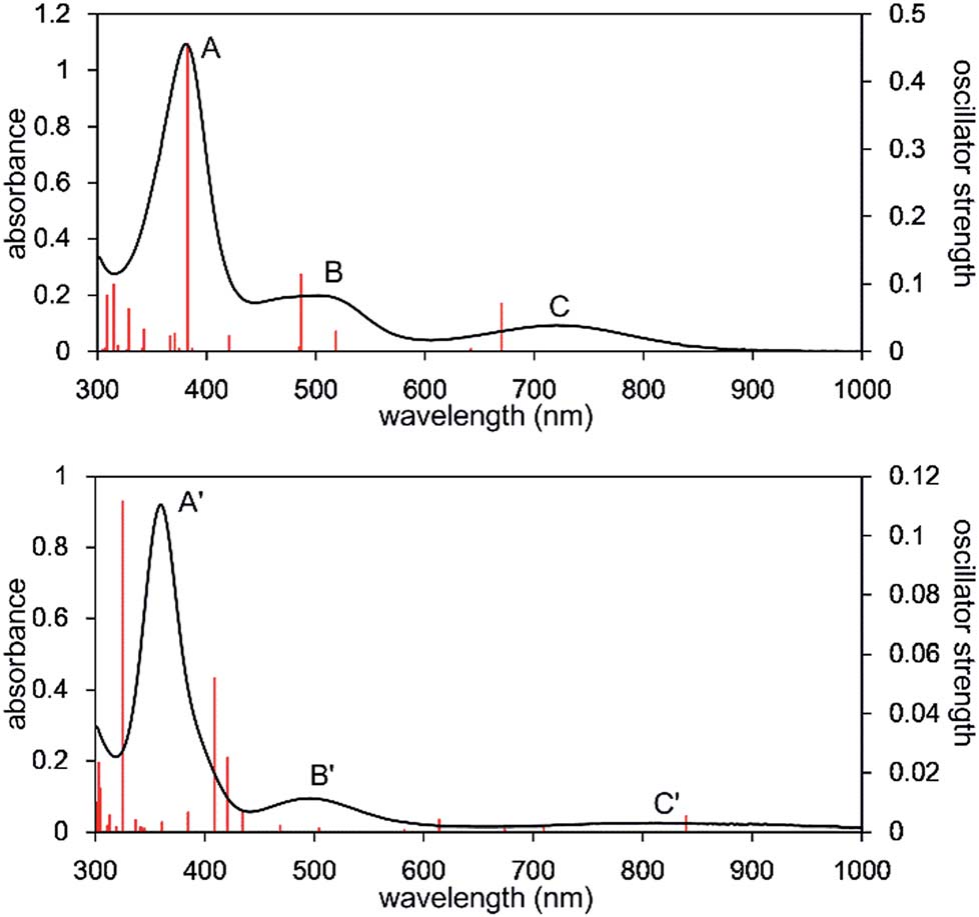
UV-Vis-NIR spectra of the *n*-octane solution of **2** measured at 293 K (upper) and 353 K (lower). Solid red bars indicate the calculated transition of the **2_opt_** (upper) and **3_opt_** (lower).

The UV-Vis-NIR spectrum of an *n*-octane solution of **2** at 293 K displays an intense band (A) at 380 nm and two relatively weak bands (B and C) at 502 and 720 nm, respectively (Fig. 3, upper). Their intensity progressively decreases with heating, and three new bands, namely A′ (362 nm), B′ (496 nm), and C′ (818 nm) are exclusively observed at 353 K (Fig. 3, lower). After cooling the solution to 293 K, the original bands, A, B, and C, gradually reappear over time, and equilibrate after 5 h. Notably, an isosbestic point is observed at 810 nm, providing good evidence for an equilibrium between two species (Fig. S8-3†).

In order to gain further insight into the electronic structure and bonding in the iron complexes, a theoretical investigation of **2** was undertaken. Geometry optimization and time-dependent density functional theory (TD-DFT) spectral calculation were carried out at the PBE0, CAM-B3LYP, and B3LYP levels, and the PBE0 functional was found to give the most satisfactory results.§
§The ground state of **2_opt_** was estimated to be the closed-shell singlet in all calculations. Calculated bond lengths and angles at the PBE0 and CAM-B3LYP levels are similar to experimental values; however, the structural parameters at the B3LYP level are inconsistent with the experimental results (see Table S2 in ESI†). The results of TD-DFT calculations at the CAM-B3LYP and B3LYP levels are also reported in ESI (Fig. S24†).


**Fig. 4:**
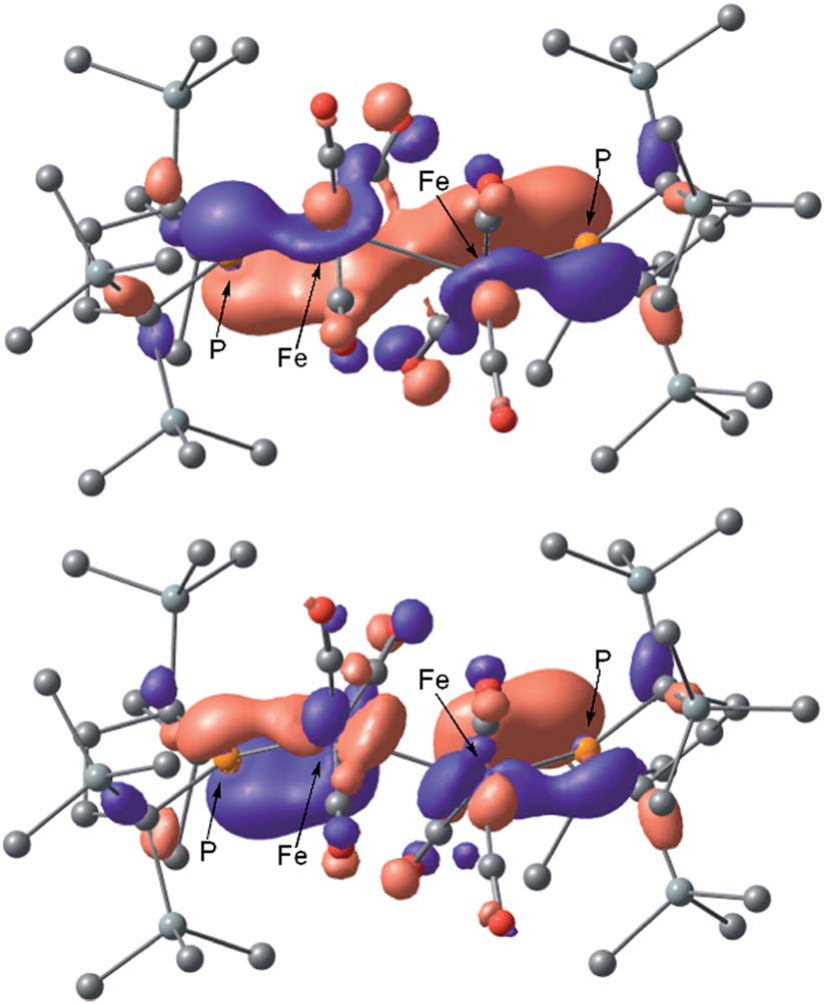
Graphical representations of the DFT(PBE0)-derived HOMO (upper) and HOMO – 1 (lower) for **2_opt_**.

The calculated spectrum of **2_opt_** is given in Fig. 3 (upper). Although a low energy absorption band is predicted to appear at 670 nm, which is blue-shifted by 50 nm as compared to the actual spectrum presumably due to the overestimation of the low-energy gap, the TD-DFT analysis of **2_opt_** is in reasonable agreement with the actual spectrum. The Mayer Fe–Fe bond order is calculated to be 0.33. The bond order between Fe and P is calculated to be 1.4, indicating this bond to have a double bond nature. Molecular orbital analysis of HOMO and HOMO – 1 of **2_opt_** highlights the π-bonding nature of the Fe–P bond (Fig. 4). This π-bonding nature was also confirmed by density distribution analysis in HOMO and HOMO – 1 (Fig. S17†).

**Fig. 5:**
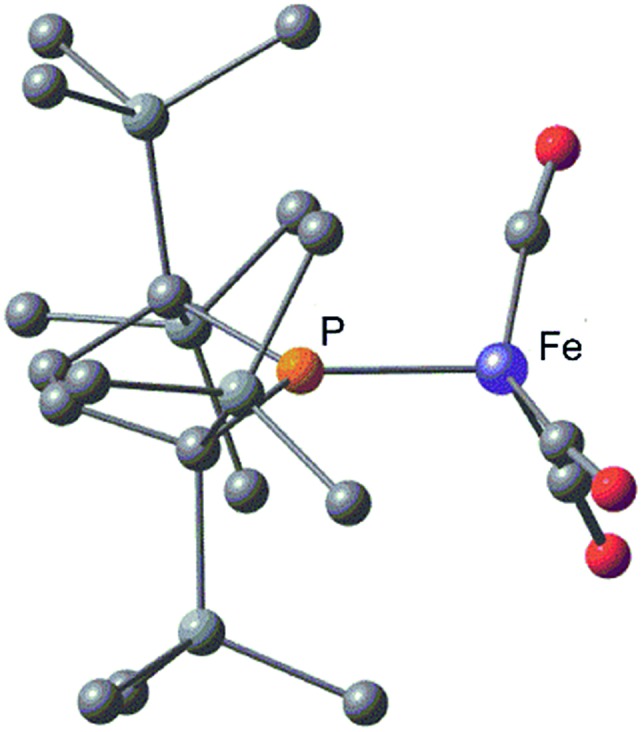
DFT(PBE0) optimized structure of **3_opt_**.

**Fig. 6:**
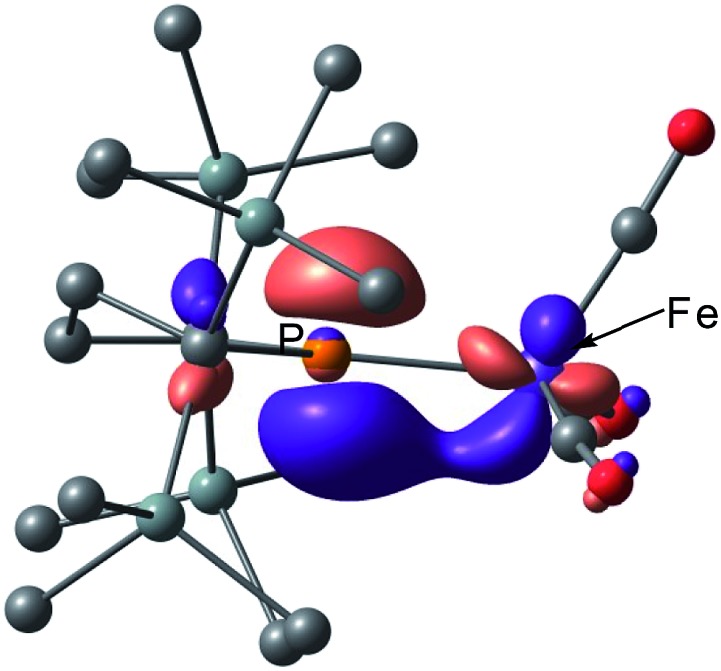
Graphical representations of the DFT(PBE0)-derived βHOMO for **3_opt_**.

Next, we calculated the molecular and electronic structures of **3** at the PBE0 level. We found that **3_opt_** possesses a slightly distorted *C*_3v_ tetrahedral coordination geometry in the open-shell doublet ground state (Fig. 5). Interestingly, DFT studies of **3_opt_** indicate that the spin densities around Fe and P are 1.50 and –0.52, respectively. The bonding orbital in **3** was carefully analysed, and the orbital interactions in **3_opt_** were depicted in Fig. S20.† We found that βHOMO consists of the d_*yz*_ orbital in iron and p_*y*_ orbital in phosphorus, and π-bonding character was confirmed in the βHOMO by molecular orbital analysis of **3_opt_** (Fig. 6). In contrast, αHOMO consists of d_*x* ^2^–*y* ^2^_ orbital in iron leading to a non-bonding interaction (molecular orbital details are given in the ESI, Fig. S19 and S20†). This non-bonding nature was also confirmed by density distribution in αHOMO (Fig. S21†). This partial Fe–P π-bonding is consistent with the estimated Mayer bond order for Fe–P (1.2). These results indicate that electron donation from the P atom to Fe found in the βHOMO electronically stabilizes the four-coordinate iron-centered radical. TD-DFT calculations based on **3_opt_** give the absorption bands shown in Fig. 3 (lower). Although the accuracy of the TD-DFT calculation of **3_opt_** appears to be limited due to the open-shell nature of **3_opt_**,^16^ the obtained predicted spectrum roughly approximates the actual spectrum. The two remarkable peaks in the range of 300–550 nm in the predicted spectrum of **3_opt_** are blue-shifted compared with those of **2_opt_**. A similar feature is also observed in the actual spectrum of **3**. It should be noted that one peak appears at 839 nm in the predicted spectrum of **3_opt_**, which effectively reproduces the actual band observed at 818 nm (band C′ in Fig. 3 (lower)). The electron density difference map of **2_opt_** and **3_opt_** indicates that these absorptions originate from the metal-to-ligand charge transfer (MLCT) transitions (Fig. S22 and S23†). It should be mentioned that Lappert *et al.* have reported the synthesis of four coordinated Co(i) tricarbonyl, [{(Me_3_Si)_2_N}(^i^Pr_2_N)P]Co(CO)_3_, by the reaction of the *in situ* generated phosphinyl radical [{(Me_3_Si)_2_N}(^i^Pr_2_N)P]˙ with Co_2_(CO)_8_.^17^ This cobalt(i) phosphido complex also adopts tetrahedral coordination geometry, as shown by X-ray diffraction analysis. Thus, complex **3** can be considered analogous to Lappert's cobalt(i) phosphido complex minus one electron.

Considering the experimental and theoretical results, it is highly probable that the generation of four-coordinate iron-centered radical **3** takes place *via* the homolysis of the unsupported Fe–Fe bond of **2** in solution. The coordination geometry of **3** was predicted to be slightly distorted tetrahedral, and the local symmetry to resemble *C*_3v_. This is consistent with the IR spectrum of **2** in *n*-octane at high temperatures. As mentioned above, two strong bands are observed at 2015 and 1938 cm^–1^ at 353 K. This spectral feature is characteristic of tetrahedral *C*_3v_-symmetrical tricarbonyl species; the former can be assigned as the symmetric A_1_ stretch, and the latter can be assigned as the asymmetric E stretch. These CO stretching vibrations are blue-shifted relative to those found in Holland's four coordinate Fe(i) dicarbonyl complex, (NacNac^*t*Bu^)Fe(CO)_2_ (1992 and 1908 cm^–1^),^8*a*^ but are significantly shifted to lower wavenumbers compared with those of previously reported five coordinate Fe(i)-tricarbonyl complexes.^6a,8a,8b^

The equilibrium constant (*K*_eq_) between **2** and **3** was determined by variable temperature NMR spectroscopy. The concentration of **3** in solution was estimated by the Evans method,^18^ and the thermodynamic parameters for the homolytic cleavage of the Fe–Fe bond in **2** were calculated to be Δ*H* = 18.8 ± 0.5 kJ mol^–1^ and Δ*S* = 46.1 ± 1.8 J mol^–1^ K^–1^, respectively. The thermodynamic parameters were also estimated based on the concentration of **2** (Δ*H* = 15.8 ± 0.2 kJ mol^–1^ and Δ*S* = 36.7 ± 0.4 J mol^–1^ K^–1^), which are roughly consistent with those obtained by the Evans method.¶
¶The thermodynamic parameters were calculated based on the concentration of **3**, which was estimated by the Evans method,^18^ in which the number of unpaired electrons at the iron center was assumed to be 1. Because it is difficult to quantitate the exact concentration of **2** and **3** simultaneously in solution, the thermodynamic parameters were alternatively calculated based on the concentration of **2**, as estimated from the relative ratio of the integral value in the ^1^H NMR spectra measured in C_6_D_6_ at various temperatures. Although the relative ratio of the integral values of sharp (diamagnetic) *vs.* broad (paramagnetic) peaks may include some experimental error, the obtained thermodynamic parameters were roughly consistent with those obtained by the Evans method. The details are given in ESI.†
 The large positive Δ*S* value is reasonable for the homolysis of Fe–Fe bond.

**Scheme 3:**
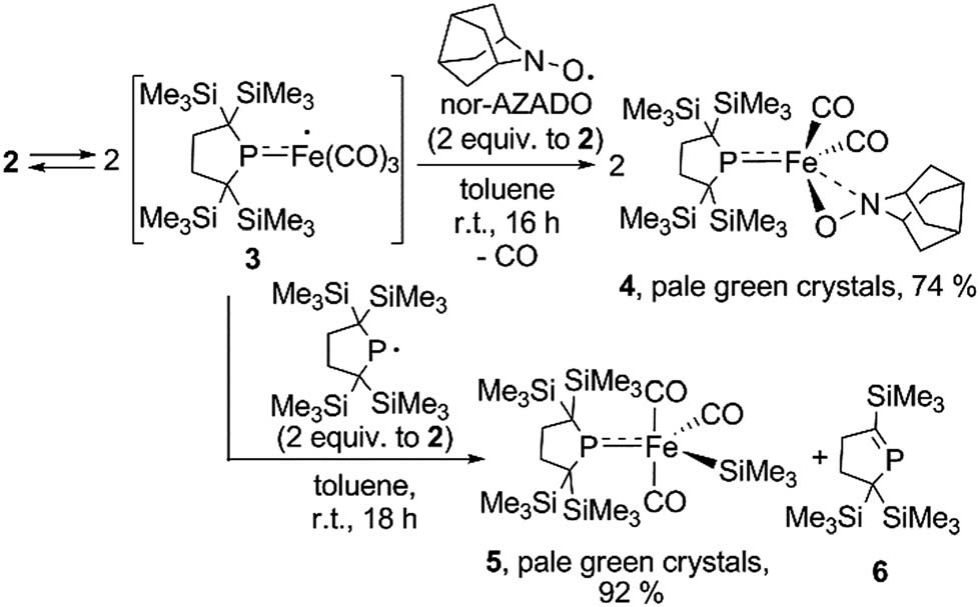
Reactions of *in situ* generated **3** with organic radicals.

**Fig. 7:**
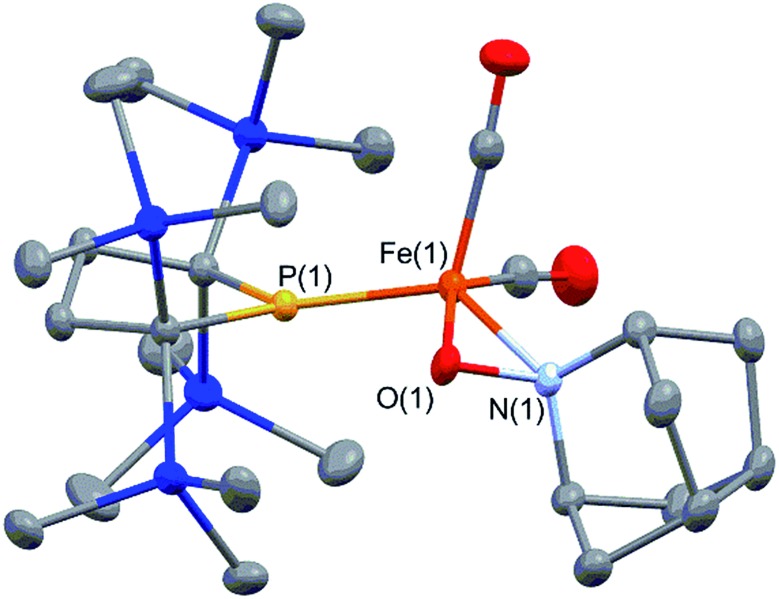
Molecular structure of **4** with 50% probability ellipsoids.

It is well known that metalloradicals can be captured by reaction with radical traps, such as stable organic radicals, metal-hydride species including HSnR_3_, and group 15 based cage compounds (*e.g.*, P_4_). As a recent representative example, Scheer *et al.* have described that the sterically hindered iron dimer [Cp^BIG^Fe(CO)_2_]_2_ (Cp^BIG^ = C_5_(4-*n*BuC_6_H_4_)_5_), readily dissociates in solution to afford a monomeric iron-centered radical, which can be easily trapped by reaction with P_4_ or As_4_.^19^ In manganese chemistry, Figueroa has synthesized an isolable mononuclear manganese-centered radical by introduction of sterically bulky isocyanide ligands, the reactivity of which was clearly indicated by its reaction with HSnR_3_ and some organic substrates.^6*k*^ Because **3** consists of only one bulky phosphido and three less hindered CO ligands, the iron center of **3** is expected to have enough room to react with certain organic substrates. Thus, we performed reactions of **3** with organic radicals. First, treatment of **3** (generated *in situ* from **2**) with 9-azanoradamantane *N*-oxyl (nor-AZADO) was examined. The reaction proceeds smoothly, even at room temperature, to afford diamagnetic five-coordinate mononuclear iron(ii) complex **4** in 74% isolated yield (Scheme 3).‖
‖One of the reviewers pointed out that dinuclear complex **2** may have the possibility of directly reacting with nor-AZADO to form product **4**. Because one broad peak assignable to **3** almost disappeared in ^1^H NMR spectrum of **2** at 253 K, we have performed the reaction of **2** with nor-AZADO in toluene-d_8_ at 253 K. We found that only *ca.* 8% of **2** was converted to product **4** after 25 days at 252 K. This result may indicate that *in situ* generated radical **3** can react with nor-AZADO, and that direct reaction of dinuclear complex with nor-AZADO can be ruled out. Identification of **4** was carried out by IR and NMR spectroscopy, and X-ray diffraction analysis. The signals due to the SiMe_3_ groups of **4** appear as two singlets at 0.36 and 0.47 ppm in the ^1^H NMR spectrum in C_6_D_6_. One phosphorus resonance is observed at 282.16 ppm in the ^31^P NMR spectrum. It is shifted to a higher field as compared to **1**, presumably due to the coordination of one σ-donating nitrogen instead of a π-accepting CO ligand. Two strong absorption bands derived from the Fe–CO moiety appear at 1958 and 1892 cm^–1^ in the IR spectrum of **4**. The ORTEP representation of **4** is depicted in Fig. 7. Two CO ligands are bound to the iron center, and the O–N bond of nor-AZADO is coordinated to the iron center in a κ^2^-fashion with a bond distance of 1.3851(14) Å. The sum of the three angles around P is 358.1°, indicating the contribution of π-bonding. Although 2,2,6,6-tetramethylpiperidine-1-oxyl (TEMPO) and its analogues are known to coordinate to the metal center in a κ^2^-(ON) coordination mode in the reaction with some transition metal complexes,^20^ to the best of our knowledge, complex **4** is the first example of a structurally characterized nor-AZADO complex having a κ^2^-(ON) moiety. The overall reaction can be explained as follows: radical recombination between *in situ*-generated iron-centered radical **3** and the oxygen-centered radical takes place to create the Fe–O bond, followed by the liberation of one CO ligand from the iron center concomitant with the formation of the Fe–N bond. In the course of this reaction, nor-AZADO formally undergoes one electron reduction to coordinate to the iron(ii) center.

Next, we performed the reaction between **3** and phosphinyl radical **1**, from which formation of five-coordinate iron-silyl complex **5** and phosphaalkene **6** in a 1 : 1 ratio is confirmed. The presence of π-donation in **5** was ascertained by ^31^P NMR, as one significantly downfield shifted singlet is observed at 461.4 ppm. The molecular structure of **5** was unequivocally determined by X-ray diffraction analysis (Fig. S27†). The relatively short Fe–P bond length (2.1009(7) Å) as well as the planar geometry around the P atom (the sum of the three angles around P was found to be 359.92°) indicate the contribution of π-donation to the Fe–P interaction. This reaction can also be considered the result of the radical behaviour of complex **3**; the iron-centered radical **3** abstracts one of four SiMe_3_ groups from **1** *via* homolytic substitution (S_H_2) to form a Fe–SiMe_3_ bond concomitant with the formation of the C=P bond to afford **6** (detailed reaction mechanism is shown in Scheme S1 in ESI†). Given the fact that diamagnetic closed shell organoiron(ii) complexes tend to adopt coordinatively saturated octahedral coordination geometry, the results described here could contribute to the development of new synthetic methodologies towards novel iron complexes.

**Scheme 4:**
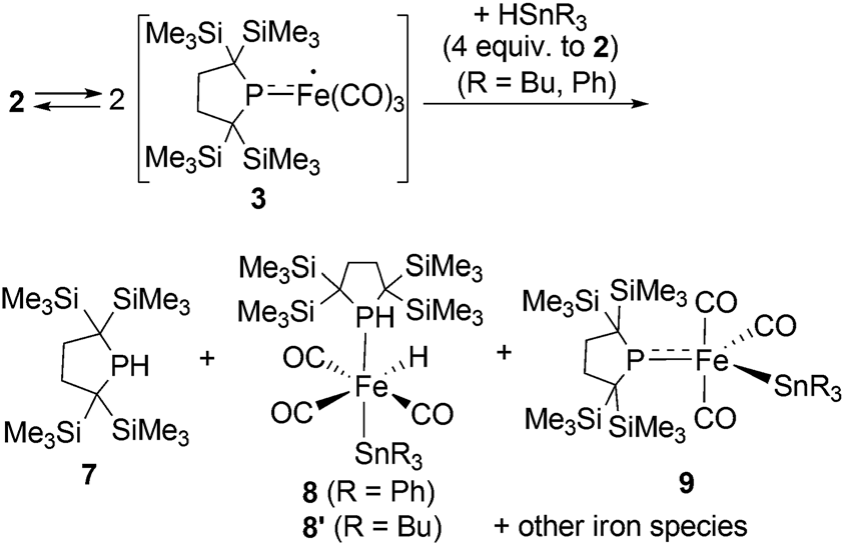
Reactions of *in situ* generated **3** with HSnR_3_ (R = Ph, Bu).

Reactions of *in situ* generated **3** with HSnR_3_ (R = Ph, Bu) were also performed. Reaction of **2** with HSnPh_3_ was monitored by ^1^H and ^31^P NMR spectra in C_6_D_6_ at room temperature. In the ^31^P NMR spectrum of the crude product, three peaks were observed at –33.6, 29.3 and 464.9 ppm, respectively, in an intensity ratio of 10 : 4 : 1. The peak at –33.6 ppm was assigned as free phosphine **7**,^10*a*^ whereas the other two peaks can be assigned as complexes **8** and **9**. Although **9** could not be obtained in pure form (see ESI† for detail), complex **8** can be isolated in 26% yield, and the molecular structure of **8** was determined by X-ray diffraction analysis (Fig. S28†). The relative ratio of **7**, **8** and **9** was determined from the ^1^H NMR spectrum of the crude product using an internal standard, and was estimated to be 60 : 37 : 3 based on iron. Formation of trace amount of Ph_3_Sn–SnPh_3_ (*ca.* 2%) was detected in the ^1^H NMR spectrum of the crude product.**
**Although powder formation was not observed after the reaction of **2** with HSnPh_3_, low solubility of Ph_3_Sn–SnPh_3_ toward C_6_D_6_ may hamper the accurate detection of the amount of Ph_3_Sn–SnPh_3_ formed in this reaction. These results strongly suggest that the reaction of **3** with HSnPh_3_ gave a complex mixture, and the major reaction pathway is the formation of free phosphine **7**. At the first stage of this reaction, iron-centered radical **3** would abstract a H atom from HSnPh_3_ to generate five coordinate “(phosphido)Fe(H)(CO)_3_” species. Because such Fe–H species is expected to be unstable, following disproportionation would afford **7** and iron species including **8** and **9**. This possible reaction sequence is shown in Scheme S2 in ESI.† In a similar manner, reaction of **2** with HSnBu_3_ was also performed. The ^31^P NMR spectrum of the crude product suggests the formation of **7** as the major product concomitant with the formation of **8′** which is analogous to **8**. The slightly broadened ^1^H NMR signals are presumably due to the partial formation of paramagnetic species and prevented further detailed analysis. Formation of **7** was also confirmed in the crude ^1^H NMR spectrum (Fig. S13†). Formation of Bu_3_Sn–SnBu_3_ was observed in *ca.* 10% yield in the GC-MS analysis, suggesting that the partial radical recombination took place after the abstraction of a H atom from the Sn center (Scheme 4).

The reactions shown above clearly indicate the radical nature of **3**. Next, we have performed the reaction of 3 (generated *in situ* from **2**) with 9,10-dihydroanthracene or 1,4-cyclohexadiene. However, no reaction took place below 333 K, and partial decomposition of **2** was confirmed by ^1^H and ^31^P NMR in the reaction at 353 K for 24 h. Considering the difference in the bond dissociation energy for the C–H bond of 1,4-cyclohexadiene (73 kcal mol^–1^)^21*a*^ and the Fe–H bond (58.5 ± 5 kcal mol^–1^; determined in the gas phase),^21*b*^ the results obtained here may be reasonable. Further research to elucidate the bond dissociation energy of Fe–H derived from complex **3** will be carried out in our laboratory.

## Conclusions

In conclusion, we succeeded in preparing coordinatively unsaturated dinuclear iron carbonyl complex **2** by the reaction of Fe_2_(CO)_9_ with phosphinyl radical **1**. The generation of four-coordinate iron-centered radical **3** was realized by the thermal homolysis of the unsupported Fe–Fe bond of **2**. Experimental analysis and theoretical calculation revealed that π-donation from the phosphido ligand to the iron center electronically stabilizes the four-coordinate iron-centered radical **3**. In both complex **2** and **3**, π-bonding electrons in the p-orbital of phosphorus lies on the HOMO and HOMO – 1 for **2** and βHOMO for **3**, which are effectively involved in π-donation from phosphorus to the iron center. The orbital interactions in **3_opt_** shown in Fig. S20† clearly suggest that the 3p_*y*_ orbital of phosphorus and the d_*yz*_ orbital of iron can effectively interact to create the π-bonding interaction. Complex **3** effectively reacted with organic radicals to afford diamagnetic five-coordinate organoiron(ii) species. These results may facilitate the development of new synthetic methodologies towards the design and construction of highly reactive metal-centered radicals. Efforts to develop novel fundamental and catalytic reactions realized by coordinatively unsaturated iron-centered radicals are now underway in our laboratory.
