# Supplementary material for: Persistent four-coordinate iron-centered radical stabilized by π-donation
Source: Chem Sci. 2015 Sep 25;7(1):191–8. doi: 10.1039/c5sc02601f (PMC5515065; doi:10.1039/c5sc02601f)
Supplement: Supplementary file 1 [file SC-007-C5SC02601F-s001.pdf]

Electronic Supplementary Material (ESI) for Chemical Science.
This journal is © The Royal Society of Chemistry 2015

Supporting information for

Persistent Four-Coordinate Iron Centered Radical Stabilized by π-Donation

Yusuke Sunada*, Shintaro Ishida, Fumiya Hirakawa, Yoshihito Shiota, Kazunari Yoshizawa, Shinji Kanegawa, Osamu Sato, Hideo Nagashima, and Takeaki Iwamoto*

Contents

1. General p. S-2
2. Synthesis of **2** p. S-2
3. Synthesis of **4** p. S-2
4. Synthesis of **5** p. S-3
5. Synthesis of **8** p. S-3
6. Reaction of **2** with $HSnBu_3$ p. S-4
7. Reaction of **2** with 9,10-dihydroanthracene p. S-4
8. Possible reaction mechanism for the formation of **5** and **6** via homolytic substitution (S_H2) p. S-5
9. Possible reaction pathway for the formation of **7**, **8** and **9** in the reaction of **2** with $HSnR_3$ (R = Ph, Bu) p. S-5
10. ^1H, ^{13}C, ^{29}Si, ^{31}P NMR spectra of **2** p. S-6
11. ^1H, ^{13}C, ^{29}Si, ^{31}P NMR spectra of **4** p. S-8
12. ^1H, ^{13}C, ^{29}Si, ^{31}P NMR spectra of **5** p. S-10
13. ^1H, ^{13}C, ^{29}Si, ^{31}P NMR spectra of **8** p. S-12
14. Variable temperature ^1H NMR spectra of solution of **2** in toluene-d_8 p. S-14
15. ESR spectra p. S-16
16. IR spectra p. S-17
17. UV-vis-NIR spectra p. S-20
18. Thermodynamic analysis p. S-22
19. NMR spectra of the crude product obtained by the reaction of **2** with **1**. p. S-24
20. NMR spectra of the mixture of **8** and **9** p. S-25
21. NMR spectra of the crude product obtained by the reaction of **2** with $HSnPh_3$ p. S-27
22. NMR spectra of the crude product obtained by the reaction of **2** with $HSnBu_3$ p. S-29
23. NMR spectra of the crude product obtained by the reaction of **2** with 9,10-dihydroanthracene p. S-31
24. Theoretical calculations p. S-32
25. X-ray diffraction analysis p. S-82
26. References p. S-109

General. Manipulation of air and moisture sensitive organometallic compounds was carried out under a dry argon atmosphere using standard Schlenk tube techniques associated with a high-vacuum line. Alternatively, the experiments were performed in a glove box filled with dry nitrogen. All solvents (pentane, *n*-octane, diethyl ether, hexamethyldisiloxane (HMDSO), mesitylene, C_6D_6, toluene-d_8) were distilled over Ph_2CO/Na prior to use. ^{1}H, ^{13}C, ^{29}Si, and ^{31}P NMR spectra were recorded on a JEOL Lambda 600 spectrometer at ambient temperature unless otherwise noted. ^{1}H, ^{13}C NMR chemical shifts (δ values) were given in ppm relative to the solvent signal (^{1}H, ^{13}C) or standard resonances (^{29}Si; external $SiMe_4$, ^{31}P; external H_3PO_4). Elemental analyses were performed by a Perkin Elmer 2400II/CHN analyzer. IR spectra were recorded on a JASCO FT/IR-550 spectrometer. UV-vis-NIR absorption spectra were recorded on a Shimadzu UV-3100PC UV-VIS-NIR scanning spectrophotometer. ESR spectra were recorded on a JEOL JES-FA 200 Electron Spin Resonance spectrometer. $Fe_2(CO)_9$[1], phosphinyl radical **1**[2] were synthesized by the method reported in the literature. Nor-AZADO was purchased from Wako Pure Chemical Industries and was used without further purification.

Synthesis of 2. In a glove box, $Fe_2(CO)_9$ (200 mg, 0.55 mmol) was suspended in pentane (5 mL), and phosphinyl radical **1** (207 mg, 0.55 mmol) was added to the suspension. The resulting mixture was stirred at room temperature for 16 h. The mixture gradually changed from a golden yellow suspension to a dark red solution. The solvent was evaporated and the residue was extracted with pentane (10 mL). A small amount of insoluble materials was removed by centrifugation, and the mother liquid was concentrated to ca. 5 mL under vacuum. This mixture was cooled to 238 K overnight, from which dark red crystals of **2** were obtained in 86 % (based on **1**) yield (244 mg). ^{1}H NMR (600 MHz, C_6D_6, r.t.) δ 0.38 (s, 72H, $SiMe_3$), 2.05 (br d, $J_{H\text{-}P}$ = 9.3 Hz, 8H, CH_2). ^{1}H NMR (toluene-d_8, r.t.) δ 0.35 (s, 72H, $SiMe_3$), 2.03 (br d, $J_{H\text{-}P}$ = 9.9 Hz, 8H, CH_2). ^{1}H NMR (600 MHz, toluene-d_8, 193 K) δ 0.31 (s, 18H, $SiMe_3$), 0.35 (s, 18H, $SiMe_3$), 0.37 (s, 18H, $SiMe_3$), 0.48 (s, 18H, $SiMe_3$), 1.69-1.87 (br, 8H, CH_2). ^{13}C{^{1}H} NMR (151 MHz, C_6D_6, r.t.) δ 3.15 (s, $SiMe_3$), 38.33 (s, CH_2), 54.33 (d, $J_{C\text{-}P}$ = 52 Hz, *C*($SiMe_3$)$_2$), 223.79 (br s, Fe-CO). ^{29}Si{^{1}H} NMR (119 MHz, C_6D_6, r.t.) δ 2.85 (d, $J_{Si\text{-}P}$ = 12.8 Hz). ^{31}P{^{1}H} NMR (243 MHz, C_6D_6, r.t.) δ 425.9 (s). IR (ATR) ν_{CO} = 1916, 1932, 1963, 2014 cm^{-1}. IR (in *n*-octane, 293 K) ν_{CO} = 1921, 1940, 1968, 2017 cm^{-1}. UV-vis-NIR (*n*-octane, 293 K) λ_{max}/nm (ε/M^{-1}cm^{-1}) = 380 (3.02×10^4), 502 (6.26×10^3), 720 (2.78×10^3). Anal. Calcd. for $C_{38}H_{80}O_6Si_8P_2Fe_2$: C, 44.25; H, 7.82. Found: C, 44.08; H, 7.76.

Synthesis of 4. In a glove box, complex **2** (20 mg, 0.019 mmol) was dissolved in toluene (0.5 mL), and nor-AZADO (5.4 mg, 0.0038 mmol) was added to the solution. The resulting mixture was allowed to stand at room temperature for 16 h. The color of the solution gradually changed from dark red to dark green. The solvent was evaporated and the residue was extracted with pentane (5 mL). A small amount of insoluble materials was removed by centrifugation, and the solvent was removed under vacuum. The remaining crude product was dissolved in pentane (3 mL), and the solution was cooled to 238 K overnight to afford **4** as pale green crystals in 74 % yield (18 mg). ^{1}H NMR (600

MHz, C_6D_6, r.t.) δ 0.36 (s, 18H, $SiMe_3$), 0.47 (s, 18H, $SiMe_3$), 1.14-1.17 (m, 2H, CH_2 of nor-AZADO), 1.25-1.28 (m, 2H, CH_2 of nor-AZADO), 2.00-2.03 (m, 1H, CH of nor-AZADO), 2.08 (d, $J_{H\text{-}P}$ = 9.9 Hz, 4H, CH_2), 2.11-2.19 (m, 5H, CH_2 and CH of nor-AZADO), 2.87 (s, 2H, NC*H* of nor-AZADO). $^{13}C\{^1H\}$ NMR (151 MHz, C_6D_6, r.t.) δ 2.46 (s, $SiMe_3$), 34.07, 35.37, 37.19 (d, $J_{C\text{-}P}$ = 79 Hz, $C(SiMe_3)_2$), 40.25, 43.27, 76.97 (s, *C*NH of nor-AZADO), 221.19 (d, $J_{C\text{-}P}$ = 20.9 Hz, Fe-CO). $^{29}Si\{^1H\}$ NMR (119 MHz, C_6D_6, r.t.) δ 1.38, 1.54 (s, $SiMe_3$). $^{31}P\{^1H\}$ NMR (243 MHz, C_6D_6, r.t.) δ 282.16 (s). IR (ATR) ν_{CO} = 1892, 1958 cm^{-1}. Anal. Calcd. for $C_{26}H_{52}O_3N_1Si_4P_1Fe_1$: C, 49.90; H, 8.37; N, 2.24. Found: C, 49.66; H, 8.14; N, 2.08.

Synthesis of 5. In a glove box, complex **2** (20 mg, 0.019 mmol) was dissolved in toluene (0.5 mL), and **1** (14.6 mg, 0.039 mmol) was added to the solution. The resulting mixture was allowed to stand at room temperature for 18 h. The color of the solution gradually changed from dark red to dark green. The solvent was evaporated and the residue was extracted with pentane (5 mL). A small amount of insoluble materials was removed by centrifugation, and the solvent was removed under vacuum. The remaining crude product was dissolved in hexamethyldisiloxane (5 mL), and the solution was cooled to 238 K overnight to afford **5** as pale green crystals in 92 % yield (21 mg). ^1H NMR (600 MHz, C_6D_6, r.t.) δ 0.29 (s, 36H, C(Si*Me*$_3$)$_2$), 0.81 (s, 9H, Fe-Si*Me*$_3$), 1.97 (br d, $J_{P\text{-}H}$ = 11.0 Hz, 4H, CH_2). $^{13}C\{^1H\}$ NMR (151 MHz, C_6D_6, r.t.) δ 2.69 (s, C(Si*Me*$_3$)$_2$), 8.15 (s, Fe-Si*Me*$_3$), 27.80 (s, CH_2), 55.75 (d, $J_{C\text{-}P}$ = 48.8 Hz, *C*($SiMe_3$)$_2$), 218.77 (d, $J_{C\text{-}P}$ = 9.3 Hz, Fe-CO). $^{29}Si\{^1H\}$ NMR (119 MHz, C_6D_6, r.t.) δ 3.74 (d, $J_{Si\text{-}P}$ = 11.0 Hz, *C*(*Si*Me$_3$)$_2$), 29.05 (d, $J_{Si\text{-}P}$ = 7.3 Hz, Fe-*Si*Me$_3$). $^{31}P\{^1H\}$ NMR (243 MHz, C_6D_6, r.t.) δ 461.4 (s). IR (ATR) ν_{CO} = 1900, 1987, 2031 cm^{-1}. Anal. Calcd. for $C_{22}H_{49}O_3Si_5P_1Fe_1$: C, 44.87; H, 8.39. Found: C, 44.64; H, 8.22.

Synthesis of 8. In a glove box, complex **2** (60 mg, 0.058 mmol) was dissolved in C_6D_6 (1.5 mL), and $HSnPh_3$ (81.7 mg, 0.232 mmol) and 1,3,5-teimethoxybenzene (internal standard; 9.8 mg, 0.058 mmol) was added to the solution. The resulting mixture was allowed to stand at room temperature for 16 h. The color of the solution gradually changed from dark red to brown. Complete consumption of **2** was confirmed by ^1H and ^{31}P NMR spectra, then the solvent was evaporated. The residue was extracted with diethyl ether (5 mL), and the solution was cooled to 238 K overnight to afford the brown crystals. ^1H, ^{13}C, and ^{31}P NMR spectra of obtained brown crystals in C_6D_6 revealed that the crystals consist of complexes **8** and **9** in a ratio of 3 : 1 (8 mg). The mother liquid was again cooled to 238 K, then brown crystals of **8** was obtained as a single product in 26 % yield (26 mg). Spectral data for **8**: ^1H NMR (600 MHz, C_6D_6, r.t.) δ -9.24 (d, 1H, $J_{H\text{-}P}$ = 38.8 Hz, Fe-H, with a satellite signal due to the coupling with Sn, $J_{H\text{-}Sn}$ = 118.7 Hz), 0.17 (s, 18H, $SiMe_3$), 0.28 (s, 18H, $SiMe_3$), 1.66-1.85 (m, 4H, CH_2), 5.68 (d, 2H, $J_{H\text{-}P}$ = 321.7 Hz, HP), 7.16-7.20 (m, 3H, C_6H_5), 7.23-7.30 (m, 6H, C_6H_5), 7.93-8.04 (m, 6H, C_6H_5). $^{13}C\{^1H\}$ NMR (151 MHz, C_6D_6, r.t.) δ 1.91 (s, $SiMe_3$), 3.39 (s, $SiMe_3$), 22.57 (d, $J_{H\text{-}P}$ = 10.1 Hz, *C*($SiMe_3$)$_2$), 37.0 (s, CH_2), 128.66 (s, *para* of C_6H_5), 128.73 (s, *ortho* of C_6H_5, with a satellite signal due to the coupling with Sn, $J_{H\text{-}Sn}$ = 47.7 Hz), 137.32 (s, *meta* of C_6H_5, with a satellite signal due to the coupling with Sn, $J_{H\text{-}Sn}$ = 37.6 Hz), 143.45 (s, *ipso* of C_6H_5, with a

satellite signal due to the coupling with Sn, J_{H-Sn} = 381.5 Hz), 213.11 (br s, Fe-CO). ^{29}Si{^{1}H} NMR (119 MHz, C_6D_6, r.t.) δ 5.39 (d, J_{Si-P} = 8.2 Hz), 8.20 (d, J_{Si-P} = 2.7 Hz). ^{31}P{^{1}H} NMR (243 MHz, C_6D_6, r.t.) δ 29.3 (s, with a satellite signal due to the coupling with Sn, J_{P-Sn} = 98.6 Hz). ^{31}P NMR (243 MHz, C_6D_6, r.t.) δ 29.3 (d, J_{P-H} = 321.7 Hz). IR (ATR) ν_{CO} or ν_{Fe-H} = 2024, 1953, 1909 cm^{-1} ($^{(}$vibronic mixing prevents definitive assignments$^{)}$. Anal. Calcd. for $C_{37}H_{57}O_3Si_4P_1Sn_1Fe_1$: C, 51.21; H, 6.62. Found: C, 50.98; H, 6.48. Spectral data for **9** (complex **9** was obtained as the mixture with **8**. The actual ^{1}H, ^{13}C and ^{31}P NMR charts were given in Figures 12-1, 12-2 and 12-3. Spectral data of **9** were tentatively assigned by comparison with those of isolated **8**.): ^{1}H NMR (600 MHz, C_6D_6, r.t.) δ 0.21 (s, 36H, $SiMe_3$), 1.95 (br d, J_{H-P} = 10.3 Hz, 4H, CH_2), 7.16-7.20 (m, 3H, C_6H_5), 7.23-7.28 (m, 6H, C_6H_5), 7.90-7.98 (m, 6H, C_6H_5). ^{13}C{^{1}H} NMR (151 MHz, C_6D_6, r.t.) δ 2.78 (s, $SiMe_3$), 22.68 (d, J_{H-P} = 14.4 Hz, *C*($SiMe_3)_2$), 38.09 (s, CH_2), 128.52, 128.61, 137.60, 144.27 (s, C_6H_5) (satellite signals due to the coupling with Sn should be observed along with these peaks, however, the intensity of these peaks were too weak to observe the satellite signals.), 217.79 (br s, Fe-CO). ^{31}P{^{1}H} NMR (243 MHz, C_6D_6, r.t.) δ 464.90 (s).

Reaction of 2 with $HSnBu_3$. In a glove box, complex **2** (20 mg, 0.019 mmol) was dissolved in C_6D_6 (0.5 mL), and $HSnBu_3$ (22.6 mg, 0.077 mmol) and 1,3,5-teimethoxybenzene (internal standard; 3.3 mg, 0.019 mmol) was added to the solution. The resulting mixture was allowed to stand at room temperature for 16 h. The color of the solution gradually changed from dark red to pale brown. The obtained crude product was analyzed by ^{1}H and ^{31}P NMR spectra. Actual charts were given in Figures 13-1 and 13-2.

Reaction of 2 with 9,10-dihydroanthracene. In a glove box, complex **2** (20 mg, 0.019 mmol) was dissolved in C_6D_6 (0.5 mL), and 9,10-dehydroanthracene (3.5 mg, 0.019 mmol) and 1,3,5-teimethoxybenzene (internal standard; 3.3 mg, 0.019 mmol) was added to the solution. The resulting mixture was allowed to stand at room temperature for 16 h. The ^{1}H and ^{31}P NMR spectra revealed that no reaction took place at this stage. Then, the mixture was allowed to stand at 333 K for 24 h, and no reaction took place which was confirmed by ^{1}H and ^{31}P NMR spectra. Then the mixture was allowed to stand at 353K for 24 h. The ^{1}H and ^{31}P NMR spectra revealed that partial decomposition including the formation of phosphaalkene **6** and free phosphine **7** occurred at this stage, however, formation of anthracene was not detected by ^{1}H NMR and GC-MS spectra. The actual NMR chart obtained after the reaction at 353K was attached in Figures 14-1 and 14-2.

In a similar manner, reaction of **2** (40 mg, 0.038 mmol) with 1,4-cyclohexadiene (3.1 mg, 0.038 mmol) was performed in the presence of anisole (4.1 mg, 0.038 mmol) as an internal standard. No reaction occurred at below 333K, and partial decomposition took place at 353K for 24 h without formation of benzene (confirmed by ^{1}H NMR and GC-MS).

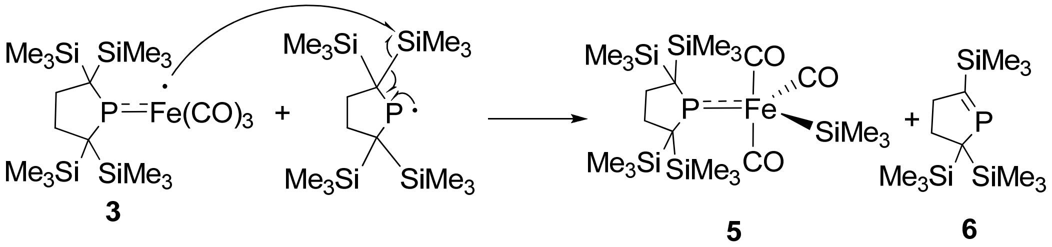

Scheme S1. Possible reaction mechanism for the formation of **5** and **6** via homolytic substitution (SH2).

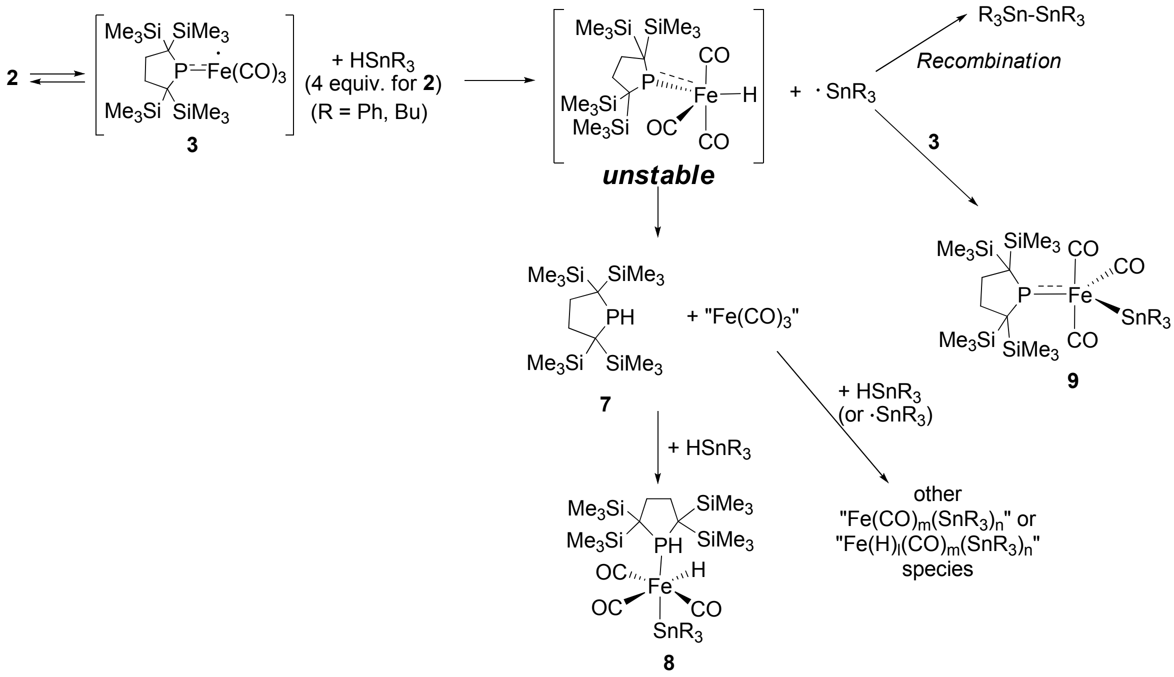

Scheme S2. Possible reaction pathway for the formation of **7**, **8** and **9** in the reaction of **2** with $HSnR_3$ (R = Ph, Bu).

<Note>: In the ^{1}H NMR spectrum of the crude product obtained by the reaction of **3** (*in situ* generated from **2**) with $HSnPh_3$, some unidentified signals were observed in the 7-8 ppm region in the ^{1}H NMR spectrum (see Figure S12-1), suggesting that iron species consisting of carbonyl and $SnPh_3$ ligands {such as "$Fe(CO)_m(SnPh_3)_n$ (m+n = 5 or 6)" or "$Fe(H)_l(CO)_m(SnPh_3)_n$ (l+m+n = 5 or 6)" shown in Scheme S2} may be generated in the course of this reaction.

Figure S1-1. ^{1}H NMR spectrum of solution of **2** in C_6D_6 at 293K.

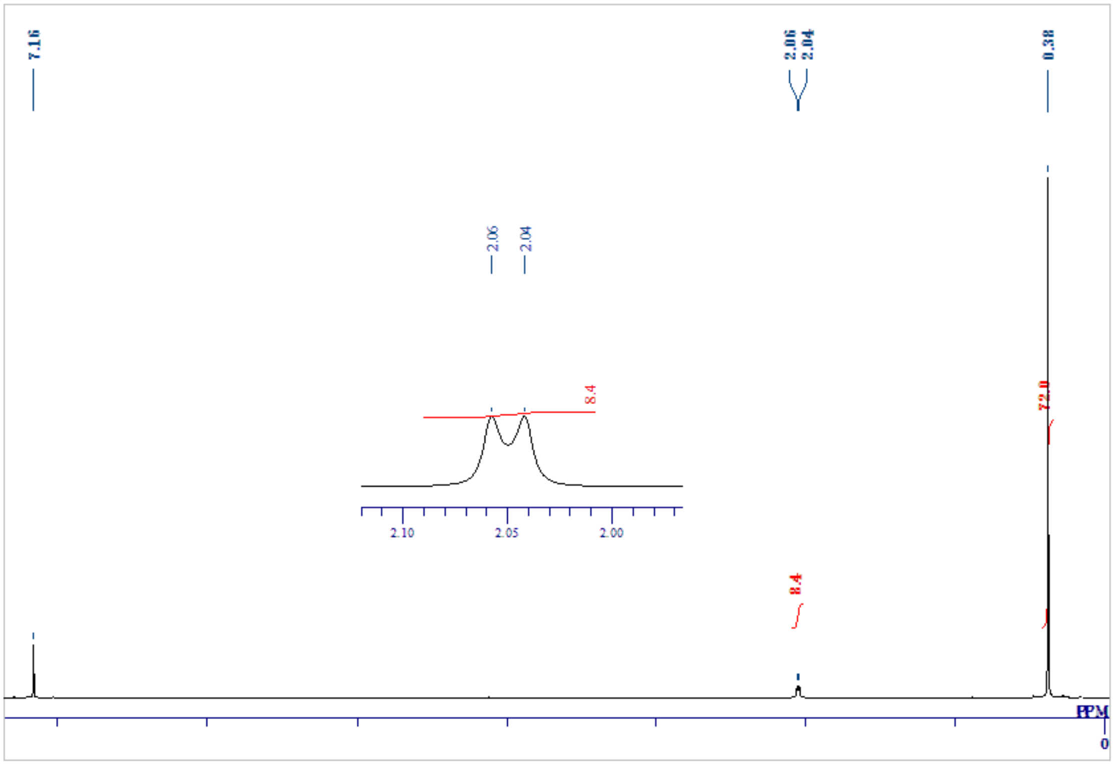

Figure S1-2. ^{13}C{^{1}H} NMR spectrum of solution of **2** in C_6D_6 at 293K.

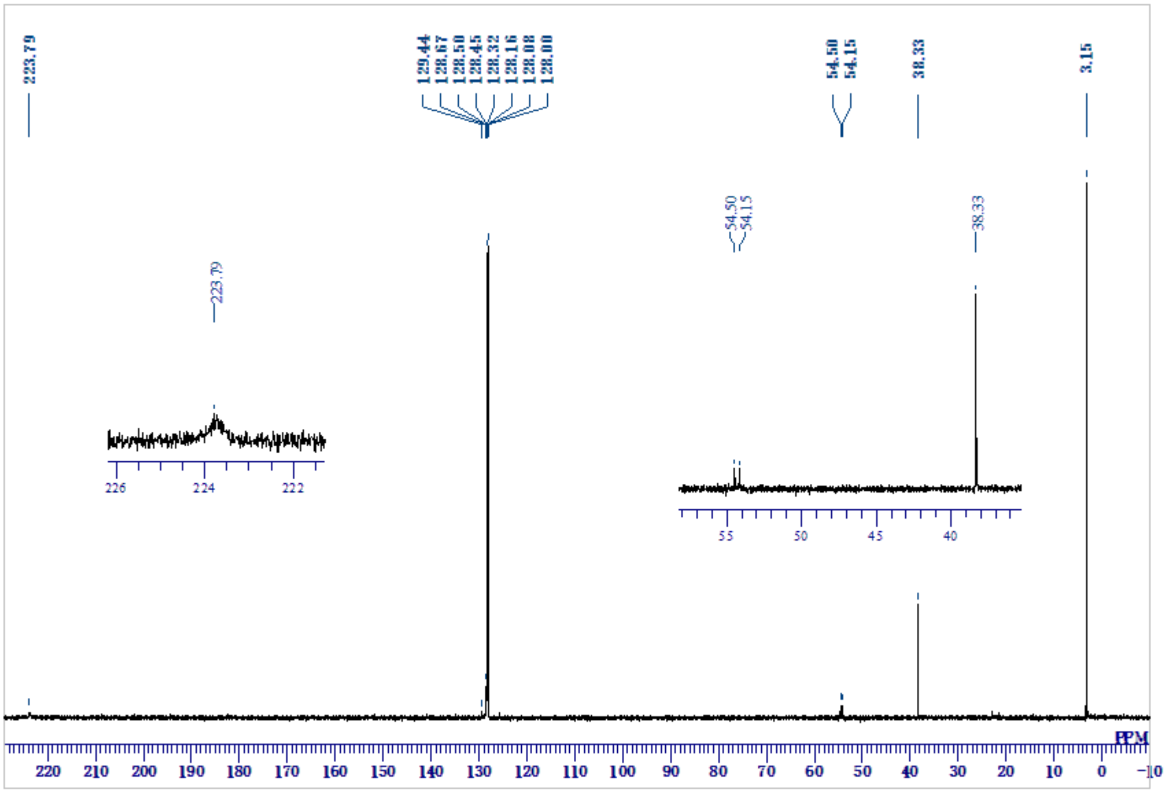

Figure S1-3. $^{29}Si\{^1H\}$ NMR spectrum of solution of **2** in C_6D_6 at 293K.

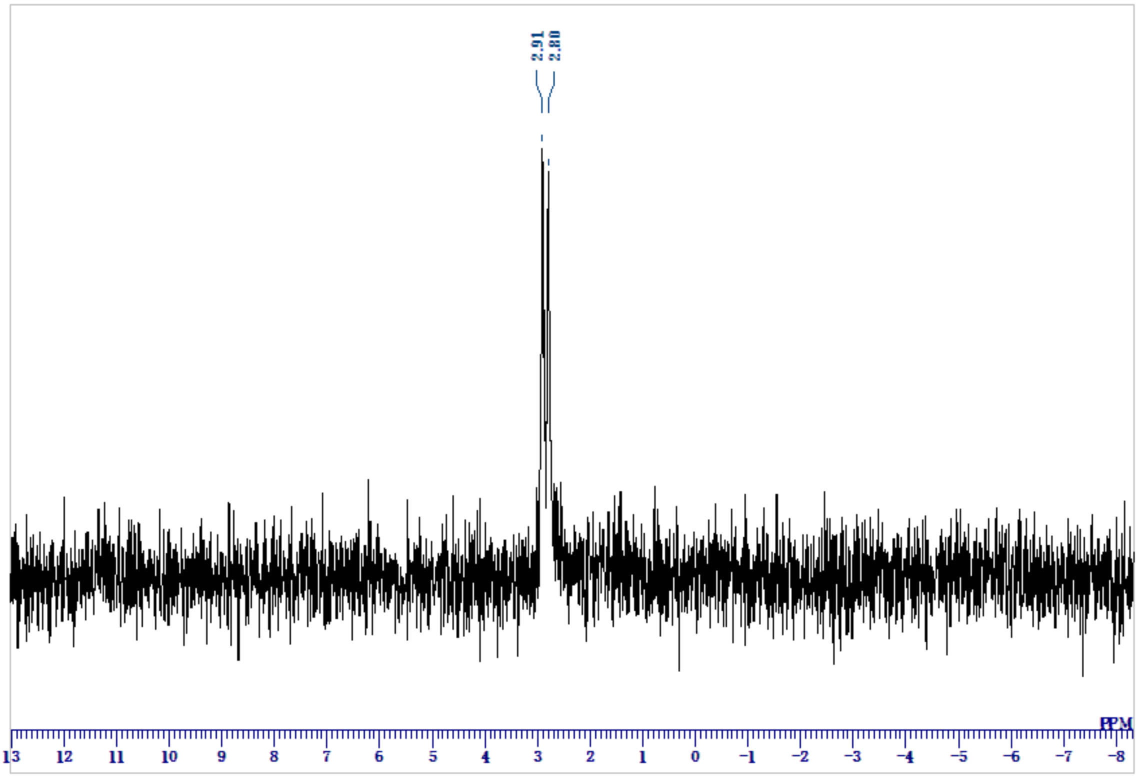

Figure S1-4. $^{31}P\{^1H\}$ NMR spectrum of solution of **2** in C_6D_6 at 293K.

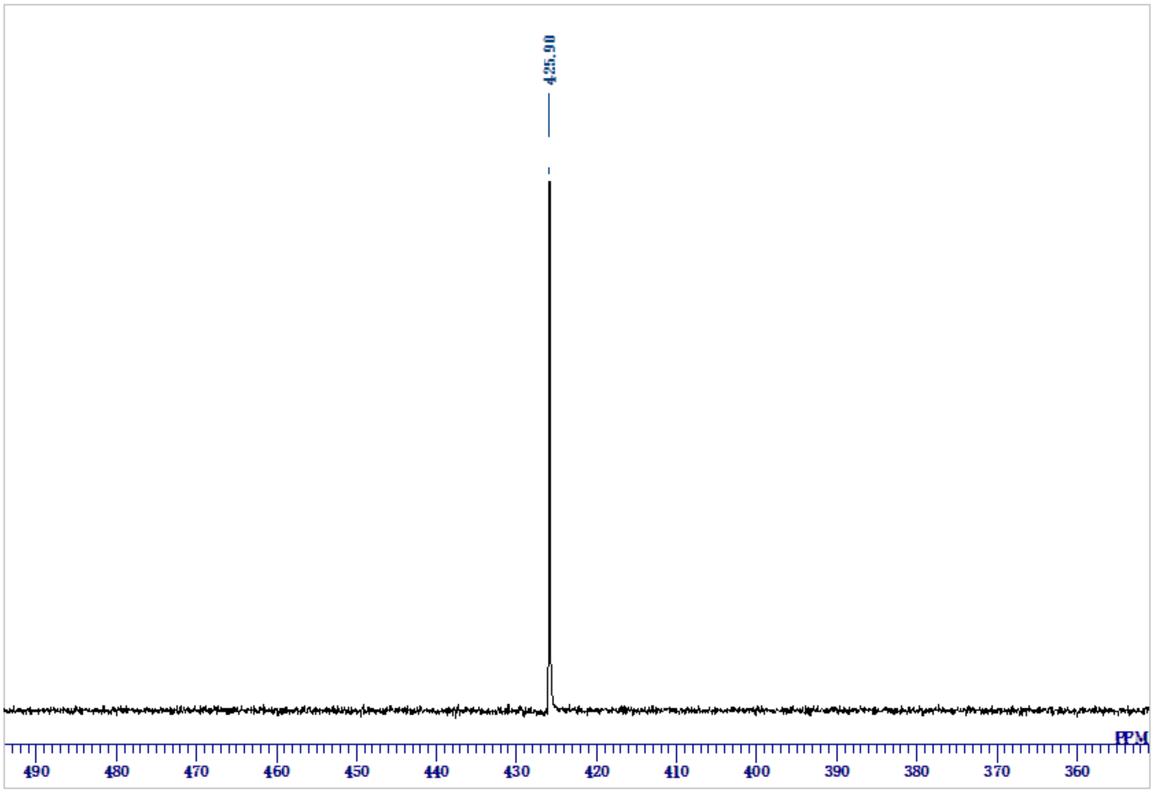

Figure S2-1. ^{1}H NMR spectrum of **4** in C_6D_6 at 293K.

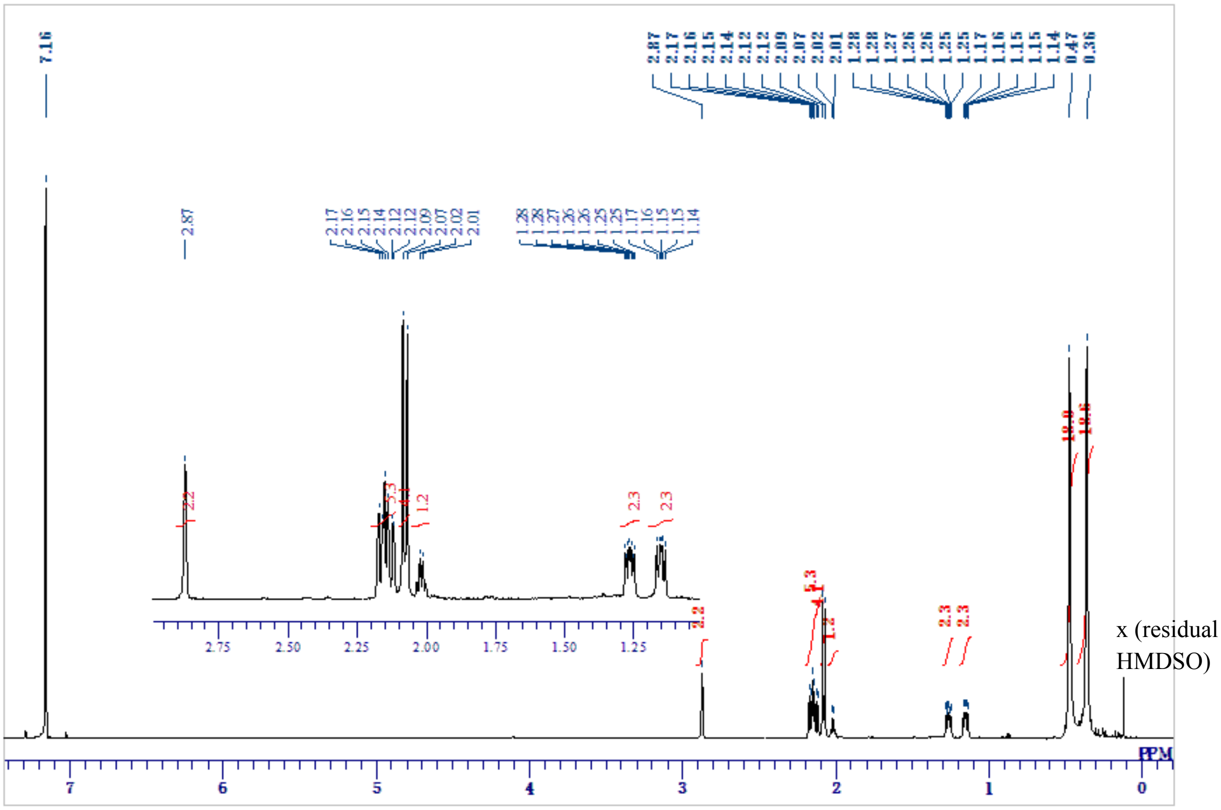

Figure S2-2. ^{13}C{^{1}H} NMR spectrum of **4** in C_6D_6 at 293K.

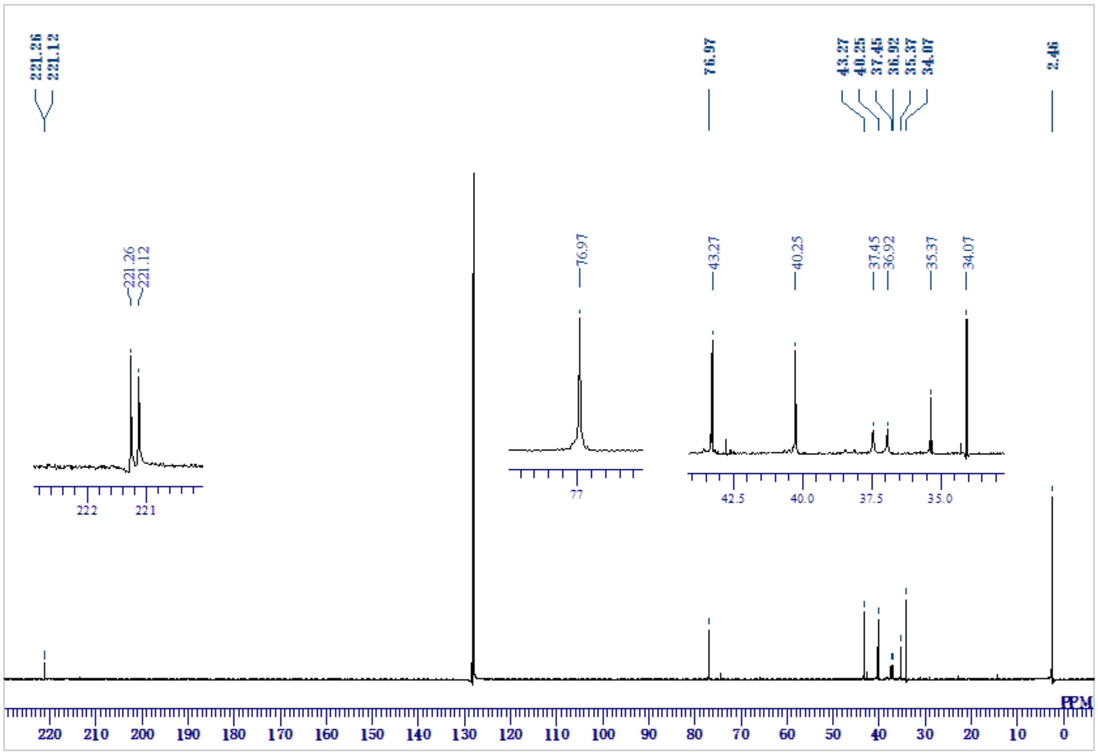

Figure S2-3. $^{29}Si\{^1H\}$ NMR spectrum of **4** in C_6D_6 at 293K.

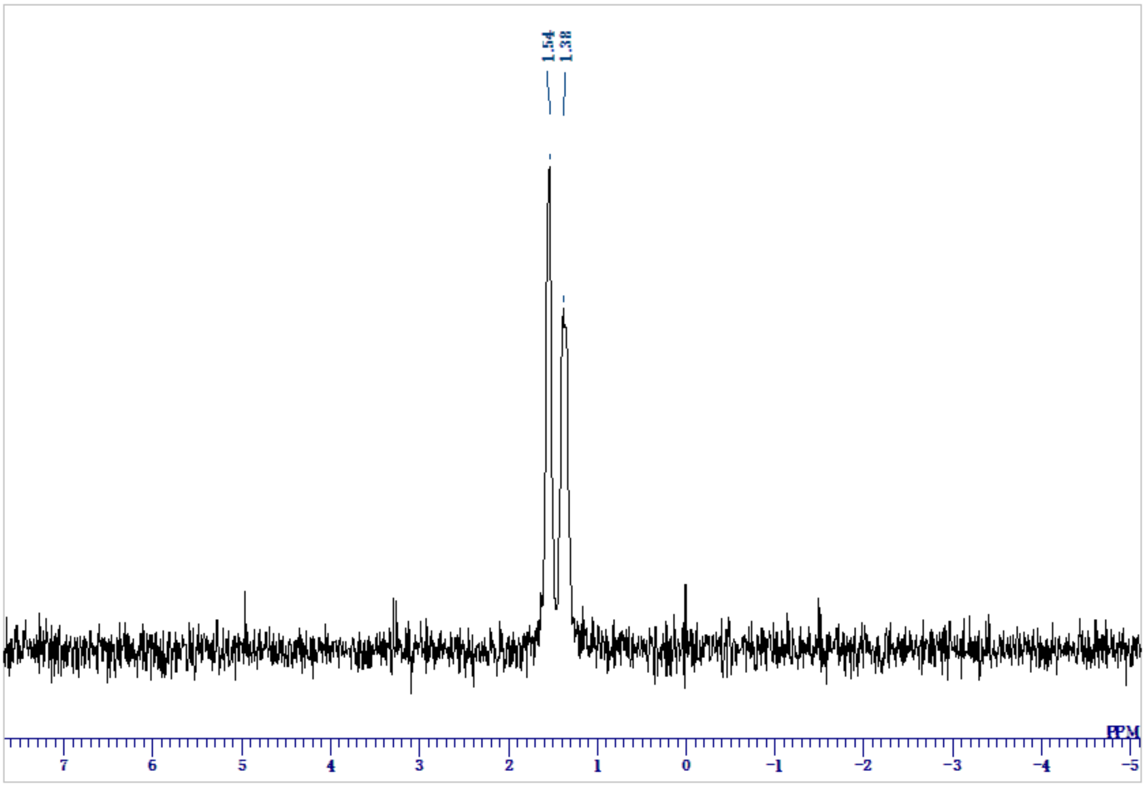

Figure S2-4. $^{31}P\{^1H\}$ NMR spectrum of **4** in C_6D_6 at 293K.

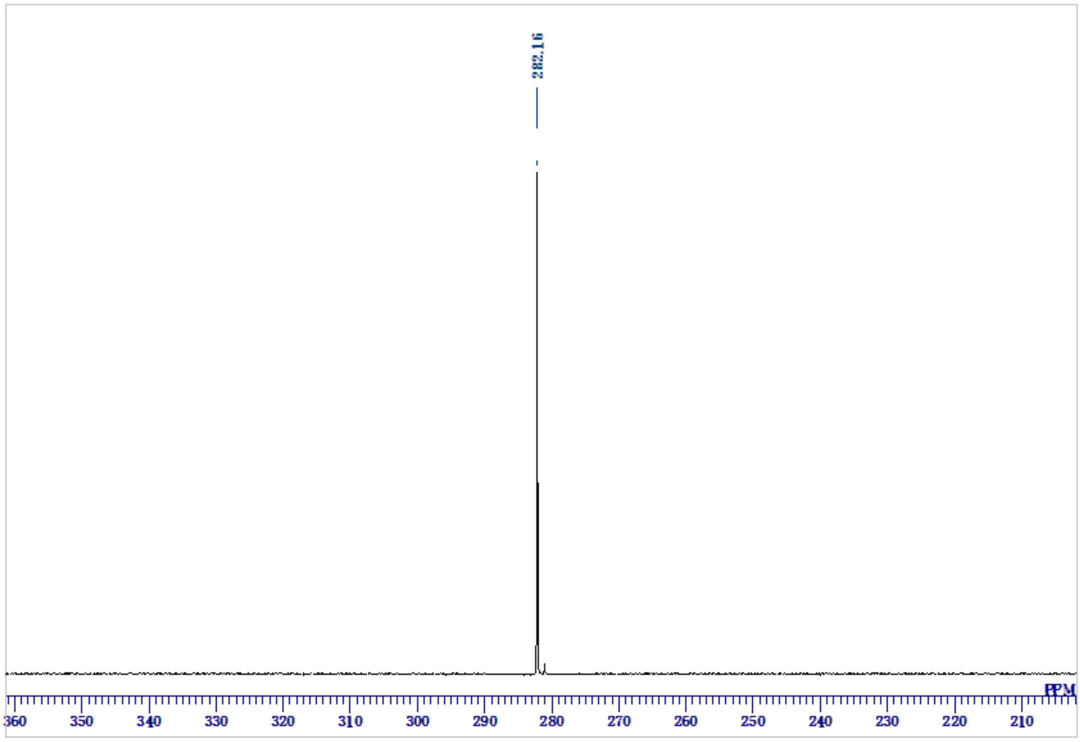

Figure S3-1. ^{1}H NMR spectrum of **5** in C_6D_6 at 293K.

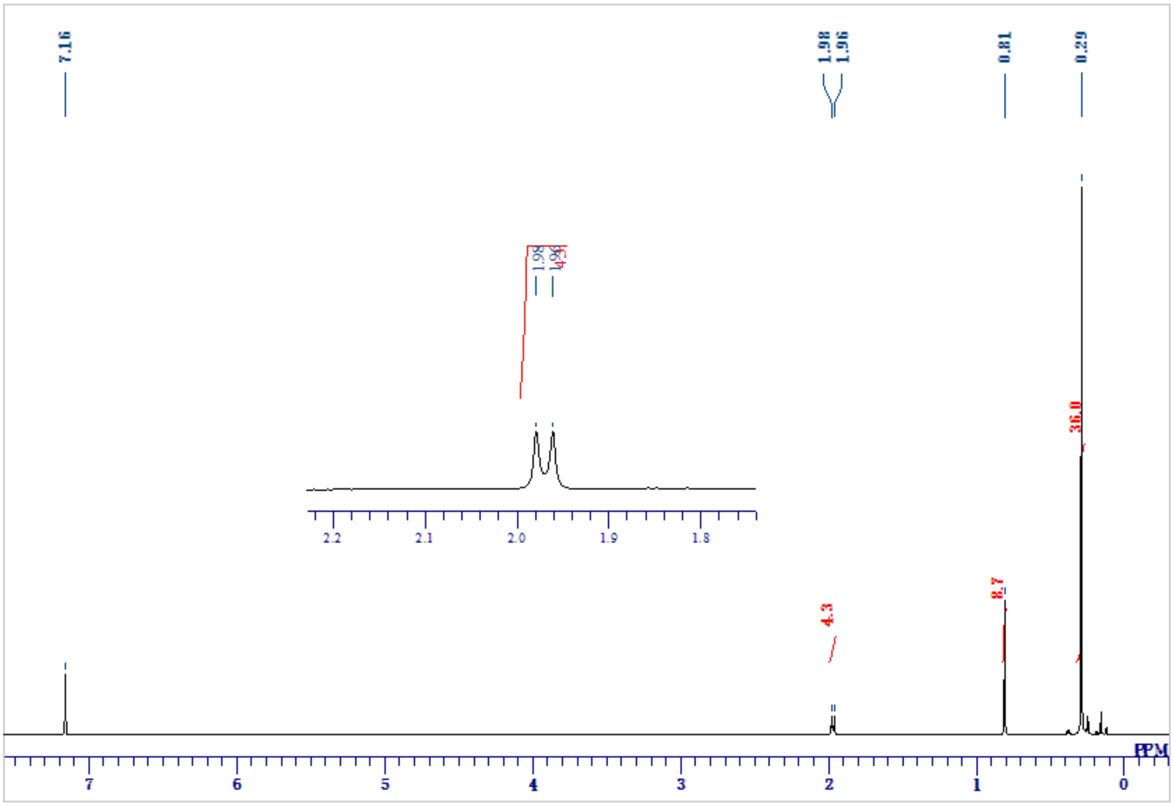

Figure S3-2. $^{13}C\{^{1}H\}$ NMR spectrum of **5** in C_6D_6 at 293K.

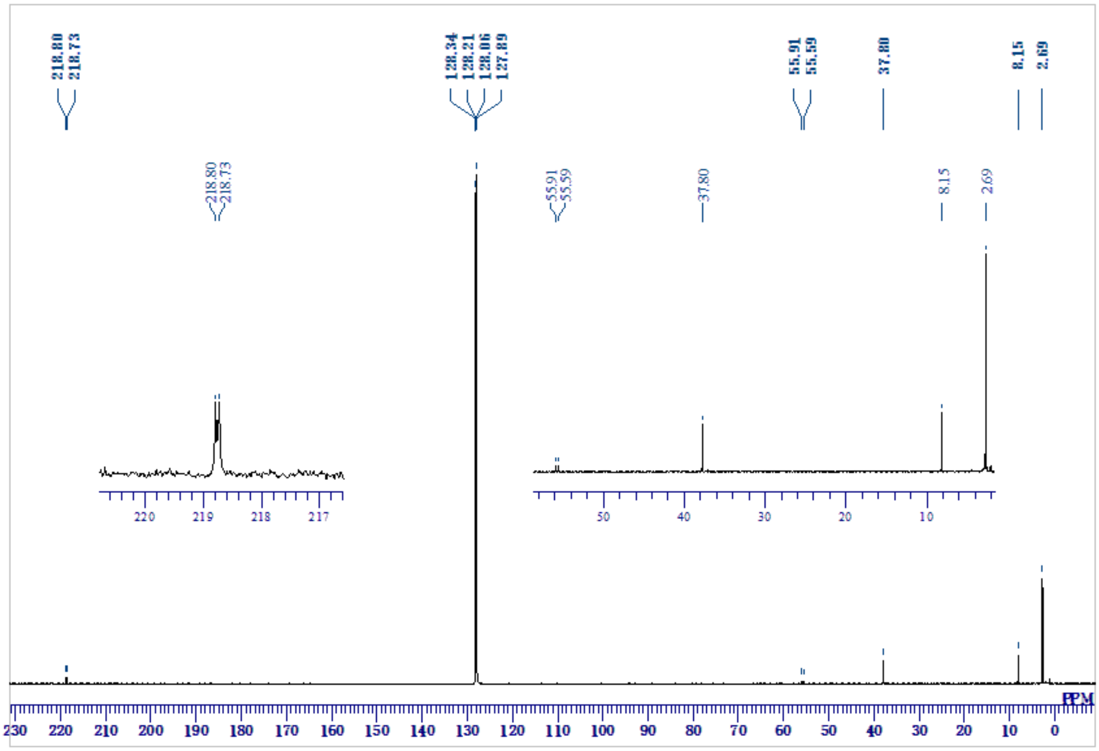

Figure S3-3. $^{29}Si\{^1H\}$ NMR spectrum of **5** in C_6D_6 at 293K.

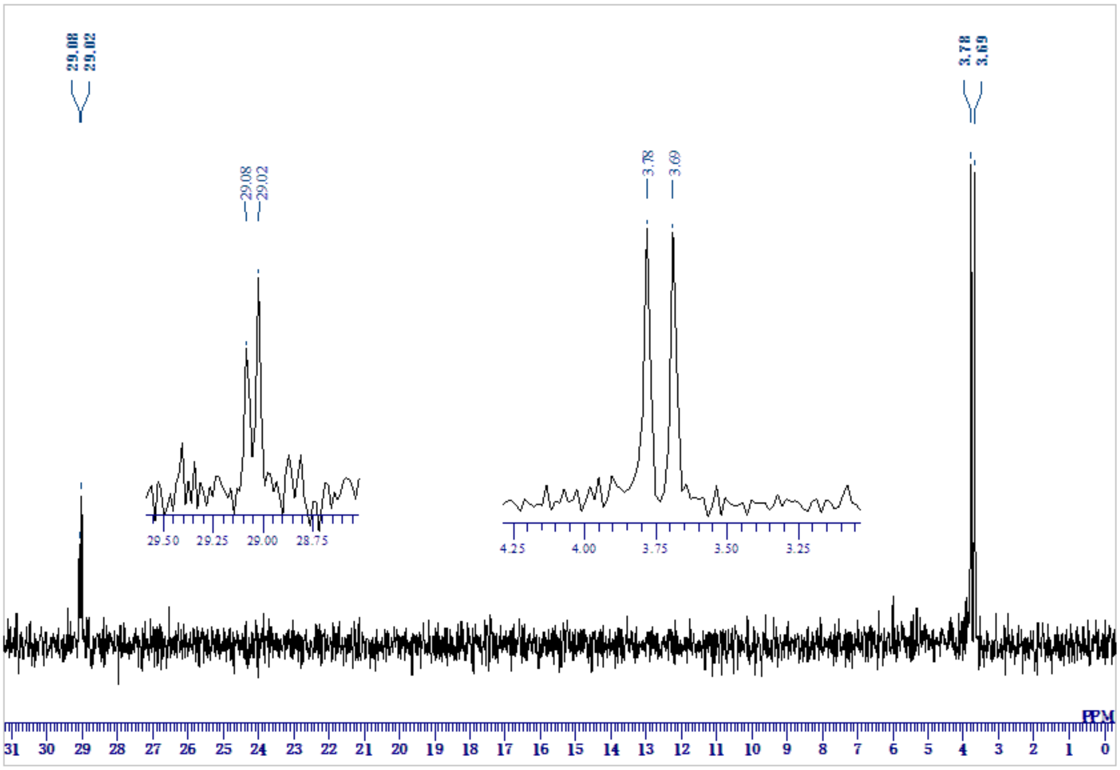

Figure S3-4. $^{31}P\{^1H\}$ NMR spectrum of **5** in C_6D_6 at 293K.

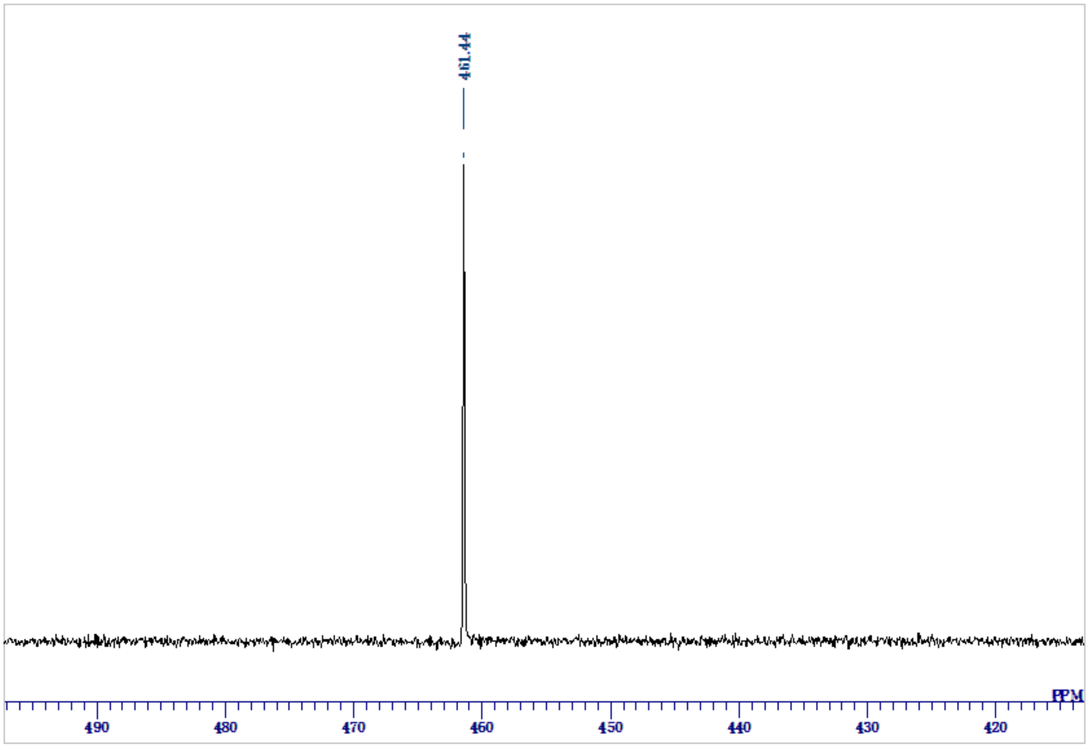

Figure S4-1. ^{1}H NMR spectrum of **8** in C_6D_6 at 293K.

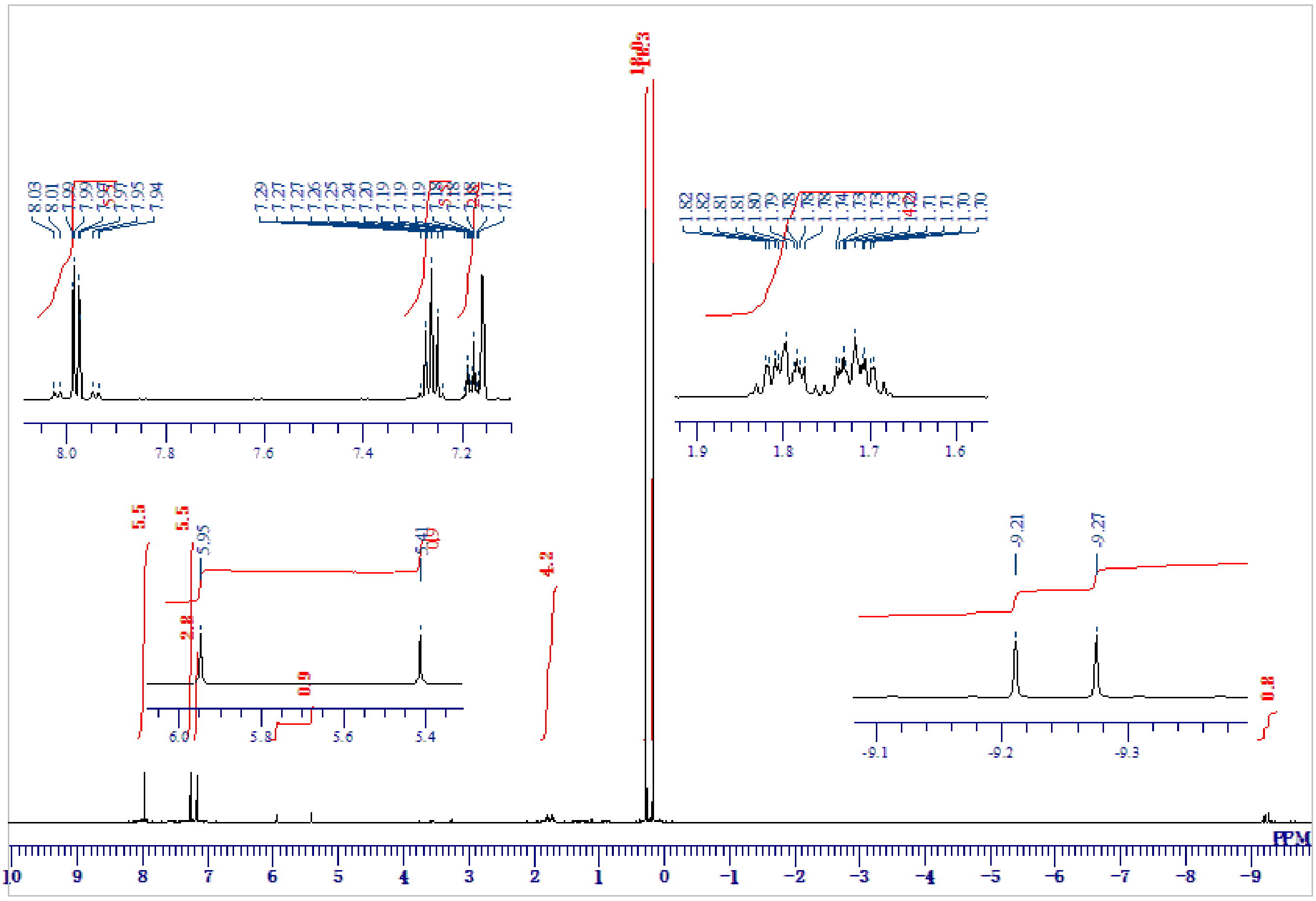

Figure S4-2. $^{13}C\{^{1}H\}$ NMR spectrum of **8** in C_6D_6 at 293K.

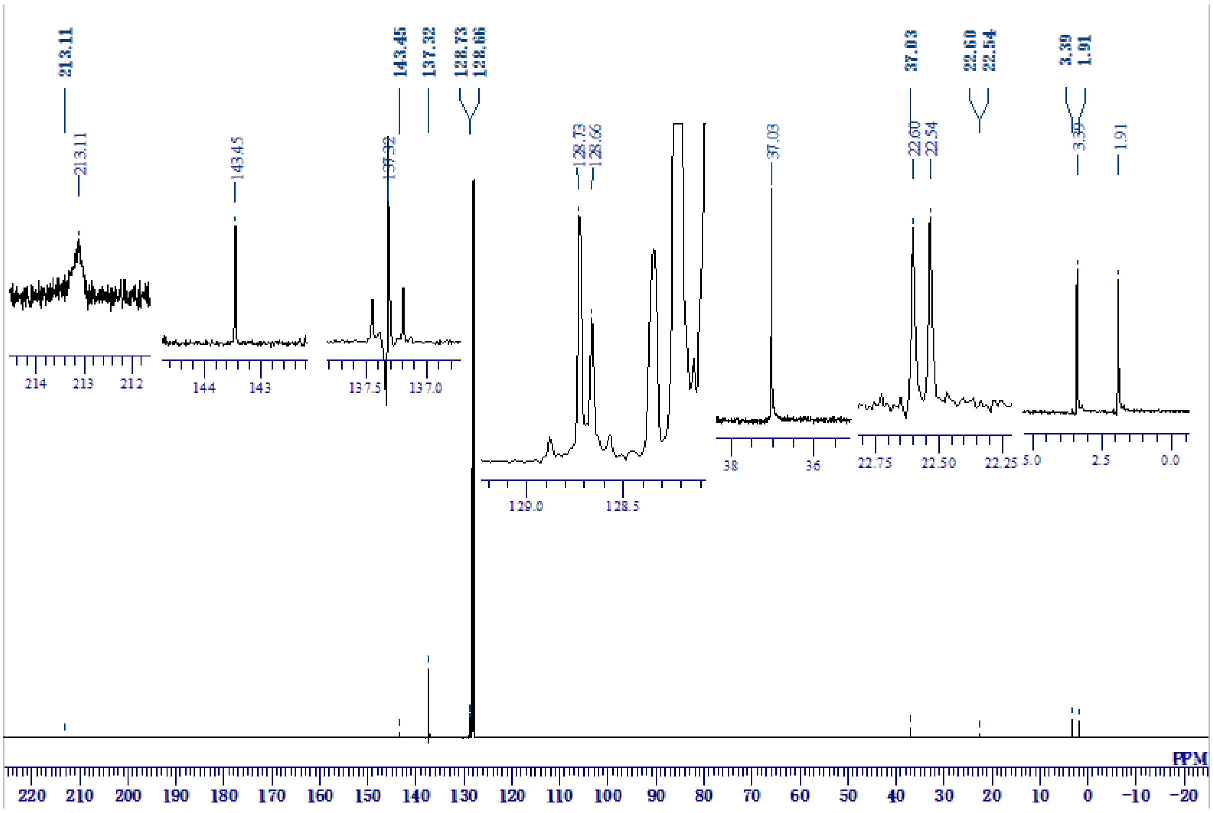

Figure S4-3. ^{29}Si{^{1}H} NMR spectrum of **8** in C_6D_6 at 293K.

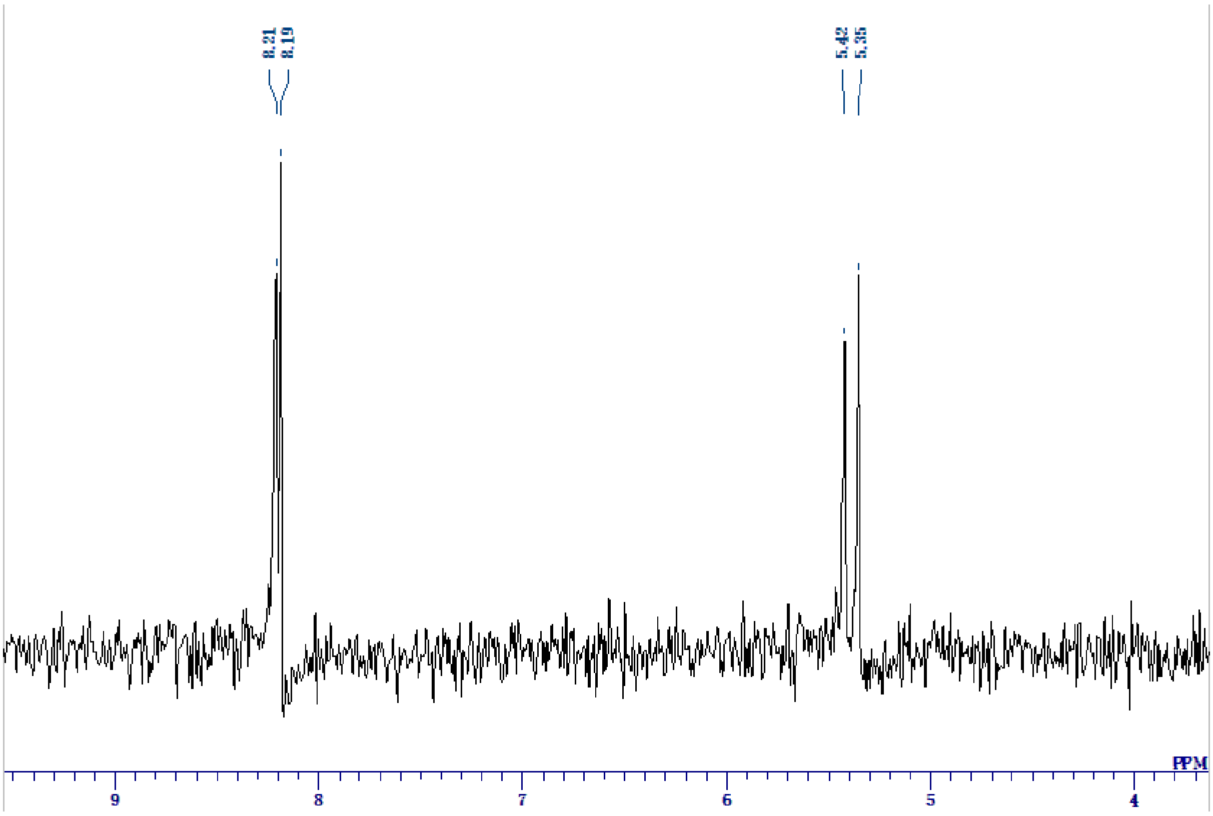

Figure S4-4. ^{31}P{^{1}H} NMR spectrum of **8** in C_6D_6 at 293K.

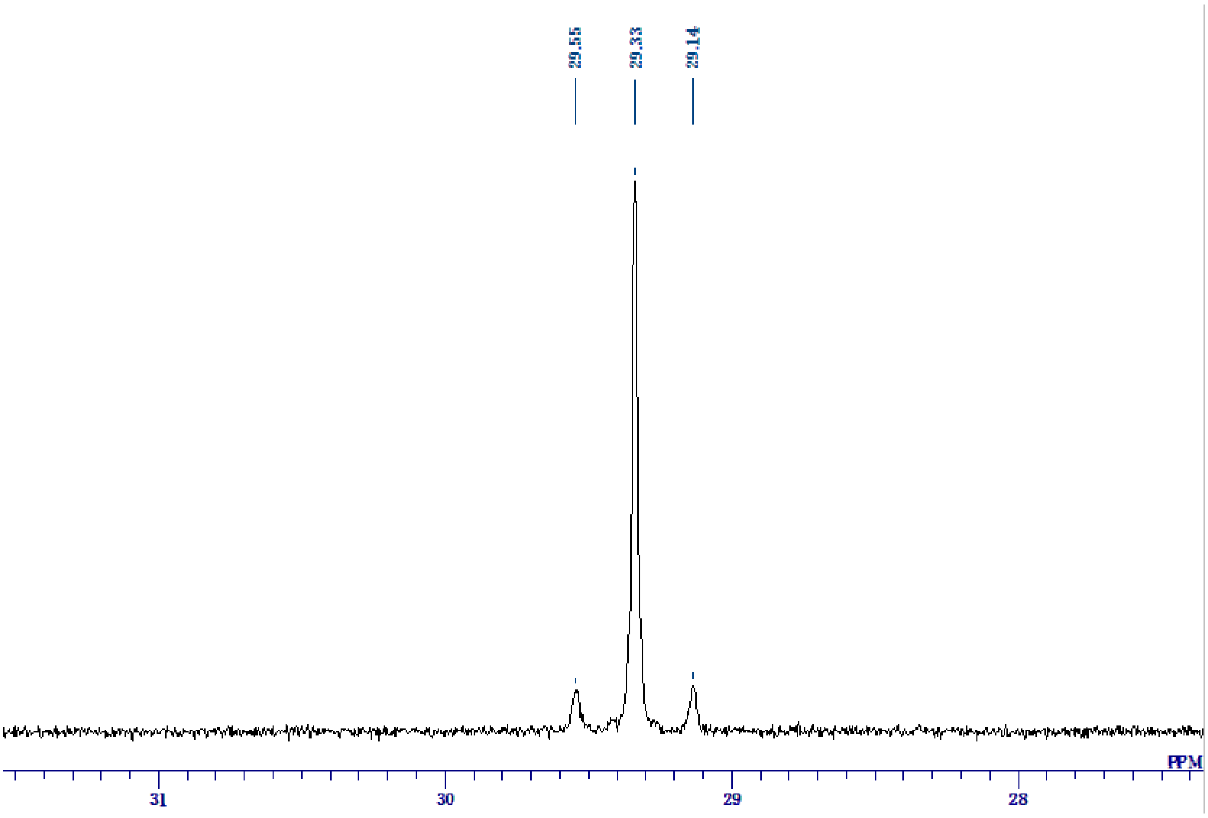

Figure S5-1. ^{1}H NMR spectra of solution of **2** in toluene-d_8 at various temperature (193 K~353 K).

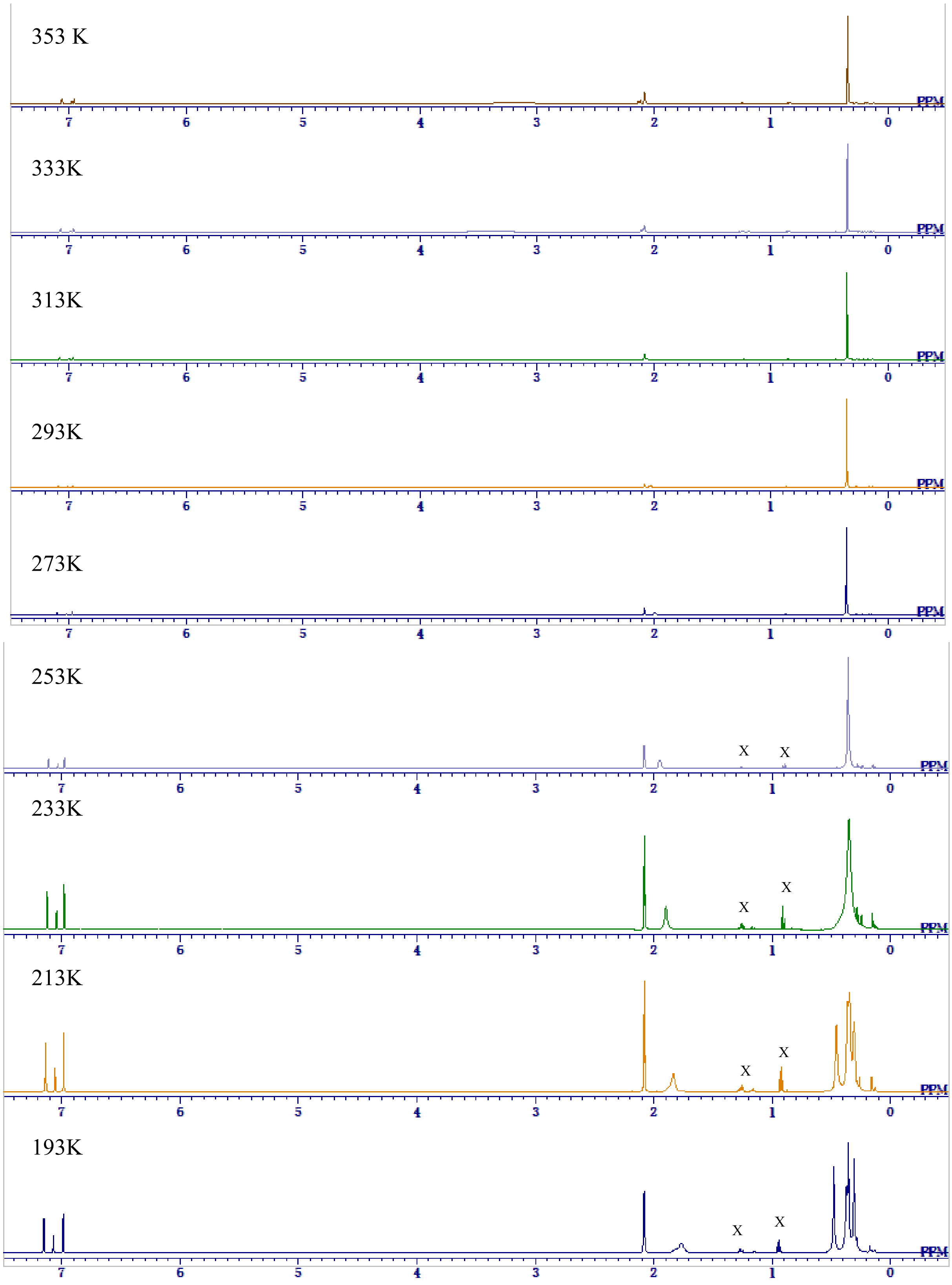

^{1}H NMR (600 MHz, in toluene-d_8)
(x = residual pentane)

Figure S5-2. Enlarged view of ^{1}H NMR spectra of solution of **2** in toluene-d_8 at various temperature (273 K~353 K). The solid-circles and open-circles indicate signals of C_7D_7H and –CH_2- moiety of complex **2**.

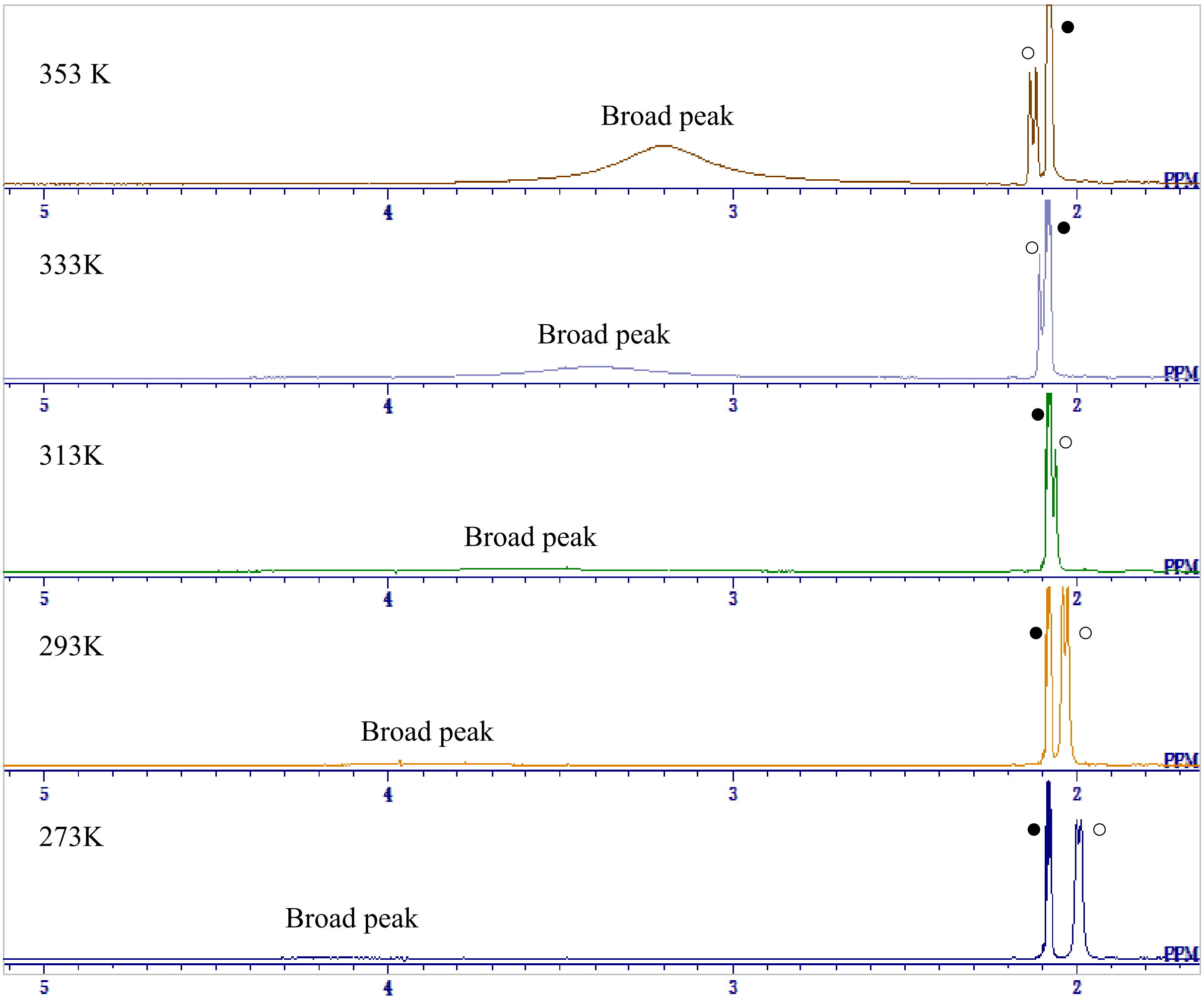

ESR spectra

A toluene solution of **2** (ca. 0.12 mM) was prepared by dissolving **2** (1.4 mg, 1.36 x 10^{-3} mmol) in toluene (11 mL); the ESR spectrum was immediately recorded at 293 K. Measurements at 313 K and 333 K were carried out subsequently. The sample was allowed to stand in the instrument for 30 min at each temperature prior to the measurement.

Figure S6-1. ESR spectra of solution of **2** in toluene.

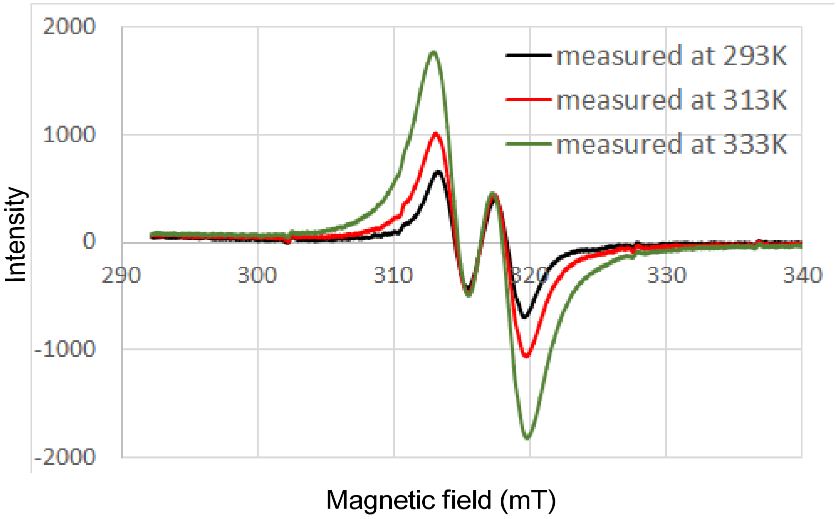

ESR spectrum of a flash-frozen toluene solution

A toluene solution of **2** (ca. 1.2 mM) was heated to 353 K, then this solution was flash-frozen. Then, the ESR spectrum was recorded at 77 K.

Figure S6-2. ESR spectrum of flash-frozen toluene solution measured at 77K.

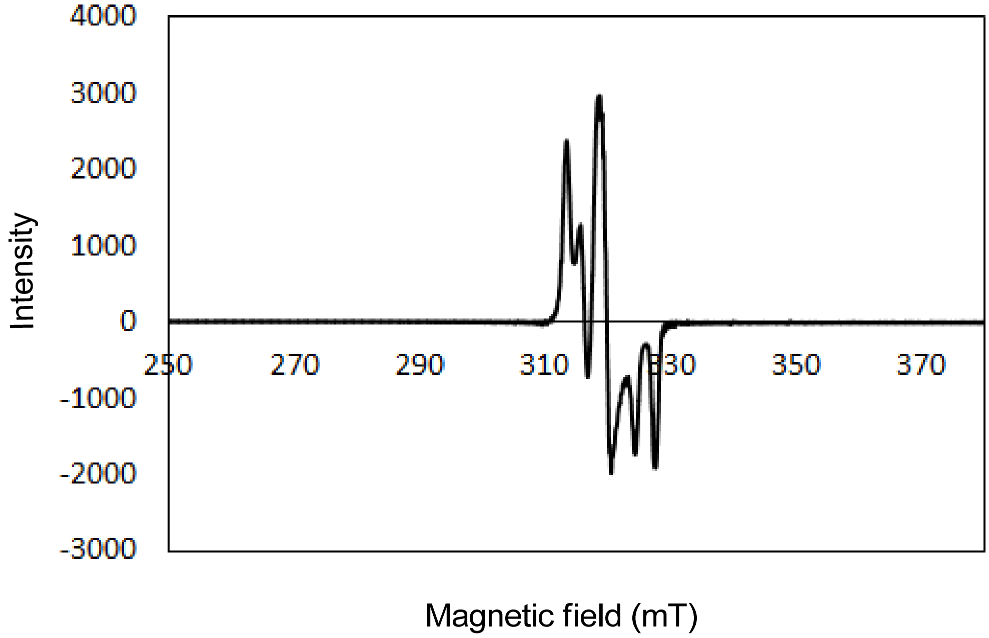

Figure S7-1. ATR-IR spectrum of **2** in the solid state.

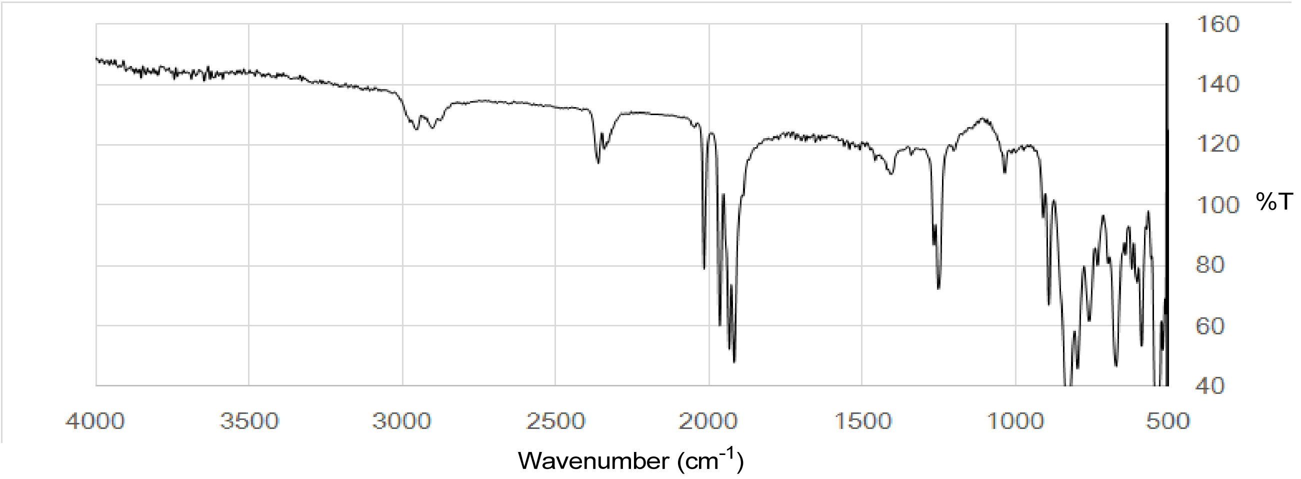

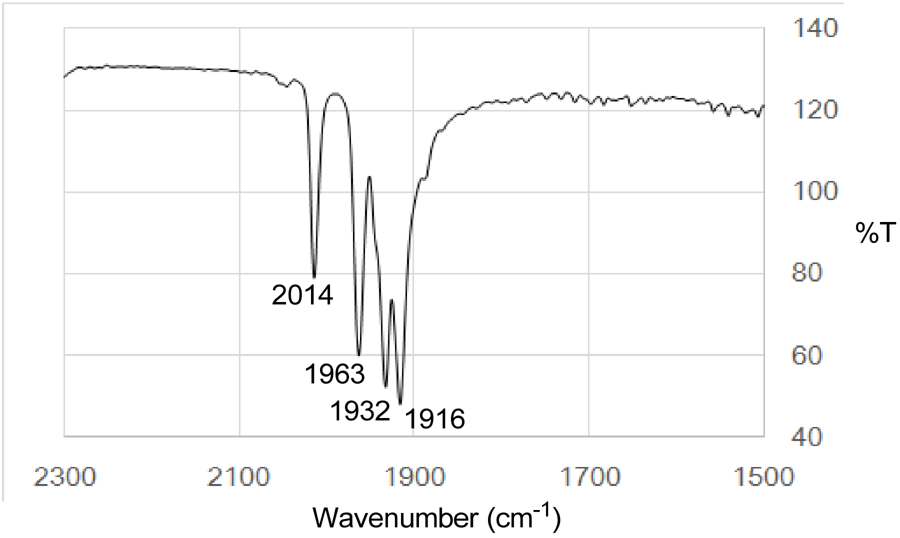

IR spectra in *n*-octane

An *n*-octane solution of **2** (ca. 0.12 mM) was prepared by dissolving **2** (1.3 mg, 1.24 x 10^{-3} mmol) in *n*-octane (10 mL) at room temperature; the IR spectrum was recorded immediately at 293 K. Next, the solution was allowed to stand for 10 min at 353 K, then the IR spectrum was measured again.

Figure S7-2-1. IR spectra of solution of **2** in *n*-octane at 293 K and 353 K.

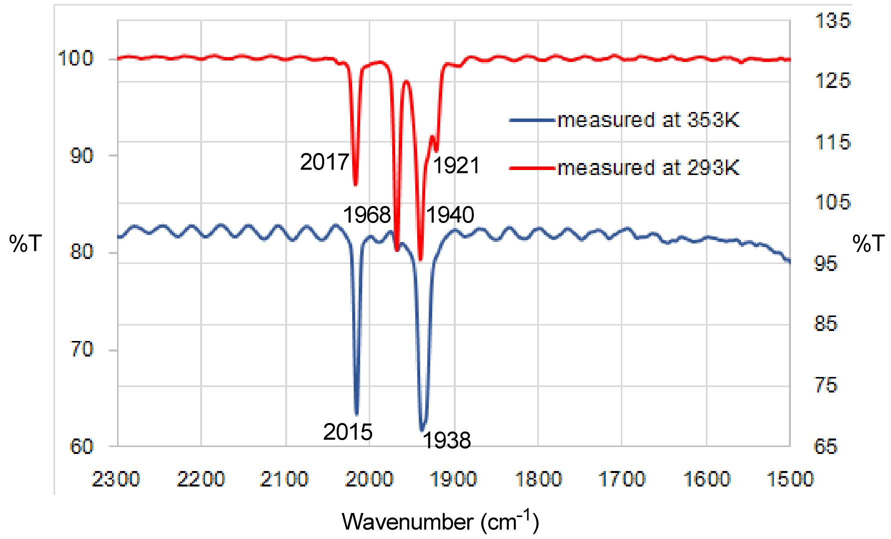

The time-course of IR spectra

An *n*-octane solution of **2** (ca. 0.12 mM) was prepared by dissolving **2** (1.3 mg, 1.24 x 10^{-3} mmol) in *n*-octane (10 mL). The IR spectrum was recorded at 353 K, then the temperature of the instrument was set to 293 K. The IR spectra were obtained periodically over 2 h.

Figure S7-2-2. The time-course of IR spectra of solution of **2** in *n*-octane after heating.

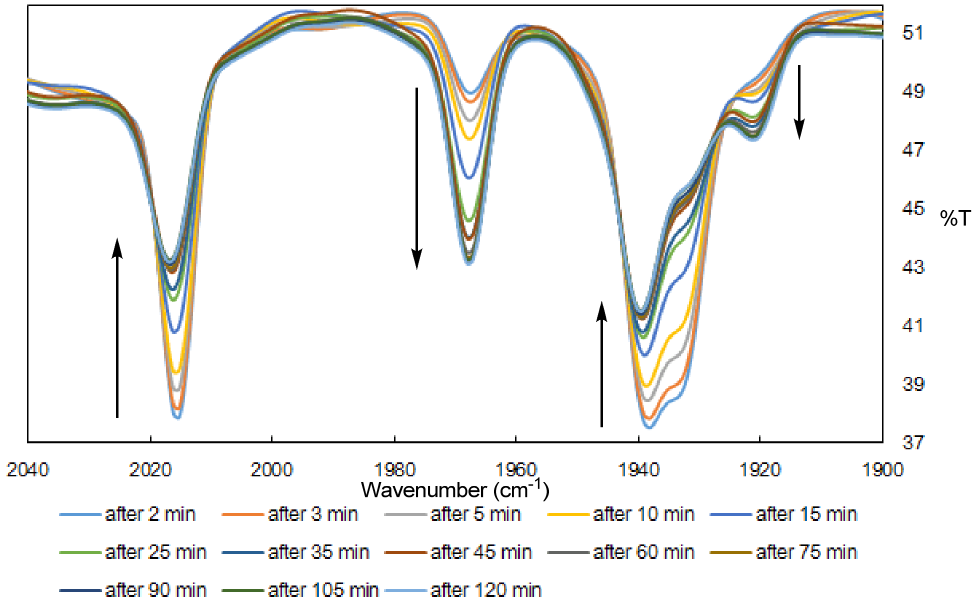

Figure S7-3. ATR-IR spectrum of **4** in the solid state.

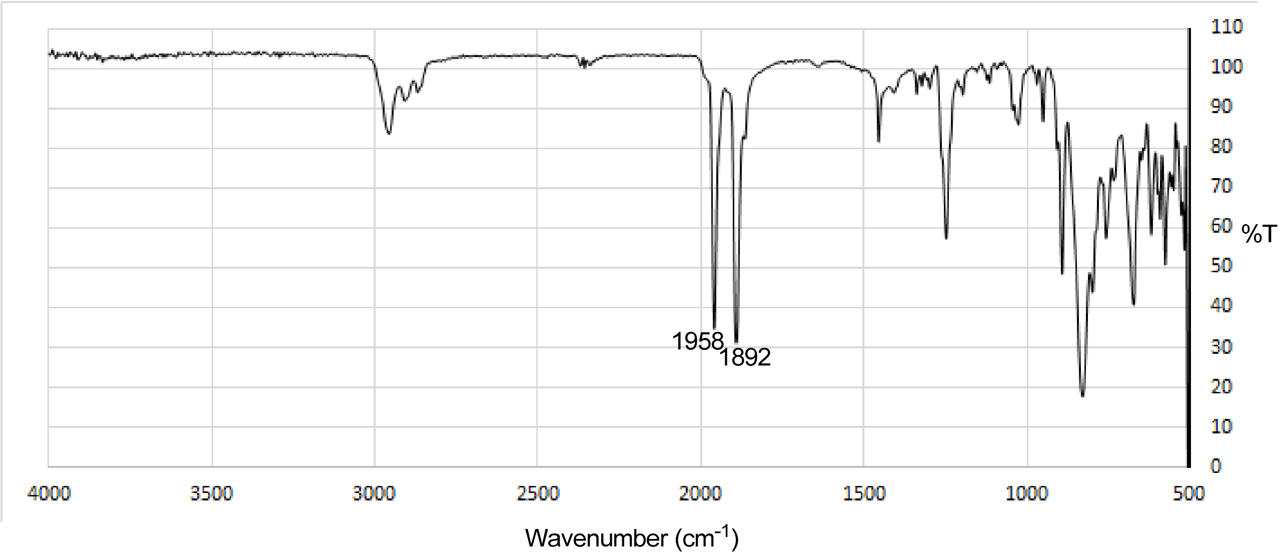

Figure S7-4. ATR-IR spectrum of **5** in the solid state.

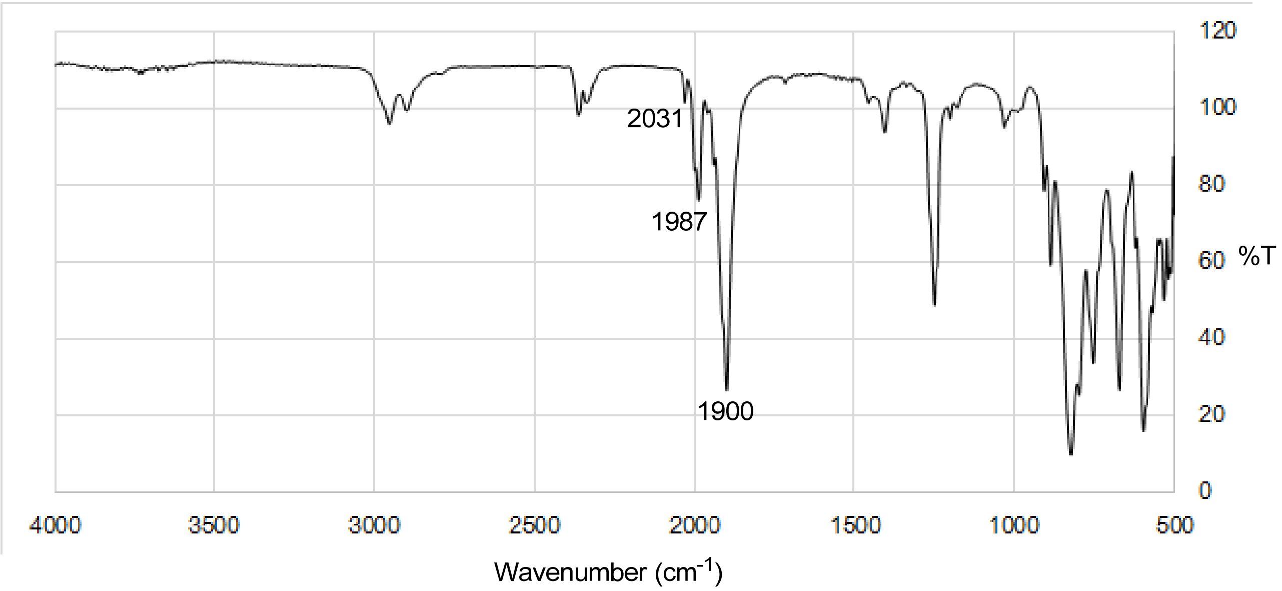

Figure S7-5. ATR-IR spectrum of **8** in the solid state.

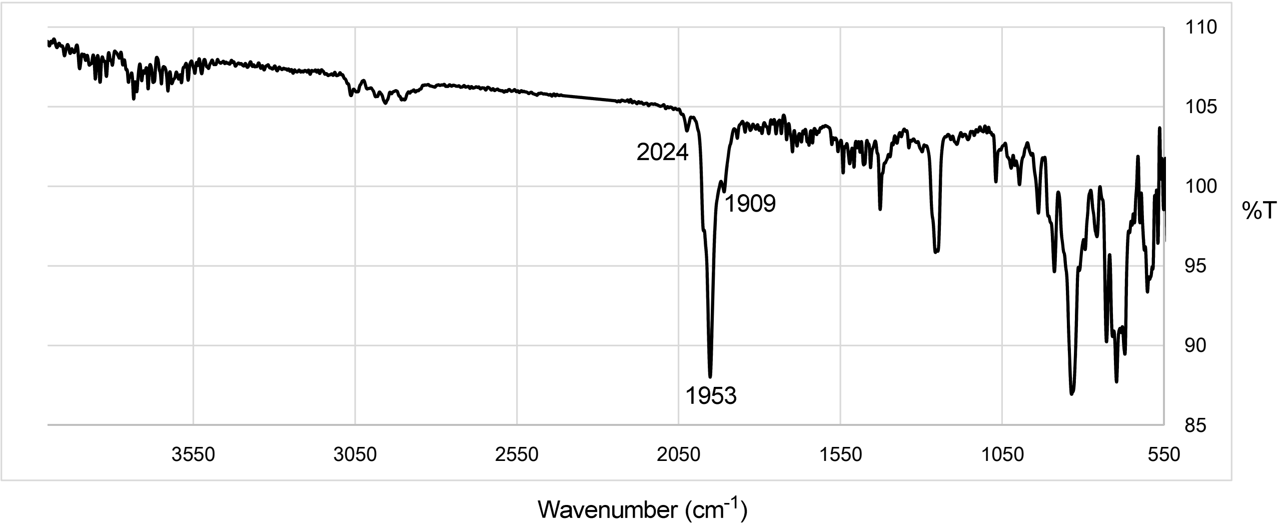

UV-vis-NIR spectra

An *n*-octane solution of **2** (ca. 0.12 mM) was prepared by dissolving **2** (1.3 mg, 1.24 x 10^{-3} mmol) in *n*-octane (10 mL) at room temperature. The UV-vis-NIR spectrum was recorded immediately at 293 K after preparing the solution (Figure S7-1). Next, this solution was allowed to stand for 10 min at 353 K, then the UV-vis-NIR spectrum was re-measured (Figure S7-2). Next, the sample was cooled to 293 K, and UV-vis-NIR spectra were recorded at 293K after 30 min, 1 h, 2 h, 3 h, and 5 h (Figure S7-3).

Figure S8-1. UV-vis-NIR spectrum of solution of **2** in *n*-octane at 293 K [λ_{max}/nm (ε/$M^{-1}cm^{-1}$) = 380 (3.02×10^4), 502 (6.26×10^3), 720 (2.78×10^3)].

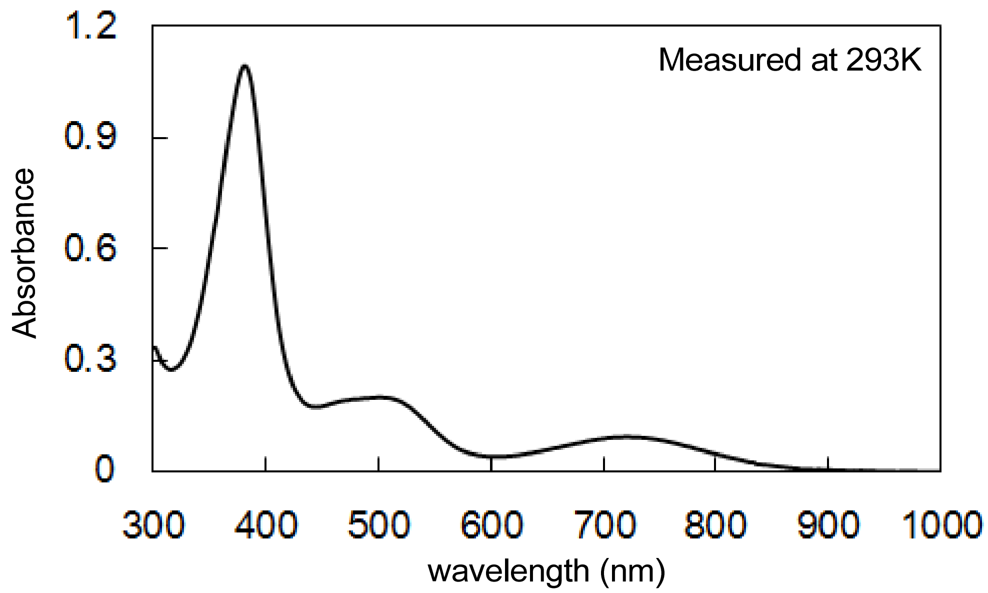

Figure S8-2. UV-vis-NIR spectrum of solution of **2** in *n*-octane at 353 K. [λ_{max}/nm (ε/$M^{-1}cm^{-1}$) = 362 (2.93×10^4), 496 (3.34×10^3), 818 (9.14×10^2)].

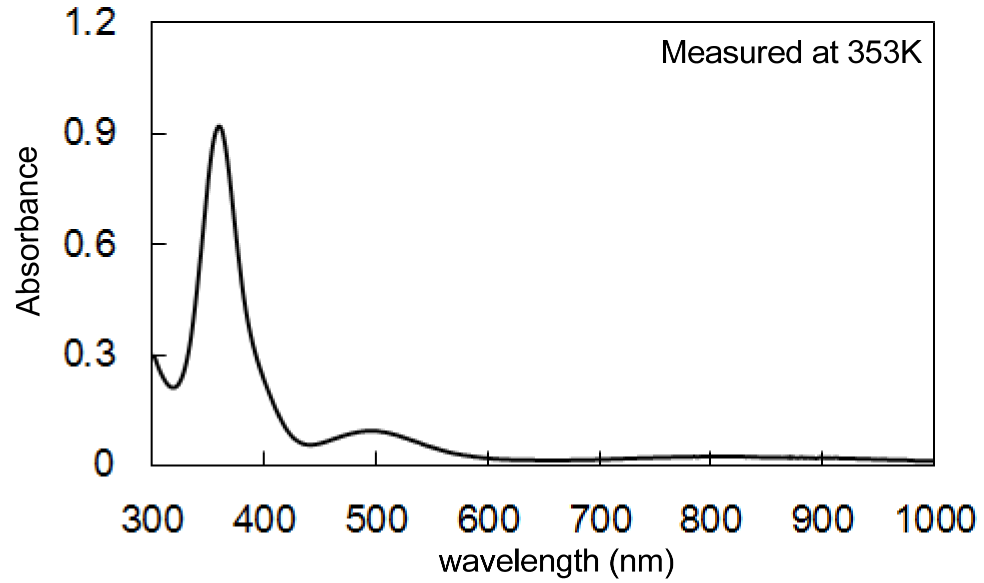

Figure S8-3. The time-course of UV-vis-NIR spectra of solution of **2** in *n*-octane before and after heating.

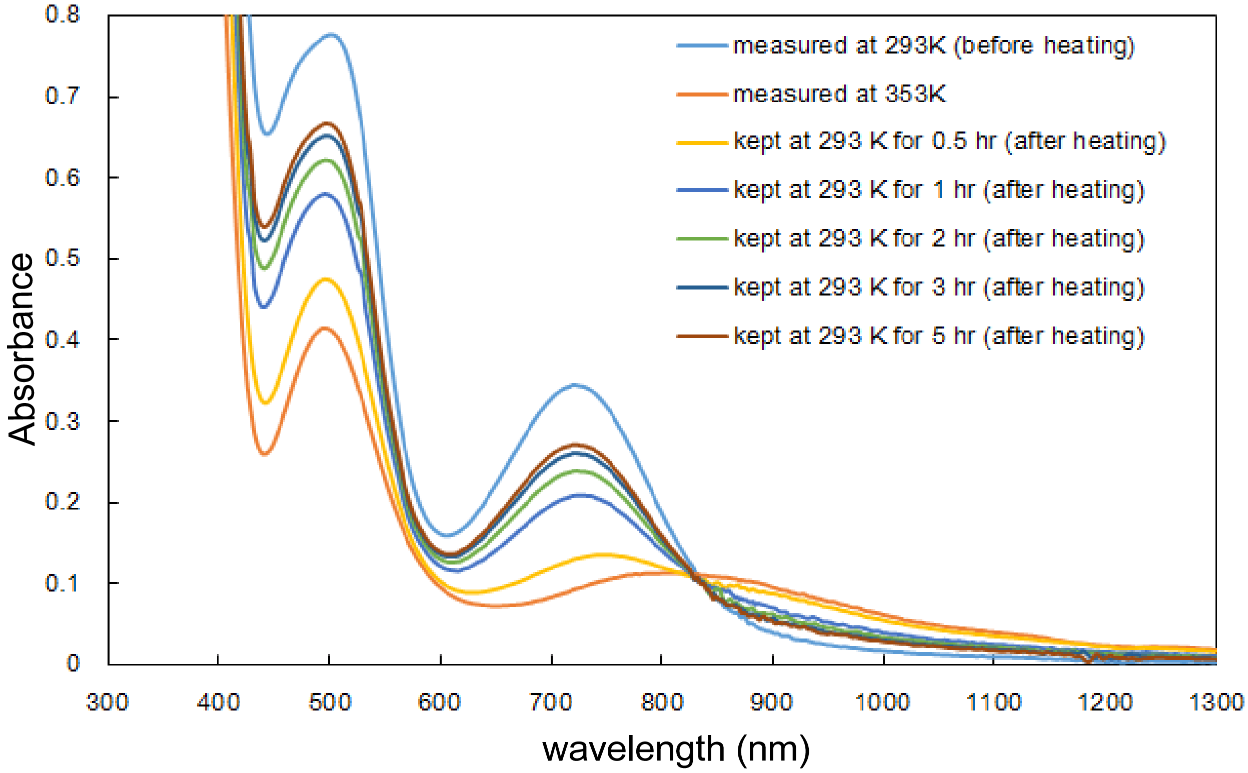

Thermodynamic analysis

The thermodynamic parameters were estimated using variable temperature ^{1}H NMR spectra. The equilibrium constant (K_{eq}) between **2** and **3** was estimated by two independent methods described below, and the K_{eq} values are summarized in Table S1-1 and S1-2. Plots of ln(K_{eq}) at various reciprocal temperatures are shown in Figure S5-1 and S5-2.

1) Estimation of K_{eq} using integral values

Complex **2** (10.0 mg, 9.7 x 10^{-3} mmol) was dissolved in C_6D_6 (16.5 mL); then mesitylene (2.7 µL, 1.95 x 10^{-2} mmol) was added as an internal standard. The initial concentration of **2** was ca. 0.588 mM. A portion of the solution (ca. 0.5 mL) was transferred into a J. Young NMR tube, and ^{1}H NMR spectra were measured at various temperatures. This solution was kept in the NMR instrument for 1 h at each temperature prior to the measurement, and ^{1}H NMR spectra were recorded at each temperature. The concentration of dinuclear complex **2** was calculated from the relative ratio of the integral value of the signals for the $SiMe_3$ group of **2** to that of the Me group of the internal standard (mesitylene), and the concentration of **3** was calculated from the following formula: [**3**]/2 = c_0 – [**2**] (c_0 = initial concentration of **2** = 0.588 mM). K_{eq} = [**3**]^2/[**2**].

Table S1-1. Equilibrium constants K_{eq} between **2** and **3** in C_6D_6 at various temperatures estimated by the values of integral.

Temp (K)	T^{-1} (K^{-1})	[**2**] (mmol L^{-1})	[**3**] (mmol L^{-1})	K_{eq} (mol L^{-1})	Ln(K_{eq})
343	0.002915	0.1068	0.9616	0.00866	-4.7494
333	0.003003	0.1705	0.8342	0.00408	-5.5013
323	0.003096	0.2515	0.6723	0.00180	-6.3215
313	0.003195	0.3132	0.5488	0.00096	-6.9467

Figure S9-1. A plot of ln(K_{eq}) vs 1/T for the equilibrium between **2** and **3**.

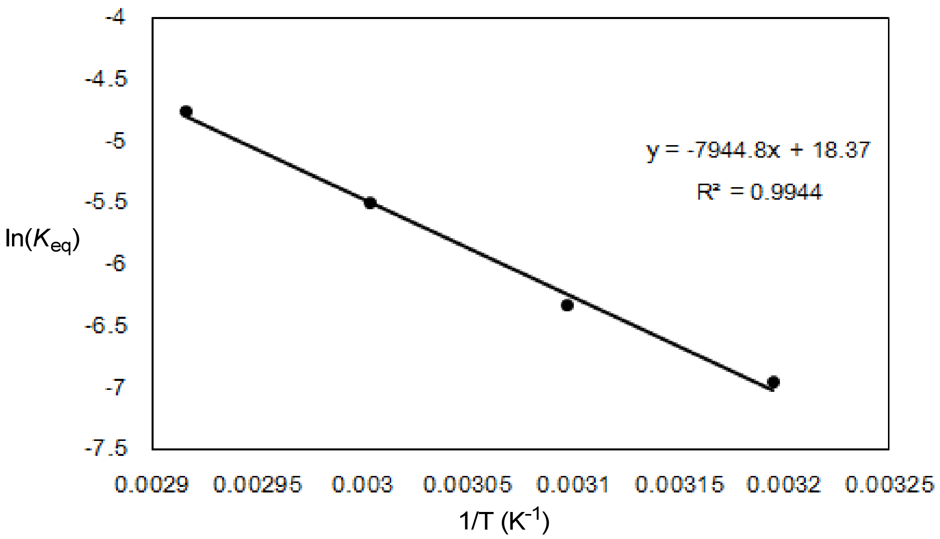

2) Estimation of K_{eq} by Evans method

Complex **2** (10.0 mg, 0.97 x 10^{-2} mmol) was dissolved in C_6D_6 (1.65 mL), then $SiMe_4$ (1.7 mg, 1.93 x 10^{-2} mmol) was added. The initial concentration of **2** was adjusted to ca. 5.88 mM. This solution was transferred into a J. Young NMR tube, and ^{1}H NMR spectra were measured at various temperatures. The solution was kept in the NMR instrument for 1 h at each temperature prior to measurement, and the ^{1}H NMR spectra were recorded at each temperature. The concentration of mononuclear complex **3** was estimated by the Evans method,[3] in which the number of unpaired electrons at the iron center was assumed to be 1, and the concentration of **2** was calculated from the following formula: [**2**] = c_0 – 0.5[**3**] (c_0 = initial concentration of **2** = 0.588 mM). K_{eq} = [**3**]^2/[**2**].

Table S1-2. Equilibrium constants K_{eq} between **2** and **3** in C_6D_6 at various temperatures estimated by using Evans method.

Temp (K)	T^{-1} (K^{-1})	[**2**] (mmol L^{-1})	[**3**] (mmol L^{-1})	K_{eq} (mol L^{-1})	Ln(K_{eq})
353	0.002833	2.1414	7.4696	0.02606	-3.6475
343	0.002915	2.7512	6.2501	0.01420	-4.2555
333	0.003003	3.5275	4.6975	0.00626	-5.0743
323	0.003096	4.2625	3.2275	0.00244	-6.0142
313	0.003195	4.7723	2.2078	0.00102	-6.8866

Figure S9-2. A plot of ln(K_{eq}) vs 1/*T* for the equilibrium between **2** and **3**.

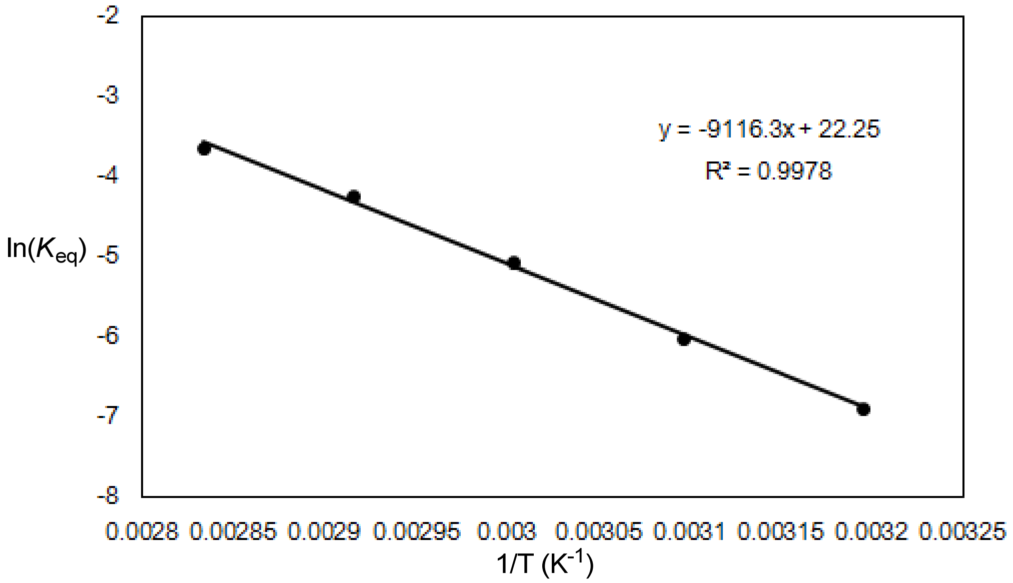

Figure S10-1. ^{1}H NMR spectrum (in C_6D_6 at room temperature) of the crude product obtained by the reaction of **2** with **1** in toluene at room temperature. The solid-circles and open-circles indicate signals of complex **5** and phosphaalkene **6**. Identification of **6** was achieved by comparison with the previously reported data.[2]

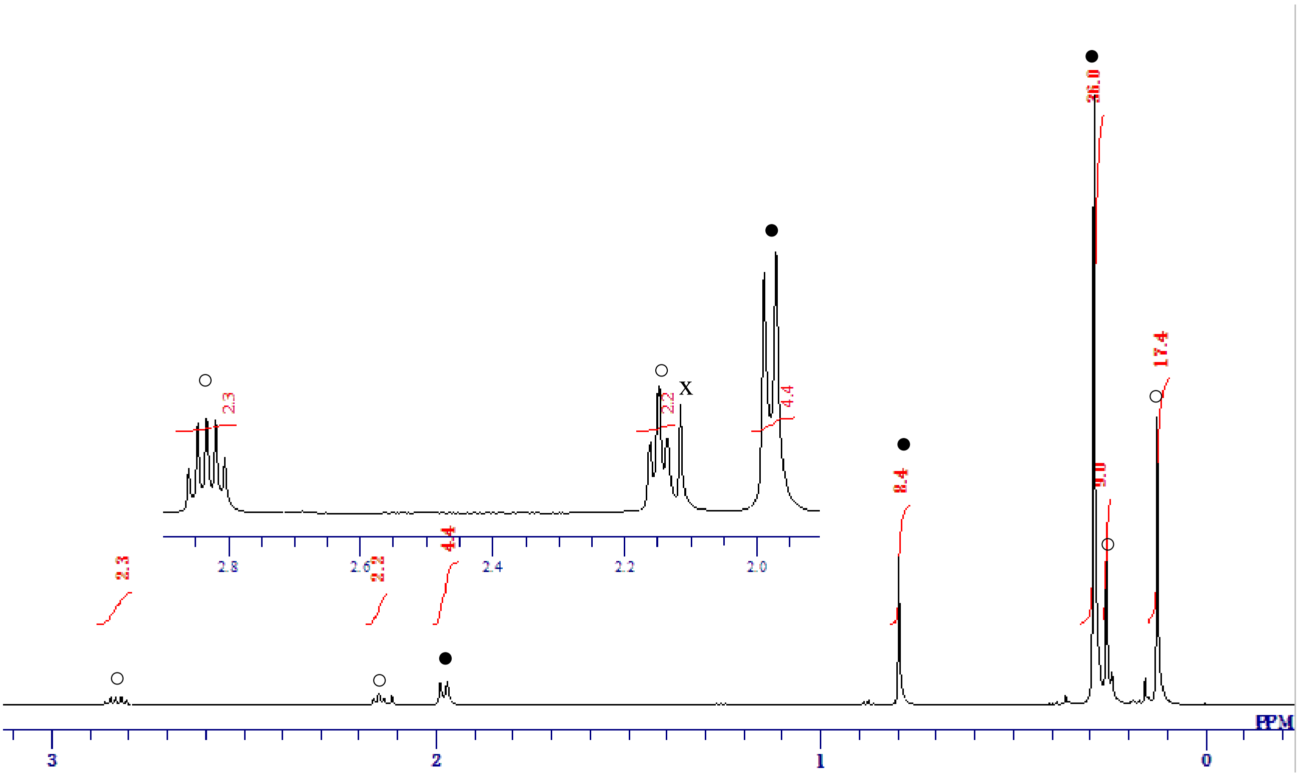

x = residual toluene

Figure S10-2. $^{31}P\{^1H\}$ NMR spectrum (in C_6D_6 at room temperature) of the crude product obtained by the reaction of **2** with **1** in toluene at room temperature. The solid-circles and open-circles indicate signals of **5** and **6**. Identification of **6** was achieved by comparison with the previously reported data.[2]

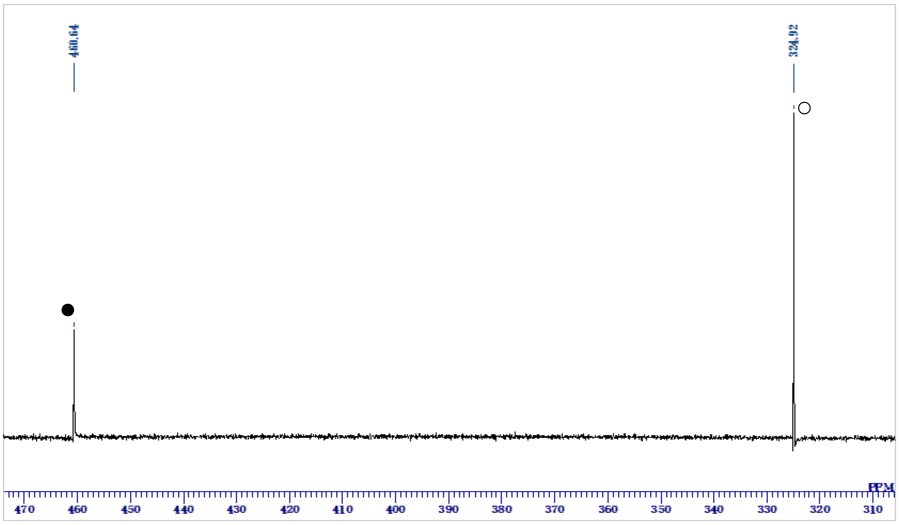

Figure S11-1. ^{1}H NMR spectrum (in C_6D_6 at room temperature) of the mixture of **8** and **9** formed by recrystallization of the crude product obtained from the reaction of **2** with $HSnPh_3$ in C_6D_6 at room temperature. The solid-circles and open-circles indicate signals of complex **8** and **9**.

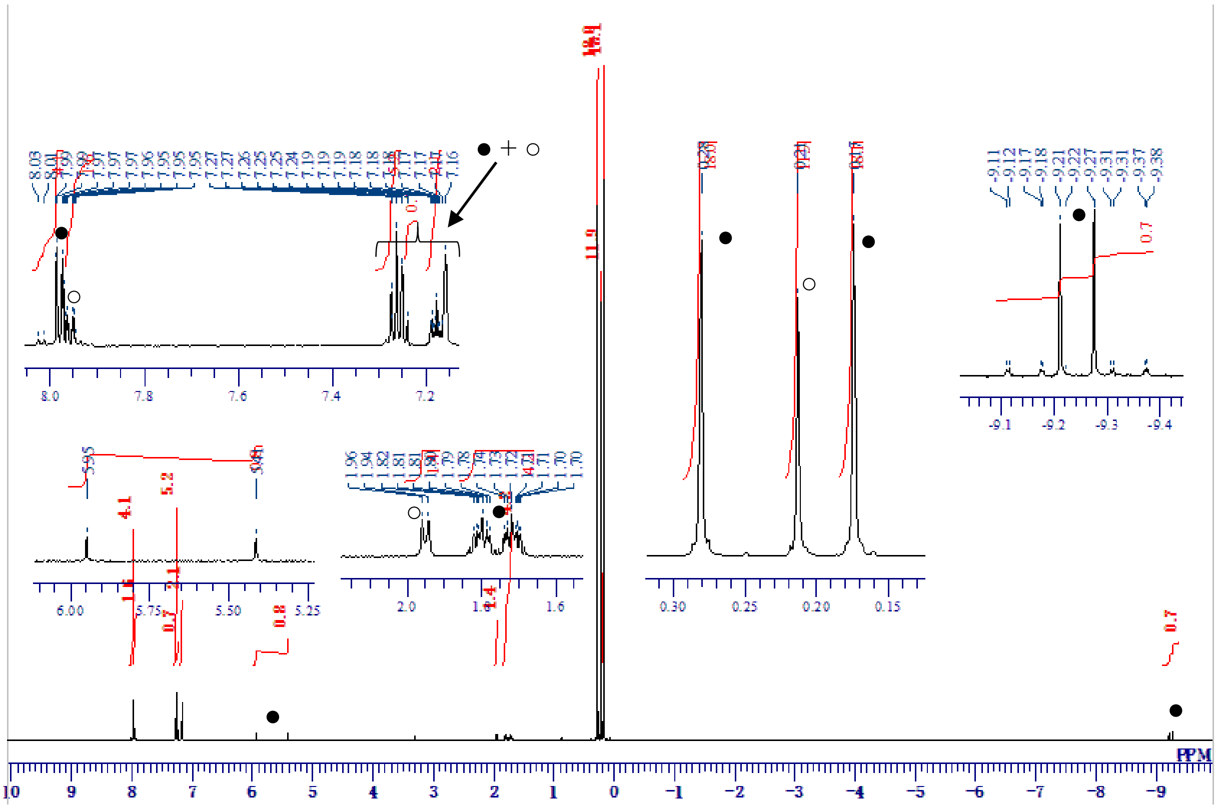

Figure S11-2. $^{13}C\{^{1}H\}$ NMR spectrum (in C_6D_6 at room temperature) of the mixture of **8** and **9** formed by recrystallization of the crude product obtained from the reaction of **2** with $HSnPh_3$ in C_6D_6 at room temperature. The solid-circles and open-circles indicate signals of complex **8** and **9**.

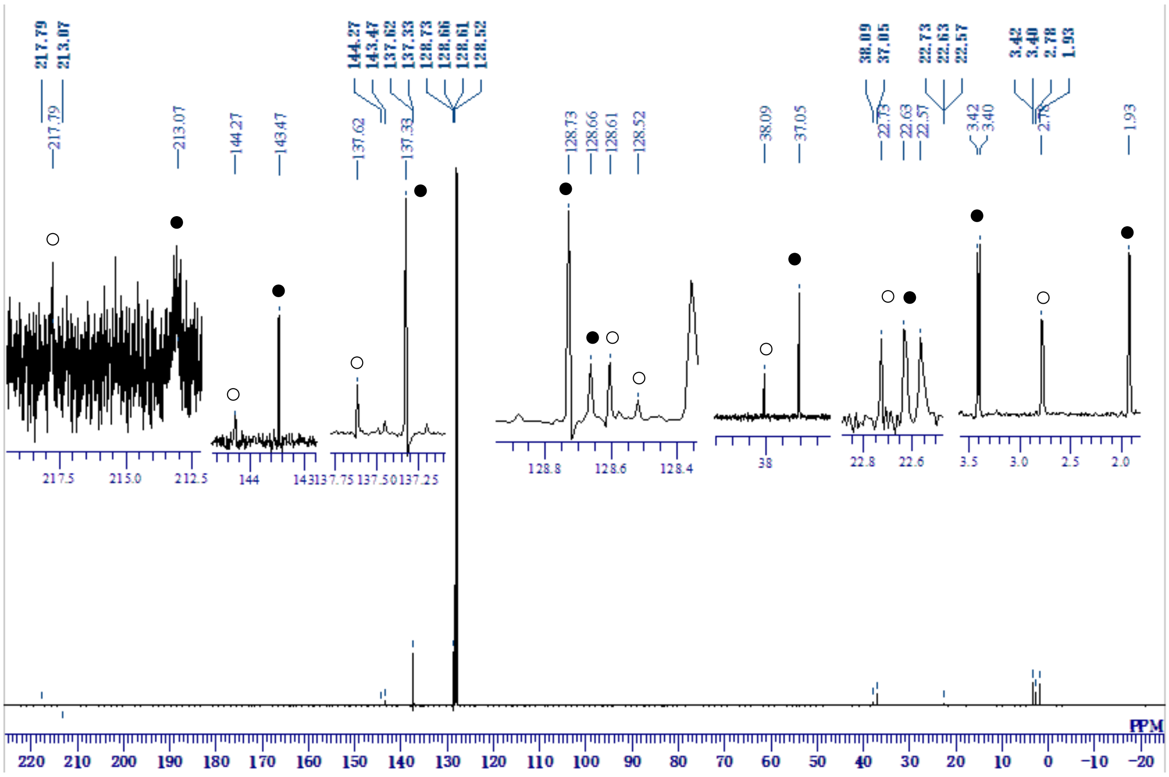

Figure S11-3. $^{31}P\{^1H\}$ NMR spectrum (in C_6D_6 at room temperature) of the mixture of **8** and **9** formed by recrystallization of the crude product obtained from the reaction of **2** with $HSnPh_3$ in C_6D_6 at room temperature. The solid-circles and open-circles indicate signals of complex **8** and **9**.

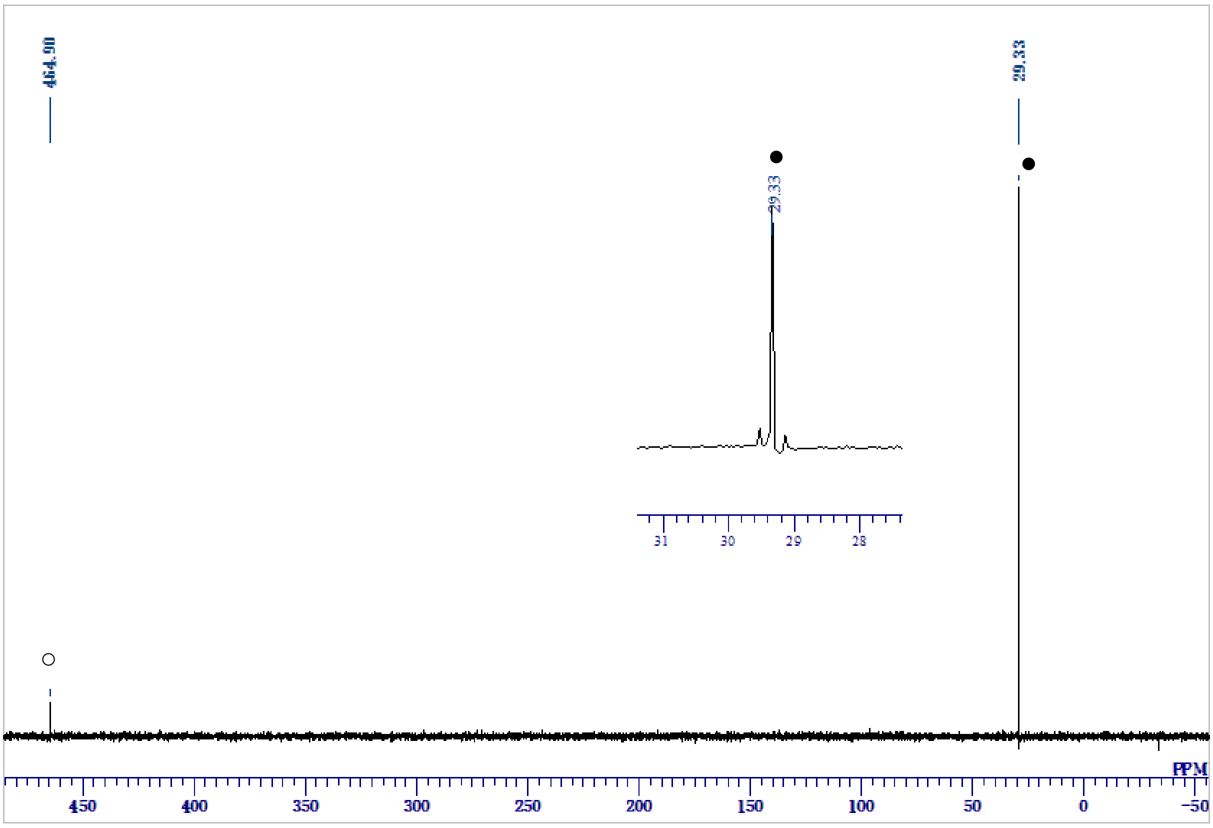

Figure S12-1. ^{1}H NMR spectrum (in C_6D_6 at room temperature) of the crude product obtained by the reaction of **2** with $HSnPh_3$ in C_6D_6 at room temperature. The solid-triangle, solid-circles, and open-circles indicate signals of free phosphine **7**, complex **8** and **9**. Identification of **7** was achieved by comparison with the previously reported data.[2] The solid-square indicates signals of internal standard (1,3,5-trimethoxybenzene).

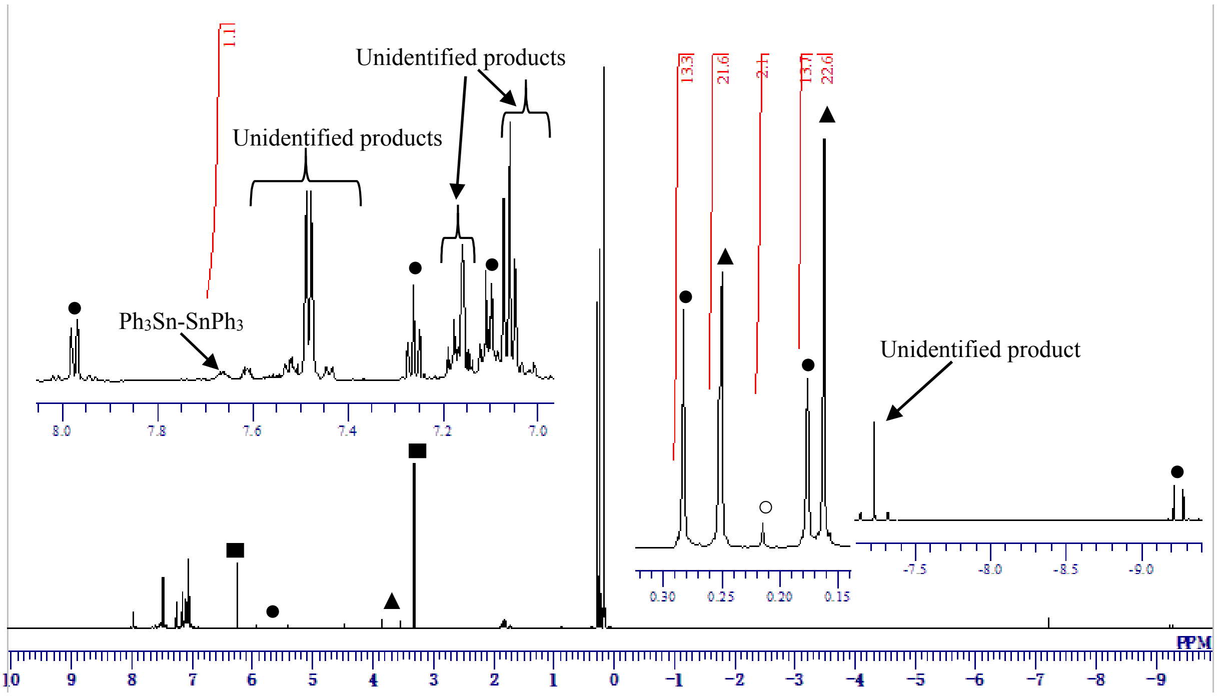

Figure S12-2. $^{31}P\{^{1}H\}$ NMR spectrum (in C_6D_6 at room temperature) of the crude product obtained by the reaction of **2** with $HSnPh_3$ in C_6D_6 at room temperature. The solid-triangles, solid-circles, and open-circles indicate signals of free phosphine **7**, complex **8** and **9**. Identification of **7** was achieved by comparison with the previously reported data.[2]

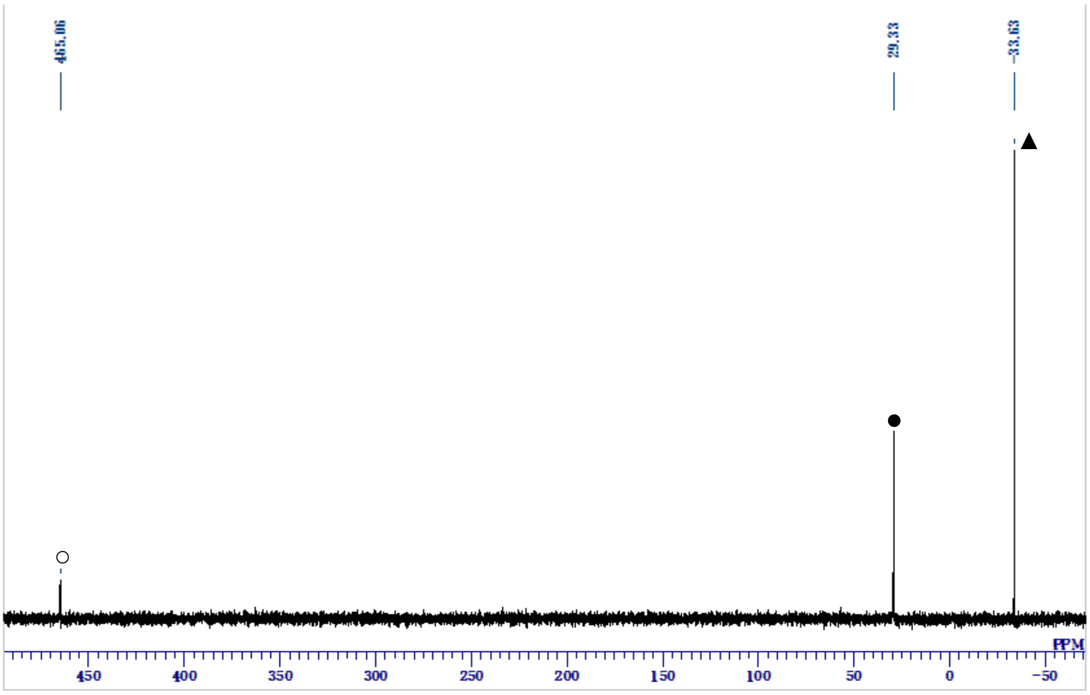

Figure S12-3. ^{31}P off-resonance NMR spectrum (in C_6D_6 at room temperature) of the crude product obtained by the reaction of **2** with $HSnPh_3$ in C_6D_6 at room temperature. The solid-triangles and solid-circles indicate signals of free phosphine **7** and complex **8**. Identification of **7** was achieved by comparison with the previously reported data.[2]

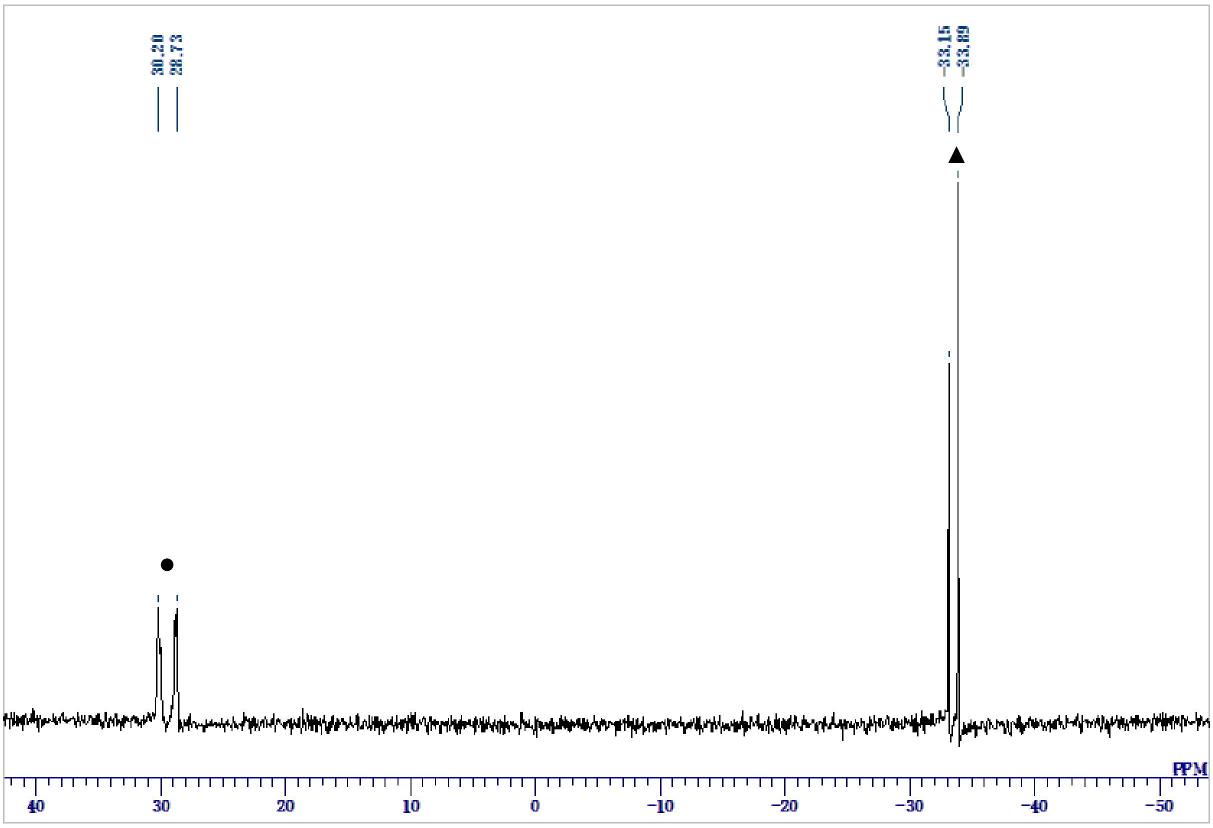

Figure S13-1. ^{1}H NMR spectrum (in C_6D_6 at room temperature) of the crude product obtained by the reaction of **2** with $HSnBu_3$ in C_6D_6 at room temperature. The solid-triangle and solid-circles indicate signals of free phosphine **7** and complex **8'**. Identification of **7** was achieved by comparison with the previously reported data.[2]

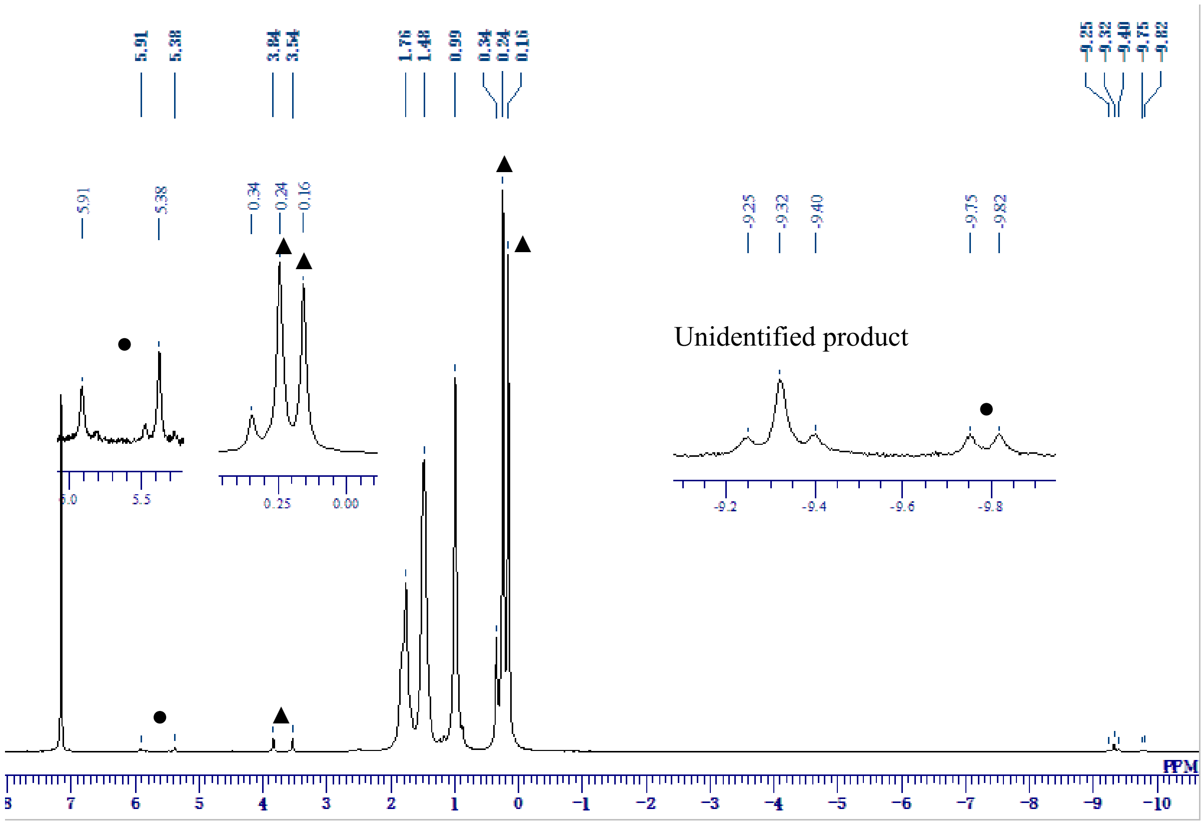

Figure S13-2. ^{31}P{^{1}H} NMR spectrum (in C_6D_6 at room temperature) of the crude product obtained by the reaction of **2** with $HSnBu_3$ in C_6D_6 at room temperature. The solid-triangle and solid-circles indicate signals of free phosphine **7** and complex **8'**. Identification of **7** was achieved by comparison with the previously reported data.[2]

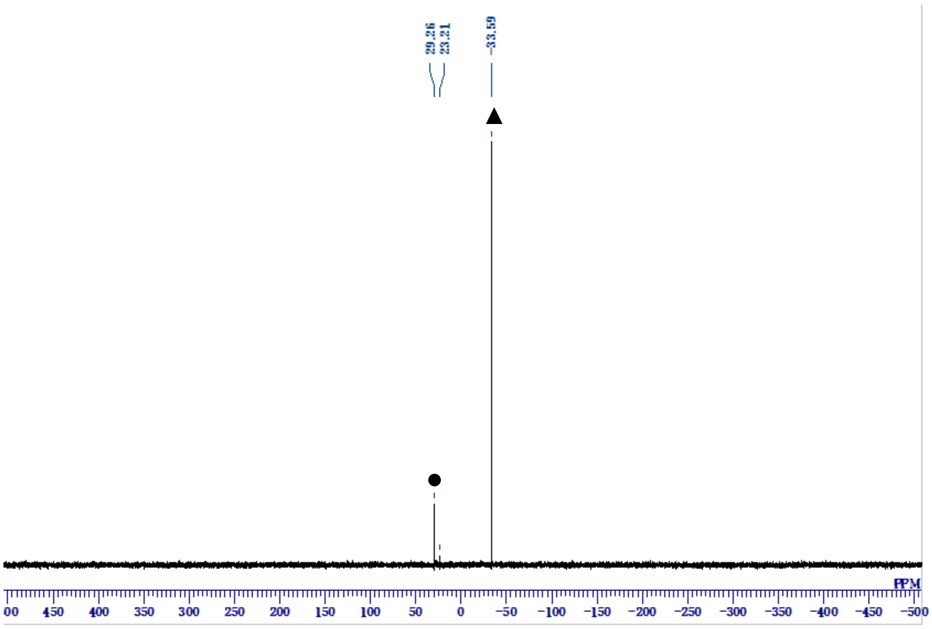

Figure S13-3. ^{31}P-off resonance NMR spectrum (in C_6D_6 at room temperature) of the crude product obtained by the reaction of **2** with $HSnBu_3$ in C_6D_6 at room temperature. The solid-triangle and solid-circles indicate signals of free phosphine **7** and complex **8'**. Identification of **7** was achieved by comparison with the previously reported data.[2]

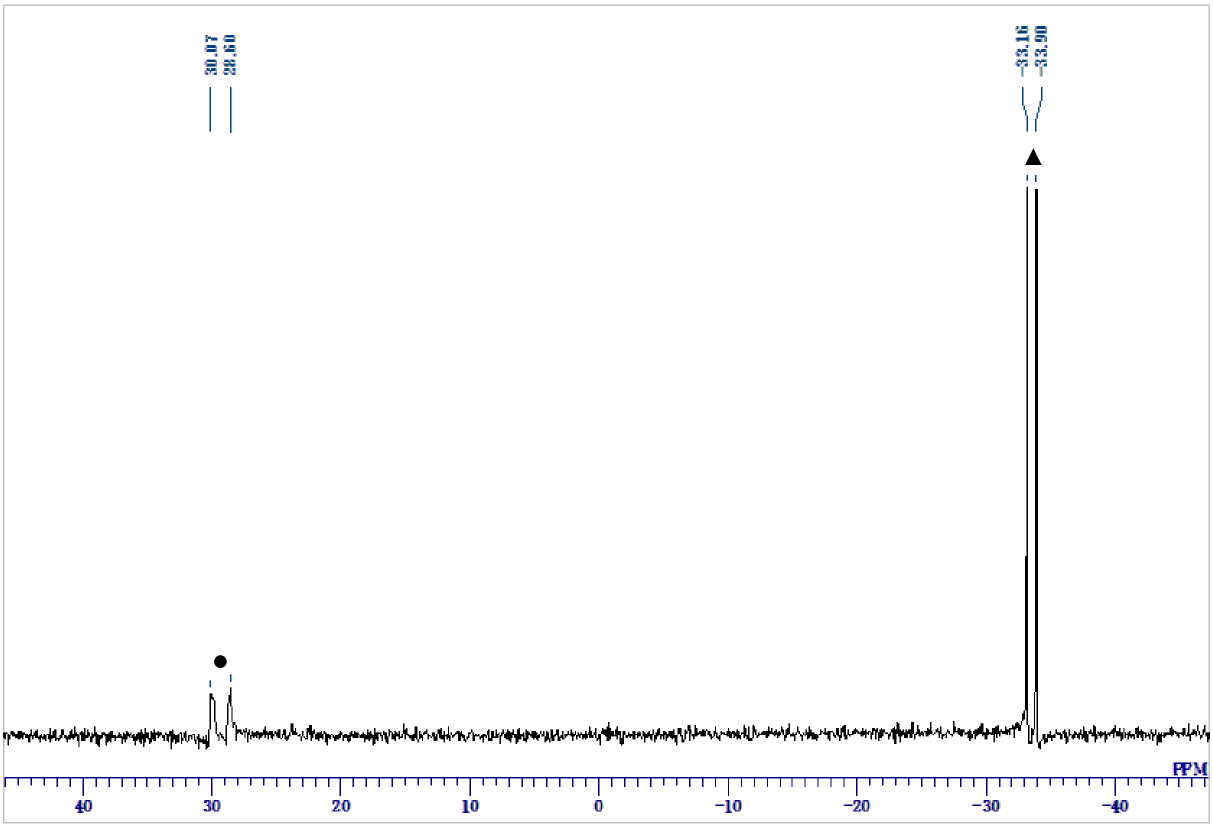

Figure S14-1. ^{1}H NMR spectrum (in C_6D_6 at room temperature) of the crude product obtained by the reaction of **2** with 9,10-dihydroanthracne in C_6D_6 at 353 K for 24 h. The solid-triangle indicates signal of free phosphine **7**, and the solid-square indicates signals of internal standard (1,3,5-trimethoxybenzene). The solid-circles indicate signal of phosphaalkene **6**. Identification of **6** was achieved by comparison with the previously reported data.[2]

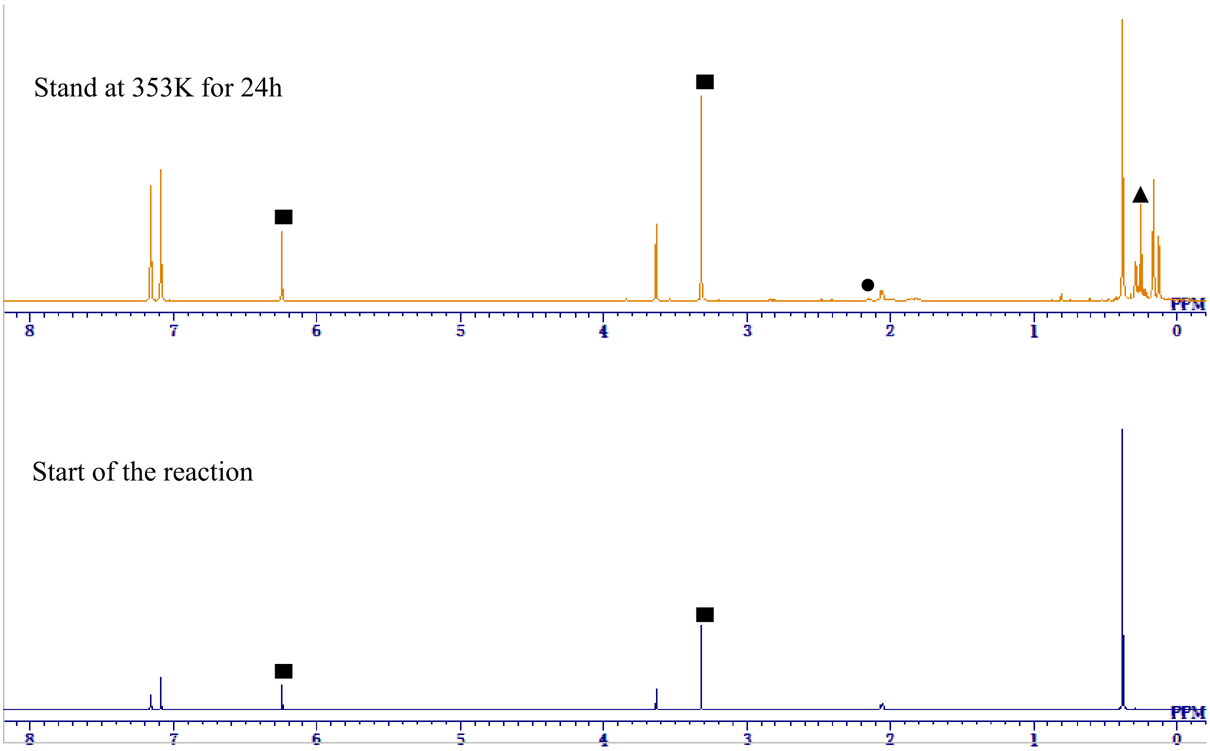

Figure S14-2. ^{31}P{^{1}H} NMR spectrum (in C_6D_6 at room temperature) of the crude product obtained by the reaction of **2** with 9,10-dihydroanthracne in C_6D_6 at 353 K for 24 h. The solid-triangle indicates signal of free phosphine **7**. The solid-circles indicate signal of phosphaalkene **6**. Identification of **6** was achieved by comparison with the previously reported data.[2]

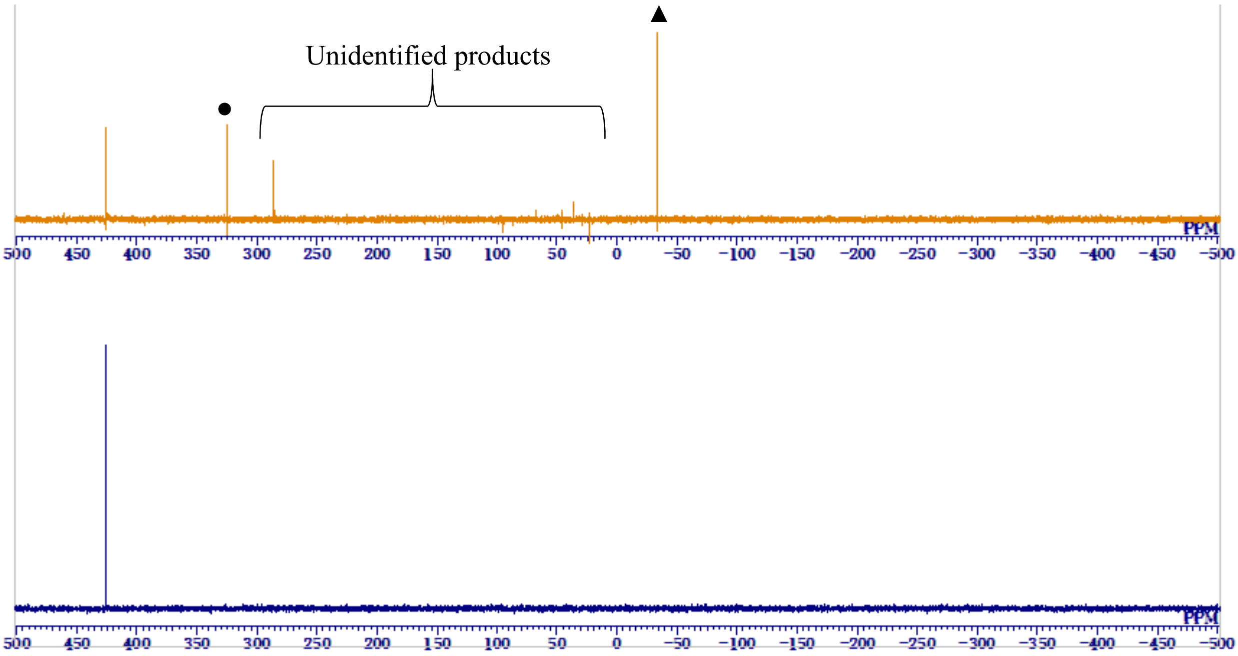

Theoretical calculations

All theoretical calculations were performed using the Gaussian 09 program package.[4] All calculations of the compounds were carried out at the PBE0, B3LYP, and CAM-B3LYP functionals[5] (Wachters-Hay basis[6,7] for Fe atoms and D95** basis[8] for H, and C, O, P, Si atoms). The Mayer bond order[9] is used as a natural extension of the Wiberg bond order. No imaginary frequencies were found in the optimized structures. The optimized structure of $\mathbf{2_{opt}}$ and $\mathbf{3_{opt}}$ obtained by the calculation at the PBE0 level are shown in Figures S15 and S18, and their MO diagrams are summarized in Figures S16 and S19. Atomic coordinates and their energies of $\mathbf{2_{opt}}$ and $\mathbf{3_{opt}}$ calculated by PBE0 level of theory are summarized in Tables S3 and S4. The transition energies and oscillator strengths of all electron transitions of $\mathbf{2_{opt}}$ and $\mathbf{3_{opt}}$ were calculated by using the time-dependent DFT method[10] (TD-DFT) at the PBE0, B3LYP or CAM-B3LYP level, and the results obtained by the calculation at the PBE0 level are summarized in Tables S5 and S6. Density distributions in (a) HOMO and (b) HOMO-1 of $\mathbf{2_{opt}}$ as well as the density distributions in (a) αHOMO and (b) βHOMO of $\mathbf{3_{opt}}$ are shown in Figures S17 and S21. The orbital interactions between the P ligand and the $Fe(CO)_3$ moiety in $\mathbf{3_{opt}}$ are summarized in Figure S20. Electron density difference map at the PBE0 level are summarized in Figures S22 and S23. The results of TD-DFT calculated by B3LYP or CAM-B3LYP are summarized in Figure S24.

Table S2. Comparison between the DFT-optimized geometry and experimental structure of isolated complex **2**. Selected bond lengths and bond angles are shown.

	Calcd. by PBE0	Calcd. by B3LYP	Calcd. by CAM-B3LYP	Exptl.
Bond lengths (Å)				
Fe(1)-Fe(2)	2.715	2.824	2.748	2.7374(10)
Fe(1)-P(1)	2.095	2.129	2.091	2.0934(12)
Fe(2)-P(2)	2.096	2.128	2.091	2.1047(13)
Fe(1)-C(17)	1.781	1.799	1.788	1.796(4)
Fe(1)-C(18)	1.740	1.761	1.756	1.771(4)
Fe(1)-C(19)	1.777	1.796	1.785	1.794(4)
Fe(2)-C(36)	1.780	1.800	1.788	1.799(4)
Fe(2)-C(37)	1.740	1.761	1.755	1.766(4)
Fe(2)-C(38)	1.778	1.794	1.786	1.790(4)
Bond angles (deg)				
Fe(2)-Fe(1)-P(1)	139.55	138.76	138.75	142.66(4)
Fe(1)-P(1)-C(1)	129.42	130.06	129.54	130.01(14)
Fe(1)-P(1)-C(4)	132.36	131.30	131.98	131.20(11)
C(1)-P(1)-C(4)	96.71	97.02	96.97	97.32(18)
Fe(1)-Fe(2)-P(2)	138.69	140.71	140.77	132.61(4)
Fe(2)-P(2)-C(20)	131.61	131.57	132.90	132.13(15)
Fe(2)-P(2)-C(23)	129.97	129.79	128.09	129.04(12)
C(20)-P(2)-C(23)	96.76	97.00	97.04	97.55(19)

Figure S15. Optimized structure of $\mathbf{2_{opt}}$.

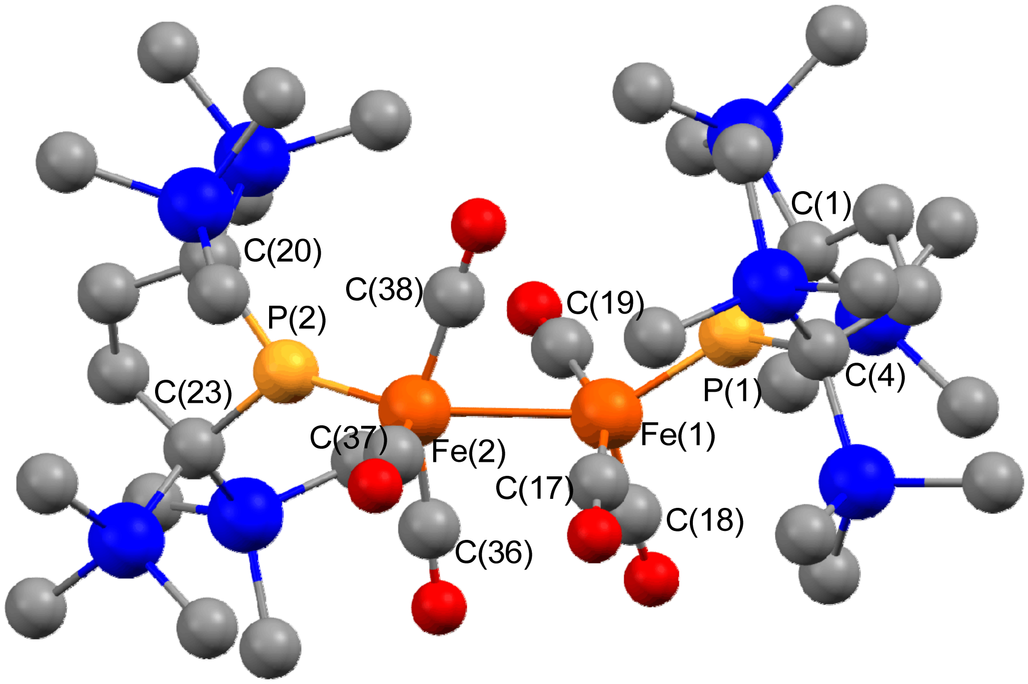

Tabale S3. Cartesian coordinates of $\mathbf{2_{opt}}$ in the closed shell singlet state.

Fe	-1.258627	0.792395	-0.547262
Fe	1.277433	0.878792	0.419366
P	-3.124233	-0.023942	-0.057054
P	3.133201	-0.023584	0.053274
Si	-4.702706	-1.113092	-2.603791
Si	-3.283158	-3.227160	-0.713892
Si	-4.950003	2.603942	0.613370
Si	-4.182997	0.461212	2.862503
Si	3.864462	-1.502013	2.855710
Si	3.503320	-3.180998	0.178361
Si	5.479537	2.190162	-0.173907
Si	4.238285	0.913769	-2.818435
O	-1.316052	3.237825	1.080658
O	-1.421825	2.340748	-2.990170
O	-0.039283	-1.449050	-1.998729
O	1.340410	3.062437	-1.544461
O	1.498368	2.805363	2.571375
O	0.011503	-1.090116	2.193986
C	-4.112393	-1.454063	-0.779644
C	-5.348265	-1.532136	0.155799
C	-5.769337	-0.130106	0.580846
C	-4.519843	0.715656	0.959650
C	-5.974853	0.283924	-2.766083
C	-5.664639	-2.617527	-3.253024
C	-3.286298	-0.726902	-3.785470
C	-4.655729	-4.515421	-0.461018
C	-2.342049	-3.775943	-2.261725
C	-2.121040	-3.425934	0.757647
C	-4.254759	3.289755	-1.008022
C	-4.431936	3.854562	1.938114
C	-6.842190	2.749456	0.514829
C	-5.726062	1.016907	3.822015
C	-3.930240	-1.350916	3.341441

C	-2.649066	1.339477	3.514779
C	-1.294556	2.236332	0.495441
C	-1.315844	1.735551	-2.008391
C	-0.519441	-0.589724	-1.383865
C	4.017016	-1.449421	0.901855
C	5.514826	-1.226841	0.551226
C	5.631562	-0.559804	-0.814841
C	4.615616	0.611498	-0.914147
C	5.550755	-2.043962	3.540102
C	2.600110	-2.710987	3.580472
C	3.447486	0.162269	3.650424
C	4.494735	-4.543230	1.056249
C	1.663110	-3.544691	0.312263
C	3.963795	-3.402500	-1.646570
C	6.289325	1.916995	1.520117
C	6.961856	2.637524	-1.275376
C	4.343307	3.680834	0.011664
C	2.679090	0.032008	-3.411744
C	4.035064	2.710730	-3.382030
C	5.699868	0.225208	-3.815775
C	1.322886	2.160689	-0.814976
C	1.367558	2.037967	1.714128
C	0.506853	-0.351215	1.446781
H	-6.185642	-2.048864	-0.330623
H	-5.105930	-2.119234	1.048923
H	-6.483924	-0.186857	1.411264
H	-6.308352	0.335539	-0.246892
H	-6.139963	0.445823	-3.839132
H	-5.658650	1.238532	-2.339584
H	-6.940994	0.008781	-2.328180
H	-6.059367	-2.348342	-4.240895
H	-6.524972	-2.853084	-2.616350
H	-5.069810	-3.526495	-3.372100
H	-2.934602	0.301182	-3.665142
H	-3.654362	-0.830502	-4.813840
H	-2.430288	-1.395680	-3.666910
H	-5.171050	-4.397095	0.497719
H	-4.182068	-5.505372	-0.454018
H	-5.408483	-4.517020	-1.254192
H	-2.073773	-4.828304	-2.101554
H	-1.414529	-3.222552	-2.419603
H	-2.932800	-3.728256	-3.180727
H	-1.408283	-2.605079	0.862397
H	-1.548418	-4.351501	0.621710
H	-2.676785	-3.518989	1.695875
H	-4.910782	4.106034	-1.335998
H	-4.189461	2.564738	-1.822400
H	-3.255653	3.710357	-0.863647
H	-4.871008	4.813467	1.632620
H	-3.350479	3.995885	1.986742
H	-4.802007	3.632535	2.942619
H	-7.094844	3.816901	0.522034
H	-7.350794	2.283613	1.365491
H	-7.257260	2.322880	-0.404415
H	-6.552682	0.310137	3.686650
H	-6.090692	2.013938	3.560112
H	-5.482771	1.025089	4.891699
H	-3.764385	-1.380388	4.425941
H	-3.039214	-1.768682	2.864353
H	-4.789196	-1.994385	3.126691
H	-2.653853	2.422732	3.385724
H	-1.746660	0.938408	3.042969
H	-2.577807	1.125578	4.588710
H	5.993526	-0.588767	1.298473
H	6.073757	-2.171385	0.558069
H	5.433951	-1.304440	-1.591228
H	6.660495	-0.216286	-0.980732
H	5.432364	-2.241375	4.612644
H	6.318567	-1.270521	3.433515
H	5.923694	-2.961143	3.073667
H	1.569495	-2.442401	3.337783

H	2.705287	-2.639878	4.671210
H	2.761378	-3.756776	3.305554
H	2.365103	0.304040	3.719347
H	3.856493	1.033241	3.133641
H	3.845863	0.158491	4.672888
H	5.572196	-4.435963	0.886963
H	4.196814	-5.504453	0.618727
H	4.327102	-4.606981	2.134349
H	1.484173	-4.558563	-0.067237
H	1.086103	-2.854459	-0.308966
H	1.277941	-3.492942	1.332385
H	3.564328	-4.373181	-1.966882
H	5.046620	-3.427401	-1.809899
H	3.527805	-2.641398	-2.299321
H	5.610043	1.545119	2.291147
H	6.659400	2.893783	1.857761
H	7.154569	1.247162	1.465213
H	7.708449	1.835173	-1.289048
H	7.448177	3.518035	-0.836660
H	6.711961	2.882400	-2.310180
H	4.953337	4.537551	0.324010
H	3.592781	3.512606	0.790068
H	3.823952	3.954714	-0.909078
H	1.774875	0.533473	-3.056704
H	2.609930	-1.014042	-3.102450
H	2.670028	0.060858	-4.508760
H	3.126443	3.176381	-2.995963
H	3.940323	2.673595	-4.475245
H	4.882030	3.361766	-3.152116
H	5.565959	0.524779	-4.862480
H	5.751398	-0.868258	-3.793866
H	6.665394	0.618782	-3.481608

Figure S16. MO diagrams for $\mathbf{2}_{\mathbf{opt}}$ obtained from DFT calculations at the PBE0 level.

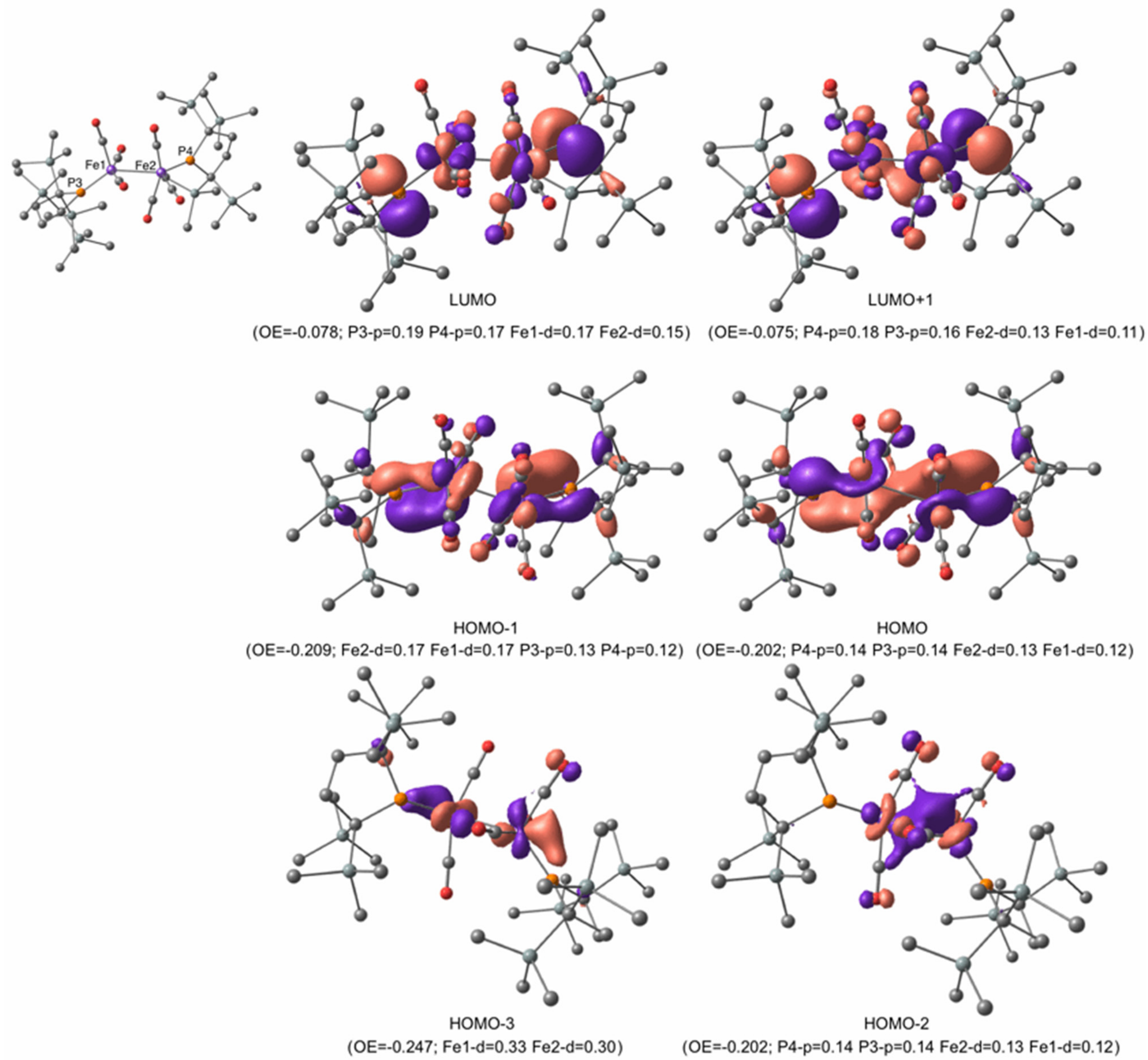

Figure S17. Density distributions in (a) HOMO and (b) HOMO-1 of **2_{opt}**. The two plot planes (left and right) indicate the π-bonding interaction in the Fe-P bonds.

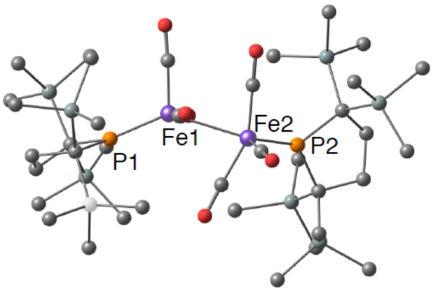

(a) HOMO

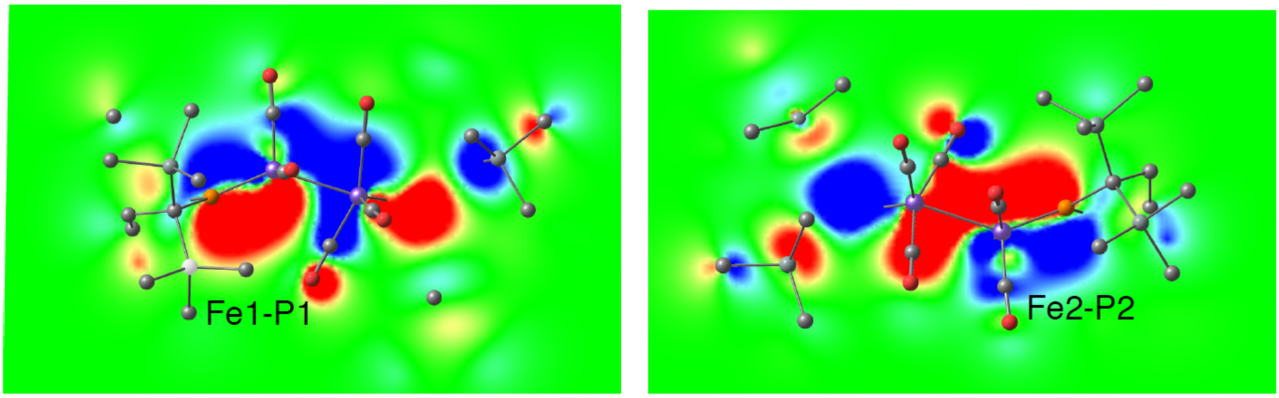

(b) HOMO-1

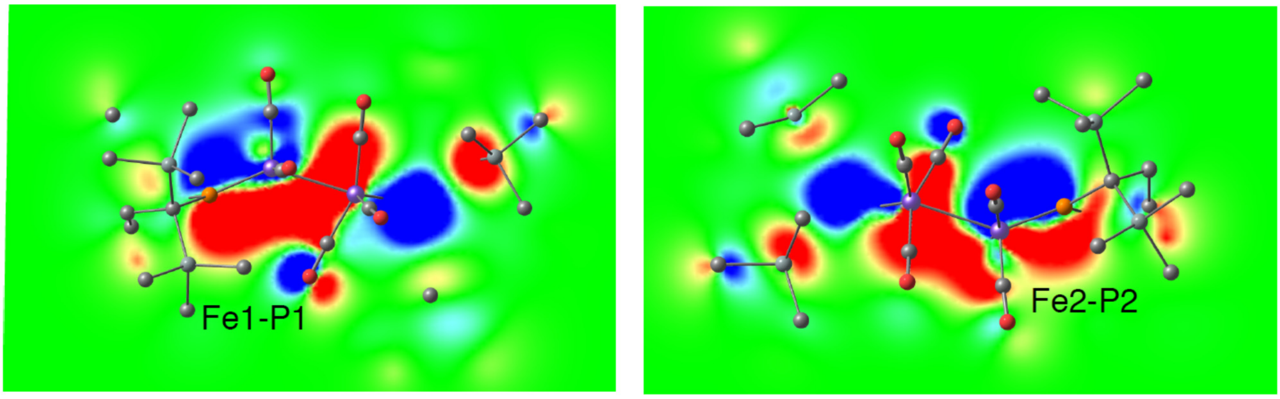

The plot planes are arranged in parallel with the Fe, P, and C(CO) atoms.

Figure S18. Optimized structure of $\mathbf{3_{opt}}$.

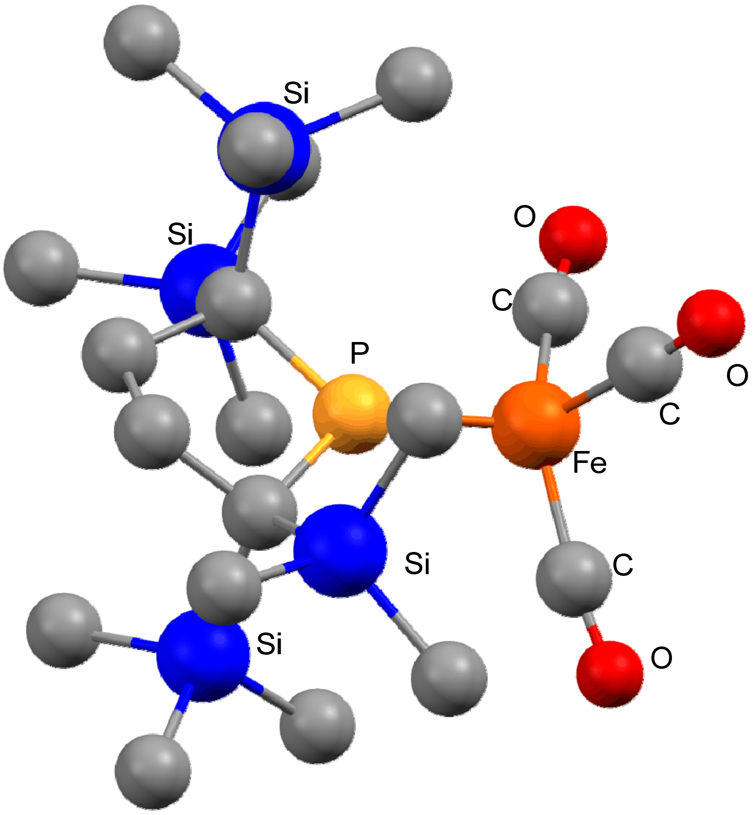

Table S4. Cartesian coordinates of $\mathbf{3_{opt}}$ in the doublet state.

Fe	0.016360	2.207397	-0.903133
P	0.000582	0.219536	-0.099467
Si	2.232402	-1.840889	-1.112871
Si	2.733619	-0.017642	1.484496
Si	-2.570703	-1.300321	-1.182608
Si	-2.453579	-0.286028	1.847931
O	2.716348	2.623756	-2.060478
O	-2.783231	2.840750	-1.643724
O	0.213187	4.417692	1.009671
C	1.697717	2.381501	-1.571189
C	-1.720901	2.504917	-1.331720
C	0.134169	3.570612	0.222746
C	1.397328	-0.926784	0.393123
C	0.693306	-1.963514	1.310104
C	-0.708774	-2.253718	0.772330
C	-1.408001	-0.930034	0.345659
C	2.452803	-0.750815	-2.634966
C	3.934594	-2.538367	-0.647879
C	1.289719	-3.395723	-1.659737
C	1.959852	1.177016	2.728561
C	4.025469	0.967276	0.515574
C	3.637450	-1.306035	2.542651
C	-3.127687	-3.110631	-1.072097
C	-4.155512	-0.273320	-1.292619
C	-1.687919	-1.059415	-2.837930
C	-3.887118	-1.489512	2.170641
C	-3.124788	1.461013	1.630734
C	-1.465112	-0.284022	3.459932
H	1.271775	-2.894196	1.383965
H	0.611820	-1.571304	2.331977
H	-1.298001	-2.796518	1.523223
H	-0.617301	-2.930698	-0.080870
H	3.171087	0.058014	-2.474373

H	1.510373	-0.301879	-2.965012
H	2.834541	-1.376129	-3.451560
H	3.866383	-3.280605	0.154727
H	4.669897	-1.782881	-0.357379
H	4.325089	-3.053506	-1.534694
H	1.892921	-3.870063	-2.444372
H	0.300481	-3.204155	-2.083671
H	1.184116	-4.126322	-0.849860
H	1.244043	1.866026	2.271789
H	2.770916	1.775784	3.161848
H	1.469848	0.651509	3.554366
H	4.911966	1.074399	1.153091
H	3.667613	1.974434	0.286289
H	4.347930	0.505324	-0.421549
H	2.944650	-1.908076	3.140195
H	4.285875	-0.767176	3.244970
H	4.267489	-1.984343	1.963109
H	-2.316415	-3.821668	-1.259862
H	-3.896125	-3.282777	-1.835629
H	-3.569442	-3.349407	-0.099008
H	-3.970830	0.788024	-1.469160
H	-4.805333	-0.370087	-0.418541
H	-4.713763	-0.653513	-2.157836
H	-1.497911	0.000193	-3.036128
H	-2.335279	-1.444988	-3.635709
H	-0.729293	-1.582348	-2.904937
H	-4.612477	-1.559567	1.355326
H	-4.425721	-1.143200	3.061456
H	-3.524288	-2.500992	2.386807
H	-2.309042	2.183698	1.525995
H	-3.691319	1.726702	2.531765
H	-3.791393	1.569605	0.772090
H	-0.618338	0.406079	3.423964
H	-1.101176	-1.277879	3.741935
H	-2.133813	0.057190	4.260185

Figure S19. MO diagrams for $\mathbf{3}_{opt}$ obtained from DFT calculations at the PBE0 level.

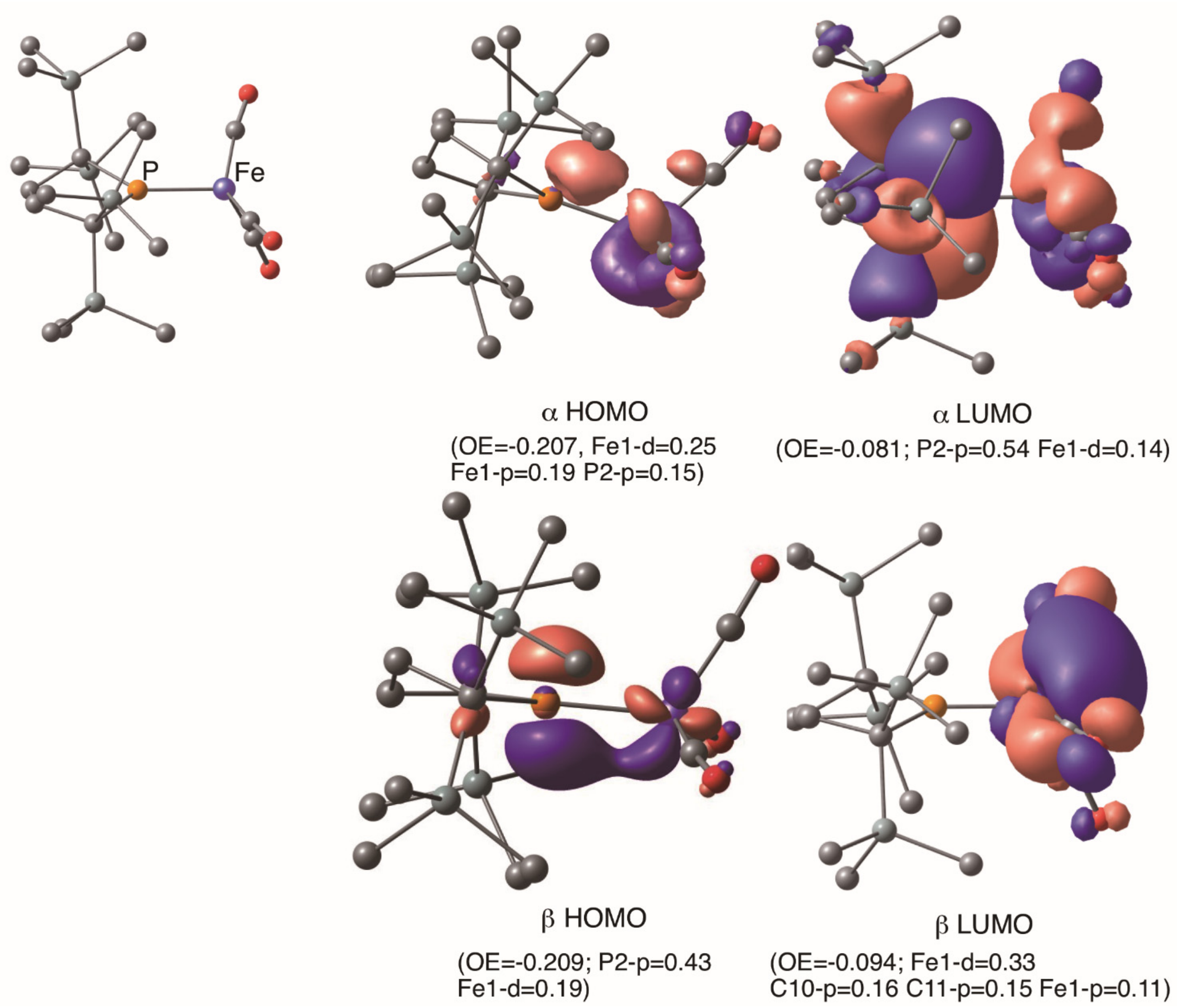

Figure S20. Orbital interactions between the P ligand and the $Fe(CO)_3$ moiety in $\mathbf{3_{opt}}$.

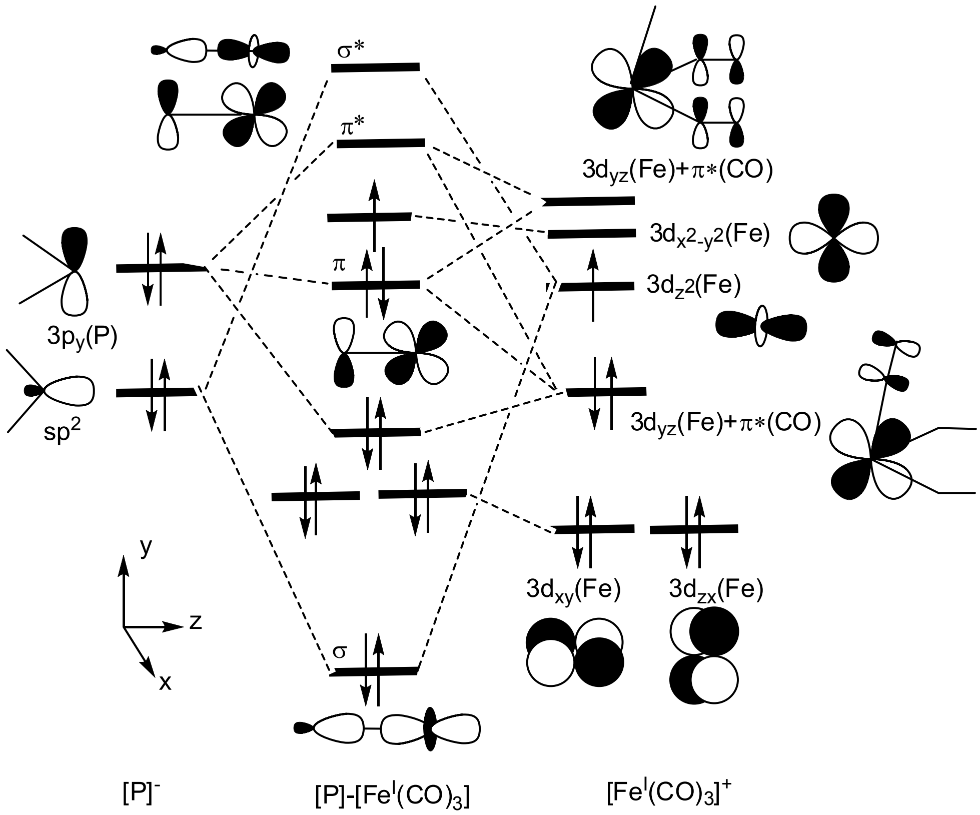

Figure S21. Density distributions in (a) αHOMO and (b) βHOMO of $\mathbf{3_{opt}}$. The plot plane for βHOMO indicates the π-bonding interaction between Fe and P atoms, whereas the plot plane for αHOMO indicates the non-bonding interaction between Fe and P atoms.

(a) αHOMO (b) βHOMO

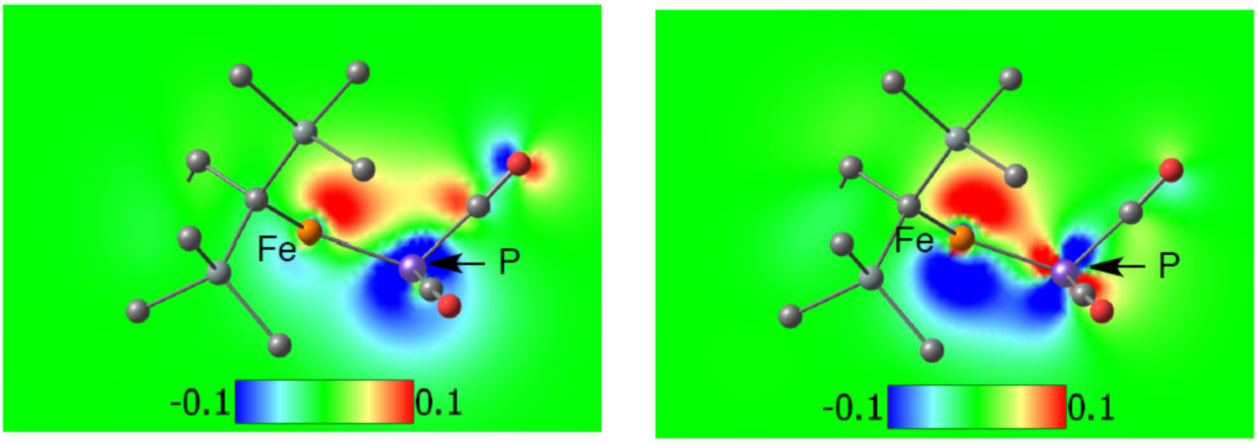

Figure S22. Calculated electron density difference map that details the nature of the transition in **2_{opt}** (red: electron density loss in transition, purple: electron density gain in transition).

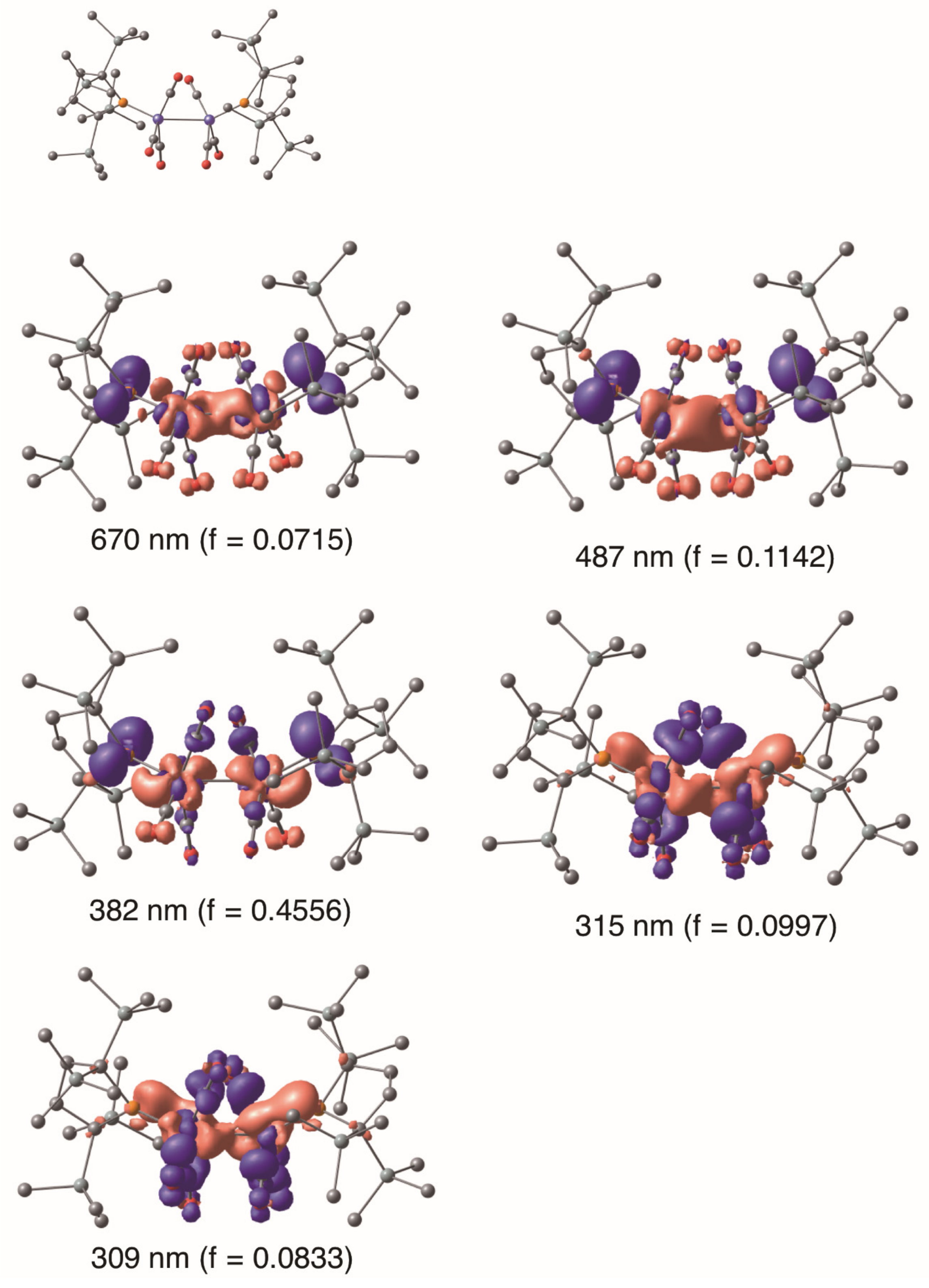

Figure S23. Calculated electron density difference map that details the nature of the transition in $\mathbf{3}_{\mathbf{opt}}$ (red: electron density loss in transition, purple: electron density gain in transition).

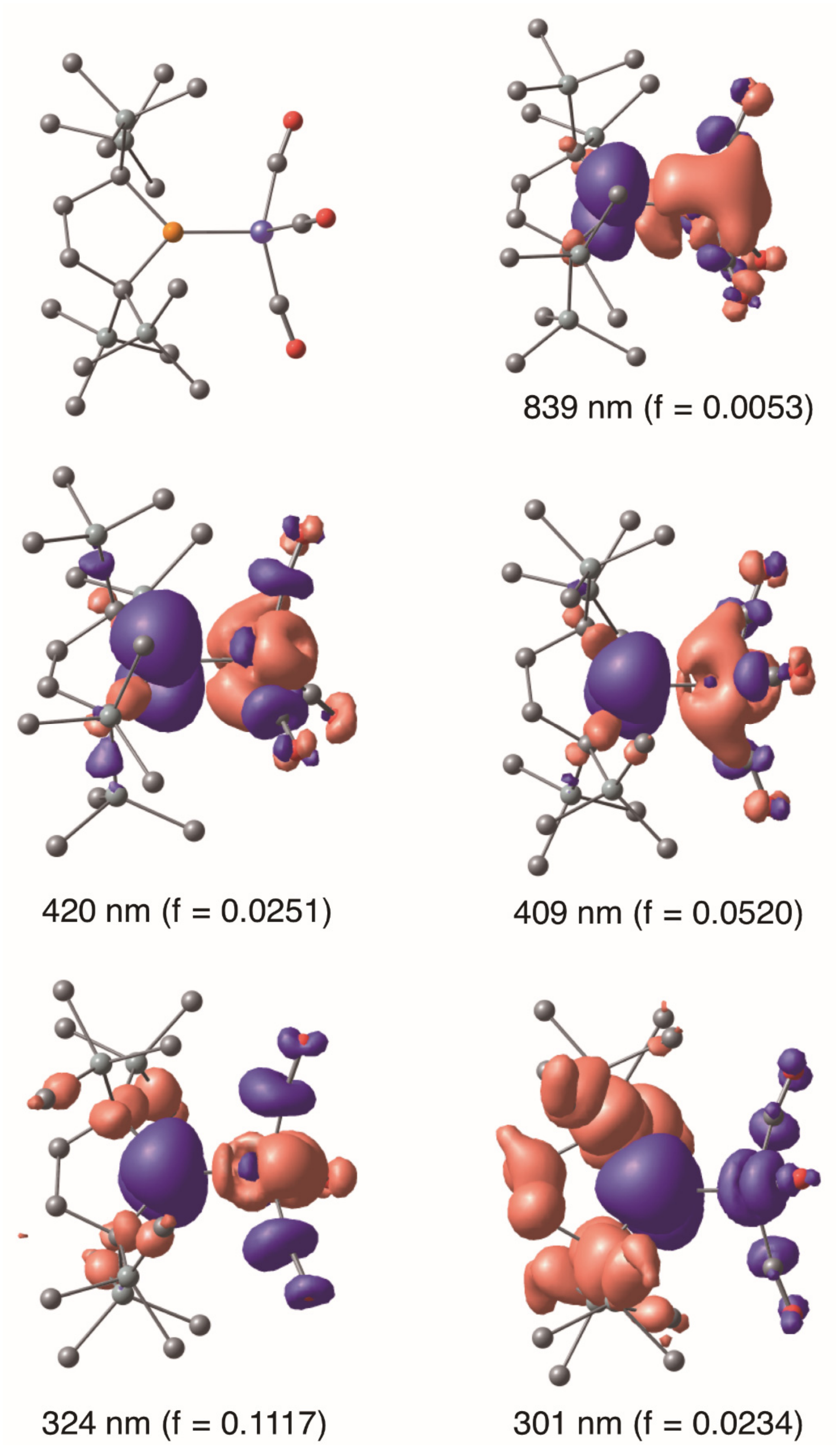

Table S5. Transition Energies, Wavelengths, and Oscillator Strengths of the Electronic Transitions of **2**$_{\mathbf{opt}}$.

```
Excited State   1:      Singlet-A      1.8495 eV  670.36 nm  f=0.0715  <S**2>=0.000
     269 -> 276         0.17716
     270 -> 277        -0.10083
     271 -> 276         0.10795
     272 -> 277         0.20560
     273 -> 276         0.35188
     274 -> 277        -0.24540
     275 -> 276         0.42736
 This state for optimization and/or second-order correction.
 Total Energy, E(TD-HF/TD-KS) =  -7470.12262527
 Copying the excited state density for this state as the 1-particle RhoCI density.

 Excited State   2:      Singlet-A      1.9342 eV  641.01 nm  f=0.0036  <S**2>=0.000
     269 -> 277        -0.17654
     272 -> 276        -0.32306
     273 -> 277        -0.29809
     274 -> 276         0.39734
     275 -> 277        -0.26362

 Excited State   3:      Singlet-A      2.3982 eV  516.98 nm  f=0.0299  <S**2>=0.000
     271 -> 276         0.13923
     272 -> 277        -0.15330
     273 -> 276        -0.26572
     274 -> 277         0.37730
     275 -> 276         0.44880

 Excited State   4:      Singlet-A      2.4348 eV  509.22 nm  f=0.0003  <S**2>=0.000
     270 -> 276        -0.21741
     271 -> 277         0.21031
     272 -> 276        -0.11767
     273 -> 277         0.17162
     274 -> 276         0.38281
     275 -> 277         0.41376

 Excited State   5:      Singlet-A      2.5456 eV  487.06 nm  f=0.1142  <S**2>=0.000
     269 -> 276        -0.14827
     270 -> 277        -0.12527
     271 -> 276         0.17476
     272 -> 277        -0.16238
     273 -> 276         0.47876
     274 -> 277         0.30359
     275 -> 276        -0.16528

 Excited State   6:      Singlet-A      2.5667 eV  483.05 nm  f=0.0063  <S**2>=0.000
     269 -> 277        -0.14402
     272 -> 276        -0.11196
     273 -> 277         0.53839
     275 -> 277        -0.35940

 Excited State   7:      Singlet-A      2.9050 eV  426.79 nm  f=0.0001  <S**2>=0.000
     270 -> 276         0.34883
     271 -> 276        -0.16970
     271 -> 277        -0.31978
     272 -> 276         0.19508
     273 -> 277         0.20860
     274 -> 276         0.27086
     274 -> 278         0.14172
     275 -> 277         0.12368

 Excited State   8:      Singlet-A      2.9486 eV  420.48 nm  f=0.0233  <S**2>=0.000
     270 -> 276         0.20831
     270 -> 277        -0.31997
     271 -> 276         0.35969
     271 -> 277         0.10266
     273 -> 276        -0.19876
     274 -> 277        -0.29510
```

275 -> 276 -0.11047

Excited State 9: Singlet-A 3.0919 eV 400.99 nm f=0.0005 <S**2>=0.000
271 -> 278 0.13676
273 -> 278 0.27497
274 -> 277 -0.13201
275 -> 276 -0.10385
275 -> 278 0.55362

Excited State 10: Singlet-A 3.2167 eV 385.44 nm f=0.0038 <S**2>=0.000
272 -> 278 -0.21536
273 -> 277 -0.12106
274 -> 276 -0.17064
274 -> 278 0.57185
275 -> 279 -0.10300

Excited State 11: Singlet-A 3.2422 eV 382.41 nm f=0.4556 <S**2>=0.000
265 -> 276 -0.12274
269 -> 276 0.26541
270 -> 277 -0.13013
271 -> 276 0.11530
272 -> 277 0.40401
273 -> 278 0.10593
274 -> 277 0.28292
274 -> 279 -0.10630
275 -> 276 -0.21054
275 -> 281 -0.11767

Excited State 12: Singlet-A 3.3195 eV 373.50 nm f=0.0040 <S**2>=0.000
263 -> 277 0.13333
264 -> 276 0.10894
265 -> 277 0.36711
266 -> 276 0.47435
272 -> 276 -0.15405

Excited State 13: Singlet-A 3.3476 eV 370.37 nm f=0.0267 <S**2>=0.000
263 -> 276 0.12898
264 -> 277 0.10310
265 -> 276 0.39087
266 -> 277 0.43224
269 -> 276 0.15069
271 -> 276 0.13182
275 -> 276 -0.10109

Excited State 14: Singlet-A 3.3772 eV 367.12 nm f=0.0230 <S**2>=0.000
266 -> 276 0.14955
269 -> 277 0.20028
270 -> 276 -0.12833
271 -> 277 0.15532
272 -> 276 0.38854
274 -> 276 0.25320
275 -> 277 -0.28565
275 -> 279 0.16509

Excited State 15: Singlet-A 3.6312 eV 341.44 nm f=0.0326 <S**2>=0.000
269 -> 278 -0.14023
270 -> 277 0.25252
271 -> 276 0.19668
271 -> 277 -0.20196
271 -> 278 0.32404
272 -> 277 0.12423
273 -> 278 0.21153
274 -> 279 0.13863
275 -> 278 -0.22206

Excited State 16: Singlet-A 3.6502 eV 339.66 nm f=0.0040 <S**2>=0.000
270 -> 276 0.17186
270 -> 277 0.20829
270 -> 278 0.32913
271 -> 277 0.29380
272 -> 278 0.19888

```
274 -> 278         0.17850
275 -> 279        -0.19358

Excited State  17:      Singlet-A      3.7743 eV  328.50 nm  f=0.0635  <S**2>=0.000
269 -> 276         0.37776
269 -> 278         0.19021
272 -> 277        -0.33966
272 -> 279         0.11434
273 -> 278         0.27994
275 -> 278        -0.22387

Excited State  18:      Singlet-A      3.7963 eV  326.59 nm  f=0.0018  <S**2>=0.000
269 -> 277         0.41509
270 -> 276         0.15549
270 -> 278         0.15312
271 -> 277         0.16007
272 -> 276        -0.22985
272 -> 278        -0.29763
274 -> 278        -0.13532

Excited State  19:      Singlet-A      3.9119 eV  316.94 nm  f=0.0085  <S**2>=0.000
273 -> 280         0.17259
274 -> 282         0.25820
275 -> 280         0.56138

Excited State  20:      Singlet-A      3.9315 eV  315.36 nm  f=0.0997  <S**2>=0.000
269 -> 276         0.11155
271 -> 278         0.19439
272 -> 277        -0.14621
272 -> 280        -0.10782
273 -> 278        -0.31736
273 -> 282         0.12019
274 -> 280         0.33656
274 -> 284        -0.11656
275 -> 281        -0.24173
275 -> 282         0.16910

Excited State  21:      Singlet-A      4.0122 eV  309.02 nm  f=0.0833  <S**2>=0.000
269 -> 276        -0.13528
270 -> 277        -0.16541
271 -> 278        -0.14896
273 -> 278         0.25843
273 -> 282         0.12646
274 -> 280         0.39086
275 -> 281         0.14121
275 -> 282         0.27986

Excited State  22:      Singlet-A      4.0299 eV  307.66 nm  f=0.0051  <S**2>=0.000
270 -> 276         0.20087
272 -> 276        -0.13654
274 -> 278         0.18653
274 -> 281         0.16726
275 -> 279         0.54601

Excited State  23:      Singlet-A      4.0638 eV  305.10 nm  f=0.0029  <S**2>=0.000
269 -> 276         0.14283
270 -> 277        -0.27065
271 -> 276        -0.24580
271 -> 277         0.11884
271 -> 278         0.27808
274 -> 279         0.38551
275 -> 281         0.17067

Excited State  24:      Singlet-A      4.1474 eV  298.95 nm  f=0.0001  <S**2>=0.000
269 -> 277         0.14800
270 -> 276        -0.24838
270 -> 277        -0.13796
270 -> 278         0.26498
271 -> 276         0.10839
271 -> 277        -0.26276
271 -> 278        -0.11842
```

271 -> 279 -0.11436
272 -> 276 -0.13936
272 -> 278 0.16913
273 -> 280 0.20187
274 -> 281 0.19945
274 -> 282 0.10031

Excited State 25: Singlet-A 4.1600 eV 298.04 nm f=0.0013 <S**2>=0.000
265 -> 277 0.15520
266 -> 276 -0.19481
266 -> 278 -0.25410
269 -> 277 0.25247
270 -> 278 -0.20478
272 -> 276 -0.11710
272 -> 278 0.27189
273 -> 280 0.16075
275 -> 280 -0.11864

Excited State 26: Singlet-A 4.2054 eV 294.82 nm f=0.0035 <S**2>=0.000
265 -> 277 0.10049
266 -> 278 -0.14427
269 -> 277 -0.18832
272 -> 276 0.11623
272 -> 278 -0.29457
273 -> 280 0.42939
274 -> 282 0.11015
275 -> 279 0.12108
275 -> 280 -0.23948

Excited State 27: Singlet-A 4.2207 eV 293.75 nm f=0.0118 <S**2>=0.000
269 -> 276 0.19005
269 -> 278 -0.14005
270 -> 277 0.16806
271 -> 276 0.20059
271 -> 278 -0.19173
273 -> 281 0.11076
274 -> 279 0.20273
274 -> 284 -0.10977
275 -> 278 0.18601
275 -> 281 0.32145

Excited State 28: Singlet-A 4.2793 eV 289.73 nm f=0.0013 <S**2>=0.000
265 -> 276 0.10401
265 -> 277 -0.19960
266 -> 276 0.23779
266 -> 278 0.26285
270 -> 278 -0.15947
271 -> 280 0.10026
273 -> 280 0.26482
275 -> 279 -0.11902
275 -> 284 -0.19898

Excited State 29: Singlet-A 4.2935 eV 288.77 nm f=0.0013 <S**2>=0.000
263 -> 278 0.10276
265 -> 276 0.34563
265 -> 278 0.28127
266 -> 277 -0.25893
269 -> 278 0.13223
275 -> 282 -0.13464

Excited State 30: Singlet-A 4.3159 eV 287.27 nm f=0.0276 <S**2>=0.000
268 -> 276 0.51658
268 -> 277 -0.39691

Excited State 31: Singlet-A 4.3376 eV 285.84 nm f=0.0175 <S**2>=0.000
267 -> 276 0.32051
267 -> 277 0.30019
268 -> 276 -0.11686
269 -> 276 0.12507
269 -> 278 -0.25337
271 -> 276 -0.11494

272 -> 279 -0.13200
274 -> 281 -0.10582
275 -> 282 0.14784
275 -> 284 0.13337

Excited State 32: Singlet-A 4.3526 eV 284.85 nm f=0.0220 <S**2>=0.000
266 -> 277 0.10439
267 -> 276 0.33049
267 -> 277 0.22784
268 -> 277 -0.13101
269 -> 278 0.28043
270 -> 277 0.10491
272 -> 277 0.11140
272 -> 279 0.14642
274 -> 280 0.10503
275 -> 281 0.10371
275 -> 283 -0.12526

Excited State 33: Singlet-A 4.3650 eV 284.04 nm f=0.0250 <S**2>=0.000
267 -> 276 -0.21939
267 -> 277 -0.21769
268 -> 276 0.10892
273 -> 279 0.24693
273 -> 280 0.16608
274 -> 279 -0.11927
274 -> 281 -0.23758
274 -> 284 -0.11699
275 -> 281 0.13830
275 -> 284 0.30775

Excited State 34: Singlet-A 4.3799 eV 283.08 nm f=0.0029 <S**2>=0.000
265 -> 276 -0.14182
266 -> 277 0.10261
271 -> 278 0.10505
272 -> 280 -0.12249
274 -> 279 -0.29764
274 -> 281 0.17970
274 -> 284 -0.25491
275 -> 281 0.30499
275 -> 284 -0.13136
275 -> 287 -0.17094

Excited State 35: Singlet-A 4.4081 eV 281.27 nm f=0.0044 <S**2>=0.000
273 -> 279 0.51461
274 -> 281 0.34645
275 -> 279 -0.12367

Excited State 36: Singlet-A 4.4820 eV 276.63 nm f=0.0003 <S**2>=0.000
269 -> 278 0.16326
272 -> 279 0.12603
273 -> 281 0.12025
273 -> 282 -0.13818
273 -> 283 0.10773
274 -> 279 0.11932
274 -> 280 -0.24326
275 -> 282 0.44463
275 -> 283 0.12700

Excited State 37: Singlet-A 4.5007 eV 275.48 nm f=0.0004 <S**2>=0.000
271 -> 280 -0.11651
272 -> 282 -0.11503
273 -> 280 -0.14682
274 -> 282 0.44641
274 -> 283 0.26250
274 -> 286 0.10686
275 -> 280 -0.13052
275 -> 282 -0.10021
275 -> 284 0.12378
275 -> 289 0.14701

Excited State 38: Singlet-A 4.5575 eV 272.05 nm f=0.0019 <S**2>=0.000

```
      272 -> 280         -0.18066
      273 -> 282          0.47555
      274 -> 279          0.16315
      274 -> 289          0.10214
      275 -> 281         -0.13023
      275 -> 282         -0.17397
      275 -> 283          0.24242

 Excited State  39:      Singlet-A      4.5736 eV  271.09 nm  f=0.0058  <S**2>=0.000
      269 -> 278         -0.10826
      270 -> 280         -0.16933
      271 -> 282         -0.12781
      273 -> 281          0.11127
      273 -> 282         -0.15123
      273 -> 283          0.12558
      274 -> 280          0.20180
      274 -> 284          0.14728
      275 -> 281          0.11056
      275 -> 283          0.38836
      275 -> 286          0.14954

 Excited State  40:      Singlet-A      4.6099 eV  268.95 nm  f=0.0030  <S**2>=0.000
      269 -> 280         -0.11886
      270 -> 278         -0.12732
      272 -> 282          0.11495
      273 -> 279         -0.25863
      274 -> 281          0.33012
      275 -> 279         -0.16687
      275 -> 284          0.39021

 Excited State  41:      Singlet-A      4.6800 eV  264.92 nm  f=0.0009  <S**2>=0.000
      271 -> 278          0.10957
      273 -> 281          0.21661
      273 -> 282          0.19789
      274 -> 279         -0.22026
      274 -> 280         -0.11767
      274 -> 284          0.30925
      275 -> 281          0.15633
      275 -> 287          0.18004
      275 -> 288         -0.14974

 Excited State  42:      Singlet-A      4.7036 eV  263.60 nm  f=0.0043  <S**2>=0.000
      263 -> 276          0.11450
      265 -> 277         -0.10065
      265 -> 278         -0.15681
      266 -> 277         -0.14146
      272 -> 280         -0.19162
      273 -> 281          0.40854
      273 -> 283          0.14034
      273 -> 290         -0.12545
      275 -> 283         -0.12294

 Excited State  43:      Singlet-A      4.7126 eV  263.09 nm  f=0.0019  <S**2>=0.000
      264 -> 276         -0.13441
      265 -> 277          0.35222
      266 -> 276         -0.19577
      266 -> 278          0.28212
      269 -> 277          0.10649
      274 -> 290          0.10996
      275 -> 287         -0.12788
      275 -> 288         -0.11115

 Excited State  44:      Singlet-A      4.7297 eV  262.14 nm  f=0.0003  <S**2>=0.000
      265 -> 277         -0.10388
      266 -> 278         -0.13658
      270 -> 282          0.10902
      271 -> 280          0.18414
      274 -> 281         -0.14031
      274 -> 282         -0.19483
      274 -> 283          0.40519
      274 -> 286          0.13311
```

275 -> 280 0.11218
275 -> 289 0.12398

Excited State 45: Singlet-A 4.7506 eV 260.99 nm f=0.0048 <S**2>=0.000
262 -> 276 -0.11574
263 -> 276 0.29002
264 -> 276 -0.23589
264 -> 277 0.25969
265 -> 278 -0.15943
266 -> 277 -0.24159
273 -> 281 -0.27223

Excited State 46: Singlet-A 4.7595 eV 260.50 nm f=0.0038 <S**2>=0.000
261 -> 276 -0.11934
262 -> 277 -0.10645
263 -> 276 0.23872
263 -> 277 0.36727
264 -> 276 0.34155
266 -> 276 -0.22674
266 -> 278 0.15633
268 -> 277 0.10354

Excited State 47: Singlet-A 4.7728 eV 259.77 nm f=0.0011 <S**2>=0.000
264 -> 277 0.10067
265 -> 276 -0.12144
265 -> 278 0.12091
267 -> 276 0.12502
268 -> 276 0.36187
268 -> 277 0.47615
273 -> 281 0.10520

Excited State 48: Singlet-A 4.7799 eV 259.39 nm f=0.0006 <S**2>=0.000
263 -> 276 -0.14427
264 -> 276 0.14390
264 -> 277 -0.18997
265 -> 276 0.23789
265 -> 278 -0.18742
267 -> 277 0.18742
268 -> 276 0.17972
268 -> 277 0.17215
272 -> 280 -0.18045
273 -> 281 -0.12732
275 -> 288 -0.10344

Excited State 49: Singlet-A 4.7879 eV 258.96 nm f=0.0037 <S**2>=0.000
245 -> 277 0.10428
253 -> 276 0.12061
253 -> 277 0.12330
263 -> 276 -0.15470
263 -> 277 -0.11360
265 -> 276 0.13161
265 -> 277 0.16465
265 -> 278 -0.11819
268 -> 277 0.16960
274 -> 282 -0.11128
274 -> 283 0.21471
274 -> 287 -0.10819
274 -> 290 -0.12064

Excited State 50: Singlet-A 4.7917 eV 258.75 nm f=0.0009 <S**2>=0.000
267 -> 276 -0.41474
267 -> 277 0.48664

Excited State 51: Singlet-A 4.8124 eV 257.63 nm f=0.0168 <S**2>=0.000
245 -> 276 0.10745
254 -> 277 -0.11235
265 -> 278 -0.10605
272 -> 279 -0.10930
272 -> 280 0.20563
273 -> 278 0.10673
273 -> 281 0.22844

273 -> 290 0.14766
274 -> 280 0.13912
274 -> 284 -0.14644
274 -> 288 -0.15692
275 -> 282 -0.10793
275 -> 290 0.19491

Excited State 52: Singlet-A 4.8634 eV 254.93 nm f=0.0023 <S**2>=0.000
269 -> 280 0.24551
269 -> 282 0.10193
270 -> 279 0.11407
270 -> 282 0.10139
271 -> 280 0.12176
272 -> 280 -0.17163
272 -> 281 0.11230
272 -> 282 -0.23486
272 -> 283 0.12012
273 -> 282 -0.10730
273 -> 283 -0.18745
274 -> 289 -0.14238
275 -> 290 0.11029

Excited State 53: Singlet-A 4.8663 eV 254.78 nm f=0.0020 <S**2>=0.000
269 -> 280 0.24704
269 -> 282 -0.10596
271 -> 279 0.12165
271 -> 281 -0.10817
272 -> 280 0.18064
272 -> 282 -0.22522
272 -> 283 0.10922
273 -> 282 0.12513
273 -> 283 0.20610
274 -> 289 0.16015
275 -> 290 -0.14223

Excited State 54: Singlet-A 4.9241 eV 251.79 nm f=0.0048 <S**2>=0.000
241 -> 276 0.10695
245 -> 276 0.16966
246 -> 277 0.14335
253 -> 276 0.17782
253 -> 277 0.12370
254 -> 277 -0.16569
257 -> 276 0.10196
265 -> 278 0.14594
266 -> 277 0.11923
269 -> 282 0.10203
272 -> 280 -0.19689
273 -> 283 0.19854
275 -> 283 -0.16182
275 -> 286 -0.10146
275 -> 287 -0.11423

Excited State 55: Singlet-A 4.9469 eV 250.63 nm f=0.0081 <S**2>=0.000
254 -> 276 0.12302
273 -> 284 0.33642
274 -> 282 0.12533
274 -> 290 -0.11942
275 -> 284 -0.12798
275 -> 287 0.23094
275 -> 288 0.27781
275 -> 289 0.13328

Excited State 56: Singlet-A 4.9999 eV 247.98 nm f=0.0039 <S**2>=0.000
254 -> 276 -0.11407
262 -> 276 0.12003
271 -> 280 0.31527
271 -> 282 -0.18278
272 -> 279 -0.12195
273 -> 289 -0.10125
274 -> 282 0.16864
274 -> 290 0.12451

275 -> 283 -0.11541
275 -> 289 0.10172
275 -> 290 0.10866

Excited State 57: Singlet-A 5.0037 eV 247.78 nm f=0.0058 <S**2>=0.000
269 -> 279 -0.18408
272 -> 281 0.22507
273 -> 284 0.33797
273 -> 288 0.10068
274 -> 287 0.16486
274 -> 288 -0.12012
275 -> 284 -0.13422
275 -> 287 -0.18072
275 -> 288 -0.21515

Excited State 58: Singlet-A 5.0127 eV 247.34 nm f=0.0136 <S**2>=0.000
261 -> 276 0.28880
261 -> 277 -0.13541
262 -> 276 -0.21286
262 -> 277 0.23598
264 -> 276 0.15674
270 -> 280 0.16066
270 -> 282 0.11766
271 -> 280 0.13272
272 -> 279 0.16500
274 -> 282 0.11649

Excited State 59: Singlet-A 5.0278 eV 246.60 nm f=0.0126 <S**2>=0.000
261 -> 276 0.19687
261 -> 277 0.32251
262 -> 276 0.41555
262 -> 277 0.16125
263 -> 276 0.12840
272 -> 279 0.17381

Excited State 60: Singlet-A 5.0335 eV 246.32 nm f=0.0016 <S**2>=0.000
261 -> 276 0.30970
262 -> 277 0.23100
264 -> 276 0.11149
270 -> 280 -0.26998
270 -> 282 -0.13997
271 -> 282 -0.16983
272 -> 279 -0.14299
274 -> 280 -0.10326
275 -> 290 0.11245

Excited State 61: Singlet-A 5.0468 eV 245.67 nm f=0.0035 <S**2>=0.000
261 -> 276 -0.11505
269 -> 278 -0.10857
269 -> 281 -0.14598
270 -> 279 -0.19845
270 -> 284 -0.11376
271 -> 281 -0.13630
272 -> 279 0.31351
273 -> 288 0.10606
274 -> 284 0.19718
275 -> 287 -0.11371
275 -> 290 0.20631

Excited State 62: Singlet-A 5.0954 eV 243.32 nm f=0.0246 <S**2>=0.000
273 -> 282 0.13082
273 -> 283 0.28598
273 -> 287 -0.15846
273 -> 288 0.13118
273 -> 290 -0.10486
274 -> 284 -0.22571
274 -> 289 -0.17004
275 -> 286 0.13680
275 -> 287 0.23372
275 -> 288 -0.23230
275 -> 290 0.13722

Excited State 63: Singlet-A 5.1200 eV 242.16 nm f=0.0098 <S**2>=0.000
269 -> 279 0.23390
270 -> 278 0.10383
270 -> 281 0.14288
271 -> 279 0.24284
271 -> 280 -0.10255
272 -> 281 -0.22163
273 -> 284 0.28378
273 -> 289 -0.11282
274 -> 288 0.10279
275 -> 287 -0.12263
275 -> 288 -0.16560
275 -> 289 0.16309

Excited State 64: Singlet-A 5.1744 eV 239.61 nm f=0.0021 <S**2>=0.000
269 -> 279 0.12322
271 -> 280 0.15558
272 -> 281 -0.21275
273 -> 287 0.23984
273 -> 288 0.25297
273 -> 289 0.24197
274 -> 288 -0.12183
274 -> 290 -0.13759
275 -> 287 -0.11536
275 -> 288 -0.11342

Excited State 65: Singlet-A 5.1816 eV 239.28 nm f=0.0032 <S**2>=0.000
257 -> 276 -0.15042
257 -> 277 0.13512
258 -> 276 0.23758
258 -> 277 -0.17059
259 -> 276 0.25862
259 -> 277 -0.21679
260 -> 276 -0.22583
260 -> 277 0.12397
273 -> 288 -0.10487
274 -> 288 -0.21266

Excited State 66: Singlet-A 5.1907 eV 238.86 nm f=0.0037 <S**2>=0.000
259 -> 277 0.11457
260 -> 276 0.15114
269 -> 279 0.10896
271 -> 284 -0.10011
272 -> 281 -0.25960
272 -> 282 -0.18278
274 -> 286 0.10288
274 -> 287 0.32972
274 -> 288 -0.16613
275 -> 289 -0.13480

Excited State 67: Singlet-A 5.1935 eV 238.73 nm f=0.0052 <S**2>=0.000
258 -> 276 0.12233
259 -> 276 0.19006
259 -> 277 -0.17061
260 -> 276 -0.20276
271 -> 279 -0.13422
272 -> 279 0.10622
273 -> 283 -0.18361
273 -> 286 -0.11384
273 -> 290 -0.18362
274 -> 287 0.13459
274 -> 288 0.25401
275 -> 289 0.10830

Excited State 68: Singlet-A 5.2015 eV 238.36 nm f=0.0003 <S**2>=0.000
255 -> 276 0.14928
255 -> 277 0.11519
257 -> 276 -0.18257
257 -> 277 -0.11164
259 -> 276 0.26414

259 -> 277 0.12149
260 -> 276 0.29020
260 -> 277 0.31047
261 -> 276 0.11172
261 -> 277 0.12677
274 -> 287 -0.11747

Excited State 69: Singlet-A 5.2107 eV 237.94 nm f=0.0011 <S**2>=0.000
259 -> 276 0.12752
260 -> 276 0.14000
260 -> 277 0.14595
270 -> 279 0.11250
270 -> 281 0.15656
271 -> 279 0.22771
272 -> 282 0.14756
273 -> 284 -0.13604
273 -> 290 -0.10326
274 -> 282 0.10812
274 -> 287 0.19372
275 -> 284 -0.10559
275 -> 291 0.14256

Excited State 70: Singlet-A 5.2270 eV 237.20 nm f=0.0005 <S**2>=0.000
269 -> 278 0.13331
270 -> 279 -0.15755
270 -> 280 0.20198
271 -> 281 -0.11431
271 -> 283 -0.11532
272 -> 279 -0.18859
272 -> 280 -0.10350
273 -> 282 -0.19640
273 -> 286 -0.10664
273 -> 287 -0.15983
273 -> 288 0.15854
273 -> 290 -0.12669
274 -> 289 0.18105
275 -> 287 0.19022
275 -> 288 -0.14198

Excited State 71: Singlet-A 5.2481 eV 236.25 nm f=0.0027 <S**2>=0.000
273 -> 284 -0.13011
274 -> 283 -0.12430
274 -> 286 0.10453
274 -> 287 0.17167
274 -> 288 -0.16115
275 -> 285 0.36713
275 -> 289 0.41999

Excited State 72: Singlet-A 5.2594 eV 235.74 nm f=0.0018 <S**2>=0.000
261 -> 277 -0.16045
262 -> 276 0.10607
263 -> 276 -0.36788
263 -> 277 0.20938
264 -> 276 0.15882
264 -> 277 0.43420
265 -> 276 0.11583
266 -> 277 -0.11026

Excited State 73: Singlet-A 5.2609 eV 235.67 nm f=0.0001 <S**2>=0.000
261 -> 276 0.12199
262 -> 277 -0.14322
263 -> 276 -0.18369
263 -> 277 0.42651
264 -> 276 -0.34802
264 -> 277 -0.22264
265 -> 277 -0.15697

Excited State 74: Singlet-A 5.2834 eV 234.67 nm f=0.0030 <S**2>=0.000
258 -> 276 0.34969
258 -> 277 -0.28332
259 -> 276 -0.22368

259 -> 277 0.13037
274 -> 287 0.11050
275 -> 285 -0.29752
275 -> 289 0.10850

Excited State 75: Singlet-A 5.2866 eV 234.52 nm f=0.0009 <S**2>=0.000
257 -> 276 0.10112
258 -> 276 0.31619
258 -> 277 -0.15296
259 -> 277 0.15903
260 -> 276 0.12605
274 -> 287 -0.10693
275 -> 285 0.35419
275 -> 286 -0.11676
275 -> 289 -0.15310
275 -> 290 -0.10299

Excited State 76: Singlet-A 5.2934 eV 234.22 nm f=0.0012 <S**2>=0.000
256 -> 276 -0.20856
256 -> 277 -0.14523
257 -> 276 -0.22086
257 -> 277 -0.19259
258 -> 277 -0.13695
259 -> 276 -0.16419
259 -> 277 -0.10038
260 -> 276 -0.15481
260 -> 277 -0.12665
262 -> 276 -0.15139
262 -> 277 -0.12646
264 -> 277 0.11236
275 -> 285 0.25419
275 -> 289 -0.13195

Excited State 77: Singlet-A 5.3099 eV 233.49 nm f=0.0008 <S**2>=0.000
255 -> 277 -0.12995
257 -> 276 0.17324
270 -> 280 0.11890
273 -> 283 0.10986
273 -> 287 0.13841
274 -> 288 0.13025
275 -> 285 0.18227
275 -> 286 0.30324
275 -> 288 0.10171
275 -> 289 -0.13745
275 -> 290 0.23580

Excited State 78: Singlet-A 5.3136 eV 233.33 nm f=0.0042 <S**2>=0.000
254 -> 276 -0.19768
254 -> 277 0.14531
255 -> 276 0.30765
255 -> 277 -0.20985
256 -> 276 -0.18182
256 -> 277 0.12590
257 -> 276 0.26307
257 -> 277 -0.20435
273 -> 290 0.10511

Excited State 79: Singlet-A 5.3289 eV 232.67 nm f=0.0011 <S**2>=0.000
253 -> 276 0.10977
255 -> 276 0.34590
255 -> 277 0.25098
256 -> 276 0.32462
256 -> 277 0.27773
275 -> 286 0.13160

Excited State 80: Singlet-A 5.3535 eV 231.60 nm f=0.0119 <S**2>=0.000
270 -> 279 -0.11652
273 -> 290 0.30581
274 -> 285 0.18477
274 -> 287 0.12209
274 -> 288 0.14899

274 -> 290 -0.12786
275 -> 283 -0.11926
275 -> 286 0.31174
275 -> 290 -0.18624

Excited State 81: Singlet-A 5.3606 eV 231.29 nm f=0.0014 <S**2>=0.000
266 -> 279 -0.10926
270 -> 279 0.16128
271 -> 281 0.11099
272 -> 280 0.10098
273 -> 283 -0.10642
273 -> 288 0.13414
273 -> 290 -0.10173
274 -> 285 0.20423
274 -> 287 -0.17679
274 -> 288 -0.18627
274 -> 289 0.17920
275 -> 283 -0.15810
275 -> 286 0.23045
275 -> 290 -0.18791

Excited State 82: Singlet-A 5.3783 eV 230.53 nm f=0.0075 <S**2>=0.000
261 -> 276 -0.16716
262 -> 277 0.17490
273 -> 287 0.17027
273 -> 288 0.15512
273 -> 290 0.13376
274 -> 286 0.16827
274 -> 289 0.13545
274 -> 290 0.37135
275 -> 287 0.16598
275 -> 291 -0.14036

Excited State 83: Singlet-A 5.3830 eV 230.33 nm f=0.0019 <S**2>=0.000
261 -> 276 -0.30703
261 -> 277 0.24255
262 -> 276 -0.19107
262 -> 277 0.39778
263 -> 277 0.14767
274 -> 290 -0.14426

Excited State 84: Singlet-A 5.3893 eV 230.06 nm f=0.0010 <S**2>=0.000
253 -> 276 -0.10175
256 -> 276 0.22324
256 -> 277 0.17014
257 -> 276 0.12628
261 -> 276 0.12029
261 -> 277 0.40487
262 -> 276 -0.27922
262 -> 277 -0.17199
264 -> 277 0.12946

Excited State 85: Singlet-A 5.4256 eV 228.52 nm f=0.0121 <S**2>=0.000
249 -> 276 0.11500
249 -> 277 -0.15190
250 -> 276 0.26644
251 -> 276 -0.16626
251 -> 277 0.23521
252 -> 276 0.12676
254 -> 276 -0.25686
257 -> 276 -0.19801
257 -> 277 0.14721
260 -> 276 0.11206
273 -> 289 0.12403
274 -> 289 0.16251

Excited State 86: Singlet-A 5.4315 eV 228.27 nm f=0.0130 <S**2>=0.000
250 -> 276 -0.15300
251 -> 276 0.10871
251 -> 277 -0.10286
270 -> 279 0.11900

273 -> 289 0.12266
273 -> 290 0.15148
274 -> 285 -0.11647
274 -> 289 0.39783
274 -> 290 -0.14237
275 -> 286 -0.11130
275 -> 288 -0.13230
275 -> 290 0.17636

Excited State 87: Singlet-A 5.4382 eV 227.99 nm f=0.0166 <S**2>=0.000
250 -> 276 -0.18714
250 -> 277 0.26573
251 -> 276 0.18955
252 -> 276 0.45393
252 -> 277 -0.25844

Excited State 88: Singlet-A 5.4432 eV 227.78 nm f=0.0193 <S**2>=0.000
250 -> 276 0.21625
250 -> 277 0.14472
251 -> 276 0.29118
251 -> 277 0.13374
252 -> 276 -0.12580
252 -> 277 0.22409
253 -> 276 0.11617
254 -> 277 -0.13781
273 -> 287 -0.11735
273 -> 289 0.24378

Excited State 89: Singlet-A 5.4484 eV 227.56 nm f=0.0023 <S**2>=0.000
249 -> 276 0.17469
250 -> 276 -0.13941
250 -> 277 -0.14084
251 -> 276 -0.19326
252 -> 277 -0.10594
254 -> 277 0.14992
256 -> 276 -0.12115
261 -> 277 0.11879
273 -> 287 -0.15978
273 -> 289 0.28567
274 -> 291 0.11681

Excited State 90: Singlet-A 5.4637 eV 226.92 nm f=0.0299 <S**2>=0.000
249 -> 276 0.36392
249 -> 277 0.24320
251 -> 276 0.16761
251 -> 277 0.18549
253 -> 276 0.23060
253 -> 277 0.20055
273 -> 287 0.11579

Excited State 91: Singlet-A 5.4692 eV 226.70 nm f=0.0207 <S**2>=0.000
270 -> 279 0.14125
271 -> 278 0.10714
271 -> 281 0.12915
272 -> 284 0.12470
273 -> 288 0.17598
273 -> 289 -0.16820
273 -> 290 0.19346
274 -> 291 0.30948
275 -> 292 0.26924

Excited State 92: Singlet-A 5.5047 eV 225.23 nm f=0.0011 <S**2>=0.000
272 -> 284 -0.17194
272 -> 289 0.10081
274 -> 285 0.46888
274 -> 291 0.12931
275 -> 286 -0.22151
275 -> 291 -0.13880

Excited State 93: Singlet-A 5.5130 eV 224.89 nm f=0.0312 <S**2>=0.000
266 -> 279 -0.10013

```
      269 -> 279         -0.14087
      272 -> 283         -0.12508
      272 -> 284         -0.18004
      272 -> 287          0.10840
      272 -> 288         -0.11898
      272 -> 289          0.13106
      273 -> 287         -0.11018
      273 -> 289          0.14910
      274 -> 283         -0.15175
      274 -> 286          0.11770
      275 -> 291          0.27567

Excited State   94:      Singlet-A      5.5234 eV  224.47 nm  f=0.0891  <S**2>=0.000
      265 -> 281         -0.12561
      266 -> 279          0.16496
      266 -> 280         -0.16810
      269 -> 278         -0.12655
      272 -> 284          0.10366
      272 -> 289         -0.10455
      273 -> 287          0.13661
      273 -> 288         -0.16375
      273 -> 289          0.10468
      273 -> 290         -0.13017
      274 -> 284         -0.10304
      274 -> 285          0.23550
      274 -> 286          0.11012
      275 -> 286         -0.13558
      275 -> 288         -0.11202
      275 -> 291          0.16834

Excited State   95:      Singlet-A      5.5362 eV  223.95 nm  f=0.0426  <S**2>=0.000
      271 -> 283          0.12230
      272 -> 284          0.17921
      273 -> 287         -0.14383
      273 -> 288          0.12725
      273 -> 290          0.11106
      273 -> 292         -0.16441
      274 -> 285          0.29606
      274 -> 287          0.10291
      274 -> 291         -0.23682
      275 -> 286         -0.13750
      275 -> 290          0.13646

Excited State   96:      Singlet-A      5.5622 eV  222.90 nm  f=0.0030  <S**2>=0.000
      265 -> 279         -0.18360
      266 -> 281          0.13408
      269 -> 280          0.11288
      269 -> 284         -0.11141
      269 -> 289         -0.12763
      272 -> 281         -0.19873
      272 -> 282          0.15965
      272 -> 283          0.33932
      272 -> 286          0.10808
      272 -> 290         -0.10079
      273 -> 291          0.13949
      274 -> 292          0.11613
      275 -> 291          0.15262

Excited State   97:      Singlet-A      5.5731 eV  222.47 nm  f=0.0000  <S**2>=0.000
      257 -> 276          0.12467
      258 -> 276         -0.14799
      258 -> 277         -0.19761
      259 -> 276         -0.35523
      259 -> 277         -0.27235
      260 -> 277          0.41208

Excited State   98:      Singlet-A      5.5802 eV  222.18 nm  f=0.0009  <S**2>=0.000
      249 -> 277         -0.10558
      257 -> 277         -0.10991
      259 -> 276         -0.10225
      259 -> 277          0.40893
```

```
 260 -> 276         -0.38905
 260 -> 277          0.28893

Excited State  99:      Singlet-A      5.5831 eV  222.07 nm  f=0.0005  <S**2>=0.000
 269 -> 279          0.10388
 269 -> 284          0.10308
 273 -> 285          0.13846
 273 -> 291          0.16392
 274 -> 283         -0.18910
 274 -> 286          0.49412
 274 -> 290         -0.14182
 275 -> 285         -0.13025

Excited State 100:      Singlet-A      5.6153 eV  220.80 nm  f=0.0058  <S**2>=0.000
 257 -> 277         -0.17773
 258 -> 276          0.15842
 258 -> 277          0.19369
 269 -> 279          0.11738
 269 -> 284          0.13728
 271 -> 279         -0.15208
 272 -> 287         -0.10330
 273 -> 291          0.22142
 274 -> 286         -0.17008
 274 -> 290          0.12849
 275 -> 291          0.14908
```

Table S6. Transition Energies, Wavelengths, and Oscillator Strengths of the Electronic Transitions of **3**$_{\mathbf{opt}}$.

```
Excited State   1:   2.310-A      0.8409 eV 1474.39 nm  f=0.0012  <S**2>=1.084
   135B ->138B        -0.19341
   136B ->138B         0.65822
   136B ->139B         0.29841
   136B ->140B         0.15795
   136B ->144B         0.14877
   137B ->138B        -0.52334
   137B ->139B        -0.18819
   137B ->140B        -0.11012
 This state for optimization and/or second-order correction.
 Total Energy, E(TD-HF/TD-KS) =  -3735.04705016
 Copying the excited state density for this state as the 1-particle RhoCI density.

 Excited State   2:   2.304-A      1.0107 eV 1226.73 nm  f=0.0031  <S**2>=1.077
   135B ->138B         0.82148
   135B ->139B         0.23357
   135B ->140B         0.21630
   135B ->144B         0.15750
   135B ->146B         0.10672
   135B ->147B         0.12312
   136B ->138B         0.30466
   136B ->139B         0.10165

 Excited State   3:   2.877-A      1.4777 eV  839.04 nm  f=0.0053  <S**2>=1.820
   137A ->139A        -0.18898
   138A ->139A         0.78592
   137B ->139B        -0.56220
   137B ->146B         0.10009
   138A <-139A         0.12942
   137B <-139B        -0.14385

 Excited State   4:   2.596-A      1.7589 eV  704.92 nm  f=0.0018  <S**2>=1.435
   137A ->139A        -0.10377
   138A ->139A        -0.20185
   134B ->138B        -0.39745
   135B ->138B        -0.15461
```

```
      135B ->139B        -0.13125
      136B ->139B         0.48818
      136B ->144B         0.10028
      136B ->146B        -0.12955
      137B ->138B         0.60336
      137B ->139B        -0.15758

 Excited State   5:      2.447-A      1.8418 eV  673.17 nm  f=0.0010  <S**2>=1.247
      138A ->139A        -0.14528
      132B ->138B         0.10951
      134B ->138B         0.81636
      134B ->139B         0.14142
      134B ->140B         0.14868
      134B ->144B         0.12004
      134B ->146B         0.10275
      135B ->139B        -0.11455
      136B ->139B         0.28196
      137B ->138B         0.22008
      137B ->139B        -0.11276

 Excited State   6:      2.609-A      2.0147 eV  615.41 nm  f=0.0042  <S**2>=1.452
      137A ->139A         0.22649
      138A ->139A         0.24383
      134B ->138B         0.12971
      135B ->138B        -0.19315
      136B ->138B         0.55354
      136B ->139B        -0.37492
      136B ->146B         0.16802
      137B ->138B         0.51736
      137B ->139B         0.18578

 Excited State   7:      3.176-A      2.1324 eV  581.44 nm  f=0.0009  <S**2>=2.272
      136A ->139A        -0.30769
      135B ->138B        -0.25005
      135B ->139B         0.79069
      135B ->146B        -0.22819
      136B ->139B         0.21772

 Excited State   8:      2.406-A      2.4568 eV  504.66 nm  f=0.0013  <S**2>=1.197
      137A ->139A         0.83528
      137A ->140A        -0.10069
      137A ->145A         0.10417
      138A ->139A         0.24949
      135B ->139B        -0.13206
      136B ->138B        -0.17804
      136B ->139B         0.33282

 Excited State   9:      3.092-A      2.6460 eV  468.57 nm  f=0.0021  <S**2>=2.140
      132A ->139A        -0.13372
      134A ->139A         0.13259
      132B ->139B         0.10644
      134B ->138B        -0.13964
      134B ->139B         0.85759
      134B ->144B         0.12821
      134B ->146B        -0.27875

 Excited State  10:      2.792-A      2.8578 eV  433.85 nm  f=0.0071  <S**2>=1.699
      136A ->139A        -0.22104
      137A ->139A         0.12817
      137A ->140A         0.13599
      138A ->140A         0.69342
      138A ->145A        -0.14163
      134B ->141B         0.18683
      135B ->143B        -0.13046
      136B ->139B        -0.18336
      136B ->140B        -0.25853
      137B ->139B        -0.35284
      137B ->140B         0.11441

 Excited State  11:      2.615-A      2.9462 eV  420.83 nm  f=0.0251  <S**2>=1.459
      132A ->139A        -0.16174
```

```
      133A ->139A        -0.17145
      136A ->139A         0.66777
      136A ->143A         0.10924
      137A ->139A        -0.19403
      138A ->139A         0.23653
      138A ->140A         0.33793
      135B ->139B         0.17891
      136B ->139B         0.13924
      136B ->140B        -0.13020
      137B ->139B         0.29083

Excited State  12:   2.500-A    3.0322 eV  408.89 nm  f=0.0520  <S**2>=1.312
      131A ->139A         0.21161
      132A ->139A         0.13170
      133A ->139A         0.27695
      136A ->139A         0.54305
      137A ->139A         0.22368
      138A ->139A        -0.30651
      138A ->140A        -0.13649
      135B ->139B         0.27854
      136B ->140B         0.10726
      137B ->139B        -0.43883

Excited State  13:   3.353-A    3.2276 eV  384.13 nm  f=0.0066  <S**2>=2.561
      132A ->141A         0.12211
      134A ->141A        -0.12073
      137A ->139A        -0.11491
      137A ->140A        -0.47496
      137A ->142A        -0.13303
      138A ->140A         0.52062
      134B ->141B        -0.14776
      136B ->140B         0.32698
      136B ->144B        -0.11033
      137B ->139B         0.10413
      137B ->140B        -0.39532

Excited State  14:   2.772-A    3.4475 eV  359.63 nm  f=0.0033  <S**2>=1.671
      132A ->139A         0.14115
      134A ->139A        -0.14919
      138A ->141A         0.91029
      136B ->141B         0.10702
      137B ->141B        -0.16243

Excited State  15:   3.322-A    3.6037 eV  344.05 nm  f=0.0013  <S**2>=2.508
      131A ->139A         0.14876
      132A ->139A        -0.17892
      132A ->140A         0.11879
      133A ->139A         0.14288
      134A ->139A         0.22533
      134A ->140A        -0.10350
      137A ->141A        -0.40257
      138A ->141A        -0.11104
      134B ->139B        -0.10309
      134B ->140B        -0.17308
      135B ->141B        -0.14152
      136B ->141B         0.55592
      137B ->141B        -0.42764
      137B ->143B         0.10422

Excited State  16:   3.417-A    3.6165 eV  342.83 nm  f=0.0015  <S**2>=2.669
      134A ->140A         0.10338
      136A ->139A        -0.13640
      136A ->140A        -0.51663
      137A ->143A        -0.11501
      138A ->141A        -0.11123
      138A ->142A         0.23573
      138A ->143A        -0.20660
      135B ->138B        -0.21223
      135B ->140B         0.55357
      135B ->146B         0.15437
      135B ->147B         0.15208
```

135B ->148B -0.10252
136B ->140B 0.15756
137B ->140B 0.12395

Excited State 17: 2.782-A 3.6495 eV 339.73 nm f=0.0016 <S**2>=1.686
128A ->139A -0.10010
131A ->139A 0.27564
132A ->139A -0.41146
133A ->139A 0.23063
134A ->139A 0.49953
135A ->139A -0.11548
137A ->141A 0.15363
138A ->141A 0.30030
134B ->139B -0.16556
135B ->140B 0.22478
136B ->140B 0.11579
136B ->141B -0.17617
137B ->141B 0.18807

Excited State 18: 3.136-A 3.6914 eV 335.87 nm f=0.0040 <S**2>=2.209
132A ->139A -0.14641
134A ->139A 0.12833
134A ->141A -0.13960
136A ->141A 0.19866
137A ->139A 0.10807
137A ->140A 0.20205
137A ->142A -0.13347
138A ->140A 0.11961
138A ->142A 0.52365
138A ->143A 0.37702
134B ->141B -0.25630
134B ->143B 0.11889
135B ->141B -0.23212
135B ->143B 0.20667
136B ->140B -0.19610
136B ->146B -0.14767
137B ->146B 0.13755

Excited State 19: 2.463-A 3.8213 eV 324.46 nm f=0.1117 <S**2>=1.266
131A ->139A 0.22401
132A ->139A 0.30063
133A ->139A 0.60196
134A ->139A -0.16010
137A ->140A 0.34417
138A ->139A 0.14724
135B ->141B -0.10270
136B ->139B 0.21486
137B ->139B 0.32787

Excited State 20: 3.283-A 3.8935 eV 318.44 nm f=0.0017 <S**2>=2.445
133A ->139A 0.10952
134A ->141A 0.11911
136A ->141A -0.30969
138A ->142A 0.46022
134B ->145B 0.11016
135B ->141B 0.62637
135B ->143B -0.10144
135B ->146B 0.11041
136B ->140B -0.10779
136B ->141B 0.20261
137B ->140B -0.24230

Excited State 21: 2.893-A 3.9701 eV 312.29 nm f=0.0057 <S**2>=1.843
136A ->140A -0.29945
137A ->141A -0.10144
137A ->143A -0.15160
138A ->142A -0.34873
138A ->143A 0.74639
138A ->146A -0.11113
135B ->140B 0.12420
135B ->141B 0.15448

136B ->141B 0.11397

Excited State 22: 2.695-A 3.9857 eV 311.07 nm f=0.0021 <S**2>=1.566
121A ->139A 0.10537
133A ->139A 0.10210
137A ->139A -0.14690
137A ->140A -0.49303
137A ->142A 0.31655
137A ->143A 0.17416
137A ->145A 0.11586
138A ->142A 0.13869
138A ->143A 0.20509
135B ->141B 0.10905
135B ->143B 0.15220
135B ->145B -0.12804
136B ->139B 0.16233
136B ->140B -0.15174
137B ->140B 0.53270

Excited State 23: 3.116-A 4.0754 eV 304.22 nm f=0.0148 <S**2>=2.177
135A ->139A -0.15224
137A ->140A 0.28464
137A ->142A -0.22290
137A ->143A -0.13912
138A ->140A 0.13638
138A ->144A -0.11731
138A ->145A 0.37814
135B ->140B -0.11458
135B ->141B 0.25841
135B ->143B -0.11734
136B ->138B 0.10015
136B ->140B 0.28584
136B ->141B 0.11462
136B ->142B 0.11935
136B ->144B -0.27609
137B ->140B 0.38299
137B ->144B 0.20867
137B ->146B 0.12624

Excited State 24: 2.963-A 4.1151 eV 301.29 nm f=0.0234 <S**2>=1.944
133A ->139A 0.10447
135A ->139A 0.94235
137B ->140B 0.12651

Excited State 25: 3.129-A 4.1369 eV 299.70 nm f=0.0101 <S**2>=2.197
131A ->141A 0.10233
132A ->141A -0.19165
134A ->141A 0.16218
135A ->139A 0.16118
136A ->142A -0.16976
136A ->143A 0.17819
137A ->140A 0.11353
137A ->142A 0.15220
137A ->145A 0.13467
138A ->142A 0.42364
138A ->143A 0.20563
138A ->145A 0.26166
134B ->141B 0.33277
135B ->141B -0.17109
135B ->143B -0.14409
135B ->145B 0.17967
136B ->138B -0.12827
136B ->140B 0.25467
136B ->141B -0.11295
136B ->146B 0.13233
137B ->140B -0.10328
137B ->146B -0.10376

Excited State 26: 2.767-A 4.2253 eV 293.43 nm f=0.0001 <S**2>=1.664
136A ->140A 0.32189
136A ->142A -0.13845

136A ->143A -0.12894
137A ->141A -0.30329
137A ->143A -0.10889
138A ->145A -0.12385
133B ->138B 0.65635
134B ->138B 0.12036
134B ->140B -0.19078
135B ->140B 0.22893
135B ->146B -0.12388
136B ->141B -0.11551
136B ->143B 0.11927
136B ->145B -0.10831
137B ->143B -0.15251

Excited State 27: 2.711-A 4.2325 eV 292.93 nm f=0.0000 <S**2>=1.588
136A ->140A -0.30917
136A ->142A 0.11726
136A ->143A 0.13852
137A ->141A 0.24204
137A ->143A 0.11676
133B ->138B 0.73925
134B ->140B 0.12289
135B ->140B -0.25001
135B ->141B -0.10932
135B ->146B 0.12890

Excited State 28: 2.687-A 4.2458 eV 292.01 nm f=0.0078 <S**2>=1.555
136A ->139A 0.13382
136A ->140A 0.24175
136A ->142A -0.10694
137A ->141A 0.54342
137A ->142A 0.11560
137A ->143A -0.21857
134B ->140B 0.32044
135B ->140B 0.28111
135B ->142B 0.11709
135B ->144B -0.19086
135B ->146B -0.14901
136B ->141B 0.33499
137B ->141B -0.27541

Excited State 29: 3.044-A 4.3060 eV 287.94 nm f=0.0017 <S**2>=2.066
136A ->140A 0.22173
136A ->142A 0.12784
137A ->141A -0.14681
137A ->142A -0.11860
137A ->143A 0.19216
138A ->143A 0.12846
138A ->146A -0.10428
134B ->140B 0.16550
135B ->138B -0.10712
135B ->140B 0.31439
135B ->143B 0.16134
135B ->145B -0.10138
136B ->140B 0.21917
136B ->143B -0.30299
136B ->144B 0.13089
136B ->145B 0.29827
137B ->141B 0.25985
137B ->143B 0.32569
137B ->145B -0.27940

Excited State 30: 3.229-A 4.3530 eV 284.82 nm f=0.0122 <S**2>=2.357
136A ->142A 0.19385
136A ->143A -0.29790
137A ->140A 0.14431
137A ->141A 0.14081
138A ->145A -0.29099
134B ->141B 0.41174
135B ->140B -0.19609
135B ->141B 0.10423

```
      135B ->143B        0.38445
      135B ->145B       -0.19289
      136B ->140B        0.27922
      136B ->143B        0.18703
      136B ->145B       -0.12930
      137B ->140B        0.16404
      137B ->141B       -0.14960

 Excited State  31:      3.049-A      4.3808 eV  283.01 nm  f=0.0015  <S**2>=2.074
      133A ->139A       -0.11406
      136A ->141A       -0.13229
      136A ->143A       -0.20237
      137A ->140A        0.21484
      137A ->142A        0.14755
      138A ->140A        0.11489
      138A ->144A       -0.14151
      138A ->145A        0.45484
      123B ->138B        0.10489
      124B ->138B        0.12308
      128B ->138B        0.12901
      129B ->138B        0.19127
      134B ->141B       -0.20192
      135B ->141B        0.17320
      135B ->145B       -0.14573
      136B ->138B       -0.13309
      136B ->141B        0.10969
      136B ->142B       -0.10074
      136B ->143B        0.11492
      136B ->144B        0.17097
      136B ->146B        0.26067
      137B ->144B       -0.21181
      137B ->146B       -0.23016

 Excited State  32:      2.997-A      4.4269 eV  280.07 nm  f=0.0060  <S**2>=1.995
      132A ->141A        0.14607
      136A ->141A       -0.22869
      136A ->143A        0.19817
      138A ->145A       -0.24335
      124B ->138B        0.12420
      128B ->138B        0.11929
      129B ->138B        0.17502
      134B ->141B       -0.27104
      135B ->140B       -0.20486
      135B ->141B       -0.15038
      135B ->143B       -0.19906
      135B ->145B        0.15146
      136B ->138B       -0.12476
      136B ->139B       -0.12251
      136B ->140B        0.28696
      136B ->144B        0.26908
      137B ->140B        0.37822
      137B ->142B        0.10149
      137B ->144B       -0.18162

 Excited State  33:      3.032-A      4.4657 eV  277.64 nm  f=0.0018  <S**2>=2.049
      123A ->139A        0.12933
      128A ->139A        0.11528
      131A ->139A       -0.19424
      132A ->139A        0.45487
      132A ->140A       -0.14159
      134A ->139A        0.68948
      134A ->140A        0.14154
      136A ->139A        0.11630
      136A ->140A       -0.13861
      137A ->141A       -0.11734
      137A ->143A        0.10032
      136B ->141B       -0.10278

 Excited State  34:      2.867-A      4.5253 eV  273.98 nm  f=0.0003  <S**2>=1.805
      132A ->139A       -0.22352
      134A ->139A       -0.16931
```

134A ->140A 0.12012
136A ->140A -0.10121
136A ->142A -0.12371
137A ->141A -0.22347
137A ->143A 0.15756
132B ->138B 0.60705
134B ->138B -0.16639
134B ->140B 0.32230
134B ->146B 0.10127
135B ->138B 0.12662
135B ->141B 0.10201
135B ->144B -0.16153
135B ->146B -0.14260
136B ->141B -0.15221
137B ->141B -0.22346

Excited State 35: 2.782-A 4.5733 eV 271.10 nm f=0.0043 <S**2>=1.684
136A ->141A 0.21189
137A ->142A 0.16191
137A ->143A -0.11437
138A ->145A -0.20971
132B ->138B 0.56497
134B ->140B -0.14557
136B ->141B 0.34176
137B ->141B 0.49876

Excited State 36: 2.922-A 4.6041 eV 269.29 nm f=0.0060 <S**2>=1.885
132A ->139A 0.11489
132A ->140A 0.19111
133A ->139A 0.10174
134A ->140A -0.15377
134A ->141A 0.11650
136A ->141A -0.25649
137A ->140A -0.18683
137A ->141A 0.12397
137A ->143A -0.22325
137A ->145A -0.14755
138A ->144A -0.11801
138A ->145A 0.33844
132B ->138B 0.36137
134B ->140B -0.30042
134B ->143B -0.11957
135B ->141B -0.15602
135B ->143B 0.12912
136B ->141B -0.18829
136B ->146B -0.12642
137B ->143B 0.23415
137B ->144B -0.13842
137B ->146B 0.14608

Excited State 37: 2.933-A 4.6392 eV 267.25 nm f=0.0050 <S**2>=1.901
136A ->141A 0.57839
137A ->142A 0.19265
137A ->143A 0.19628
137A ->145A -0.28303
138A ->145A 0.18627
135B ->141B 0.19157
135B ->143B -0.13549
136B ->139B -0.19517
136B ->140B 0.16245
136B ->143B -0.12613
136B ->144B 0.12944
136B ->146B -0.23732
137B ->144B -0.13333
137B ->146B 0.17137

Excited State 38: 3.039-A 4.6652 eV 265.76 nm f=0.0028 <S**2>=2.058
131A ->140A -0.15173
132A ->140A 0.13048
132A ->141A 0.12732
133A ->139A -0.17845

133A ->140A -0.14803
134A ->140A -0.15395
134A ->141A -0.11709
136A ->141A 0.11180
137A ->140A 0.13040
137A ->141A 0.12421
137A ->142A 0.12722
137A ->145A 0.10860
138A ->145A -0.18994
134B ->140B -0.21099
134B ->141B -0.20546
135B ->141B 0.25809
135B ->146B -0.12728
136B ->141B -0.32465
136B ->143B -0.23409
137B ->141B -0.30910
137B ->143B 0.36595
137B ->145B -0.11989
137B ->146B -0.13980

Excited State 39: 3.016-A 4.7077 eV 263.37 nm f=0.0052 <S**2>=2.024
134A ->142A -0.10821
136A ->140A 0.28918
136A ->141A 0.18881
136A ->142A 0.37923
136A ->143A 0.21325
137A ->142A 0.14183
138A ->146A -0.37358
129B ->138B 0.11233
132B ->138B 0.22000
135B ->141B 0.15328
135B ->144B 0.16770
135B ->146B 0.28240
135B ->147B 0.11091
136B ->141B -0.11341
136B ->143B 0.10486
137B ->141B -0.23449
137B ->143B -0.21249
137B ->144B 0.11631
137B ->145B 0.10739

Excited State 40: 2.849-A 4.7211 eV 262.62 nm f=0.0071 <S**2>=1.779
124A ->139A 0.14219
131A ->139A 0.35657
132A ->139A 0.16524
133A ->139A -0.18964
133A ->140A 0.13356
136A ->142A -0.11435
137A ->142A -0.19095
138A ->146A 0.10333
121B ->138B 0.12414
123B ->138B 0.17195
124B ->138B 0.21750
126B ->138B 0.12643
127B ->138B 0.13491
128B ->138B 0.28190
129B ->138B 0.41150
130B ->138B -0.14622
131B ->138B 0.15481
134B ->141B 0.12554
136B ->140B -0.19469
136B ->144B -0.18135
136B ->146B -0.14918
137B ->142B -0.11342
137B ->144B 0.16237
137B ->146B 0.16468

Excited State 41: 2.899-A 4.7276 eV 262.26 nm f=0.0044 <S**2>=1.851
124A ->139A 0.20744
131A ->139A 0.51767
132A ->139A 0.22098

```
      133A ->139A        -0.41634
      137A ->143A         0.17445
      123B ->138B        -0.10479
      124B ->138B        -0.11312
      128B ->138B        -0.16398
      129B ->138B        -0.24160
      136B ->140B         0.16039
      136B ->144B         0.14625
      137B ->139B         0.11584
      137B ->142B         0.10951
      137B ->143B        -0.12996
      137B ->144B        -0.22067
      137B ->146B         0.12886

 Excited State  42:   2.767-A      4.7610 eV  260.42 nm  f=0.0047  <S**2>=1.664
      131A ->139A        -0.17698
      133A ->139A         0.11102
      137A ->141A         0.26622
      137A ->142A        -0.37399
      137A ->143A         0.51497
      138A ->146A        -0.20377
      132B ->138B         0.13104
      134B ->138B         0.10890
      134B ->140B        -0.34649
      134B ->146B        -0.18953
      134B ->147B        -0.10650
      135B ->140B         0.13686
      135B ->144B        -0.12189
      136B ->143B         0.11372
      137B ->141B        -0.11849
      137B ->143B        -0.21066

 Excited State  43:   2.558-A      4.8167 eV  257.40 nm  f=0.0004  <S**2>=1.386
      138A ->146A         0.31200
      128B ->138B        -0.10938
      129B ->138B        -0.13816
      131B ->138B         0.81974
      132B ->138B         0.16885
      135B ->146B         0.10265
      136B ->145B         0.10027
      137B ->143B        -0.10054
      137B ->145B        -0.11839

 Excited State  44:   2.893-A      4.8315 eV  256.62 nm  f=0.0011  <S**2>=1.842
      138A ->146A         0.53515
      131B ->138B        -0.46860
      134B ->146B        -0.17984
      135B ->144B         0.14905
      135B ->146B         0.21645
      136B ->145B         0.22615
      137B ->141B        -0.16267
      137B ->143B        -0.24314
      137B ->145B        -0.23984

 Excited State  45:   2.776-A      4.8598 eV  255.12 nm  f=0.0039  <S**2>=1.677
      121A ->139A        -0.27738
      122A ->139A        -0.32302
      123A ->139A         0.17536
      125A ->139A         0.37453
      126A ->139A        -0.22460
      127A ->139A        -0.36614
      130A ->139A         0.14316
      131A ->140A        -0.17705
      132A ->140A        -0.15859
      133A ->139A        -0.15884
      133A ->140A        -0.32277
      137A ->142A        -0.12856
      137A ->145A        -0.10444
      136B ->139B         0.13114
      136B ->140B        -0.12090
      136B ->144B        -0.10307
```

136B ->146B 0.21567

Excited State 46: 2.854-A 4.9094 eV 252.55 nm f=0.0077 <S**2>=1.786
122A ->139A -0.11361
125A ->139A 0.14180
127A ->139A -0.14476
131A ->139A 0.10008
132A ->140A 0.25261
133A ->140A 0.22450
134A ->140A -0.14823
136A ->141A -0.12420
136A ->142A -0.13439
137A ->142A 0.18468
137A ->143A 0.21175
138A ->146A -0.19431
134B ->140B 0.17193
135B ->139B -0.15444
135B ->144B 0.36389
135B ->146B -0.25871
136B ->140B 0.11980
136B ->144B 0.14266
137B ->143B -0.12037
137B ->144B 0.34340

Excited State 47: 2.675-A 4.9269 eV 251.65 nm f=0.0041 <S**2>=1.539
125A ->139A 0.11077
127A ->139A -0.13350
131A ->139A 0.18854
131A ->140A 0.33381
133A ->140A 0.53095
136A ->141A 0.12837
136A ->143A 0.27584
138A ->146A 0.20882
128B ->138B -0.10832
129B ->138B -0.12039
134B ->140B -0.17051
134B ->143B 0.11004
135B ->141B 0.10596
135B ->144B -0.19998
136B ->140B 0.11896
136B ->146B 0.15815
137B ->146B -0.19581

Excited State 48: 2.565-A 4.9565 eV 250.15 nm f=0.0087 <S**2>=1.395
132A ->140A -0.15457
132A ->141A 0.14125
134A ->140A 0.12753
136A ->141A -0.29587
136A ->142A 0.20759
136A ->143A -0.12183
137A ->142A 0.44237
137A ->143A 0.30844
138A ->146A 0.17687
134B ->140B -0.15313
135B ->141B -0.17122
135B ->144B -0.20820
135B ->145B 0.11698
135B ->146B 0.10921
136B ->144B -0.11488
136B ->146B -0.12042
137B ->142B -0.16945
137B ->144B 0.27683
137B ->146B 0.14132

Excited State 49: 2.781-A 4.9800 eV 248.96 nm f=0.0091 <S**2>=1.684
133B ->139B 0.97296

Excited State 50: 2.470-A 5.0063 eV 247.66 nm f=0.0004 <S**2>=1.276
126B ->138B 0.15335
128B ->138B -0.15602
129B ->138B 0.32961

130B ->138B 0.91370

Excited State 51: 2.711-A 5.0407 eV 245.97 nm f=0.0011 <S**2>=1.587
132A ->139A -0.10905
132A ->140A -0.23276
134A ->140A 0.20871
136A ->142A 0.19882
136A ->143A 0.12457
138A ->144A -0.20476
138A ->146A 0.21714
127B ->138B -0.10896
128B ->138B 0.56826
129B ->138B -0.24801
130B ->138B 0.19400
134B ->140B -0.14861
135B ->144B 0.18718
135B ->146B -0.27996

Excited State 52: 2.669-A 5.0457 eV 245.72 nm f=0.0002 <S**2>=1.531
132A ->139A 0.13161
132A ->140A 0.24856
134A ->140A -0.22151
138A ->144A 0.19367
138A ->146A -0.13370
127B ->138B -0.10428
128B ->138B 0.60107
129B ->138B -0.29166
130B ->138B 0.19542
134B ->140B 0.12095
135B ->139B 0.10487
135B ->144B -0.22804
135B ->146B 0.25799
136B ->144B -0.14334

Excited State 53: 2.730-A 5.0684 eV 244.62 nm f=0.0064 <S**2>=1.613
131A ->140A 0.11024
132A ->139A -0.11611
132A ->140A -0.20034
134A ->140A 0.20734
136A ->142A -0.11459
137A ->143A -0.13312
138A ->144A 0.73117
138A ->145A 0.21976
134B ->140B -0.11574
135B ->144B 0.13165
136B ->145B 0.13044
137B ->144B -0.14973
137B ->145B -0.26876

Excited State 54: 2.668-A 5.0804 eV 244.04 nm f=0.0017 <S**2>=1.529
130A ->139A 0.18270
132A ->140A 0.16603
132A ->141A -0.12856
133A ->141A -0.19300
134A ->140A -0.15268
136A ->142A 0.40242
136A ->143A 0.16619
137A ->143A 0.10467
137A ->145A 0.17856
138A ->144A 0.27147
138A ->146A 0.31886
128B ->138B -0.10624
135B ->146B -0.19297
136B ->143B -0.12406
136B ->144B -0.17803
136B ->145B -0.11815
136B ->146B -0.15349
137B ->142B 0.14122
137B ->144B -0.23595
137B ->145B 0.23053
137B ->146B -0.12797

```
Excited State  55:   2.876-A    5.0884 eV  243.66 nm  f=0.0041  <S**2>=1.818
     130A ->139A        0.48725
     136A ->143A       -0.15402
     137A ->142A        0.14930
     137A ->145A        0.26520
     138A ->144A       -0.41159
     138A ->145A       -0.14585
     138A ->146A       -0.19023
     136B ->144B       -0.18649
     136B ->145B        0.10502
     136B ->146B       -0.12283
     137B ->142B        0.28524
     137B ->144B       -0.27567
     137B ->145B       -0.18224

Excited State  56:   2.475-A    5.0949 eV  243.35 nm  f=0.0001  <S**2>=1.281
     125B ->138B       -0.10458
     127B ->138B        0.93319
     129B ->138B       -0.26964
     130B ->138B        0.11050

Excited State  57:   2.903-A    5.1086 eV  242.70 nm  f=0.0018  <S**2>=1.857
     121A ->139A        0.15065
     125A ->139A       -0.16905
     128A ->139A        0.16485
     130A ->139A        0.77976
     137A ->142A       -0.14022
     137A ->145A       -0.10540
     138A ->144A        0.12095
     136B ->144B        0.19580
     136B ->146B        0.11585
     137B ->142B       -0.18984
     137B ->144B        0.19594
     137B ->145B        0.16069

Excited State  58:   2.952-A    5.1448 eV  240.99 nm  f=0.0013  <S**2>=1.928
     120A ->139A        0.10655
     125A ->139A        0.16787
     128A ->139A        0.63691
     129A ->139A        0.16295
     130A ->139A       -0.16072
     131A ->139A        0.10324
     132A ->139A       -0.20430
     136A ->143A       -0.10578
     136A ->145A       -0.10495
     135B ->143B        0.12340
     135B ->146B        0.10169
     136B ->143B       -0.30714
     136B ->145B        0.11342
     137B ->143B       -0.13307
     137B ->145B        0.34833
     137B ->149B        0.10201

Excited State  59:   2.682-A    5.1796 eV  239.37 nm  f=0.0082  <S**2>=1.548
     128A ->139A        0.13031
     137A ->143A        0.10979
     137A ->145A        0.19816
     125B ->138B       -0.31233
     126B ->138B        0.51813
     127B ->138B       -0.12379
     129B ->138B       -0.25401
     129B ->139B        0.12623
     135B ->143B       -0.14943
     135B ->144B        0.18705
     136B ->143B        0.26610
     136B ->145B       -0.20275
     137B ->142B       -0.17527
     137B ->143B        0.26707
     137B ->144B       -0.14604
```

Excited State 60: 2.726-A 5.1883 eV 238.97 nm f=0.0047 <S**2>=1.608
128A ->139A 0.21558
137A ->143A 0.11529
125B ->138B 0.43728
126B ->138B -0.40140
128B ->138B 0.13159
129B ->138B 0.17975
135B ->143B -0.11226
135B ->144B 0.19539
135B ->145B 0.10113
136B ->143B 0.36949
136B ->144B -0.10217
136B ->145B -0.20608
137B ->143B 0.33744
137B ->144B -0.15139

Excited State 61: 2.650-A 5.1924 eV 238.78 nm f=0.0012 <S**2>=1.505
137A ->145A 0.25737
123B ->139B 0.12168
124B ->139B 0.14270
125B ->138B 0.69285
126B ->138B 0.13137
128B ->139B 0.14330
129B ->139B 0.21450
135B ->145B -0.10446
136B ->143B -0.13313
136B ->144B 0.13729
137B ->142B -0.28571
137B ->143B -0.10480

Excited State 62: 2.754-A 5.2024 eV 238.32 nm f=0.0167 <S**2>=1.646
128A ->139A 0.33988
129A ->139A 0.23110
136A ->142A 0.14669
136A ->143A 0.15290
137A ->145A -0.17425
137A ->146A -0.14731
125B ->138B 0.27403
126B ->138B 0.48227
128B ->138B -0.12985
135B ->144B -0.19347
136B ->143B 0.12457
137B ->142B 0.24431
137B ->144B 0.15126
137B ->145B -0.25663

Excited State 63: 2.783-A 5.2080 eV 238.06 nm f=0.0088 <S**2>=1.686
128A ->139A -0.30864
129A ->139A -0.18318
132A ->141A 0.10137
136A ->142A -0.16946
137A ->145A -0.17387
123B ->139B -0.11444
124B ->139B -0.12498
125B ->138B 0.33268
126B ->138B 0.41216
128B ->139B -0.11599
129B ->139B -0.19703
134B ->144B 0.10986
135B ->143B 0.11219
135B ->144B 0.23061
136B ->144B -0.12877
137B ->142B 0.17680
137B ->144B -0.12757
137B ->145B 0.26018

Excited State 64: 2.824-A 5.2260 eV 237.25 nm f=0.0078 <S**2>=1.743
129A ->139A -0.11276
131A ->141A 0.10280
133A ->141A 0.12579
137A ->146A 0.12382

124B ->139B 0.10034
129B ->139B 0.13309
136B ->144B 0.12468
136B ->145B -0.13360
137B ->142B 0.69932
137B ->143B 0.18689
137B ->144B 0.42171
137B ->147B -0.12755

Excited State 65: 2.889-A 5.2349 eV 236.84 nm f=0.0027 <S**2>=1.836
126A ->139A -0.14944
127A ->139A 0.20878
128A ->139A -0.34819
129A ->139A 0.85115
131A ->139A 0.10586

Excited State 66: 3.009-A 5.2861 eV 234.55 nm f=0.0068 <S**2>=2.013
127A ->139A 0.11249
129A ->139A -0.18605
132A ->142A 0.11537
132A ->143A 0.11113
136A ->142A -0.11271
137A ->145A -0.19104
137A ->146A 0.18444
132B ->139B -0.37401
134B ->138B -0.11619
134B ->139B 0.18551
134B ->142B -0.10767
134B ->144B 0.18701
134B ->145B 0.17201
134B ->146B 0.41311
134B ->147B 0.13120
135B ->145B 0.17760
135B ->146B 0.14213
137B ->143B -0.16077
137B ->145B -0.21121
137B ->146B -0.24404

Excited State 67: 2.784-A 5.3025 eV 233.82 nm f=0.0026 <S**2>=1.688
127A ->139A 0.12261
128A ->139A 0.10313
131A ->141A -0.13943
132A ->141A -0.20626
133A ->141A -0.29434
136A ->143A -0.11908
132B ->139B 0.66290
134B ->139B -0.15032
134B ->141B -0.10297
134B ->143B -0.11293
134B ->144B 0.32383
134B ->145B 0.10811
135B ->145B 0.12660
137B ->146B -0.17713

Excited State 68: 3.002-A 5.3074 eV 233.61 nm f=0.0068 <S**2>=2.003
127A ->139A -0.13714
131A ->141A -0.19140
132A ->142A 0.11030
133A ->141A -0.29449
136A ->141A -0.11494
136A ->142A -0.17420
137A ->145A 0.15570
134B ->141B 0.13317
134B ->142B -0.13908
134B ->143B 0.31000
134B ->144B 0.23251
134B ->145B -0.31661
134B ->146B 0.24007
134B ->147B 0.10992
135B ->143B -0.10400
135B ->145B -0.25156

```
      136B ->144B        0.11154
      136B ->145B       -0.12805
      136B ->146B        0.11175
      137B ->145B       -0.10009
      137B ->146B        0.35883

 Excited State  69:   2.915-A      5.3274 eV  232.73 nm  f=0.0150  <S**2>=1.874
      124A ->139A       -0.12128
      125A ->139A        0.30614
      126A ->139A       -0.33334
      127A ->139A        0.76148
      129A ->139A       -0.24978
      131A ->139A        0.10566
      134B ->143B        0.12073
      134B ->145B       -0.11394

 Excited State  70:   3.042-A      5.3658 eV  231.06 nm  f=0.0031  <S**2>=2.064
      125A ->139A        0.17169
      126A ->139A        0.40760
      131A ->141A        0.11428
      132A ->141A        0.14590
      132A ->142A        0.12083
      132A ->143A       -0.11920
      133A ->141A        0.23219
      134A ->142A       -0.13226
      136A ->141A       -0.14880
      137A ->145A       -0.11374
      132B ->139B        0.34266
      134B ->141B        0.10396
      134B ->143B        0.43734
      134B ->144B       -0.13413
      134B ->145B       -0.20037
      135B ->143B        0.10675
      135B ->144B        0.10603
      136B ->146B       -0.17667
      137B ->146B       -0.16504

 Excited State  71:   2.943-A      5.3690 eV  230.92 nm  f=0.0002  <S**2>=1.915
      125A ->139A        0.55331
      126A ->139A        0.62835
      127A ->139A        0.12454
      128A ->139A       -0.22146
      132B ->139B       -0.13031
      134B ->143B       -0.24607
      135B ->145B       -0.12753

 Excited State  72:   2.948-A      5.3748 eV  230.68 nm  f=0.0049  <S**2>=1.922
      126A ->139A        0.26028
      131A ->141A       -0.12531
      133A ->141A       -0.20830
      133A ->143A        0.10038
      134A ->143A        0.10959
      137A ->146A       -0.17731
      123B ->139B        0.13081
      124B ->139B        0.12077
      128B ->139B        0.14208
      129B ->139B        0.22110
      132B ->139B       -0.32342
      134B ->143B        0.18333
      134B ->146B       -0.17896
      135B ->143B        0.17753
      135B ->145B        0.33937
      136B ->143B        0.19536
      136B ->144B       -0.12422
      136B ->145B        0.30137
      137B ->143B        0.17078
      137B ->145B        0.18907

 Excited State  73:   2.776-A      5.4192 eV  228.79 nm  f=0.0041  <S**2>=1.676
      124A ->139A       -0.10412
      125A ->139A       -0.16218
```

131A ->141A -0.14672
132A ->140A -0.10015
132A ->141A 0.10827
132A ->142A -0.15130
133A ->140A -0.16592
133A ->141A -0.15409
133A ->142A -0.16949
134A ->141A -0.13280
134A ->142A 0.13163
136A ->142A -0.25046
136A ->143A 0.30613
137A ->145A -0.16653
124B ->139B 0.11136
128B ->139B 0.11813
129B ->139B 0.17063
134B ->141B 0.15504
134B ->143B -0.11865
134B ->146B -0.16416
135B ->143B 0.14211
135B ->145B -0.29471
135B ->146B 0.13874
136B ->143B -0.17245
136B ->145B -0.30606
137B ->146B -0.24083

Excited State 74: 2.732-A 5.4389 eV 227.96 nm f=0.0012 <S**2>=1.616
132A ->141A 0.20580
134A ->141A -0.15952
134A ->143A -0.10737
136A ->143A 0.16369
123B ->138B 0.21971
124B ->138B 0.37971
126B ->138B -0.17818
128B ->138B -0.13598
128B ->139B 0.11651
129B ->138B -0.22737
129B ->139B 0.18315
132B ->139B 0.15948
134B ->143B -0.18012
134B ->145B 0.16527
134B ->146B 0.20200
135B ->146B -0.21659
136B ->144B -0.13617
136B ->145B 0.11091
136B ->146B 0.25502
137B ->146B 0.30481

Excited State 75: 2.794-A 5.4611 eV 227.03 nm f=0.0022 <S**2>=1.702
124A ->139A -0.14758
132A ->141A 0.11177
136A ->143A 0.17852
136A ->145A -0.12737
123B ->138B -0.23887
124B ->138B -0.37007
126B ->138B 0.14416
129B ->138B 0.21328
134B ->143B -0.17771
134B ->146B 0.19694
135B ->145B -0.22281
136B ->142B -0.11208
136B ->143B 0.32632
136B ->145B 0.41613
137B ->145B 0.22425
137B ->149B 0.12549

Excited State 76: 2.736-A 5.4921 eV 225.75 nm f=0.0013 <S**2>=1.621
123A ->139A -0.13285
124A ->139A 0.53506
131A ->139A -0.20705
123B ->138B -0.24054
123B ->139B 0.14589

```
     124B ->138B        -0.36299
     126B ->138B         0.11696
     128B ->139B         0.12702
     129B ->138B         0.15803
     129B ->139B         0.21187
     131B ->139B        -0.28738
     135B ->143B         0.15190
     135B ->145B         0.16418
     135B ->146B        -0.10615
     136B ->143B        -0.12342
     136B ->145B        -0.11648
     136B ->146B         0.16412

 Excited State  77:   2.904-A      5.5056 eV  225.20 nm  f=0.0022  <S**2>=1.858
     123A ->139A        -0.10955
     124A ->139A         0.50603
     125A ->139A         0.16932
     126A ->139A        -0.14627
     131A ->139A        -0.15145
     133A ->142A        -0.12812
     123B ->138B         0.16530
     124B ->138B         0.22829
     131B ->139B        -0.22934
     135B ->145B        -0.22993
     135B ->146B         0.15859
     136B ->143B         0.18923
     136B ->145B         0.18887
     136B ->146B        -0.29888
     137B ->143B         0.10441
     137B ->145B         0.14960
     137B ->146B        -0.29783

 Excited State  78:   2.907-A      5.5909 eV  221.76 nm  f=0.0058  <S**2>=1.863
     121A ->139A         0.21802
     122A ->139A         0.37469
     123A ->139A        -0.13304
     125A ->139A         0.35488
     126A ->139A        -0.25177
     127A ->139A        -0.18257
     133A ->142A        -0.13768
     136A ->146A        -0.18118
     137A ->145A         0.13208
     124B ->138B         0.13316
     131B ->139B         0.30389
     134B ->143B        -0.10027
     134B ->144B        -0.12330
     135B ->143B         0.30578
     135B ->145B         0.26676
     136B ->144B         0.11723

 Excited State  79:   2.779-A      5.6205 eV  220.59 nm  f=0.0002  <S**2>=1.681
     134A ->145A        -0.19931
     136A ->140A         0.12961
     136A ->144A        -0.24377
     136A ->145A         0.83735
     137A ->146A         0.13687
     135B ->144B         0.13055
     135B ->146B        -0.11341
     136B ->145B         0.17900

 Excited State  80:   2.606-A      5.6276 eV  220.31 nm  f=0.0007  <S**2>=1.447
     121A ->139A         0.10326
     122A ->139A         0.17183
     125A ->139A         0.11805
     131A ->143A        -0.11736
     133A ->143A        -0.17470
     122B ->138B         0.25448
     123B ->138B         0.66154
     124B ->138B        -0.48813
     134B ->144B         0.19476
```

Excited State 81: 2.900-A 5.6352 eV 220.02 nm f=0.0029 <S**2>=1.852
122A ->139A 0.10987
131A ->143A -0.20548
132A ->142A 0.15785
133A ->141A 0.18626
133A ->142A 0.17019
133A ->143A -0.32901
137A ->146A -0.23233
123B ->138B -0.30944
124B ->138B 0.21030
132B ->139B -0.12391
134B ->139B -0.12933
134B ->142B -0.14630
134B ->144B 0.51379
134B ->146B -0.18628
137B ->145B 0.10566

Excited State 82: 2.887-A 5.6543 eV 219.27 nm f=0.0067 <S**2>=1.834
122A ->139A 0.18480
124A ->139A 0.29883
131A ->143A 0.12238
133A ->142A 0.17598
133A ->143A 0.18840
136A ->146A 0.18724
137A ->145A -0.20115
131B ->139B 0.61132
134B ->143B 0.10964
134B ->145B 0.15289
135B ->143B -0.22979
135B ->145B -0.19830
136B ->144B -0.17508
136B ->146B 0.10566

Excited State 83: 2.653-A 5.6735 eV 218.53 nm f=0.0114 <S**2>=1.509
121A ->139A 0.14447
122A ->139A 0.29241
124A ->139A -0.16249
125A ->139A 0.14068
131A ->142A -0.11443
131A ->143A -0.14222
132A ->141A -0.17742
132A ->142A -0.26883
133A ->142A -0.28505
133A ->143A -0.15051
134A ->141A 0.16597
134A ->142A 0.13594
136A ->143A -0.10266
137A ->144A 0.10923
137A ->145A -0.21751
123B ->138B -0.16641
129B ->139B 0.10332
131B ->139B -0.26627
134B ->143B 0.26255
134B ->145B 0.10785
135B ->143B -0.23142
135B ->145B -0.10484
136B ->146B 0.19960

Excited State 84: 2.772-A 5.7039 eV 217.37 nm f=0.0003 <S**2>=1.671
121A ->139A -0.13165
122A ->139A -0.34469
123A ->139A 0.15827
124A ->139A 0.32670
125A ->139A -0.14678
131A ->142A -0.11786
131A ->143A -0.19621
132A ->141A -0.11782
132A ->142A -0.25167
133A ->142A -0.28983
133A ->143A -0.24446
134A ->141A 0.12260

134A ->142A 0.12939
134A ->143A -0.13597
131B ->139B 0.44876
134B ->143B 0.10254
137B ->146B 0.12107

Excited State 85: 3.124-A 5.7503 eV 215.62 nm f=0.0005 <S**2>=2.190
135A ->140A 0.94000
137B ->149B -0.12136

Excited State 86: 3.046-A 5.7745 eV 214.71 nm f=0.0010 <S**2>=2.070
122A ->139A -0.16741
123A ->139A -0.34312
133A ->143A -0.10065
135A ->140A -0.17295
138A ->147A -0.22752
138A ->150A 0.13115
122B ->138B -0.21199
123B ->138B 0.11796
136B ->142B -0.45809
137B ->145B 0.11277
137B ->147B 0.47570
137B ->149B -0.26676
137B ->150B -0.10843

Excited State 87: 2.455-A 5.7887 eV 214.18 nm f=0.0003 <S**2>=1.256
123A ->139A -0.18192
137A ->146A 0.12936
122B ->138B 0.90045
123B ->138B -0.21885
124B ->138B 0.16206
136B ->142B -0.12546

Excited State 88: 2.987-A 5.7997 eV 213.78 nm f=0.0010 <S**2>=1.980
122A ->139A 0.29732
123A ->139A 0.68740
124A ->139A 0.14246
138A ->147A -0.13321
122B ->138B 0.12638
136B ->142B -0.42302
137B ->147B 0.26462
137B ->149B 0.14714

Excited State 89: 2.847-A 5.8268 eV 212.78 nm f=0.0094 <S**2>=1.776
131A ->142A 0.19166
132A ->142A -0.18160
132A ->143A -0.20340
133A ->141A -0.14493
133A ->142A 0.18496
133A ->143A -0.18434
134A ->142A 0.20471
134A ->143A 0.11477
137A ->146A 0.73149
122B ->138B -0.12600
136B ->145B 0.11858

Excited State 90: 2.863-A 5.8281 eV 212.74 nm f=0.0001 <S**2>=1.799
126B ->139B 0.14521
129B ->139B 0.22092
130B ->139B 0.93983

Excited State 91: 2.969-A 5.8335 eV 212.54 nm f=0.0009 <S**2>=1.954
122A ->139A -0.13274
123A ->139A -0.26268
131A ->143A 0.17402
132A ->142A -0.17188
133A ->141A 0.16291
133A ->142A -0.10600
133A ->143A 0.24478
134A ->142A 0.10799
135A ->140A 0.13910

138A ->148A 0.10229
138A ->149A 0.21979
138A ->150A -0.25348
130B ->139B -0.16120
134B ->142B -0.10097
134B ->144B 0.24858
136B ->142B -0.33314
136B ->145B -0.17953
137B ->145B -0.12583
137B ->148B -0.11195
137B ->149B 0.38035
137B ->150B 0.14660

Excited State 92: 2.969-A 5.8764 eV 210.99 nm f=0.0005 <S**2>=1.954
123A ->139A 0.11994
131A ->143A 0.10363
132A ->142A -0.17726
133A ->141A 0.14350
133A ->143A 0.18110
134A ->142A 0.12926
138A ->147A -0.39742
134B ->144B 0.18024
135B ->142B 0.26841
136B ->142B 0.49516
136B ->143B 0.11878
136B ->144B 0.16540
137B ->147B 0.37952
137B ->148B -0.11899
137B ->149B -0.12749

Excited State 93: 2.861-A 5.8800 eV 210.86 nm f=0.0001 <S**2>=1.797
125B ->139B 0.14100
127B ->139B -0.17605
128B ->139B 0.84779
129B ->139B -0.40860
130B ->139B 0.11578

Excited State 94: 2.886-A 5.9017 eV 210.08 nm f=0.0022 <S**2>=1.833
122A ->139A -0.10767
123A ->139A -0.16586
131A ->143A -0.18375
132A ->141A -0.12060
132A ->142A 0.17619
133A ->141A -0.16773
133A ->142A 0.13856
133A ->143A -0.27135
134A ->142A -0.11086
138A ->147A -0.42062
138A ->148A 0.12463
138A ->149A 0.11163
138A ->150A -0.10864
134B ->144B -0.23780
136B ->142B 0.19486
137B ->145B -0.13136
137B ->148B -0.20083
137B ->149B 0.44570
137B ->150B 0.15887

Excited State 95: 2.780-A 5.9307 eV 209.05 nm f=0.0069 <S**2>=1.682
132A ->143A 0.12060
133A ->142A 0.10878
134A ->143A -0.12777
138A ->147A 0.44333
127B ->139B 0.53837
129B ->139B -0.17362
135B ->142B 0.42385
135B ->144B 0.12319
137B ->147B 0.23725
137B ->148B -0.10303
137B ->149B 0.12151

```
Excited State  96:   2.761-A    5.9345 eV  208.92 nm  f=0.0044  <S**2>=1.656
     132A ->143A        -0.16936
     133A ->142A        -0.10670
     134A ->143A         0.15974
     138A ->147A        -0.37111
     125B ->139B        -0.10874
     127B ->139B         0.73636
     129B ->139B        -0.19286
     135B ->142B        -0.11630
     137B ->147B        -0.22139
     137B ->148B         0.10286

Excited State  97:   2.912-A    5.9404 eV  208.71 nm  f=0.0027  <S**2>=1.870
     132A ->143A        -0.25341
     134A ->142A        -0.11729
     134A ->143A         0.20335
     138A ->147A        -0.10006
     127B ->139B        -0.19708
     135B ->140B        -0.11553
     135B ->142B         0.73276
     135B ->144B         0.23015
     137B ->147B        -0.25944
     137B ->148B         0.13128

Excited State  98:   2.685-A    5.9940 eV  206.85 nm  f=0.0019  <S**2>=1.553
     122A ->139A         0.13331
     131A ->142A         0.21209
     132A ->142A        -0.13240
     132A ->143A         0.28608
     133A ->142A         0.25520
     134A ->142A         0.20184
     134A ->143A        -0.24698
     136A ->146A         0.11690
     137A ->144A         0.34615
     137A ->145A         0.16879
     138A ->147A        -0.32688
     134B ->145B        -0.21297
     135B ->142B         0.16391
     135B ->143B         0.11299
     136B ->142B        -0.14645
     136B ->146B        -0.16418
     137B ->147B        -0.32723
     137B ->148B         0.10547

Excited State  99:   2.853-A    6.0177 eV  206.03 nm  f=0.0146  <S**2>=1.785
     132A ->143A        -0.18062
     134A ->143A         0.14953
     134A ->146A        -0.14018
     136A ->146A         0.26640
     137A ->144A         0.68184
     138A ->147A         0.13187
     126B ->139B        -0.10119
     134B ->141B        -0.12084
     134B ->143B        -0.28187
     134B ->145B        -0.27772
     137B ->147B         0.13492

Excited State 100:   2.852-A    6.0258 eV  205.75 nm  f=0.0075  <S**2>=1.783
     124B ->139B         0.10399
     125B ->139B        -0.16400
     126B ->139B         0.87172
     128B ->139B        -0.10961
     129B ->139B        -0.36891
```

Figure S24-1. The calculated absorption spectra based on B3LYP level of theory. The upper chart indicates the actual spectrum of **2** (black line) and calculated spectrum of $\mathbf{2_{opt}}$ (red solid bars), whereas the lower chart includes the actual spectrum of **3** (black line) and calculated spectrum of $\mathbf{3_{opt}}$ (red solid bars).

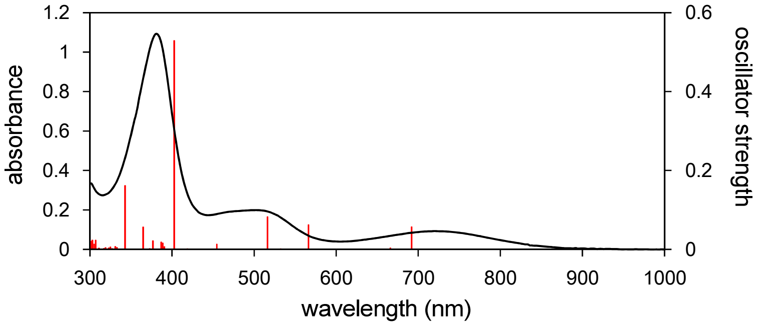

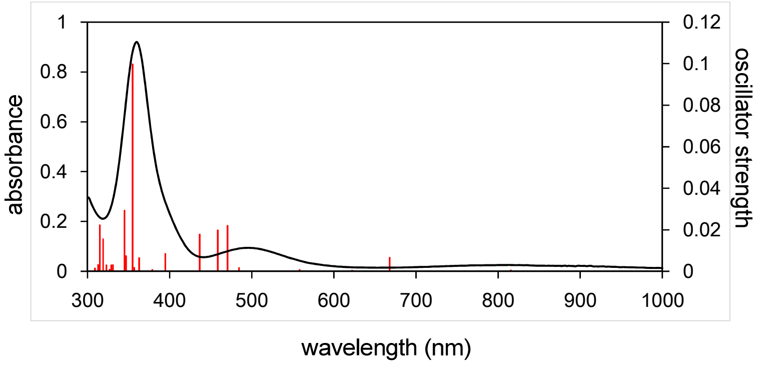

Figure S24-2. The calculated absorption spectra based on CAM-B3LYP level of theory. The upper chart indicates the actual spectrum of **2** (black line) and calculated spectrum of $\mathbf{2_{opt}}$ (red solid bars), whereas the lower chart includes the actual spectrum of **3** (black line) and calculated spectrum of $\mathbf{3_{opt}}$ (red solid bars).

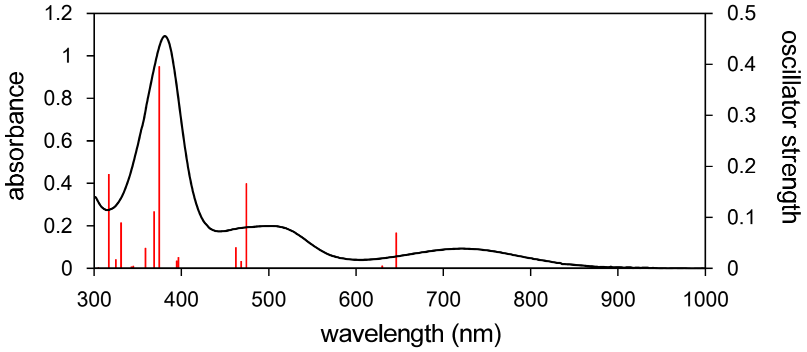

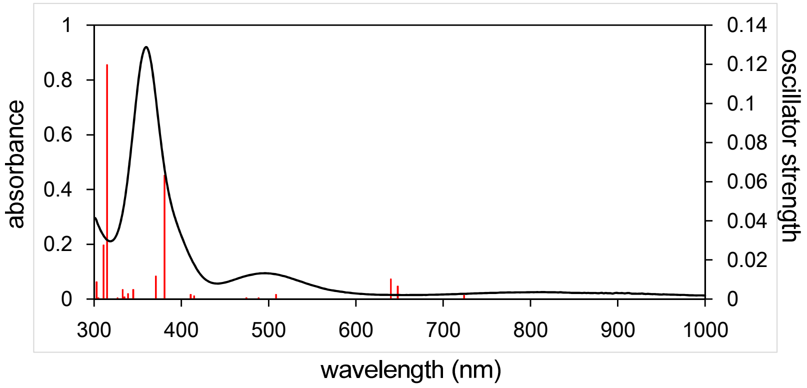

X-ray data collection and reduction

X-ray crystallography was performed on a Rigaku Saturn CCD area detector with graphite monochromated Mo-Kα radiation (λ=0.71075 Å). The data were collected at 123(2) K using ω scan in the θ range of 1.55 ≤ θ≤ 30.60 deg (**2**), 1.88 ≤ θ≤ 30.66 deg (**4**), 2.08 ≤ θ≤ 30.47 deg (**5**) and 3.03 ≤ θ≤ 27.48 deg (**8**). The data obtained were processed using Crystal-Clear (Rigaku) on a Pentium computer, and were corrected for Lorentz and polarization effects. The structures were solved by direct methods[11], and expanded using Fourier techniques[12]. Hydrogen atoms were refined using the riding model. The final cycle of full-matrix least-squares refinement on F^2 was based on 12,402 observed reflections and 505 variable parameters for **2**, 7,658 observed reflections and 325 variable parameters for **4**, 7,170 observed reflections and 289 variable parameters for **5**, and 9,661 observed reflections and 422 variable parameters for **8**. Neutral atom scattering factors were taken from Cromer and Waber[13]. All calculations were performed using SHELXL-97[14]. Details of final refinement as well as the bond lengths and angle are summarized in Tables S7, S8, S9 and S10, and the numbering scheme employed is also shown in Figures S25, 26, 27 and 28, which were drawn with ORTEP at 50% probability ellipsoids.

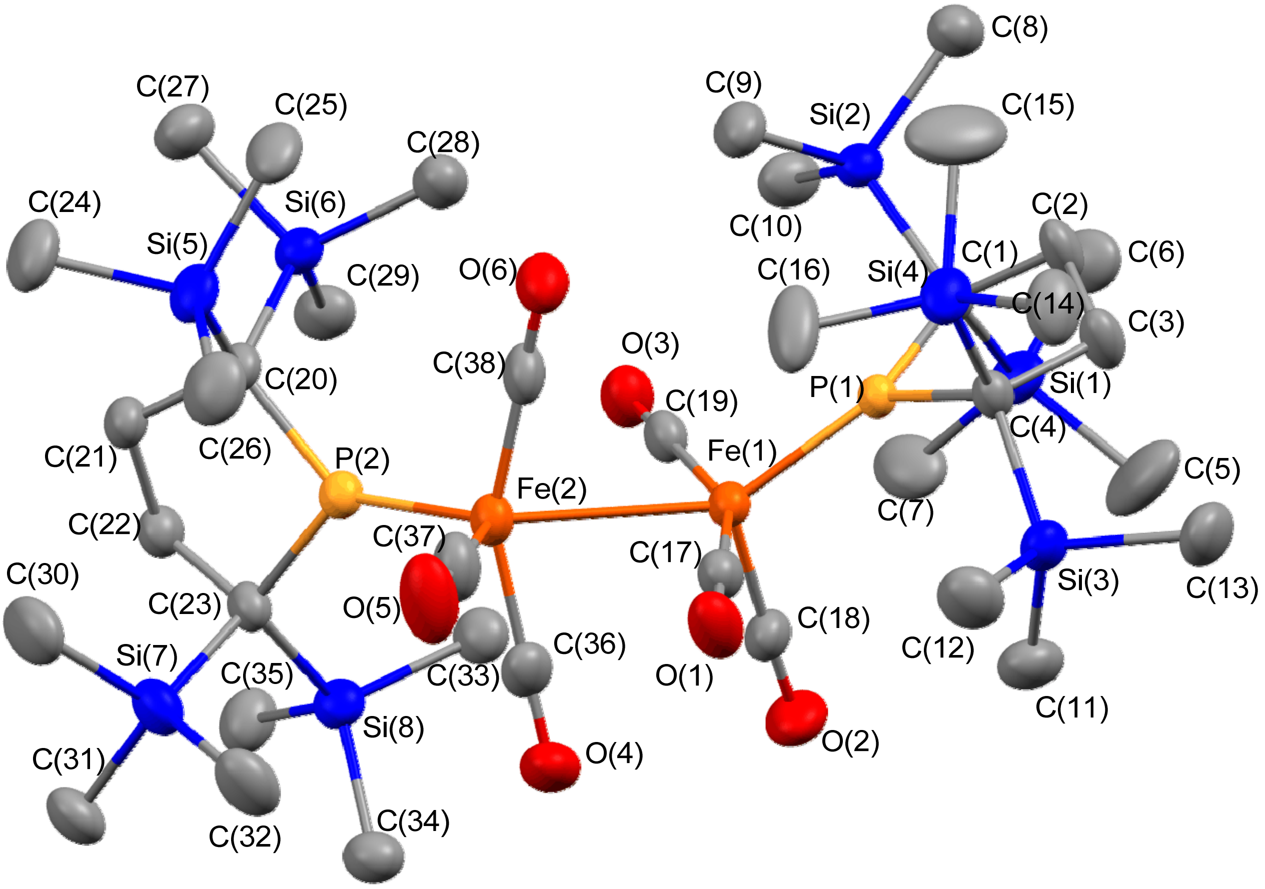

Figure S25. ORTEP drawing of **2** (50% probability of the thermal ellipsoids)

Table S7-1. Crystal data and structure refinement for **2**

Empirical Formula	$C_{38}H_{80}Fe_2O_6P_2Si_8$
Formula Weight	1031.37
Crystal Color, Habit	red, platelet
Crystal Dimensions	0.050 X 0.020 X 0.020 mm
Crystal System	monoclinic
Lattice Type	Primitive
Lattice Parameters	a = 15.6926(12) Å
	b = 17.5019(12) Å
	c = 21.2042(17) Å
	β = 110.6328(14) ^{o}
	V = 5450.2(7) Å^3
Space Group	$P2_1/c$ (#14)
Z value	4
D_{calc}	1.257 g/cm^3
F_{000}	2200.00
μ(MoKα)	8.037 cm^{-1}
Diffractometer	Saturn724
Radiation	MoKα (λ = 0.71075 Å)
	multi-layer mirror monochromated
Voltage, Current	50kV, 40mA
Temperature	123 K
Detector Aperture	72.8 x 72.8 mm
Data Images	720 exposures
ω oscillation Range (χ=45.0, ϕ=0.0)	-105.0 - 75.0^{o}
Exposure Rate	80.0 sec./^{o}
Detector Swing Angle	-14.85^{o}
Detector Position	40.15 mm
Pixel Size	0.070 mm
$2\theta_{max}$	55.0^{o}
No. of Reflections Measured	Total: 52592
	Unique: 12402 (R_{int} = 0.1118)
Corrections	Lorentz-polarization
Structure Solution	Direct Methods (SIR2008)
Refinement	Full-matrix least-squares on F^2
Function Minimized	Σ w $(Fo^2 - Fc^2)^2$
Least Squares Weights	$\omega = 1/ [\sigma^2(Fo^2) + (0.0722 \cdot P)^2 + 10.6473 \cdot P]$
	where $P = (Max(Fo^2,0) + 2Fc^2)/3$
$2\theta_{max}$ cutoff	55.0^{o}
Anomalous Dispersion	All non-hydrogen atoms
No. Observations (All reflections)	12402
No. Variables	505
Reflection/Parameter Ratio	24.56
Residuals: R1 (I>2.00σ(I))	0.0716
Residuals: R (All reflections)	0.0829
Residuals: wR2 (All reflections)	0.1846
Goodness of Fit Indicator	1.092
Max Shift/Error in Final Cycle	0.001
Maximum peak in Final Diff. Map	1.20 e^-/Å^3
Minimum peak in Final Diff. Map	-0.81 e^-/Å^3

Table S7-2. Atomic coordinates and B_{iso}/B_{eq}

atom	x	y	z	B_{eq}
Fe1	0.63753(4)	0.00563(3)	0.74022(3)	2.167(11)
Fe2	0.70293(4)	0.09218(3)	0.65975(3)	2.356(11)
P1	0.61672(6)	0.00639(5)	0.83246(4)	1.804(15)
P2	0.82505(6)	0.08855(5)	0.63974(5)	2.153(16)
Si1	0.68699(8)	-0.15121(6)	0.90833(6)	3.08(2)
Si2	0.80445(7)	-0.00180(6)	0.95545(5)	2.266(17)
Si3	0.39861(7)	0.00480(7)	0.78925(6)	2.74(2)
Si4	0.51607(8)	0.14115(6)	0.87359(6)	2.94(2)
Si5	0.91258(8)	0.25357(6)	0.68062(6)	2.93(2)
Si6	1.01251(7)	0.10723(6)	0.75867(6)	2.517(18)
Si7	0.78921(9)	0.09290(7)	0.48235(6)	2.99(2)
Si8	0.82148(8)	-0.06597(6)	0.55925(6)	2.601(19)
O1	0.4778(2)	0.0917(2)	0.65338(16)	3.97(6)
O2	0.5748(2)	-0.13849(18)	0.67055(18)	4.22(7)
O3	0.8298(2)	-0.04213(18)	0.78622(15)	3.36(5)
O4	0.5927(2)	-0.0219(2)	0.56309(16)	4.00(6)
O5	0.5784(2)	0.2070(2)	0.5779(2)	4.99(8)
O6	0.7448(2)	0.19350(17)	0.77764(17)	3.55(6)
C1	0.6822(2)	-0.0411(2)	0.91341(18)	2.07(5)
C2	0.6277(3)	-0.0221(3)	0.9604(2)	3.13(8)
C3	0.5273(3)	-0.0132(3)	0.9190(2)	3.06(7)
C4	0.5155(2)	0.0331(2)	0.85400(18)	2.16(6)
C5	0.5747(4)	-0.1980(3)	0.8922(4)	5.93(15)
C6	0.7594(4)	-0.1872(3)	0.9946(3)	4.30(10)
C7	0.7306(4)	-0.1901(3)	0.8438(3)	4.54(11)
C8	0.8317(3)	0.0043(3)	1.0487(2)	3.36(8)
C9	0.8975(3)	-0.0616(3)	0.9446(3)	3.53(8)
C10	0.8164(3)	0.0966(2)	0.9267(2)	3.49(8)
C11	0.4048(3)	-0.0822(3)	0.7410(3)	3.95(9)
C12	0.3372(3)	0.0810(3)	0.7281(3)	4.29(10)
C13	0.3218(3)	-0.0195(3)	0.8377(3)	4.35(10)
C14	0.4087(3)	0.1700(3)	0.8855(3)	4.06(10)
C15	0.6026(4)	0.1664(4)	0.9577(3)	6.41(17)
C16	0.5371(4)	0.2006(3)	0.8084(3)	4.90(12)
C17	0.5410(3)	0.0600(2)	0.6888(2)	2.81(7)
C18	0.5997(3)	-0.0814(2)	0.6968(2)	2.97(7)
C19	0.7554(3)	-0.0210(2)	0.76866(19)	2.55(6)
C20	0.9321(3)	0.1446(2)	0.6735(2)	2.43(6)
C21	0.9810(3)	0.1307(2)	0.6214(2)	2.65(6)
C22	0.9520(3)	0.0534(2)	0.5868(2)	2.64(6)
C23	0.8479(3)	0.0432(2)	0.56804(19)	2.40(6)
C24	0.9983(4)	0.3055(3)	0.6535(3)	4.18(10)
C25	0.9271(4)	0.2894(3)	0.7671(3)	3.88(9)
C26	0.7972(3)	0.2878(3)	0.6263(3)	4.24(10)
C27	1.1214(3)	0.1638(3)	0.7852(3)	3.71(8)
C28	0.9650(3)	0.1110(3)	0.8276(2)	3.36(8)
C29	1.0467(3)	0.0060(2)	0.7529(2)	3.33(8)
C30	0.8352(4)	0.1895(3)	0.4748(3)	4.54(10)
C31	0.8175(4)	0.0373(3)	0.4164(2)	3.74(8)
C32	0.6632(3)	0.1049(3)	0.4590(3)	4.24(10)
C33	0.8191(3)	-0.1112(2)	0.6380(2)	3.10(7)
C34	0.7135(3)	-0.0939(3)	0.4897(2)	3.53(8)
C35	0.9165(3)	-0.1150(3)	0.5399(3)	3.75(9)
C36	0.6392(3)	0.0202(3)	0.6017(2)	3.14(7)
C37	0.6261(3)	0.1618(3)	0.6113(3)	3.61(8)
C38	0.7317(3)	0.1508(2)	0.7335(2)	2.87(7)

$B_{eq} = 8/3\ \pi^2(U_{11}(aa^*)^2 + U_{22}(bb^*)^2 + U_{33}(cc^*)^2 + 2U_{12}(aa^*bb^*)\cos\gamma + 2U_{13}(aa^*cc^*)\cos\beta + 2U_{23}(bb^*cc^*)\cos\alpha)$

Table S7-3. Anisotropic displacement parameters

atom	U_{11}	U_{22}	U_{33}	U_{12}	U_{13}	U_{23}
Fe1	0.0290(3)	0.0258(3)	0.0295(3)	-0.00177(19)	0.0127(2)	0.0001(2)
Fe2	0.0304(3)	0.0278(3)	0.0339(3)	0.0025(2)	0.0145(2)	0.0051(2)

P1	0.0231(4)	0.0215(4)	0.0254(4)	0.0008(3)	0.0103(3)	0.0019(3)
P2	0.0291(5)	0.0229(4)	0.0312(5)	0.0015(3)	0.0123(4)	0.0020(4)
Si1	0.0421(6)	0.0233(5)	0.0453(7)	-0.0032(4)	0.0077(5)	0.0070(5)
Si2	0.0276(5)	0.0278(5)	0.0302(5)	-0.0006(4)	0.0096(4)	-0.0028(4)
Si3	0.0247(5)	0.0415(6)	0.0372(6)	-0.0018(4)	0.0102(4)	0.0024(5)
Si4	0.0372(6)	0.0302(5)	0.0509(7)	0.0032(4)	0.0241(5)	-0.0056(5)
Si5	0.0428(6)	0.0208(5)	0.0526(7)	-0.0015(4)	0.0227(5)	0.0022(5)
Si6	0.0304(5)	0.0291(5)	0.0354(6)	-0.0033(4)	0.0107(4)	-0.0011(4)
Si7	0.0468(7)	0.0367(6)	0.0323(6)	0.0080(5)	0.0168(5)	0.0070(5)
Si8	0.0356(6)	0.0252(5)	0.0376(6)	0.0013(4)	0.0124(5)	-0.0008(4)
O1	0.0349(17)	0.073(2)	0.0440(18)	0.0125(15)	0.0154(14)	0.0156(16)
O2	0.064(2)	0.0397(18)	0.060(2)	-0.0163(15)	0.0253(18)	-0.0153(15)
O3	0.0367(16)	0.0479(18)	0.0461(17)	0.0065(13)	0.0184(13)	0.0083(14)
O4	0.0463(19)	0.064(2)	0.0380(17)	-0.0115(16)	0.0106(14)	-0.0070(15)
O5	0.055(2)	0.068(2)	0.076(3)	0.0325(18)	0.0345(19)	0.041(2)
O6	0.0549(19)	0.0364(16)	0.0519(19)	-0.0043(14)	0.0292(16)	-0.0041(14)
C1	0.0281(17)	0.0248(16)	0.0271(17)	0.0012(13)	0.0116(14)	0.0040(13)
C2	0.037(2)	0.058(3)	0.029(2)	0.0092(19)	0.0182(17)	0.0124(18)
C3	0.032(2)	0.054(3)	0.033(2)	0.0093(18)	0.0147(17)	0.0159(18)
C4	0.0250(17)	0.0314(18)	0.0271(17)	0.0038(14)	0.0110(14)	0.0049(14)
C5	0.059(3)	0.044(3)	0.103(5)	-0.021(2)	0.005(3)	0.021(3)
C6	0.064(3)	0.032(2)	0.058(3)	0.005(2)	0.009(2)	0.013(2)
C7	0.083(4)	0.025(2)	0.061(3)	0.005(2)	0.022(3)	-0.002(2)
C8	0.039(2)	0.049(3)	0.037(2)	-0.0026(18)	0.0105(18)	-0.0015(19)
C9	0.030(2)	0.051(3)	0.051(3)	0.0044(18)	0.0115(19)	-0.008(2)
C10	0.052(3)	0.037(2)	0.049(3)	-0.0118(19)	0.023(2)	-0.0042(19)
C11	0.040(2)	0.055(3)	0.049(3)	-0.006(2)	0.008(2)	-0.010(2)
C12	0.042(3)	0.068(3)	0.047(3)	0.009(2)	0.008(2)	0.007(2)
C13	0.037(2)	0.070(3)	0.063(3)	-0.013(2)	0.023(2)	-0.002(3)
C14	0.057(3)	0.039(2)	0.074(3)	0.014(2)	0.042(3)	0.007(2)
C15	0.072(4)	0.069(4)	0.089(5)	0.018(3)	0.012(3)	-0.047(3)
C16	0.091(4)	0.025(2)	0.101(4)	0.002(2)	0.071(4)	0.002(2)
C17	0.035(2)	0.042(2)	0.034(2)	-0.0046(17)	0.0154(17)	-0.0006(17)
C18	0.036(2)	0.039(2)	0.041(2)	-0.0084(17)	0.0180(18)	-0.0023(18)
C19	0.041(2)	0.0284(18)	0.0312(19)	-0.0005(16)	0.0174(17)	0.0043(15)
C20	0.036(2)	0.0223(17)	0.036(2)	-0.0024(14)	0.0151(16)	0.0018(14)
C21	0.035(2)	0.0327(19)	0.037(2)	-0.0040(15)	0.0177(17)	0.0010(16)
C22	0.035(2)	0.033(2)	0.036(2)	0.0005(16)	0.0177(17)	-0.0006(16)
C23	0.039(2)	0.0265(17)	0.0297(18)	-0.0001(15)	0.0167(16)	0.0020(14)
C24	0.060(3)	0.031(2)	0.079(4)	-0.007(2)	0.038(3)	0.005(2)
C25	0.063(3)	0.028(2)	0.066(3)	-0.0113(19)	0.035(3)	-0.013(2)
C26	0.056(3)	0.030(2)	0.077(4)	0.010(2)	0.026(3)	0.014(2)
C27	0.037(2)	0.049(3)	0.054(3)	-0.0066(19)	0.014(2)	-0.006(2)
C28	0.040(2)	0.048(2)	0.038(2)	-0.0031(19)	0.0110(18)	0.0030(19)
C29	0.041(2)	0.037(2)	0.042(2)	0.0039(17)	0.0060(19)	0.0054(18)
C30	0.084(4)	0.045(3)	0.045(3)	0.003(3)	0.025(3)	0.016(2)
C31	0.058(3)	0.055(3)	0.031(2)	0.006(2)	0.018(2)	0.0016(19)
C32	0.051(3)	0.069(3)	0.040(3)	0.022(2)	0.015(2)	0.015(2)
C33	0.045(2)	0.0295(19)	0.044(2)	0.0029(17)	0.0161(19)	0.0031(17)
C34	0.049(3)	0.041(2)	0.040(2)	-0.0027(19)	0.011(2)	-0.0043(18)
C35	0.048(3)	0.034(2)	0.067(3)	0.0005(19)	0.029(2)	-0.001(2)
C36	0.028(2)	0.049(2)	0.041(2)	0.0002(18)	0.0118(17)	0.0034(19)
C37	0.045(2)	0.047(3)	0.053(3)	0.006(2)	0.028(2)	0.012(2)
C38	0.039(2)	0.032(2)	0.046(2)	0.0016(16)	0.0250(19)	0.0077(18)

The general temperature factor expression: $\exp(-2\pi^2(a^{*2}U_{11}h^2 + b^{*2}U_{22}k^2 + c^{*2}U_{33}l^2 + 2a^*b^*U_{12}hk + 2a^*c^*U_{13}hl + 2b^*c^*U_{23}kl))$

Table S7-4. Bond lengths (Å)

atom	atom	distance	atom	atom	distance
Fe1	Fe2	2.7374(10)	Fe1	P1	2.0934(12)
Fe1	C17	1.796(4)	Fe1	C18	1.771(4)
Fe1	C19	1.794(4)	Fe2	P2	2.1047(13)
Fe2	C36	1.799(4)	Fe2	C37	1.766(4)
Fe2	C38	1.790(4)	P1	C1	1.858(3)
P1	C4	1.861(4)	P2	C20	1.857(4)

P2	C23	1.859(5)	Si1	C1	1.933(4)
Si1	C5	1.862(6)	Si1	C6	1.889(5)
Si1	C7	1.860(7)	Si2	C1	1.935(4)
Si2	C8	1.872(5)	Si2	C9	1.875(5)
Si2	C10	1.859(5)	Si3	C4	1.928(3)
Si3	C11	1.856(5)	Si3	C12	1.873(5)
Si3	C13	1.887(7)	Si4	C4	1.936(4)
Si4	C14	1.859(6)	Si4	C15	1.874(6)
Si4	C16	1.850(7)	Si5	C20	1.946(4)
Si5	C24	1.874(6)	Si5	C25	1.873(6)
Si5	C26	1.869(5)	Si6	C20	1.918(4)
Si6	C27	1.881(5)	Si6	C28	1.862(6)
Si6	C29	1.868(4)	Si7	C23	1.931(4)
Si7	C30	1.867(6)	Si7	C31	1.880(6)
Si7	C32	1.873(5)	Si8	C23	1.949(4)
Si8	C33	1.860(5)	Si8	C34	1.877(4)
Si8	C35	1.886(6)	O1	C17	1.154(5)
O2	C18	1.143(5)	O3	C19	1.154(5)
O4	C36	1.151(5)	O5	C37	1.147(6)
O6	C38	1.158(5)	C1	C2	1.561(7)
C2	C3	1.518(5)	C3	C4	1.552(6)
C20	C21	1.571(7)	C21	C22	1.531(5)
C22	C23	1.549(6)			

Table S7-5. Bond angles (^{o})

atom	atom	atom	angle	atom	atom	atom	angle
Fe2	Fe1	P1	142.66(4)	Fe2	Fe1	C17	75.51(16)
Fe2	Fe1	C18	106.41(17)	Fe2	Fe1	C19	77.57(14)
P1	Fe1	C17	100.83(16)	P1	Fe1	C18	110.93(17)
P1	Fe1	C19	99.76(14)	C17	Fe1	C18	94.81(18)
C17	Fe1	C19	153.0(2)	C18	Fe1	C19	93.89(18)
Fe1	Fe2	P2	132.61(4)	Fe1	Fe2	C36	78.40(16)
Fe1	Fe2	C37	114.53(19)	Fe1	Fe2	C38	78.87(15)
P2	Fe2	C36	99.60(16)	P2	Fe2	C37	112.76(19)
P2	Fe2	C38	103.10(15)	C36	Fe2	C37	89.1(2)
C36	Fe2	C38	155.2(2)	C37	Fe2	C38	91.3(2)
Fe1	P1	C1	130.01(14)	Fe1	P1	C4	131.20(11)
C1	P1	C4	97.32(18)	Fe2	P2	C20	132.13(15)
Fe2	P2	C23	129.04(12)	C20	P2	C23	97.55(19)
C1	Si1	C5	113.3(2)	C1	Si1	C6	107.49(18)
C1	Si1	C7	115.9(2)	C5	Si1	C6	103.8(3)
C5	Si1	C7	105.8(3)	C6	Si1	C7	109.7(2)
C1	Si2	C8	109.1(2)	C1	Si2	C9	115.68(18)
C1	Si2	C10	112.13(18)	C8	Si2	C9	105.3(2)
C8	Si2	C10	106.4(2)	C9	Si2	C10	107.7(2)
C4	Si3	C11	112.43(19)	C4	Si3	C12	116.0(2)
C4	Si3	C13	107.5(2)	C11	Si3	C12	108.4(2)
C11	Si3	C13	106.4(3)	C12	Si3	C13	105.4(3)
C4	Si4	C14	111.0(2)	C4	Si4	C15	112.4(2)
C4	Si4	C16	112.2(2)	C14	Si4	C15	101.2(3)
C14	Si4	C16	111.0(3)	C15	Si4	C16	108.5(3)
C20	Si5	C24	107.6(2)	C20	Si5	C25	115.86(19)
C20	Si5	C26	114.24(18)	C24	Si5	C25	106.5(2)
C24	Si5	C26	107.5(2)	C25	Si5	C26	104.7(3)
C20	Si6	C27	109.3(2)	C20	Si6	C28	114.5(2)
C20	Si6	C29	111.75(18)	C27	Si6	C28	108.6(2)
C27	Si6	C29	105.5(2)	C28	Si6	C29	106.8(2)
C23	Si7	C30	114.71(19)	C23	Si7	C31	107.9(2)
C23	Si7	C32	114.1(2)	C30	Si7	C31	101.9(3)
C30	Si7	C32	106.3(3)	C31	Si7	C32	111.4(2)
C23	Si8	C33	113.70(19)	C23	Si8	C34	115.68(18)
C23	Si8	C35	107.9(2)	C33	Si8	C34	107.2(2)
C33	Si8	C35	105.6(2)	C34	Si8	C35	105.9(2)
P1	C1	Si1	114.49(18)	P1	C1	Si2	113.7(2)
P1	C1	C2	104.2(2)	Si1	C1	Si2	109.17(17)
Si1	C1	C2	106.8(3)	Si2	C1	C2	107.9(2)
C1	C2	C3	110.0(3)	C2	C3	C4	110.0(4)
P1	C4	Si3	116.2(2)	P1	C4	Si4	111.0(2)
P1	C4	C3	104.0(3)	Si3	C4	Si4	109.57(17)

Si3	C4	C3	106.5(3)	Si4	C4	C3	109.2(3)
Fe1	C17	O1	176.3(4)	Fe1	C18	O2	177.8(5)
Fe1	C19	O3	176.4(3)	P2	C20	Si5	113.7(2)
P2	C20	Si6	112.9(2)	P2	C20	C21	104.4(2)
Si5	C20	Si6	109.26(18)	Si5	C20	C21	109.6(3)
Si6	C20	C21	106.7(2)	C20	C21	C22	109.7(3)
C21	C22	C23	109.5(3)	P2	C23	Si7	114.3(2)
P2	C23	Si8	113.6(2)	P2	C23	C22	103.3(2)
Si7	C23	Si8	109.68(17)	Si7	C23	C22	107.4(3)
Si8	C23	C22	107.9(3)	Fe2	C36	O4	174.5(4)
Fe2	C37	O5	176.9(5)	Fe2	C38	O6	173.9(3)

Table S7-6. Torsion Angles(°)

(Those having bond angles > 160 or < 20 degrees are excluded.)

atom1	atom2	atom3	atom4	angle	atom1	atom2	atom3	atom4	angle
Fe2	Fe1	P1	C1	-103.17(7)	Fe2	Fe1	P1	C4	93.84(8)
P1	Fe1	Fe2	P2	103.37(6)	P1	Fe1	Fe2	C36	-164.20(6)
P1	Fe1	Fe2	C37	-80.58(6)	P1	Fe1	Fe2	C38	5.74(6)
C17	Fe1	Fe2	P2	-167.71(14)	C17	Fe1	Fe2	C36	-75.28(14)
C17	Fe1	Fe2	C37	8.34(14)	C17	Fe1	Fe2	C38	94.66(14)
C18	Fe1	Fe2	P2	-76.90(14)	C18	Fe1	Fe2	C36	15.54(14)
C18	Fe1	Fe2	C37	99.15(14)	C18	Fe1	Fe2	C38	-174.53(14)
C19	Fe1	Fe2	P2	13.53(13)	C19	Fe1	Fe2	C36	105.96(13)
C19	Fe1	Fe2	C37	-170.42(13)	C19	Fe1	Fe2	C38	-84.10(13)
C17	Fe1	P1	C1	176.58(14)	C17	Fe1	P1	C4	13.59(15)
C18	Fe1	P1	C1	77.11(15)	C18	Fe1	P1	C4	-85.88(15)
C19	Fe1	P1	C1	-20.91(14)	C19	Fe1	P1	C4	176.10(14)
Fe1	Fe2	P2	C20	-98.96(7)	Fe1	Fe2	P2	C23	97.01(7)
C36	Fe2	P2	C20	178.02(15)	C36	Fe2	P2	C23	13.99(16)
C37	Fe2	P2	C20	84.93(18)	C37	Fe2	P2	C23	-79.09(18)
C38	Fe2	P2	C20	-12.14(15)	C38	Fe2	P2	C23	-176.16(14)
Fe1	P1	C1	Si1	-60.1(3)	Fe1	P1	C1	Si2	66.3(2)
Fe1	P1	C1	C2	-176.46(8)	Fe1	P1	C4	Si3	37.6(3)
Fe1	P1	C4	Si4	-88.40(18)	Fe1	P1	C4	C3	154.28(10)
C1	P1	C4	Si3	-129.3(2)	C1	P1	C4	Si4	104.65(18)
C1	P1	C4	C3	-12.7(2)	C4	P1	C1	Si1	107.0(2)
C4	P1	C1	Si2	-126.5(2)	C4	P1	C1	C2	-9.3(2)
Fe2	P2	C20	Si5	-43.3(3)	Fe2	P2	C20	Si6	81.9(2)
Fe2	P2	C20	C21	-162.67(9)	Fe2	P2	C23	Si7	69.4(2)
Fe2	P2	C23	Si8	-57.5(2)	Fe2	P2	C23	C22	-174.21(9)
C20	P2	C23	Si7	-98.7(2)	C20	P2	C23	Si8	134.35(19)
C20	P2	C23	C22	17.7(2)	C23	P2	C20	Si5	124.2(2)
C23	P2	C20	Si6	-110.6(2)	C23	P2	C20	C21	4.9(2)
C5	Si1	C1	P1	-67.7(4)	C5	Si1	C1	Si2	163.5(3)
C5	Si1	C1	C2	47.1(3)	C6	Si1	C1	P1	178.1(3)
C6	Si1	C1	Si2	49.4(3)	C6	Si1	C1	C2	-67.0(3)
C7	Si1	C1	P1	55.0(3)	C7	Si1	C1	Si2	-73.8(3)
C7	Si1	C1	C2	169.8(2)	C8	Si2	C1	P1	140.2(2)
C8	Si2	C1	Si1	-90.6(2)	C8	Si2	C1	C2	25.1(3)
C9	Si2	C1	P1	-101.4(3)	C9	Si2	C1	Si1	27.8(3)
C9	Si2	C1	C2	143.5(2)	C10	Si2	C1	P1	22.6(3)
C10	Si2	C1	Si1	151.8(2)	C10	Si2	C1	C2	-92.5(3)
C11	Si3	C4	P1	29.2(3)	C11	Si3	C4	Si4	155.9(2)
C11	Si3	C4	C3	-86.1(3)	C12	Si3	C4	P1	-96.5(3)
C12	Si3	C4	Si4	30.2(3)	C12	Si3	C4	C3	148.3(3)
C13	Si3	C4	P1	145.9(2)	C13	Si3	C4	Si4	-87.4(3)
C13	Si3	C4	C3	30.7(3)	C14	Si4	C4	P1	173.5(2)
C14	Si4	C4	Si3	43.9(3)	C14	Si4	C4	C3	-72.4(3)
C15	Si4	C4	P1	-73.9(3)	C15	Si4	C4	Si3	156.5(3)
C15	Si4	C4	C3	40.2(4)	C16	Si4	C4	P1	48.7(3)
C16	Si4	C4	Si3	-80.8(3)	C16	Si4	C4	C3	162.8(2)
C24	Si5	C20	P2	-139.8(2)	C24	Si5	C20	Si6	93.1(3)
C24	Si5	C20	C21	-23.4(3)	C25	Si5	C20	P2	101.2(3)
C25	Si5	C20	Si6	-25.9(3)	C25	Si5	C20	C21	-142.4(2)
C26	Si5	C20	P2	-20.6(4)	C26	Si5	C20	Si6	-147.7(3)
C26	Si5	C20	C21	95.8(3)	C27	Si6	C20	P2	176.3(2)
C27	Si6	C20	Si5	-56.1(3)	C27	Si6	C20	C21	62.3(3)
C28	Si6	C20	P2	-61.6(3)	C28	Si6	C20	Si5	65.9(3)
C28	Si6	C20	C21	-175.7(2)	C29	Si6	C20	P2	60.0(3)

C29	Si6	C20	Si5	-172.5(2)	C29	Si6	C20	C21	-54.1(3)
C30	Si7	C23	P2	69.0(3)	C30	Si7	C23	Si8	-162.1(3)
C30	Si7	C23	C22	-45.0(3)	C31	Si7	C23	P2	-178.3(2)
C31	Si7	C23	Si8	-49.4(3)	C31	Si7	C23	C22	67.7(3)
C32	Si7	C23	P2	-54.0(3)	C32	Si7	C23	Si8	75.0(3)
C32	Si7	C23	C22	-168.0(2)	C33	Si8	C23	P2	-20.8(3)
C33	Si8	C23	Si7	-150.1(2)	C33	Si8	C23	C22	93.2(3)
C34	Si8	C23	P2	104.0(3)	C34	Si8	C23	Si7	-25.3(3)
C34	Si8	C23	C22	-142.1(2)	C35	Si8	C23	P2	-137.6(2)
C35	Si8	C23	Si7	93.1(2)	C35	Si8	C23	C22	-23.7(3)
P1	C1	C2	C3	30.0(4)	Si1	C1	C2	C3	-91.5(3)
Si2	C1	C2	C3	151.2(2)	C1	C2	C3	C4	-42.4(5)
C2	C3	C4	P1	32.5(4)	C2	C3	C4	Si3	155.8(3)
C2	C3	C4	Si4	-86.0(4)	P2	C20	C21	C22	-27.2(3)
Si5	C20	C21	C22	-149.30(19)	Si6	C20	C21	C22	92.5(2)
C20	C21	C22	C23	43.2(4)	C21	C22	C23	P2	-36.6(4)
C21	C22	C23	Si7	84.6(3)	C21	C22	C23	Si8	-157.2(3)

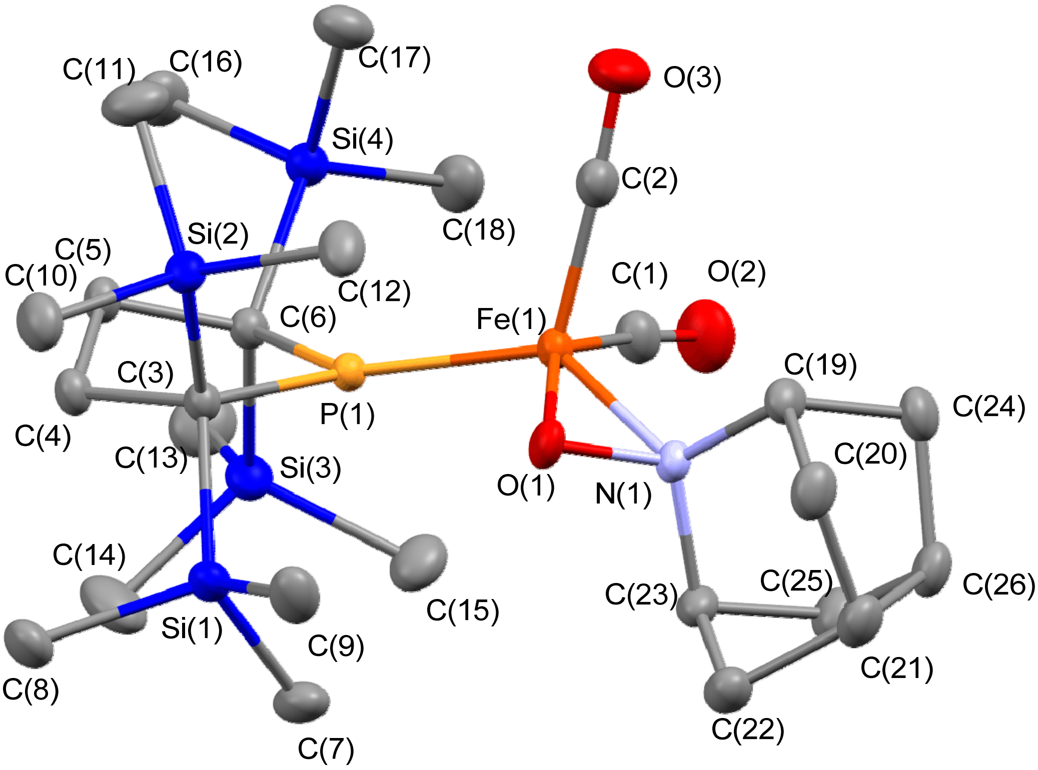

Figure S26. ORTEP drawing of **4** (50% probability of the thermal ellipsoids)

Table S8-1. Crystal data and structure refinement for **4**

Empirical Formula	$C_{26}H_{52}FeNO_3PSi_4$
Formula Weight	625.86
Crystal Color, Habit	palegreen, platelet
Crystal Dimensions	0.100 X 0.080 X 0.050 mm
Crystal System	triclinic
Lattice Type	Primitive
Lattice Parameters	a = 9.4542(8) Å
	b = 11.3877(10) Å
	c = 16.3807(15) Å
	α = 75.408(5) ^{o}
	β = 87.846(7) ^{o}
	γ = 79.154(6) ^{o}
	V = 1676.1(3) Å^3
Space Group	P-1 (#2)
Z value	2
D_{calc}	1.240 g/cm^3
F_{000}	672.00
μ(MoKα)	6.662 cm^{-1}
Diffractometer	Saturn724
Radiation	MoKα (λ = 0.71075 Å)
	multi-layer mirror monochromated
Voltage, Current	50kV, 40mA
Temperature	123 K
Detector Aperture	72.8 x 72.8 mm
Data Images	1080 exposures
ω oscillation Range (χ=45.0, ϕ=0.0)	-105.0 - 75.0^o
Exposure Rate	32.0 sec./^{o}
Detector Swing Angle	-14.70^o
Detector Position	40.10 mm
Pixel Size	0.070 mm
$2\theta_{max}$	55.0^o
No. of Reflections Measured	Total: 24288
	Unique: 7658 (R_{int} = 0.0235)
Corrections	Lorentz-polarization
	Absorption
	(trans. factors: 0.851 - 0.967)
Structure Solution	Direct Methods (SIR2008)
Refinement	Full-matrix least-squares on F^2
Function Minimized	Σ w (Fo^2 - Fc^2)^2
Least Squares Weights	$\omega = 1/ [\sigma^2(Fo^2) + (0.0455 \cdot P)^2 + 0.9471 \cdot P]$
	where P = (Max(Fo^2,0) + 2Fc^2)/3
$2\theta_{max}$ cutoff	55.0^o
Anomalous Dispersion	All non-hydrogen atoms
No. Observations (All reflections)	7658
No. Variables	325
Reflection/Parameter Ratio	23.56
Residuals: R1 (I>2.00σ(I))	0.0318
Residuals: R (All reflections)	0.0328
Residuals: wR2 (All reflections)	0.0855
Goodness of Fit Indicator	1.069
Max Shift/Error in Final Cycle	0.001
Maximum peak in Final Diff. Map	0.46 e$^-$/Å^3
Minimum peak in Final Diff. Map	-0.45 e$^-$/Å^3

Table S8-2. Atomic coordinates and B_{iso}/B_{eq}

atom	x	y	z	B_{eq}
Fe1	0.29167(2)	-0.001720(17)	0.292965(12)	1.258(5)
P1	0.20257(4)	0.18630(3)	0.23918(2)	1.163(6)
Si1	0.36415(4)	0.27691(4)	0.07178(3)	1.547(7)
Si2	0.07636(4)	0.17867(4)	0.06396(2)	1.431(7)
Si3	0.20217(5)	0.40374(4)	0.32197(3)	1.683(7)
Si4	-0.05414(4)	0.25412(4)	0.36025(2)	1.517(7)
O1	0.40689(11)	-0.03521(10)	0.19880(6)	1.561(17)
O2	0.30749(16)	-0.01942(13)	0.47266(8)	3.08(2)
O3	0.07799(13)	-0.16114(12)	0.31119(9)	2.87(2)
N1	0.47381(12)	-0.10214(11)	0.27445(7)	1.282(17)
C1	0.29260(17)	-0.00579(14)	0.40099(10)	1.94(2)
C2	0.16602(17)	-0.10172(14)	0.30349(10)	1.92(2)
C3	0.18013(14)	0.26033(12)	0.12445(8)	1.23(2)
C4	0.09146(16)	0.39269(13)	0.11973(9)	1.49(2)
C5	-0.00158(15)	0.39199(13)	0.19848(9)	1.49(2)
C6	0.08590(15)	0.31063(12)	0.27849(9)	1.29(2)
C7	0.50149(17)	0.28705(17)	0.14797(11)	2.30(3)
C8	0.33835(18)	0.42468(15)	-0.01431(10)	2.27(3)
C9	0.44820(19)	0.14934(16)	0.02336(10)	2.28(3)
C10	0.07256(19)	0.25885(15)	-0.05165(10)	2.12(3)
C11	-0.11714(17)	0.19451(18)	0.09453(12)	2.49(3)
C12	0.14916(19)	0.01096(14)	0.07665(10)	2.10(3)
C13	0.0996(2)	0.49375(18)	0.39356(13)	2.97(3)
C14	0.2624(3)	0.52835(18)	0.23730(13)	3.24(4)
C15	0.36279(19)	0.30283(17)	0.38228(12)	2.57(3)
C16	-0.20532(18)	0.38722(16)	0.36026(12)	2.51(3)
C17	-0.14235(18)	0.13730(16)	0.33055(11)	2.30(3)
C18	0.01604(19)	0.18980(17)	0.47072(10)	2.46(3)
C19	0.48657(16)	-0.23851(13)	0.28429(9)	1.59(2)
C20	0.60589(17)	-0.28277(15)	0.22754(10)	2.01(3)
C21	0.73649(17)	-0.25202(15)	0.26566(10)	2.02(3)
C22	0.72857(17)	-0.11198(15)	0.23060(10)	2.09(3)
C23	0.61294(15)	-0.06291(13)	0.28748(9)	1.59(2)
C24	0.55011(18)	-0.30574(14)	0.37134(10)	1.97(2)
C25	0.67368(17)	-0.13552(15)	0.37431(10)	1.95(2)
C26	0.69900(17)	-0.26774(15)	0.36295(10)	1.97(2)

$B_{eq} = 8/3\ \pi^2(U_{11}(aa^*)^2 + U_{22}(bb^*)^2 + U_{33}(cc^*)^2 + 2U_{12}(aa^*bb^*)\cos\gamma + 2U_{13}(aa^*cc^*)\cos\beta + 2U_{23}(bb^*cc^*)\cos\alpha)$

Table S8-3. Anisotropic displacement parameters

atom	U_{11}	U_{22}	U_{33}	U_{12}	U_{13}	U_{23}
Fe1	0.01673(11)	0.01323(10)	0.01645(11)	-0.00078(7)	-0.00029(7)	-0.00254(8)
P1	0.01487(16)	0.01270(16)	0.01569(16)	-0.00112(12)	-0.00006(12)	-0.00285(12)
Si1	0.01815(19)	0.01960(19)	0.01925(19)	-0.00364(15)	0.00332(14)	-0.00196(15)
Si2	0.01924(19)	0.01772(19)	0.01783(18)	-0.00287(14)	-0.00168(14)	-0.00534(15)
Si3	0.0245(2)	0.01868(19)	0.0231(2)	-0.00532(16)	-0.00122(16)	-0.00817(16)
Si4	0.01850(19)	0.01891(19)	0.01882(19)	-0.00132(15)	0.00288(14)	-0.00411(15)
O1	0.0195(5)	0.0210(5)	0.0151(5)	0.0030(4)	-0.0037(4)	-0.0021(4)
O2	0.0513(8)	0.0422(7)	0.0205(6)	-0.0005(6)	0.0011(5)	-0.0081(5)
O3	0.0280(6)	0.0309(6)	0.0518(8)	-0.0131(5)	0.0024(6)	-0.0078(6)
N1	0.0167(5)	0.0147(5)	0.0160(5)	-0.0005(4)	-0.0032(4)	-0.0029(4)
C1	0.0270(8)	0.0205(7)	0.0240(7)	-0.0002(6)	0.0024(6)	-0.0048(6)
C2	0.0226(7)	0.0199(7)	0.0272(8)	0.0003(6)	-0.0005(6)	-0.0033(6)
C3	0.0165(6)	0.0139(6)	0.0153(6)	-0.0015(5)	0.0002(5)	-0.0029(5)
C4	0.0222(7)	0.0146(6)	0.0178(6)	-0.0010(5)	-0.0003(5)	-0.0022(5)
C5	0.0194(7)	0.0155(6)	0.0194(7)	0.0007(5)	-0.0006(5)	-0.0030(5)
C6	0.0175(6)	0.0139(6)	0.0175(6)	-0.0012(5)	0.0004(5)	-0.0047(5)
C7	0.0207(7)	0.0369(9)	0.0296(8)	-0.0106(6)	0.0014(6)	-0.0040(7)
C8	0.0292(8)	0.0261(8)	0.0273(8)	-0.0082(6)	0.0045(6)	0.0017(6)
C9	0.0305(8)	0.0277(8)	0.0245(8)	0.0001(6)	0.0091(6)	-0.0044(6)
C10	0.0351(9)	0.0251(8)	0.0193(7)	-0.0037(6)	-0.0047(6)	-0.0042(6)
C11	0.0213(7)	0.0440(10)	0.0362(9)	-0.0101(7)	0.0014(6)	-0.0197(8)
C12	0.0338(8)	0.0192(7)	0.0283(8)	-0.0038(6)	-0.0093(6)	-0.0078(6)

C13	0.0408(10)	0.0361(10)	0.0435(10)	-0.0040(8)	0.0008(8)	-0.0263(8)
C14	0.0570(12)	0.0343(10)	0.0386(10)	-0.0276(9)	-0.0012(9)	-0.0075(8)
C15	0.0297(8)	0.0334(9)	0.0380(9)	-0.0037(7)	-0.0102(7)	-0.0150(7)
C16	0.0256(8)	0.0295(8)	0.0370(9)	0.0026(7)	0.0078(7)	-0.0089(7)
C17	0.0265(8)	0.0294(8)	0.0345(9)	-0.0112(6)	0.0089(7)	-0.0103(7)
C18	0.0314(9)	0.0381(9)	0.0199(7)	-0.0033(7)	0.0023(6)	-0.0024(7)
C19	0.0211(7)	0.0141(6)	0.0255(7)	-0.0014(5)	-0.0016(6)	-0.0067(5)
C20	0.0287(8)	0.0220(7)	0.0245(7)	0.0043(6)	-0.0015(6)	-0.0101(6)
C21	0.0207(7)	0.0254(8)	0.0275(8)	0.0040(6)	-0.0008(6)	-0.0066(6)
C22	0.0196(7)	0.0282(8)	0.0282(8)	-0.0021(6)	0.0014(6)	-0.0027(6)
C23	0.0177(7)	0.0179(7)	0.0246(7)	-0.0033(5)	-0.0043(5)	-0.0044(6)
C24	0.0303(8)	0.0160(7)	0.0249(7)	0.0004(6)	-0.0003(6)	-0.0015(6)
C25	0.0249(7)	0.0242(7)	0.0246(7)	0.0002(6)	-0.0089(6)	-0.0072(6)
C26	0.0244(7)	0.0225(7)	0.0241(7)	0.0041(6)	-0.0059(6)	-0.0038(6)

The general temperature factor expression: $\exp(-2\pi^2(a^{*2}U_{11}h^2 + b^{*2}U_{22}k^2 + c^{*2}U_{33}l^2 + 2a^*b^*U_{12}hk + 2a^*c^*U_{13}hl + 2b^*c^*U_{23}kl))$

Table S8-4. Bond lengths (Å)

atom	atom	distance	atom	atom	distance
Fe1	P1	2.1205(4)	Fe1	O1	1.9300(11)
Fe1	N1	1.9389(11)	Fe1	C1	1.7587(17)
Fe1	C2	1.7699(18)	P1	C3	1.8580(13)
P1	C6	1.8585(15)	Si1	C3	1.9327(14)
Si1	C7	1.8729(19)	Si1	C8	1.8856(15)
Si1	C9	1.8627(19)	Si2	C3	1.9209(16)
Si2	C10	1.8822(15)	Si2	C11	1.8661(17)
Si2	C12	1.8656(16)	Si3	C6	1.9282(17)
Si3	C13	1.874(2)	Si3	C14	1.875(2)
Si3	C15	1.8672(17)	Si4	C6	1.9271(15)
Si4	C16	1.8775(17)	Si4	C17	1.864(2)
Si4	C18	1.8684(16)	O1	N1	1.3851(14)
O2	C1	1.155(2)	O3	C2	1.152(2)
N1	C19	1.502(2)	N1	C23	1.505(2)
C3	C4	1.5656(19)	C4	C5	1.533(2)
C5	C6	1.5625(18)	C19	C20	1.528(2)
C19	C24	1.524(2)	C20	C21	1.539(3)
C21	C22	1.541(2)	C21	C26	1.592(2)
C22	C23	1.527(2)	C23	C25	1.525(2)
C24	C26	1.540(2)	C25	C26	1.537(3)

Table S8-5. Bond angles (°)

atom	atom	atom	angle	atom	atom	atom	angle
P1	Fe1	O1	98.58(3)	P1	Fe1	N1	131.31(4)
P1	Fe1	C1	101.54(5)	P1	Fe1	C2	113.69(5)
O1	Fe1	N1	41.95(4)	O1	Fe1	C1	145.35(6)
O1	Fe1	C2	103.06(7)	N1	Fe1	C1	104.91(6)
N1	Fe1	C2	104.29(6)	C1	Fe1	C2	94.29(8)
Fe1	P1	C3	125.47(5)	Fe1	P1	C6	134.73(4)
C3	P1	C6	97.90(6)	C3	Si1	C7	112.10(7)
C3	Si1	C8	108.15(6)	C3	Si1	C9	115.42(8)
C7	Si1	C8	107.62(8)	C7	Si1	C9	105.71(8)
C8	Si1	C9	107.51(7)	C3	Si2	C10	109.17(7)
C3	Si2	C11	111.35(8)	C3	Si2	C12	115.13(7)
C10	Si2	C11	104.42(8)	C10	Si2	C12	108.62(8)
C11	Si2	C12	107.58(9)	C6	Si3	C13	112.68(8)
C6	Si3	C14	112.65(9)	C6	Si3	C15	112.17(8)
C13	Si3	C14	102.11(9)	C13	Si3	C15	107.35(9)
C14	Si3	C15	109.29(9)	C6	Si4	C16	108.57(7)
C6	Si4	C17	112.13(7)	C6	Si4	C18	115.00(7)
C16	Si4	C17	104.29(8)	C16	Si4	C18	107.90(8)
C17	Si4	C18	108.34(8)	Fe1	O1	N1	69.37(6)
Fe1	N1	O1	68.68(6)	Fe1	N1	C19	120.69(9)
Fe1	N1	C23	119.89(10)	O1	N1	C19	111.16(12)
O1	N1	C23	112.14(10)	C19	N1	C23	114.32(10)

Fe1	C1	O2	171.85(14)	Fe1	C2	O3	175.90(14)
P1	C3	Si1	111.08(7)	P1	C3	Si2	114.41(7)
P1	C3	C4	104.54(9)	Si1	C3	Si2	109.59(7)
Si1	C3	C4	108.35(9)	Si2	C3	C4	108.58(9)
C3	C4	C5	110.21(10)	C4	C5	C6	109.91(11)
P1	C6	Si3	110.30(7)	P1	C6	Si4	114.38(7)
P1	C6	C5	103.64(10)	Si3	C6	Si4	110.79(8)
Si3	C6	C5	111.29(10)	Si4	C6	C5	106.17(9)
N1	C19	C20	109.70(12)	N1	C19	C24	108.32(13)
C20	C19	C24	100.99(11)	C19	C20	C21	100.17(14)
C20	C21	C22	105.77(12)	C20	C21	C26	104.37(13)
C22	C21	C26	104.36(14)	C21	C22	C23	100.19(12)
N1	C23	C22	109.60(13)	N1	C23	C25	108.37(11)
C22	C23	C25	100.73(11)	C19	C24	C26	100.28(12)
C23	C25	C26	100.47(14)	C21	C26	C24	104.30(14)
C21	C26	C25	104.17(12)	C24	C26	C25	105.88(13)

Table S8-6. Torsion Angles(^{o})

(Those having bond angles > 160 or < 20 degrees are excluded.)

atom1	atom2	atom3	atom4	angle	atom1	atom2	atom3	atom4	angle
P1	Fe1	O1	N1	146.18(4)	O1	Fe1	P1	C3	24.26(4)
O1	Fe1	P1	C6	-175.10(4)	P1	Fe1	N1	O1	-47.12(8)
P1	Fe1	N1	C19	-149.77(5)	P1	Fe1	N1	C23	56.90(9)
N1	Fe1	P1	C3	53.95(6)	N1	Fe1	P1	C6	-145.41(6)
C1	Fe1	P1	C3	175.89(6)	C1	Fe1	P1	C6	-23.47(6)
C2	Fe1	P1	C3	-84.15(7)	C2	Fe1	P1	C6	76.49(7)
O1	Fe1	N1	O1	-0.00(5)	O1	Fe1	N1	C19	-102.65(11)
O1	Fe1	N1	C23	104.02(10)	N1	Fe1	O1	N1	-0.00(6)
C1	Fe1	O1	N1	21.13(13)	C2	Fe1	O1	N1	-96.95(6)
C1	Fe1	N1	O1	-167.75(7)	C1	Fe1	N1	C19	89.60(9)
C1	Fe1	N1	C23	-63.74(9)	C2	Fe1	N1	O1	93.75(7)
C2	Fe1	N1	C19	-8.90(10)	C2	Fe1	N1	C23	-162.23(8)
Fe1	P1	C3	Si1	-70.83(9)	Fe1	P1	C3	Si2	53.89(8)
Fe1	P1	C3	C4	172.52(4)	Fe1	P1	C6	Si3	91.75(8)
Fe1	P1	C6	Si4	-33.92(12)	Fe1	P1	C6	C5	-149.04(4)
C3	P1	C6	Si3	-104.07(7)	C3	P1	C6	Si4	130.26(8)
C3	P1	C6	C5	15.14(9)	C6	P1	C3	Si1	122.93(8)
C6	P1	C3	Si2	-112.36(8)	C6	P1	C3	C4	6.27(9)
C7	Si1	C3	P1	-26.85(10)	C7	Si1	C3	Si2	-154.24(7)
C7	Si1	C3	C4	87.44(9)	C8	Si1	C3	P1	-145.32(8)
C8	Si1	C3	Si2	87.29(8)	C8	Si1	C3	C4	-31.03(10)
C9	Si1	C3	P1	94.26(9)	C9	Si1	C3	Si2	-33.13(8)
C9	Si1	C3	C4	-151.45(8)	C10	Si2	C3	P1	-176.65(7)
C10	Si2	C3	Si1	-51.15(8)	C10	Si2	C3	C4	67.03(9)
C11	Si2	C3	P1	68.58(9)	C11	Si2	C3	Si1	-165.92(7)
C11	Si2	C3	C4	-47.74(9)	C12	Si2	C3	P1	-54.20(9)
C12	Si2	C3	Si1	71.29(8)	C12	Si2	C3	C4	-170.53(7)
C13	Si3	C6	P1	-161.09(7)	C13	Si3	C6	Si4	-33.42(9)
C13	Si3	C6	C5	84.46(10)	C14	Si3	C6	P1	84.02(9)
C14	Si3	C6	Si4	-148.30(8)	C14	Si3	C6	C5	-30.43(11)
C15	Si3	C6	P1	-39.81(10)	C15	Si3	C6	Si4	87.87(9)
C15	Si3	C6	C5	-154.25(9)	C16	Si4	C6	P1	-157.32(9)
C16	Si4	C6	Si3	77.26(9)	C16	Si4	C6	C5	-43.69(11)
C17	Si4	C6	P1	-42.63(10)	C17	Si4	C6	Si3	-168.05(6)
C17	Si4	C6	C5	71.00(9)	C18	Si4	C6	P1	81.72(11)
C18	Si4	C6	Si3	-43.69(10)	C18	Si4	C6	C5	-164.65(9)
Fe1	O1	N1	Fe1	0.000(11)	Fe1	O1	N1	C19	115.88(8)
Fe1	O1	N1	C23	-114.76(9)	Fe1	N1	C19	C20	152.05(7)
Fe1	N1	C19	C24	-98.57(10)	Fe1	N1	C23	C22	-151.82(7)
Fe1	N1	C23	C25	99.12(9)	O1	N1	C19	C20	74.97(12)
O1	N1	C19	C24	-175.65(9)	O1	N1	C23	C22	-74.46(12)
O1	N1	C23	C25	176.48(9)	C19	N1	C23	C22	53.24(13)
C19	N1	C23	C25	-55.82(14)	C23	N1	C19	C20	-53.23(14)
C23	N1	C19	C24	56.15(13)	P1	C3	C4	C5	-27.19(13)
Si1	C3	C4	C5	-145.71(9)	Si2	C3	C4	C5	95.33(10)
C3	C4	C5	C6	41.11(16)	C4	C5	C6	P1	-33.54(14)
C4	C5	C6	Si3	84.99(13)	C4	C5	C6	Si4	-154.38(11)
N1	C19	C20	C21	62.04(12)	N1	C19	C24	C26	-63.15(13)
C20	C19	C24	C26	52.07(14)	C24	C19	C20	C21	-52.14(13)

C19	C20	C21	C22	-78.51(12)	C19	C20	C21	C26	31.27(12)
C20	C21	C22	C23	78.60(14)	C20	C21	C26	C24	-0.14(14)
C20	C21	C26	C25	-110.96(12)	C22	C21	C26	C24	110.67(12)
C22	C21	C26	C25	-0.15(14)	C26	C21	C22	C23	-31.18(14)
C21	C22	C23	N1	-62.01(13)	C21	C22	C23	C25	52.07(14)
N1	C23	C25	C26	62.65(13)	C22	C23	C25	C26	-52.35(13)
C19	C24	C26	C21	-31.15(13)	C19	C24	C26	C25	78.43(13)
C23	C25	C26	C21	31.50(13)	C23	C25	C26	C24	-78.17(12)

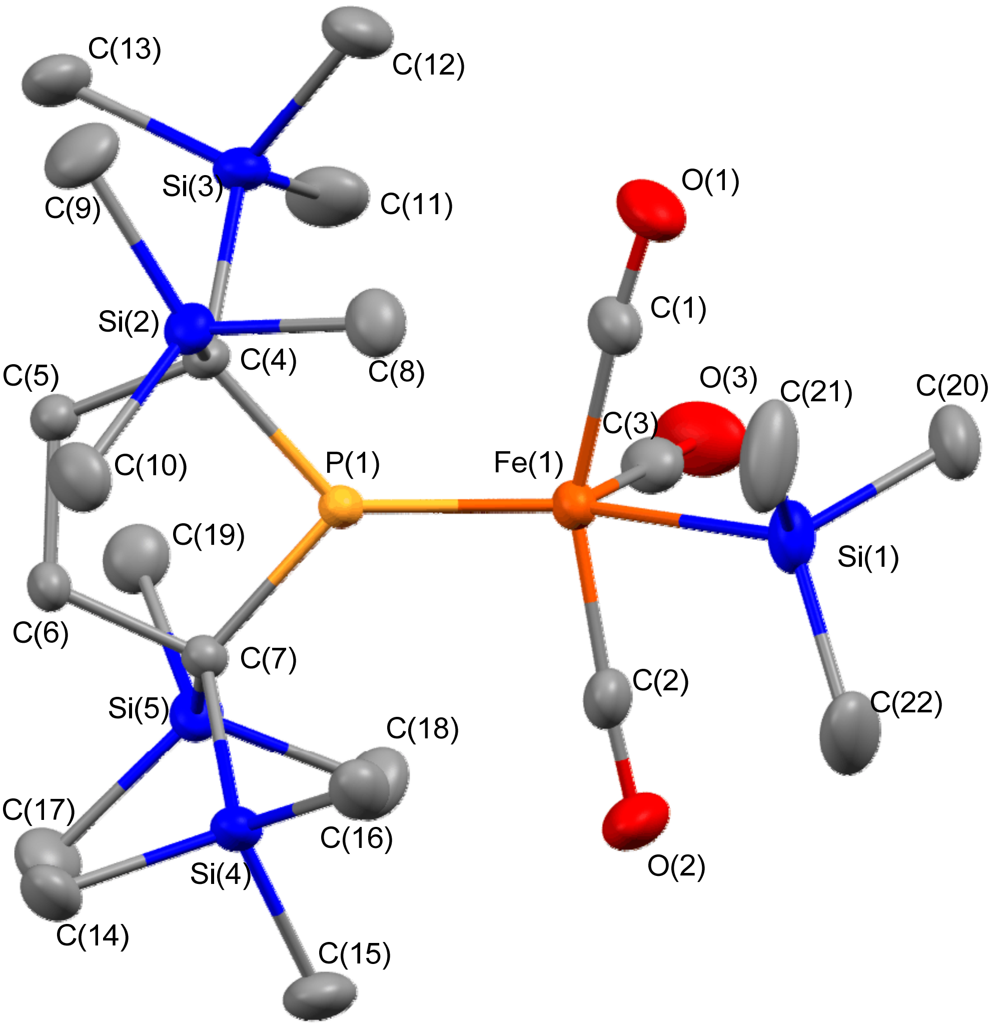

Figure S27. ORTEP drawing of **5** (50% probability of the thermal ellipsoids)

Table S9-1. Crystal data and structure refinement for **5**

Empirical Formula	$C_{22}H_{49}FeO_3PSi_5$
Formula Weight	588.88
Crystal Color, Habit	palegreen, platelet
Crystal Dimensions	0.050 X 0.020 X 0.015 mm
Crystal System	triclinic
Lattice Type	Primitive
Lattice Parameters	a = 11.1846(9) Å
	b = 11.2283(10) Å
	c = 14.972(2) Å
	α = 83.758(15) ^{o}
	β = 76.946(13) ^{o}
	γ = 60.902(8) ^{o}
	V = 1600.5(3) $Å^3$
Space Group	P-1 (#2)
Z value	2
D_{calc}	1.222 g/cm^3
F_{000}	632.00
μ(MoKα)	7.279 cm^{-1}
Diffractometer	Saturn724
Radiation	MoKα (λ = 0.71075 Å)
	multi-layer mirror monochromated
Voltage, Current	50kV, 40mA
Temperature	123 K
Detector Aperture	72.8 x 72.8 mm
Data Images	720 exposures
ω oscillation Range (χ=45.0, ϕ=0.0)	-105.0 - 75.0^o
Exposure Rate	48.0 sec./^{o}
Detector Swing Angle	-14.83^o
Detector Position	40.15 mm
Pixel Size	0.070 mm
$2\theta_{max}$	55.0^o
No. of Reflections Measured	Total: 15690
	Unique: 7170 (R_{int} = 0.0405)
Corrections	Lorentz-polarization
	Absorption
	(trans. factors: 0.888 - 0.989)
Structure Solution	Direct Methods (SIR2008)
Refinement	Full-matrix least-squares on F^2
Function Minimized	Σ w $(Fo^2 - Fc^2)^2$
Least Squares Weights	$\omega = 1/ [\sigma^2(Fo^2) + (0.0434 \cdot P)^2 + 1.0913 \cdot P]$
	where $P = (Max(Fo^2,0) + 2Fc^2)/3$
$2\theta_{max}$ cutoff	54.9^o
Anomalous Dispersion	All non-hydrogen atoms
No. Observations (All reflections)	7170
No. Variables	289
Reflection/Parameter Ratio	24.81
Residuals: R1 (I>2.00σ(I))	0.0444
Residuals: R (All reflections)	0.0561
Residuals: wR2 (All reflections)	0.1048
Goodness of Fit Indicator	1.072
Max Shift/Error in Final Cycle	0.001
Maximum peak in Final Diff. Map	0.47 $e^-/Å^3$
Minimum peak in Final Diff. Map	-0.42 $e^-/Å^3$

Table S9-2. Atomic coordinates and B_{iso}/B_{eq}

atom	x	y	z	B_{eq}
Fe1	0.32634(3)	0.06317(3)	0.82748(2)	1.669(7)
P1	0.18346(6)	0.21841(6)	0.75550(4)	1.401(10)
Si1	0.52825(8)	-0.16076(7)	0.79686(5)	2.437(13)
Si2	0.04508(7)	0.14433(7)	0.62541(4)	1.769(11)
Si3	-0.10598(7)	0.21646(7)	0.83390(5)	1.919(12)
Si4	0.34403(7)	0.34095(7)	0.60729(5)	1.895(12)
Si5	0.15623(7)	0.50191(7)	0.79220(5)	1.944(12)
O1	0.2205(2)	-0.1325(2)	0.85931(15)	3.38(4)
O2	0.5546(2)	0.1310(2)	0.78977(16)	3.62(4)
O3	0.2874(3)	0.0995(3)	1.02420(14)	4.26(5)
C1	0.2586(3)	-0.0527(3)	0.84385(17)	2.30(4)
C2	0.4639(3)	0.1054(3)	0.80123(18)	2.37(4)
C3	0.3038(3)	0.0839(3)	0.94691(19)	2.50(4)
C4	0.0168(2)	0.2467(2)	0.73049(15)	1.49(3)
C5	-0.0598(2)	0.4029(2)	0.70883(16)	1.69(4)
C6	0.0474(2)	0.4466(2)	0.65889(16)	1.72(4)
C7	0.1813(2)	0.3760(2)	0.70272(15)	1.52(3)
C8	0.1854(3)	-0.0355(3)	0.62773(18)	2.53(5)
C9	-0.1220(3)	0.1500(3)	0.6162(2)	3.04(5)
C10	0.0933(3)	0.2176(3)	0.51323(17)	2.68(5)
C11	-0.0844(3)	0.2569(3)	0.94472(19)	3.34(5)
C12	-0.0905(3)	0.0422(3)	0.8431(2)	3.08(5)
C13	-0.2908(3)	0.3388(3)	0.8241(2)	2.81(5)
C14	0.2840(3)	0.4562(3)	0.50836(18)	2.74(5)
C15	0.4777(3)	0.3763(3)	0.6367(2)	3.09(5)
C16	0.4421(3)	0.1608(3)	0.56517(18)	2.53(5)
C17	0.1471(3)	0.6595(3)	0.7299(2)	2.97(5)
C18	0.2928(3)	0.4353(3)	0.8634(2)	3.05(5)
C19	-0.0134(3)	0.5604(3)	0.87518(18)	2.67(5)
C20	0.5496(3)	-0.2597(3)	0.9066(2)	3.05(5)
C21	0.5145(4)	-0.2633(3)	0.7126(2)	4.47(8)
C22	0.7003(3)	-0.1652(3)	0.7461(2)	3.91(6)

$B_{eq} = 8/3\ \pi^2(U_{11}(aa^*)^2 + U_{22}(bb^*)^2 + U_{33}(cc^*)^2 + 2U_{12}(aa^*bb^*)\cos\gamma + 2U_{13}(aa^*cc^*)\cos\beta + 2U_{23}(bb^*cc^*)\cos\alpha)$

Table S9-3. Anisotropic displacement parameters

atom	U_{11}	U_{22}	U_{33}	U_{12}	U_{13}	U_{23}
Fe1	0.02140(17)	0.01956(17)	0.02341(18)	-0.00974(14)	-0.00766(13)	0.00287(13)
P1	0.0170(3)	0.0162(3)	0.0210(3)	-0.0086(2)	-0.0047(2)	0.0014(2)
Si1	0.0309(4)	0.0240(4)	0.0286(4)	-0.0041(3)	-0.0113(3)	0.0011(3)
Si2	0.0223(3)	0.0231(3)	0.0242(3)	-0.0112(3)	-0.0075(3)	-0.0014(3)
Si3	0.0218(3)	0.0267(3)	0.0269(3)	-0.0149(3)	-0.0020(3)	0.0011(3)
Si4	0.0199(3)	0.0230(3)	0.0282(4)	-0.0113(3)	-0.0021(3)	0.0035(3)
Si5	0.0250(3)	0.0189(3)	0.0318(4)	-0.0106(3)	-0.0079(3)	-0.0021(3)
O1	0.0549(13)	0.0372(11)	0.0523(13)	-0.0325(11)	-0.0206(10)	0.0127(9)
O2	0.0278(10)	0.0418(12)	0.0727(16)	-0.0198(9)	-0.0149(10)	0.0078(11)
O3	0.0758(17)	0.0801(17)	0.0274(11)	-0.0532(15)	-0.0109(11)	-0.0018(11)
C1	0.0337(13)	0.0279(13)	0.0292(13)	-0.0161(11)	-0.0123(11)	0.0057(10)
C2	0.0236(12)	0.0255(13)	0.0356(14)	-0.0072(10)	-0.0093(11)	0.0050(11)
C3	0.0338(14)	0.0359(14)	0.0326(14)	-0.0212(12)	-0.0099(11)	0.0014(11)
C4	0.0164(10)	0.0193(11)	0.0228(11)	-0.0100(9)	-0.0043(9)	0.0004(9)
C5	0.0191(11)	0.0211(11)	0.0249(12)	-0.0101(9)	-0.0052(9)	0.0009(9)
C6	0.0215(11)	0.0188(11)	0.0256(12)	-0.0094(9)	-0.0085(9)	0.0047(9)
C7	0.0179(10)	0.0175(10)	0.0218(11)	-0.0088(9)	-0.0029(9)	0.0012(9)
C8	0.0381(14)	0.0266(13)	0.0322(14)	-0.0133(12)	-0.0118(12)	-0.0038(11)
C9	0.0315(14)	0.0495(17)	0.0420(16)	-0.0217(13)	-0.0100(12)	-0.0113(13)
C10	0.0443(16)	0.0369(15)	0.0234(13)	-0.0198(13)	-0.0105(11)	0.0003(11)
C11	0.0443(17)	0.061(2)	0.0279(14)	-0.0335(16)	0.0013(12)	-0.0019(13)
C12	0.0366(15)	0.0325(14)	0.0511(18)	-0.0225(13)	-0.0028(13)	0.0064(13)
C13	0.0236(12)	0.0380(15)	0.0433(16)	-0.0165(12)	0.0021(11)	-0.0025(12)
C14	0.0333(14)	0.0336(14)	0.0334(14)	-0.0168(12)	-0.0015(11)	0.0090(11)
C15	0.0277(14)	0.0432(16)	0.0514(18)	-0.0234(13)	-0.0025(13)	0.0023(14)
C16	0.0265(13)	0.0297(13)	0.0312(14)	-0.0096(11)	0.0012(11)	0.0001(11)

C17	0.0430(16)	0.0226(13)	0.0515(18)	-0.0185(12)	-0.0101(13)	-0.0002(12)
C18	0.0378(15)	0.0363(15)	0.0462(17)	-0.0156(13)	-0.0177(13)	-0.0086(13)
C19	0.0358(14)	0.0320(14)	0.0325(14)	-0.0138(12)	-0.0061(11)	-0.0097(11)
C20	0.0429(16)	0.0294(14)	0.0425(16)	-0.0129(13)	-0.0208(13)	0.0092(12)
C21	0.055(2)	0.0368(17)	0.053(2)	0.0084(15)	-0.0282(17)	-0.0169(15)
C22	0.0308(15)	0.0418(17)	0.0472(18)	0.0011(13)	-0.0004(13)	0.0032(14)

The general temperature factor expression: $\exp(-2\pi^2(a^{*2}U_{11}h^2 + b^{*2}U_{22}k^2 + c^{*2}U_{33}l^2 + 2a^*b^*U_{12}hk + 2a^*c^*U_{13}hl + 2b^*c^*U_{23}kl))$

Table S9-4. Bond lengths (Å)

atom	atom	distance	atom	atom	distance
Fe1	P1	2.1009(7)	Fe1	Si1	2.4274(7)
Fe1	C1	1.769(4)	Fe1	C2	1.773(3)
Fe1	C3	1.773(3)	P1	C4	1.852(3)
P1	C7	1.849(3)	Si1	C20	1.874(3)
Si1	C21	1.875(4)	Si1	C22	1.881(4)
Si2	C4	1.927(3)	Si2	C8	1.862(2)
Si2	C9	1.874(4)	Si2	C10	1.874(3)
Si3	C4	1.943(2)	Si3	C11	1.862(4)
Si3	C12	1.870(4)	Si3	C13	1.874(3)
Si4	C7	1.939(2)	Si4	C14	1.872(3)
Si4	C15	1.871(4)	Si4	C16	1.870(3)
Si5	C7	1.931(3)	Si5	C17	1.876(3)
Si5	C18	1.865(3)	Si5	C19	1.864(3)
O1	C1	1.150(4)	O2	C2	1.155(4)
O3	C3	1.149(4)	C4	C5	1.566(3)
C5	C6	1.525(4)	C6	C7	1.574(3)

Table S9-5. Bond angles (^{o})

atom	atom	atom	angle	atom	atom	atom	angle
P1	Fe1	Si1	138.27(3)	P1	Fe1	C1	100.19(9)
P1	Fe1	C2	99.83(8)	P1	Fe1	C3	118.64(8)
Si1	Fe1	C1	74.51(7)	Si1	Fe1	C2	78.93(8)
Si1	Fe1	C3	103.05(8)	C1	Fe1	C2	153.44(10)
C1	Fe1	C3	93.04(15)	C2	Fe1	C3	92.51(15)
Fe1	P1	C4	131.74(8)	Fe1	P1	C7	130.30(9)
C4	P1	C7	97.88(11)	Fe1	Si1	C20	109.07(8)
Fe1	Si1	C21	113.63(10)	Fe1	Si1	C22	116.47(11)
C20	Si1	C21	107.54(16)	C20	Si1	C22	106.88(15)
C21	Si1	C22	102.65(17)	C4	Si2	C8	112.29(12)
C4	Si2	C9	110.55(12)	C4	Si2	C10	113.68(14)
C8	Si2	C9	110.38(15)	C8	Si2	C10	106.55(11)
C9	Si2	C10	102.95(15)	C4	Si3	C11	111.47(16)
C4	Si3	C12	116.15(12)	C4	Si3	C13	108.04(11)
C11	Si3	C12	108.10(16)	C11	Si3	C13	105.89(13)
C12	Si3	C13	106.62(16)	C7	Si4	C14	108.12(11)
C7	Si4	C15	116.51(13)	C7	Si4	C16	113.08(13)
C14	Si4	C15	104.89(16)	C14	Si4	C16	108.35(13)
C15	Si4	C16	105.36(13)	C7	Si5	C17	107.86(13)
C7	Si5	C18	115.37(11)	C7	Si5	C19	112.28(14)
C17	Si5	C18	109.85(17)	C17	Si5	C19	105.33(12)
C18	Si5	C19	105.68(13)	Fe1	C1	O1	175.0(3)
Fe1	C2	O2	175.7(3)	Fe1	C3	O3	178.8(2)
P1	C4	Si2	112.28(10)	P1	C4	Si3	114.62(12)
P1	C4	C5	103.6(2)	Si2	C4	Si3	110.33(15)
Si2	C4	C5	109.48(16)	Si3	C4	C5	106.08(13)
C4	C5	C6	109.33(17)	C5	C6	C7	110.38(19)
P1	C7	Si4	113.02(10)	P1	C7	Si5	112.58(12)
P1	C7	C6	104.64(19)	Si4	C7	Si5	110.51(15)
Si4	C7	C6	108.99(15)	Si5	C7	C6	106.69(13)

Table S9-6. Torsion Angles(^{o})

(Those having bond angles > 160 or < 20 degrees are excluded.)

atom1	atom2	atom3	atom4	angle	atom1	atom2	atom3	atom4	angle
P1	Fe1	Si1	C20	-156.52(4)	P1	Fe1	Si1	C21	-36.56(7)
P1	Fe1	Si1	C22	82.45(7)	Si1	Fe1	P1	C4	87.10(6)
Si1	Fe1	P1	C7	-96.92(6)	C1	Fe1	P1	C4	8.99(9)
C1	Fe1	P1	C7	-175.03(8)	C2	Fe1	P1	C4	171.45(9)
C2	Fe1	P1	C7	-12.57(10)	C3	Fe1	P1	C4	-90.19(14)
C3	Fe1	P1	C7	85.79(14)	C1	Fe1	Si1	C20	-68.52(10)
C1	Fe1	Si1	C21	51.44(10)	C1	Fe1	Si1	C22	170.45(10)
C2	Fe1	Si1	C20	111.07(10)	C2	Fe1	Si1	C21	-128.97(10)
C2	Fe1	Si1	C22	-9.96(10)	C3	Fe1	Si1	C20	21.04(13)
C3	Fe1	Si1	C21	141.00(13)	C3	Fe1	Si1	C22	-99.99(13)
Fe1	P1	C4	Si2	-82.86(13)	Fe1	P1	C4	Si3	44.06(16)
Fe1	P1	C4	C5	159.14(6)	Fe1	P1	C7	Si4	60.96(17)
Fe1	P1	C7	Si5	-65.12(12)	Fe1	P1	C7	C6	179.41(5)
C4	P1	C7	Si4	-122.06(14)	C4	P1	C7	Si5	111.85(12)
C4	P1	C7	C6	-3.62(12)	C7	P1	C4	Si2	100.23(13)
C7	P1	C4	Si3	-132.85(12)	C7	P1	C4	C5	-17.77(12)
C8	Si2	C4	P1	45.54(18)	C8	Si2	C4	Si3	-83.65(15)
C8	Si2	C4	C5	159.98(15)	C9	Si2	C4	P1	169.29(13)
C9	Si2	C4	Si3	40.11(15)	C9	Si2	C4	C5	-76.27(18)
C10	Si2	C4	P1	-75.51(15)	C10	Si2	C4	Si3	155.30(12)
C10	Si2	C4	C5	38.92(18)	C11	Si3	C4	P1	30.77(16)
C11	Si3	C4	Si2	158.69(12)	C11	Si3	C4	C5	-82.84(17)
C12	Si3	C4	P1	-93.63(18)	C12	Si3	C4	Si2	34.29(17)
C12	Si3	C4	C5	152.76(16)	C13	Si3	C4	P1	146.71(15)
C13	Si3	C4	Si2	-85.38(15)	C13	Si3	C4	C5	33.1(2)
C14	Si4	C7	P1	136.40(16)	C14	Si4	C7	Si5	-96.42(16)
C14	Si4	C7	C6	20.5(2)	C15	Si4	C7	P1	-105.86(17)
C15	Si4	C7	Si5	21.32(15)	C15	Si4	C7	C6	138.26(15)
C16	Si4	C7	P1	16.43(19)	C16	Si4	C7	Si5	143.61(13)
C16	Si4	C7	C6	-99.45(16)	C17	Si5	C7	P1	-177.18(13)
C17	Si5	C7	Si4	55.39(15)	C17	Si5	C7	C6	-62.95(17)
C18	Si5	C7	P1	59.61(18)	C18	Si5	C7	Si4	-67.81(16)
C18	Si5	C7	C6	173.84(14)	C19	Si5	C7	P1	-61.56(14)
C19	Si5	C7	Si4	171.01(11)	C19	Si5	C7	C6	52.67(16)
P1	C4	C5	C6	35.4(2)	Si2	C4	C5	C6	-84.5(2)
Si3	C4	C5	C6	156.47(15)	C4	C5	C6	C7	-41.0(3)
C5	C6	C7	P1	25.5(2)	C5	C6	C7	Si4	146.64(17)
C5	C6	C7	Si5	-94.0(2)					

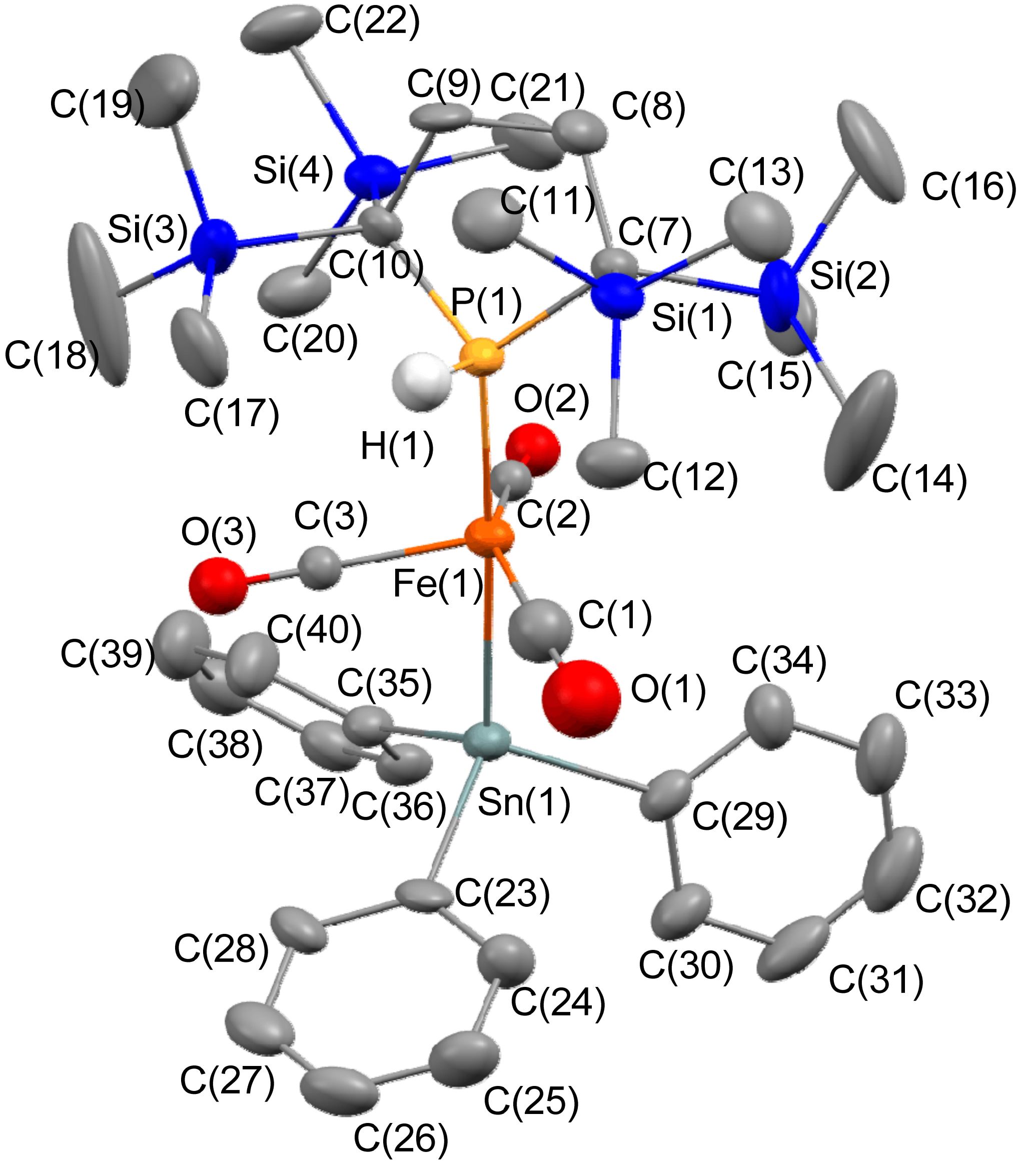

Figure S28. ORTEP drawing of **8** (50% probability of the thermal ellipsoids). (Three CO ligands on the iron were found to be disordered. The site occupancy factor for C1, C2, C3, O1, O2 and O3 was defined as 0.6, and the site occupancy factor for C4, C5, C6, O4, O5 and O6 was defined as 0.4. Only three CO ligands {C(1)-O(1), C(2)-O(2) and C(3)-O(3)} were shown in this figure for clarity.)

Table S10-1. Crystal data and structure refinement for **8**

Empirical Formula	$C_{37}H_{56}FeO_3PSi_4Sn$
Formula Weight	866.70
Crystal Color, Habit	colorless, block
Crystal Dimensions	0.120 X 0.120 X 0.100 mm
Crystal System	orthorhombic
Lattice Type	Primitive
Lattice Parameters	a = 11.387(3) Å
	b = 14.883(4) Å
	c = 24.983(6) Å
	V = 4234.0(17) $Å^3$
Space Group	$P2_12_12_1$ (#19)
Z value	4
D_{calc}	1.360 g/cm^3
F_{000}	1796.00
μ(MoKα)	11.149 cm^{-1}
Diffractometer	Saturn70
Radiation	MoKα (λ = 0.71075 Å)
	multi-layer mirror monochromated
Voltage, Current	50kV, 16mA
Temperature	-149.8^{o}C
Detector Aperture	70.0 x 70.0 mm
Data Images	720 exposures
ω oscillation Range (χ=45.0, ϕ=270.0)	-110.0 - 70.0^o
Exposure Rate	2.0 sec./^{o}
Detector Swing Angle	-19.55^o
Detector Position	45.06 mm
Pixel Size	0.137 mm
$2\theta_{max}$	55.0^o
No. of Reflections Measured	Total: 34784
	Unique: 9661 (R_{int} = 0.0733)
	Friedel pairs: 4309
Corrections	Lorentz-polarization
	Absorption
	(trans. factors: 0.715 - 0.894)
Structure Solution	Direct Methods (SHELXS97)
Refinement	Full-matrix least-squares on F^2
Function Minimized	Σ w $(Fo^2 - Fc^2)^2$
Least Squares Weights	w = 1/ [$\sigma^2(Fo^2) + (0.0618 \cdot P)^2$
	+ 0.0000 · P]
	where P = (Max(Fo^2,0) + 2Fc^2)/3
$2\theta_{max}$ cutoff	55.0^o
Anomalous Dispersion	All non-hydrogen atoms
No. Observations (All reflections)	9661
No. Variables	422
Reflection/Parameter Ratio	22.89
Residuals: R1 (I>2.00σ(I))	0.0592
Residuals: R (All reflections)	0.0741
Residuals: wR2 (All reflections)	0.1351
Goodness of Fit Indicator	1.089
Flack Parameter (Friedel pairs = 4309)	0.02(2)
Max Shift/Error in Final Cycle	0.002
Maximum peak in Final Diff. Map	1.28 $e^-/Å^3$
Minimum peak in Final Diff. Map	-0.88 $e^-/Å^3$

Table S10-2. Atomic coordinates and B_{iso}/B_{eq} and occupancy

atom	x	y	z	B_{eq}	occ
Sn1	0.14482(3)	0.00222(3)	0.858978(13)	1.966(9)	1
Fe1	0.19779(7)	0.02449(5)	0.95825(3)	1.937(14)	1
P1	0.25188(12)	0.03918(10)	1.04406(5)	1.89(2)	1
Si1	0.20130(15)	0.20091(11)	1.11643(6)	2.28(3)	1
Si2	-0.00983(16)	0.06955(14)	1.09559(9)	3.68(4)	1
Si3	0.51013(15)	-0.02436(13)	1.06563(8)	3.41(4)	1
Si4	0.31121(16)	-0.16966(11)	1.07326(6)	2.39(3)	1
O1	0.1490(9)	0.2086(7)	0.9305(4)	5.9(2)	0.600000
O2	0.0997(7)	-0.1575(6)	0.9591(3)	2.85(16)	0.600000
O3	0.4473(6)	0.0180(5)	0.9193(3)	3.31(14)	0.600000
O4	0.0230(9)	0.1729(7)	0.9515(4)	2.86(19)	0.400000
O5	0.0597(11)	-0.1378(8)	0.9629(5)	3.0(3)	0.400000
O6	0.4006(12)	0.1154(9)	0.9176(5)	4.6(3)	0.400000
C1	0.1733(11)	0.1342(9)	0.9429(5)	4.1(2)	0.600000
C2	0.1434(9)	-0.0866(6)	0.9601(4)	1.75(15)	0.600000
C3	0.3510(7)	0.0137(6)	0.9321(3)	1.94(14)	0.600000
C4	0.0876(12)	0.1138(9)	0.9572(5)	1.6(2)	0.400000
C5	0.1009(16)	-0.0657(12)	0.9631(7)	2.9(3)	0.400000
C6	0.3100(15)	0.0840(11)	0.9339(6)	3.1(3)	0.400000
C7	0.1587(5)	0.0771(4)	1.1011(2)	1.95(9)	1
C8	0.1981(5)	0.0155(4)	1.1475(2)	2.76(11)	1
C9	0.3231(5)	-0.0139(4)	1.14020(19)	2.61(11)	1
C10	0.3458(5)	-0.0431(3)	1.0800(2)	1.78(9)	1
C11	0.3495(7)	0.2120(5)	1.1468(3)	3.96(14)	1
C12	0.1989(7)	0.2729(4)	1.0559(3)	3.37(13)	1
C13	0.1014(7)	0.2519(5)	1.1678(3)	4.15(15)	1
C14	-0.0798(7)	0.1646(7)	1.0598(6)	9.5(4)	1
C15	-0.0681(6)	-0.0336(6)	1.0627(3)	5.1(2)	1
C16	-0.0700(8)	0.0615(6)	1.1653(4)	6.6(3)	1
C17	0.5497(6)	0.0906(5)	1.0448(3)	4.45(16)	1
C18	0.5782(9)	-0.0980(7)	1.0163(6)	11.7(5)	1
C19	0.5909(7)	-0.0339(8)	1.1326(4)	7.5(3)	1
C20	0.3407(7)	-0.2193(4)	1.0053(3)	3.67(14)	1
C21	0.1556(6)	-0.1994(5)	1.0916(3)	3.77(14)	1
C22	0.4072(7)	-0.2315(5)	1.1227(3)	4.04(16)	1
C23	0.2140(6)	0.1053(4)	0.8077(2)	2.43(11)	1
C24	0.1540(7)	0.1860(4)	0.8006(3)	3.12(12)	1
C25	0.2014(7)	0.2553(5)	0.7690(3)	3.82(15)	1
C26	0.3097(7)	0.2423(5)	0.7446(3)	3.80(14)	1
C27	0.3683(7)	0.1638(5)	0.7509(2)	3.77(14)	1
C28	0.3230(6)	0.0935(5)	0.7824(2)	2.83(12)	1
C29	-0.0418(5)	0.0014(4)	0.8437(2)	2.35(9)	1
C30	-0.0820(5)	0.0021(5)	0.7910(2)	3.16(11)	1
C31	-0.2009(6)	-0.0018(6)	0.7794(3)	4.09(14)	1
C32	-0.2820(6)	-0.0081(5)	0.8199(4)	4.67(16)	1
C33	-0.2448(5)	-0.0083(6)	0.8722(3)	4.24(15)	1
C34	-0.1266(5)	-0.0043(5)	0.8843(3)	3.10(11)	1
C35	0.2109(5)	-0.1243(4)	0.8306(2)	2.29(10)	1
C36	0.1430(6)	-0.1828(4)	0.8014(2)	2.34(10)	1
C37	0.1825(6)	-0.2670(4)	0.7870(2)	3.02(12)	1
C38	0.2905(7)	-0.2966(5)	0.8013(3)	3.60(13)	1
C39	0.3618(7)	-0.2392(5)	0.8315(3)	4.38(16)	1
C40	0.3249(6)	-0.1546(5)	0.8456(3)	3.71(14)	1

$B_{eq} = 8/3\ \pi^2(U_{11}(aa^*)^2 + U_{22}(bb^*)^2 + U_{33}(cc^*)^2 + 2U_{12}(aa^*bb^*)\cos\gamma + 2U_{13}(aa^*cc^*)\cos\beta + 2U_{23}(bb^*cc^*)\cos\alpha)$

Table S10-3. Anisotropic displacement parameters

atom	U_{11}	U_{22}	U_{33}	U_{12}	U_{13}	U_{23}
Sn1	0.02882(19)	0.02694(19)	0.01892(16)	0.00196(19)	-0.00182(14)	-0.00054(17)
Fe1	0.0290(4)	0.0245(4)	0.0200(4)	-0.0029(3)	-0.0022(3)	-0.0003(3)
P1	0.0255(7)	0.0284(7)	0.0178(6)	0.0040(6)	-0.0007(6)	-0.0007(6)
Si1	0.0371(9)	0.0264(8)	0.0233(8)	0.0003(8)	0.0010(7)	-0.0027(7)
Si2	0.0234(9)	0.0451(11)	0.0712(14)	0.0024(8)	0.0123(9)	0.0039(11)

Si3	0.0254(8)	0.0463(12)	0.0576(12)	0.0012(8)	0.0062(8)	0.0234(9)
Si4	0.0407(9)	0.0246(8)	0.0254(8)	0.0004(8)	-0.0008(7)	0.0016(7)
C7	0.030(3)	0.023(3)	0.021(3)	0.002(2)	0.002(2)	0.002(2)
C8	0.044(3)	0.037(4)	0.023(3)	0.008(3)	0.003(2)	-0.001(3)
C9	0.049(3)	0.036(3)	0.014(2)	0.003(3)	-0.007(2)	0.006(3)
C10	0.023(3)	0.023(3)	0.022(2)	-0.003(2)	0.003(2)	0.004(2)
C11	0.062(5)	0.043(4)	0.046(4)	-0.001(4)	-0.006(4)	-0.011(3)
C12	0.063(4)	0.029(3)	0.036(4)	-0.004(3)	-0.008(3)	0.003(3)
C13	0.067(5)	0.038(4)	0.052(4)	0.001(4)	0.011(4)	-0.016(3)
C14	0.042(5)	0.070(7)	0.250(16)	0.006(5)	-0.023(7)	0.051(9)
C15	0.024(3)	0.100(7)	0.068(5)	-0.020(4)	0.002(3)	-0.000(5)
C16	0.076(6)	0.074(6)	0.099(7)	-0.021(5)	0.062(6)	-0.035(5)
C17	0.045(4)	0.053(4)	0.071(5)	-0.014(4)	0.027(4)	-0.005(4)
C18	0.078(7)	0.069(7)	0.295(19)	-0.030(6)	0.114(10)	-0.069(9)
C19	0.039(4)	0.149(11)	0.097(7)	-0.014(5)	-0.011(5)	0.047(7)
C20	0.071(5)	0.034(3)	0.034(3)	0.015(4)	-0.010(4)	-0.004(3)
C21	0.059(5)	0.043(4)	0.041(4)	-0.023(4)	0.006(4)	0.006(3)
C22	0.082(6)	0.030(4)	0.042(4)	0.009(4)	-0.020(4)	0.008(3)
C23	0.040(3)	0.040(3)	0.012(3)	-0.003(3)	-0.003(3)	0.003(2)
C24	0.044(4)	0.035(3)	0.040(4)	0.005(3)	0.004(4)	0.005(3)
C25	0.057(5)	0.038(4)	0.051(4)	-0.005(4)	-0.009(4)	0.012(3)
C26	0.072(5)	0.041(4)	0.032(4)	-0.016(4)	-0.002(4)	0.007(3)
C27	0.057(4)	0.062(5)	0.025(3)	-0.011(4)	0.001(3)	-0.005(3)
C28	0.040(4)	0.047(4)	0.021(3)	-0.006(3)	0.006(3)	-0.001(3)
C29	0.030(3)	0.024(3)	0.036(3)	0.001(3)	-0.012(2)	-0.003(3)
C30	0.041(3)	0.037(3)	0.043(3)	0.006(4)	-0.015(3)	-0.002(4)
C31	0.055(4)	0.038(3)	0.063(4)	0.006(4)	-0.036(4)	-0.004(4)
C32	0.036(4)	0.041(4)	0.101(6)	0.002(4)	-0.021(4)	-0.009(5)
C33	0.033(3)	0.044(4)	0.084(5)	0.008(3)	0.001(3)	-0.008(5)
C34	0.034(3)	0.034(3)	0.050(3)	0.002(3)	0.006(3)	-0.006(3)
C35	0.035(3)	0.031(3)	0.021(3)	0.005(3)	-0.001(3)	-0.000(2)
C36	0.040(3)	0.022(3)	0.026(3)	0.003(3)	-0.002(3)	0.003(2)
C37	0.052(4)	0.034(3)	0.029(3)	-0.002(3)	0.008(3)	-0.001(3)
C38	0.061(5)	0.036(4)	0.039(4)	0.011(4)	0.013(4)	0.001(3)
C39	0.052(4)	0.059(5)	0.055(5)	0.022(4)	0.004(4)	-0.007(4)
C40	0.043(4)	0.054(4)	0.044(4)	0.018(3)	-0.003(3)	-0.016(3)

The general temperature factor expression: $\exp(-2\pi^2(a^{*2}U_{11}h^2 + b^{*2}U_{22}k^2 + c^{*2}U_{33}l^2 + 2a^*b^*U_{12}hk + 2a^*c^*U_{13}hl + 2b^*c^*U_{23}kl))$

Table S10-4. Bond lengths (Å)

atom	atom	distance	atom	atom	distance
Sn1	Fe1	2.5739(10)	Sn1	C23	2.147(6)
Sn1	C29	2.160(5)	Sn1	C35	2.148(6)
Fe1	P1	2.2412(16)	Fe1	C1	1.700(13)
Fe1	C2	1.766(10)	Fe1	C3	1.870(8)
Fe1	C4	1.828(13)	Fe1	C5	1.741(18)
Fe1	C6	1.670(17)	P1	C7	1.864(5)
P1	C10	1.856(5)	Si1	C7	1.944(5)
Si1	C11	1.858(8)	Si1	C12	1.853(6)
Si1	C13	1.876(8)	Si2	C7	1.927(6)
Si2	C14	1.854(11)	Si2	C15	1.864(8)
Si2	C16	1.875(9)	Si3	C10	1.925(5)
Si3	C17	1.844(8)	Si3	C18	1.822(12)
Si3	C19	1.915(9)	Si4	C10	1.932(5)
Si4	C20	1.881(6)	Si4	C21	1.883(7)
Si4	C22	1.888(7)	O1	O4	1.618(14)
O1	C1	1.184(17)	O1	C4	1.711(16)
O2	O5	0.550(15)	O2	C2	1.167(13)
O2	C5	1.37(2)	O3	O6	1.545(15)
O3	C3	1.143(11)	O3	C6	1.882(18)
O4	C1	1.819(16)	O4	C4	1.155(16)
O5	C2	1.222(16)	O5	C5	1.17(2)
O6	C3	1.655(16)	O6	C6	1.20(2)
C1	C4	1.082(18)	C1	C6	1.74(2)
C2	C5	0.58(2)	C3	C6	1.147(19)

C7	C8	1.546(8)	C8	C9	1.500(8)
C9	C10	1.588(7)	C23	C24	1.393(9)
C23	C28	1.404(9)	C24	C25	1.406(10)
C25	C26	1.390(11)	C26	C27	1.354(11)
C27	C28	1.406(10)	C29	C30	1.393(8)
C29	C34	1.403(8)	C30	C31	1.385(9)
C31	C32	1.373(11)	C32	C33	1.374(12)
C33	C34	1.381(8)	C35	C36	1.375(8)
C35	C40	1.425(9)	C36	C37	1.379(8)
C37	C38	1.354(10)	C38	C39	1.398(10)
C39	C40	1.374(11)	P1	H1	1.52(6)

Table S10-5. Bond angles (^{o})

atom	atom	atom	angle	atom	atom	atom	angle
Fe1	Sn1	C23	113.37(15)	Fe1	Sn1	C29	113.72(14)
Fe1	Sn1	C35	110.41(15)	C23	Sn1	C29	105.0(2)
C23	Sn1	C35	107.5(2)	C29	Sn1	C35	106.3(2)
Sn1	Fe1	P1	177.04(5)	Sn1	Fe1	C1	82.4(4)
Sn1	Fe1	C2	79.8(3)	Sn1	Fe1	C3	82.6(2)
Sn1	Fe1	C4	85.4(4)	Sn1	Fe1	C5	79.6(6)
Sn1	Fe1	C6	84.0(6)	P1	Fe1	C1	99.6(4)
P1	Fe1	C2	99.4(3)	P1	Fe1	C3	95.0(3)
P1	Fe1	C4	97.5(4)	P1	Fe1	C5	100.5(6)
P1	Fe1	C6	95.0(6)	C1	Fe1	C2	147.9(5)
C1	Fe1	C3	99.0(5)	C1	Fe1	C4	35.5(6)
C1	Fe1	C5	130.7(7)	C1	Fe1	C6	62.2(7)
C2	Fe1	C3	104.8(4)	C2	Fe1	C4	116.1(5)
C2	Fe1	C5	19.0(7)	C2	Fe1	C6	140.8(7)
C3	Fe1	C4	134.2(5)	C3	Fe1	C5	123.3(7)
C3	Fe1	C6	37.3(6)	C4	Fe1	C5	97.3(7)
C4	Fe1	C6	97.7(7)	C5	Fe1	C6	156.8(8)
Fe1	P1	C7	127.18(18)	Fe1	P1	C10	123.78(17)
C7	P1	C10	99.1(2)	C7	Si1	C11	113.0(3)
C7	Si1	C12	112.5(3)	C7	Si1	C13	111.5(3)
C11	Si1	C12	107.2(3)	C11	Si1	C13	103.6(3)
C12	Si1	C13	108.4(3)	C7	Si2	C14	114.7(3)
C7	Si2	C15	115.7(3)	C7	Si2	C16	107.5(3)
C14	Si2	C15	105.3(4)	C14	Si2	C16	109.9(5)
C15	Si2	C16	103.1(4)	C10	Si3	C17	115.1(3)
C10	Si3	C18	116.9(4)	C10	Si3	C19	107.1(3)
C17	Si3	C18	105.2(4)	C17	Si3	C19	101.5(4)
C18	Si3	C19	110.0(5)	C10	Si4	C20	115.1(3)
C10	Si4	C21	113.6(3)	C10	Si4	C22	107.5(3)
C20	Si4	C21	107.2(3)	C20	Si4	C22	107.2(3)
C21	Si4	C22	105.7(3)	O4	O1	C1	79.3(9)
O4	O1	C4	40.5(6)	C1	O1	C4	38.8(8)
O5	O2	C2	82.4(17)	O5	O2	C5	57.6(17)
C2	O2	C5	24.8(9)	O6	O3	C3	74.3(8)
O6	O3	C6	39.6(7)	C3	O3	C6	34.8(7)
O1	O4	C1	39.8(6)	O1	O4	C4	74.1(9)
C1	O4	C4	34.3(8)	O2	O5	C2	71.1(16)
O2	O5	C5	99(2)	C2	O5	C5	27.9(11)
O3	O6	C3	41.7(5)	O3	O6	C6	85.5(11)
C3	O6	C6	43.9(9)	Fe1	C1	O1	175.3(11)
Fe1	C1	O4	115.6(8)	Fe1	C1	C4	78.7(10)
Fe1	C1	C6	58.1(7)	O1	C1	O4	60.9(8)
O1	C1	C4	97.9(13)	O1	C1	C6	125.2(12)
O4	C1	C4	37.0(9)	O4	C1	C6	173.0(10)
C4	C1	C6	136.8(14)	Fe1	C2	O2	174.6(9)
Fe1	C2	O5	149.2(10)	Fe1	C2	C5	78(2)
O2	C2	O5	26.5(8)	O2	C2	C5	98(2)
O5	C2	C5	71(2)	Fe1	C3	O3	170.8(8)
Fe1	C3	O6	108.4(6)	Fe1	C3	C6	61.8(9)
O3	C3	O6	64.0(7)	O3	C3	C6	110.6(11)
O6	C3	C6	46.6(10)	Fe1	C4	O1	109.0(8)
Fe1	C4	O4	172.9(12)	Fe1	C4	C1	65.8(9)
O1	C4	O4	65.4(9)	O1	C4	C1	43.3(9)
O4	C4	C1	108.7(14)	Fe1	C5	O2	140.4(13)
Fe1	C5	O5	163.7(16)	Fe1	C5	C2	83(2)
O2	C5	O5	23.4(8)	O2	C5	C2	57.6(18)

O5	C5	C2	81(2)	Fe1	C6	O3	115.4(9)
Fe1	C6	O6	170.1(15)	Fe1	C6	C1	59.8(7)
Fe1	C6	C3	80.9(10)	O3	C6	O6	54.9(9)
O3	C6	C1	172.6(11)	O3	C6	C3	34.7(7)
O6	C6	C1	130.1(14)	O6	C6	C3	89.5(14)
C1	C6	C3	139.5(14)	P1	C7	Si1	107.2(3)
P1	C7	Si2	119.6(3)	P1	C7	C8	103.2(4)
Si1	C7	Si2	108.5(3)	Si1	C7	C8	110.0(4)
Si2	C7	C8	108.0(4)	C7	C8	C9	110.9(4)
C8	C9	C10	110.5(4)	P1	C10	Si3	112.0(3)
P1	C10	Si4	118.9(3)	P1	C10	C9	100.6(3)
Si3	C10	Si4	108.8(3)	Si3	C10	C9	107.1(3)
Si4	C10	C9	108.4(3)	Sn1	C23	C24	120.8(5)
Sn1	C23	C28	120.3(4)	C24	C23	C28	118.8(6)
C23	C24	C25	121.1(7)	C24	C25	C26	119.0(7)
C25	C26	C27	120.4(7)	C26	C27	C28	121.7(7)
C23	C28	C27	118.9(6)	Sn1	C29	C30	119.4(4)
Sn1	C29	C34	123.3(4)	C30	C29	C34	117.2(5)
C29	C30	C31	121.3(6)	C30	C31	C32	120.3(7)
C31	C32	C33	119.6(6)	C32	C33	C34	120.5(7)
C29	C34	C33	121.0(6)	Sn1	C35	C36	122.2(5)
Sn1	C35	C40	120.7(4)	C36	C35	C40	116.8(6)
C35	C36	C37	122.1(6)	C36	C37	C38	121.5(6)
C37	C38	C39	118.1(6)	C38	C39	C40	121.4(7)
C35	C40	C39	120.1(6)	Fe1	P1	H1	107(2)
C7	P1	H1	96(2)	C10	P1	H1	97(2)

Table S10-6. Torsion Angles(°)

(Those having bond angles > 160 or < 20 degrees are excluded.)

atom1	atom2	atom3	atom4	angle	atom1	atom2	atom3	atom4	angle
Fe1	Sn1	C23	C24	-84.4(4)	Fe1	Sn1	C23	C28	93.2(4)
C23	Sn1	Fe1	C1	38.93(19)	C23	Sn1	Fe1	C2	-167.86(18)
C23	Sn1	Fe1	C3	-61.23(19)	C23	Sn1	Fe1	C4	74.52(19)
C23	Sn1	Fe1	C5	172.82(18)	C23	Sn1	Fe1	C6	-23.73(19)
Fe1	Sn1	C29	C30	171.4(3)	Fe1	Sn1	C29	C34	-11.9(5)
C29	Sn1	Fe1	C1	-80.95(19)	C29	Sn1	Fe1	C2	72.26(19)
C29	Sn1	Fe1	C3	178.89(19)	C29	Sn1	Fe1	C4	-45.36(19)
C29	Sn1	Fe1	C5	52.94(19)	C29	Sn1	Fe1	C6	-143.61(19)
Fe1	Sn1	C35	C36	132.8(3)	Fe1	Sn1	C35	C40	-41.4(4)
C35	Sn1	Fe1	C1	159.61(17)	C35	Sn1	Fe1	C2	-47.18(17)
C35	Sn1	Fe1	C3	59.45(17)	C35	Sn1	Fe1	C4	-164.80(17)
C35	Sn1	Fe1	C5	-66.50(17)	C35	Sn1	Fe1	C6	96.95(17)
C23	Sn1	C29	C30	46.9(5)	C23	Sn1	C29	C34	-136.4(4)
C29	Sn1	C23	C24	40.3(4)	C29	Sn1	C23	C28	-142.1(4)
C23	Sn1	C35	C36	-103.1(4)	C23	Sn1	C35	C40	82.7(4)
C35	Sn1	C23	C24	153.3(4)	C35	Sn1	C23	C28	-29.2(4)
C29	Sn1	C35	C36	9.0(4)	C29	Sn1	C35	C40	-165.2(3)
C35	Sn1	C29	C30	-66.9(4)	C35	Sn1	C29	C34	109.8(4)
Sn1	Fe1	C1	O4	89.5(7)	Sn1	Fe1	C1	C4	92.7(6)
Sn1	Fe1	C1	C6	-87.2(2)	Sn1	Fe1	C2	O5	-83.6(14)
Sn1	Fe1	C2	C5	-87.7(9)	Sn1	Fe1	C3	O6	91.7(4)
Sn1	Fe1	C3	C6	89.9(3)	Sn1	Fe1	C4	O1	-81.0(6)
Sn1	Fe1	C4	C1	-83.4(6)	Sn1	Fe1	C5	O2	84.4(19)
Sn1	Fe1	C5	C2	88.8(17)	Sn1	Fe1	C6	O3	-89.1(8)
Sn1	Fe1	C6	C1	84.5(3)	Sn1	Fe1	C6	C3	-85.5(7)
P1	Fe1	C1	O4	-92.7(7)	P1	Fe1	C1	C4	-89.5(6)
P1	Fe1	C1	C6	90.6(2)	C1	Fe1	P1	C7	66.6(4)
C1	Fe1	P1	C10	-155.2(4)	P1	Fe1	C2	O5	99.3(14)
P1	Fe1	C2	C5	95.1(9)	C2	Fe1	P1	C7	-87.5(4)
C2	Fe1	P1	C10	50.8(4)	P1	Fe1	C3	O6	-89.9(4)
P1	Fe1	C3	C6	-91.7(3)	C3	Fe1	P1	C7	166.6(3)
C3	Fe1	P1	C10	-55.2(3)	P1	Fe1	C4	O1	98.5(6)
P1	Fe1	C4	C1	96.0(6)	C4	Fe1	P1	C7	30.7(4)
C4	Fe1	P1	C10	169.0(4)	P1	Fe1	C5	O2	-92.6(19)
P1	Fe1	C5	C2	-88.2(17)	C5	Fe1	P1	C7	-68.2(6)
C5	Fe1	P1	C10	70.1(6)	P1	Fe1	C6	O3	88.1(8)
P1	Fe1	C6	C1	-98.3(3)	P1	Fe1	C6	C3	91.6(7)
C6	Fe1	P1	C7	129.2(6)	C6	Fe1	P1	C10	-92.6(6)

C1	Fe1	C2	O5	-26(2)
C2	Fe1	C1	O4	32.8(14)
C2	Fe1	C1	C6	-143.9(8)
C1	Fe1	C3	C6	8.9(6)
C3	Fe1	C1	C4	173.9(6)
C1	Fe1	C4	O1	2.5(7)
C4	Fe1	C1	O4	-3.2(7)
C4	Fe1	C1	C6	-179.9(11)
C1	Fe1	C5	C2	159.2(14)
C5	Fe1	C1	C4	23.5(12)
C1	Fe1	C6	O3	-173.7(12)
C1	Fe1	C6	C3	-170.1(11)
C6	Fe1	C1	C4	179.9(10)
C2	Fe1	C3	O6	169.0(4)
C3	Fe1	C2	O5	-163.0(13)
C2	Fe1	C4	O1	-157.2(6)
C4	Fe1	C2	O5	-4.0(16)
C2	Fe1	C6	O3	-23.3(15)
C2	Fe1	C6	C3	-19.8(15)
C6	Fe1	C2	C5	-154.9(11)
C3	Fe1	C4	C1	-8.5(10)
C4	Fe1	C3	C6	13.8(8)
C3	Fe1	C5	C2	15(2)
C5	Fe1	C3	C6	162.2(7)
C3	Fe1	C6	C1	170.1(11)
C6	Fe1	C3	O6	1.8(9)
C4	Fe1	C5	O2	168.3(18)
C5	Fe1	C4	O1	-159.8(8)
C4	Fe1	C6	O3	-173.6(8)
C4	Fe1	C6	C3	-170.0(8)
C6	Fe1	C4	C1	-0.1(8)
C5	Fe1	C6	C1	129.8(19)
C6	Fe1	C5	O2	38(3)
Fe1	P1	C7	Si1	-103.0(2)
Fe1	P1	C7	C8	140.87(18)
Fe1	P1	C10	Si4	-46.3(4)
C7	P1	C10	Si3	-130.3(3)
C7	P1	C10	C9	-16.8(3)
C10	P1	C7	Si2	-124.9(3)
C11	Si1	C7	P1	-70.5(4)
C11	Si1	C7	C8	41.1(4)
C12	Si1	C7	Si2	-79.4(4)
C13	Si1	C7	P1	173.2(3)
C13	Si1	C7	C8	-75.2(4)
C14	Si2	C7	Si1	40.6(5)
C15	Si2	C7	P1	40.2(5)
C15	Si2	C7	C8	-77.3(4)
C16	Si2	C7	Si1	-81.9(4)
C17	Si3	C10	P1	24.1(4)
C17	Si3	C10	C9	-85.3(4)
C18	Si3	C10	Si4	33.4(5)
C19	Si3	C10	P1	136.0(4)
C19	Si3	C10	C9	26.6(5)
C20	Si4	C10	Si3	-60.5(4)
C21	Si4	C10	P1	-54.7(4)
C21	Si4	C10	C9	59.2(4)
C22	Si4	C10	Si3	58.8(3)
O4	O1	C1	O4	-0.0(4)
O4	O1	C1	C6	176.1(11)
C1	O1	O4	C4	1.0(8)
O4	O1	C4	O4	0.0(6)
C4	O1	O4	C1	-1.0(8)
C1	O1	C4	Fe1	-3.3(11)
C1	O1	C4	C1	0.0(10)
C4	O1	C1	C4	0.0(7)
O5	O2	C2	O5	-0.0(13)
C2	O2	O5	C2	-0.0(5)
O5	O2	C5	Fe1	-178(3)
O5	O2	C5	C2	177(3)
C5	O2	O5	C5	0.0(9)
C2	O2	C5	O5	-177(3)
C1	Fe1	C2	C5	-30.5(16)
C2	Fe1	C1	C4	36.0(14)
C1	Fe1	C3	O6	10.6(6)
C3	Fe1	C1	O4	170.6(6)
C3	Fe1	C1	C6	-6.1(4)
C1	Fe1	C4	C1	0.0(7)
C4	Fe1	C1	C4	0.0(7)
C1	Fe1	C5	O2	154.8(15)
C5	Fe1	C1	O4	20.2(13)
C5	Fe1	C1	C6	-156.5(8)
C1	Fe1	C6	C1	0.0(5)
C6	Fe1	C1	O4	176.7(11)
C6	Fe1	C1	C6	0.0(6)
C2	Fe1	C3	C6	167.2(4)
C3	Fe1	C2	C5	-167.1(9)
C2	Fe1	C4	C1	-159.6(5)
C4	Fe1	C2	C5	-8.1(11)
C2	Fe1	C6	C1	150.3(7)
C6	Fe1	C2	O5	-150.8(14)
C3	Fe1	C4	O1	-6.0(11)
C4	Fe1	C3	O6	15.6(8)
C3	Fe1	C5	O2	11(2)
C5	Fe1	C3	O6	164.0(7)
C3	Fe1	C6	O3	-3.6(4)
C3	Fe1	C6	C3	0.0(4)
C6	Fe1	C3	C6	0.0(9)
C4	Fe1	C5	C2	172.7(17)
C5	Fe1	C4	C1	-162.3(8)
C4	Fe1	C6	C1	0.1(6)
C6	Fe1	C4	O1	2.4(9)
C5	Fe1	C6	O3	-44(2)
C5	Fe1	C6	C3	-40(2)
C6	Fe1	C5	C2	43(3)
Fe1	P1	C7	Si2	21.0(4)
Fe1	P1	C10	Si3	82.2(3)
Fe1	P1	C10	C9	-164.30(14)
C7	P1	C10	Si4	101.3(3)
C10	P1	C7	Si1	111.1(3)
C10	P1	C7	C8	-5.1(3)
C11	Si1	C7	Si2	159.0(3)
C12	Si1	C7	P1	51.1(4)
C12	Si1	C7	C8	162.7(3)
C13	Si1	C7	Si2	42.7(4)
C14	Si2	C7	P1	-82.7(5)
C14	Si2	C7	C8	159.8(5)
C15	Si2	C7	Si1	163.5(3)
C16	Si2	C7	P1	154.7(4)
C16	Si2	C7	C8	37.3(4)
C17	Si3	C10	Si4	157.7(3)
C18	Si3	C10	P1	-100.1(5)
C18	Si3	C10	C9	150.4(5)
C19	Si3	C10	Si4	-90.4(4)
C20	Si4	C10	P1	69.4(4)
C20	Si4	C10	C9	-176.7(3)
C21	Si4	C10	Si3	175.4(3)
C22	Si4	C10	P1	-171.3(3)
C22	Si4	C10	C9	-57.4(4)
O4	O1	C1	C4	-1.0(8)
C1	O1	O4	C1	0.0(6)
O4	O1	C4	Fe1	175.2(12)
O4	O1	C4	C1	178.5(13)
C4	O1	O4	C4	0.0(7)
C1	O1	C4	O4	-178.5(15)
C4	O1	C1	O4	1.0(10)
C4	O1	C1	C6	177.1(19)
O5	O2	C2	C5	-2.3(19)
C2	O2	O5	C5	1.1(9)
O5	O2	C5	O5	0.0(16)
C5	O2	O5	C2	-1.1(12)
C2	O2	C5	Fe1	5.2(13)
C2	O2	C5	C2	0.0(11)

C5	O2	C2	O5	2(2)
O6	O3	C3	O6	0.0(5)
C3	O3	O6	C3	0.0(4)
O6	O3	C6	Fe1	-177.9(16)
O6	O3	C6	C3	175.9(16)
C6	O3	O6	C6	0.0(8)
C3	O3	C6	O6	-175.9(15)
C6	O3	C3	O6	2.7(10)
O1	O4	C1	Fe1	-176.4(13)
O1	O4	C1	C4	178.4(14)
O1	O4	C4	C1	-1.1(9)
C1	O4	C4	C1	-0.0(7)
C4	O4	C1	O1	-178.4(17)
O2	O5	C2	Fe1	173.3(18)
O2	O5	C2	C5	177.6(19)
O2	O5	C5	C2	-2(2)
C2	O5	C5	C2	-0.0(10)
C5	O5	C2	O2	-178(3)
O3	O6	C3	Fe1	174.3(8)
O3	O6	C3	C6	176.5(10)
O3	O6	C6	C1	-173.1(14)
C3	O6	C6	O3	2.3(9)
C3	O6	C6	C3	-0.0(4)
C6	O6	C3	O3	-176.5(14)
Fe1	C1	C4	Fe1	0.00(3)
Fe1	C1	C4	O4	-175.2(11)
Fe1	C1	C6	O6	-179.2(18)
O1	C1	C4	Fe1	176.6(11)
O1	C1	C4	O4	1.4(15)
O1	C1	C6	O6	5(2)
O4	C1	C4	Fe1	175.2(12)
O4	C1	C4	O4	-0.0(5)
C4	C1	C6	O6	-179.3(17)
C6	C1	C4	Fe1	0.1(16)
C6	C1	C4	O4	-175.1(14)
Fe1	C2	C5	O2	176.7(7)
O2	C2	C5	Fe1	-176.7(8)
O2	C2	C5	O5	1.1(10)
O5	C2	C5	O2	-1.1(11)
Fe1	C3	C6	Fe1	-0.00(3)
Fe1	C3	C6	O6	177.7(12)
O3	C3	C6	Fe1	-174.3(8)
O3	C3	C6	O6	3.4(13)
O6	C3	C6	Fe1	-177.7(13)
O6	C3	C6	O6	-0.0(6)
P1	C7	C8	C9	28.0(5)
Si2	C7	C8	C9	155.6(3)
C8	C9	C10	P1	36.1(5)
C8	C9	C10	Si4	-89.4(5)
Sn1	C23	C28	C27	-177.3(3)
C28	C23	C24	C25	-0.5(9)
C24	C25	C26	C27	0.4(10)
C26	C27	C28	C23	0.2(9)
Sn1	C29	C34	C33	-177.3(4)
C34	C29	C30	C31	0.6(10)
C30	C31	C32	C33	1.7(12)
C32	C33	C34	C29	1.0(11)
Sn1	C35	C40	C39	173.8(4)
C40	C35	C36	C37	-0.2(8)
C36	C37	C38	C39	0.1(9)
C38	C39	C40	C35	1.4(10)
C5	O2	C2	C5	0.0(18)
O6	O3	C3	C6	-2.7(8)
C3	O3	O6	C6	2.4(7)
O6	O3	C6	O6	0.0(8)
C6	O3	O6	C3	-2.4(9)
C3	O3	C6	Fe1	6.2(8)
C3	O3	C6	C3	-0.0(7)
C6	O3	C3	C6	-0.0(9)
O1	O4	C1	O1	0.0(5)
O1	O4	C4	O1	0.0(4)
C1	O4	C4	O1	1.1(11)
C4	O4	C1	Fe1	5.2(13)
C4	O4	C1	C4	-0.0(12)
O2	O5	C2	O2	-0.0(9)
O2	O5	C5	O2	-0.0(9)
C2	O5	C5	O2	2(2)
C5	O5	C2	Fe1	-4(2)
C5	O5	C2	C5	0.0(19)
O3	O6	C3	O3	0.0(4)
O3	O6	C6	O3	0.0(3)
O3	O6	C6	C3	-2.3(9)
C3	O6	C6	C1	-171(2)
C6	O6	C3	Fe1	-2.1(13)
C6	O6	C3	C6	-0.0(11)
Fe1	C1	C4	O1	-176.6(10)
Fe1	C1	C6	Fe1	0.00(3)
Fe1	C1	C6	C3	15.2(17)
O1	C1	C4	O1	0.0(5)
O1	C1	C6	Fe1	-175.9(14)
O1	C1	C6	C3	-160.7(17)
O4	C1	C4	O1	-1.4(15)
C4	C1	C6	Fe1	-0.1(18)
C4	C1	C6	C3	15(3)
C6	C1	C4	O1	-177(2)
Fe1	C2	C5	Fe1	-0.00(2)
Fe1	C2	C5	O5	177.8(10)
O2	C2	C5	O2	0.0(4)
O5	C2	C5	Fe1	-177.8(12)
O5	C2	C5	O5	0.0(6)
Fe1	C3	C6	O3	174.3(7)
Fe1	C3	C6	C1	-13.2(14)
O3	C3	C6	O3	0.0(4)
O3	C3	C6	C1	172.4(15)
O6	C3	C6	O3	-3.4(14)
O6	C3	C6	C1	169(3)
Si1	C7	C8	C9	-86.2(4)
C7	C8	C9	C10	-44.2(6)
C8	C9	C10	Si3	153.3(4)
Sn1	C23	C24	C25	177.1(4)
C24	C23	C28	C27	0.4(8)
C23	C24	C25	C26	0.1(10)
C25	C26	C27	C28	-0.6(10)
Sn1	C29	C30	C31	177.5(4)
C30	C29	C34	C33	-0.5(9)
C29	C30	C31	C32	-1.2(12)
C31	C32	C33	C34	-1.6(12)
Sn1	C35	C36	C37	-174.6(3)
C36	C35	C40	C39	-0.7(9)
C35	C36	C37	C38	0.5(9)
C37	C38	C39	C40	-1.1(10)

Reference

(1) E. H. Braye, W. Hübel, *Inorg. Synth.,* 1966, **8**, 178-181.

(2) S. Ishida, F. Hirakawa, T. Iwamoto, *J. Am. Chem. Soc.*, 2011, **133**, 12968-12971.

(3) (a) E. M. Schubert, *J. Chem. Edu.*, 1992, **69**, 62; (b) C. Piguet, *J. Chem. Edu.*, 1997, **74**, 815-816; (c) G. A. Bain, J. F. Berry, *J. Chem. Edu.*, 2008, **85**, 532-536.

(4) Gaussian 09, Revision D.01, M. J. Frisch, G. W. Trucks, H. B. Schlegel, G. E. Scuseria, M. A. Robb, J. R. Cheeseman, G. Scalmani, V. Barone, B. Mennucci, G. A. Petersson, H. Nakatsuji, M. Caricato, X. Li, H. P. Hratchian, A. F. Izmaylov, J. Bloino, G. Zheng, J. L. Sonnenberg, M. Hada, M. Ehara, K. Toyota, R. Fukuda, J. Hasegawa, M. Ishida, T. Nakajima, Y. Honda, O. Kitao, H. Nakai, T. Vreven, J. A. Montgomery, Jr., J. E. Peralta, F. Ogliaro, M. Bearpark, J. J. Heyd, E. Brothers, K. N. Kudin, V. N. Staroverov, R. Kobayashi, J. Normand, K. Raghavachari, A. Rendell, J. C. Burant, S. S. Iyengar, J. Tomasi, M. Cossi, N. Rega, J. M. Millam, M. Klene, J. E. Knox, J. B. Cross, V. Bakken, C. Adamo, J. Jaramillo, R. Gomperts, R. E. Stratmann, O. Yazyev, A. J. Austin, R. Cammi, C. Pomelli, J. W. Ochterski, R. L. Martin, K. Morokuma, V. G. Zakrzewski, G. A. Voth, P. Salvador, J. J. Dannenberg, S. Dapprich, A. D. Daniels, Ö. Farkas, J. B. Foresman, J. V. Ortiz, J. Cioslowski, and D. J. Fox, Gaussian, Inc., Wallingford CT, 2009.

(5) (a) C. Adamo, V. Barone, *J. Chem. Phys.,* 1999, **110**, 6158-6170; (b) A. D. Becke, *J. Chem. Phys.,* 1993, **98**, 1372-1377; (c) C. Lee, W. Yang, R. G. Parr, *Phys. Rev. B*, 1988, **37**, 785-789; (d) T. Yanai, D. Tew, N. Handy, *Chem. Phys. Lett.*, 2004, **393**, 51-57.

(6) A. J. H. Wachters, *J. Chem. Phys.,* 1970, **52**, 1033-1036.

(7) P. J. Hay, *J. Chem. Phys.,* 1977, **66**, 4377-4384

(8) T. H. Dunning, Jr. P. J. Hay, in *Modern Theoretical Chemistry*, Ed. H. F. Schaefer III, Vol. 3 (Plenum, New York, 1977), pp 1-28.

(9) I. Mayer, *Int. J. Quantum Chem.,* 1983, **23**, 341-363.

(10) M. E. Casida, C. Jamorski, K. C. Casida, D. R. Salahub, *J. Chem. Phys.,* 1998, **108**, 4439-4449.

(11) SIR2008: M. C. Burla, R. Caliandro, M. Camalli, B. Carrozzini, G. L. Cascarano, L. De Caro, C. Giacovazzo, G. Polidori, D. Siliqi, R. Spagna, *J. Appl. Crystallogr*. 2007, **40**, 609-613.

(12) DIRDIF99: P. T. Beurskens, G. Admiraal, G. Beurskens, W. P. Bosman, R. de Gelder, R. Israel, J. M. M. Smits, The DIRDIF-99 program system; *Technical Report of the Crystallography Laboratory*; University of Nijmegen, Nijmegen, The Netherlands, 1999.

(13) D. T. Cromer, J. T. Waber, *International Tables for X-ray Crystallography*; Kynoch Press: Birmingham, U.K., 1974, *Vol. 4*.

(14) SHELX97: G.M. Sheldrick, *Acta Cryst.,* 2008, **A64**, 112-122.
